# Supplementary material for: Novel Matrine Derivatives as Potential Larvicidal Agents against Aedes albopictus: Synthesis, Biological Evaluation, and Mechanistic Analysis
Source: Molecules. 2023 Mar 29;28(7):3035. doi: 10.3390/molecules28073035 (PMC10096473; doi:10.3390/molecules28073035)
Supplement: Supplementary file 1 [file molecules-28-03035-s001.zip › molecules-2259036-supplementary.pdf]

Novel matrine derivatives as potential larvicidal agents against *Aedes albopictus*: Synthesis, biological evaluation, and mechanistic analysis

Song Ang [1, 2, †], Jinfeng Liang [1, 2, †], Wende Zheng [1, 2], Zhen Zhang [1, 2], Jinxuan Li [1, 2], Zhenping Yan [1, 2], Wing-Leung Wong [3], Kun Zhang [1, 2, *], Min Chen [1, 2, *], and Panpan Wu [1, 2, *]

[1] School of Biotechnology and Health Sciences, Wuyi University, Jiangmen, 529020, P. R. China

[2] International Healthcare Innovation Institute (Jiangmen), Jiangmen, 529040, P. R. China

[3] The State Key Laboratory of Chemical Biology and Drug Discovery, Department of Applied Biology and Chemical Technology, The Hong Kong Polytechnic University, Hung Hom, Kowloon, Hong Kong SAR, China

***** Correspondence: kzhang@gdut.edu.cn (K.Z.); cmin0501@outlook.com (M.C.); wyuchemwpp@126.com (P.W.); Tel.: +86 13822330019 (K.Z.); +86 18312066545 (M.C.); +86 18825179347(P.W.)

† These authors contributed equally to this work.

Data for **4c** ($C_{25}H_{37}ClN_4O_3S$): yield: 42%; white powder; mp: 130.2-132.0 °C; ^{1}H NMR (500 MHz, Chloroform-*d*) δ_H 7.83 – 7.77 (m, 2H), 7.51 – 7.45 (m, 2H), 3.72 – 3.61 (m, 2H), 3.60 – 3.50 (m, 3H), 3.51 – 3.44 (m, 2H), 3.18 (t, J = 11.8 Hz, 1H), 2.68 – 2.62 (m, 1H), 2.61 – 2.56 (m, 1H), 2.42 – 2.39 (m, 1H), 2.32 – 2.23 (m, 1H), 2.05 – 2.01 (m, 1H), 1.97 – 1.94 (m, 2H), 1.85 – 1.82 (m, 7H), 1.70 – 1.66 (m, 1H), 1.48 – 1.43 (m, 5H), 1.41 – 1.32 (m, 4H), 1.32 – 1.24 (m, 1H); ^{13}C NMR (126 MHz, Chloroform-*d*) δ_C 171.62, 138.72, 138.66, 129.11, 129.05, 129.00, 62.89, 57.31, 57.25, 56.68, 56.64, 45.70, 45.26, 41.56, 41.29, 39.18, 34.47, 33.28, 27.86, 20.77, 20.69. HRMS (ESI): $C_{25}H_{38}ClN_4O_3S$ (509.2348) $[M+H]^+$= 509.2345.

Data for **4d** ($C_{26}H_{39}ClN_4O_3S$): yield: 81%; white powder; mp: 131.1-133.4 °C; ^{1}H NMR (500 MHz, Chloroform-*d*) δ_H 7.79 (d, J = 8.7 Hz, 2H), 7.46 (d, J = 8.6 Hz, 2H), 3.70 – 3.62 (m, 1H), 3.62 – 3.55 (m, 1H), 3.55 – 3.43 (m, 4H), 3.17 (t, J = 11.6 Hz, 1H), 2.64 (d, J = 11.1 Hz, 1H), 2.57 (d, J = 11.3 Hz, 1H), 2.46 – 2.32 (m, 5H), 2.31 (s, 3H), 2.28 – 2.18 (m, 1H), 2.07 – 1.88 (m, 3H), 1.87 – 1.77 (m, 5H), 1.76 (d, J = 16.3 Hz, 1H), 1.70 – 1.61 (m, 1H), 1.51 – 1.42 (m, 1H), 1.41 – 1.37 (m, 2H), 1.45 – 1.31 (m, 3H), 1.34 – 1.23 (m, 1H); ^{13}C NMR (126 MHz, Chloroform-*d*) δ_C 171.35, 138.64, 138.27, 129.09, 128.98, 62.91, 57.34, 56.68, 56.63, 55.12, 54.67, 47.49, 45.97, 45.39, 41.34, 39.18, 34.44, 33.33, 31.08, 27.88, 20.81, 20.73. HRMS (ESI): $C_{26}H_{40}ClN_4O_3S$ (523.2504) $[M+H]^+$=523.2497.

Data for **4e** ($C_{25}H_{34}ClN_3O_4S_2$): yield: 75%; white powder; mp: 120.5-123.0 °C; ^{1}H NMR (500 MHz, Chloroform-*d*) δ_H 7.91 – 7.73 (m, 2H), 7.55 – 7.44 (m, 2H), 6.13 (dd, J = 20.1, 6.6 Hz, 1H), 4.64 – 4.51 (m, 1H), 3.64 – 3.52 (m, 1H), 3.55 – 3.46 (m, 1H), 3.41 – 3.31 (m, 1H), 3.30 – 3.22 (m, 1H), 3.26 – 3.13 (m, 1H), 2.92 – 2.82 (m, 1H), 2.66 – 2.60 (m, 1H), 2.60 – 2.53 (m, 1H), 2.36 – 2.16 (m, 2H), 2.09 – 1.93 (m, 2H), 1.96 – 1.79 (m, 6H), 1.80 – 1.64 (m, 3H), 1.56 – 1.48 (m, 1H), 1.51 – 1.41 (m, 1H), 1.43 – 1.38 (m, 1H), 1.41 – 1.34 (m, 3H), 1.37 – 1.24 (m, 1H); ^{13}C NMR (126 MHz, Chloroform-*d*) δ_C 205.43, 173.24, 138.73, 138.10, 129.15, 129.01, 62.86, 59.32, 57.54, 57.12, 56.63, 47.34, 39.34, 36.14, 34.32, 31.69, 30.93, 28.09, 27.89, 27.52, 21.47, 20.81, 20.71. HRMS (ESI): $C_{25}H_{35}ClN_3O_4S_2$ (540.1752) $[M+H]^+$=540.1747.

Data for **4f** ($C_{24}H_{31}ClN_4O_3S_2$): yield: 55%; white powder; mp: 156.9-158.5 °C;

^{1}H NMR (500 MHz, Chloroform-*d*) δ_H 12.49 (d, *J* = 8.6 Hz, 1H), 7.78 (d, *J* = 8.7 Hz, 2H), 7.53 (d, *J* = 3.6 Hz, 1H), 7.43 (d, *J* = 8.6 Hz, 2H), 7.01 (d, *J* = 3.6 Hz, 1H), 3.63 – 3.55 (m, 1H), 3.51 (dd, *J* = 12.3, 5.9 Hz, 1H), 3.21 (dd, *J* = 12.4, 10.8 Hz, 1H), 2.67 – 2.50 (m, 5H), 2.08 – 2.00 (m, 1H), 2.00 – 1.92 (m, 1H), 1.91 (dd, *J* = 10.3, 5.0 Hz, 1H), 1.90 – 1.75 (m, 5H), 1.57 – 1.43 (m, 1H), 1.46 – 1.34 (m, 5H), 1.37 – 1.27 (m, 1H), 1.27 (d, *J* = 1.9 Hz, 1H); ^{13}C NMR (126 MHz, Chloroform-*d*) δ_C 171.03, 160.03, 138.64, 138.45, 136.48, 129.03, 128.94, 113.43, 62.78, 57.19, 56.63, 47.24, 39.33, 35.87, 34.39, 31.09, 28.08, 27.89, 20.84, 20.72, 20.60. HRMS (ESI): $C_{24}H_{32}ClN_4O_3S_2$ (523.1599) $[M+H]^+$=523.1606.

Data for **4g** ($C_{31}H_{47}ClN_4O_3S$): yield: 84%; white powder; mp: 214.4-215.5 °C; ^{1}H NMR (500 MHz, Chloroform-*d*) δ_H 7.80 (dd, *J* = 8.7, 2.3 Hz, 2H), 7.46 (dd, *J* = 8.6, 2.0 Hz, 2H), 4.70 – 4.63 (m, 1H), 3.97 – 3.88 (m, 1H), 3.55 – 3.46 (m, 2H), 3.17 (t, *J* = 11.6 Hz, 1H), 3.03 – 2.93 (m, 1H), 2.63 (d, *J* = 11.3 Hz, 1H), 2.60 – 2.52 (m, 1H), 2.52 (t, *J* = 5.1 Hz, 5H), 2.52 – 2.45 (m, 1H), 2.40 – 2.31 (m, 1H), 2.30 – 2.20 (m, 1H), 2.01 (t, *J* = 2.9 Hz, 1H), 2.00 – 1.93 (m, 1H), 1.93 – 1.89 (m, 1H), 1.92 – 1.71 (m, 8H), 1.68 – 1.51 (m, 6H), 1.52 – 1.24 (m, 10H); ^{13}C NMR (126 MHz, Chloroform-*d*) δ_C 171.10, 138.62, 138.30, 129.12, 128.95, 62.84, 62.69, 57.33, 56.69, 50.16, 47.39, 45.20, 41.27, 39.19, 34.41, 33.42, 31.26, 28.62, 28.52, 27.98, 27.90, 27.54, 27.45, 26.06, 24.54, 20.86, 20.76. HRMS (ESI): $C_{31}H_{48}ClN_4O_3S$ (591.3130) $[M+H]^+$=591.3120.

Data for **4h** ($C_{31}H_{41}ClN_4O_3S$): yield: 56%; white powder; mp: 170.6-172.1 °C;^{1}H NMR (500 MHz, Chloroform-*d*) δ_H 7.85 – 7.78 (m, 2H), 7.51 – 7.45 (m, 2H), 7.30 (dd, *J* = 8.9, 7.4 Hz, 2H), 6.95 (d, *J* = 8.2 Hz, 2H), 6.91 (d, *J* = 7.3 Hz, 1H), 3.86 – 3.78 (m, 1H), 3.78 – 3.72 (m, 1H), 3.72 – 3.60 (m, 2H), 3.57 – 3.49 (m, 2H), 3.25 – 3.10 (m, 3H), 3.17 (s, 1H), 2.65 (dd, *J* = 11.6, 3.5 Hz, 1H), 2.62 – 2.55 (m, 1H), 2.49 – 2.40 (m, 1H), 2.35 – 2.25 (m, 1H), 2.06 – 1.91 (m, 3H), 1.90 – 1.76 (m, 5H), 1.76 – 1.67 (m, 1H), 1.58 – 1.51 (m, 1H), 1.54 – 1.43 (m, 1H), 1.46 – 1.40 (m, 1H), 1.40 (d, *J* = 4.1 Hz, 2H), 1.37 (d, *J* = 2.6 Hz, 1H), 1.35 (d, *J* = 7.0 Hz, 1H), 1.29 (d, *J* = 15.1 Hz, 2H); ^{13}C NMR (126 MHz, Chloroform-*d*) δ_C 171.47, 151.00, 138.68, 138.19, 129.25, 129.13, 129.00, 120.46, 116.63, 62.86, 57.32, 56.71, 56.66, 49.83, 49.39,

47.48, 45.55, 41.46, 39.20, 34.43, 33.46, 31.15, 27.95, 27.91, 20.88, 20.84, 20.79. HRMS (ESI): $C_{31}H_{41}ClN_4O_3S$ (585.2660) $[M+H]^+$= 585.2668.

Data for **4i** ($C_{25}H_{36}BrN_3O_3S$): yield: 66%; white powder; mp: 161.5-163.5 °C; ^1H NMR (500 MHz, Chloroform-*d*) δ_H 7.78 – 7.71 (m, 2H), 7.66 – 7.60 (m, 2H), 3.58 – 3.47 (m, 2H), 3.46 (t, J = 6.9 Hz, 2H), 3.45 – 3.35 (m, 2H), 3.20 (dd, J = 12.2, 10.9 Hz, 1H), 2.67 – 2.60 (m, 1H), 2.61 – 2.55 (m, 1H), 2.32 – 2.23 (m, 1H), 2.22 – 2.13 (m, 1H), 2.02 (t, J = 3.1 Hz, 1H), 2.01 – 1.72 (m, 11H), 1.72 – 1.60 (m, 1H), 1.58 – 1.48 (m, 1H), 1.51 – 1.25 (m, 7H); ^{13}C NMR (126 MHz, Chloroform-*d*) δ_C 171.39, 139.04, 131.88, 129.22, 127.00, 62.84, 57.42, 56.70, 56.66, 47.25, 46.60, 45.60, 39.19, 34.68, 34.41, 31.39, 28.08, 27.94, 26.15, 24.44, 20.87, 20.77, 20.45. HRMS (ESI): $C_{25}H_{37}BrN_3O_3S$ (538.1734) $[M+H]^+$=538.1743.

Data for **4j** ($C_{25}H_{36}BrN_3O_4S$): yield: 64%; white powder; mp: 149.5-151.9 °C; ^1H NMR (500 MHz, Chloroform-*d*) δ_H 7.76 – 7.70 (m, 2H), 7.67 – 7.61 (m, 2H), 3.74 – 3.65 (m, 4H), 3.68 – 3.62 (m, 1H), 3.65 – 3.55 (m, 1H), 3.55 – 3.44 (m, 4H), 3.17 (dd, J = 12.1, 11.1 Hz, 1H), 2.68 – 2.61 (m, 1H), 2.61 – 2.54 (m, 1H), 2.43 – 2.33 (m, 1H), 2.29 – 2.20 (m, 1H), 2.03 (t, J = 3.1 Hz, 1H), 2.01 – 1.89 (m, 2H), 1.89 – 1.75 (m, 6H), 1.75 – 1.62 (m, 3H), 1.57 – 1.48 (m, 1H), 1.51 – 1.40 (m, 1H), 1.44 – 1.32 (m, 3H); ^{13}C NMR (126 MHz, Chloroform-*d*) δ_C 171.59, 138.69, 131.97, 129.23, 127.15, 66.91, 66.75, 62.83, 57.29, 56.69, 56.65, 47.44, 46.03, 41.87, 39.19, 34.42, 33.23, 31.10, 27.94, 27.90, 20.86, 20.77, 20.72. HRMS (ESI): $C_{25}H_{37}BrN_3O_4S$ (554.1682) $[M+H]^+$=554.1689.

Data for **4k** ($C_{25}H_{37}BrN_4O_3S$): yield: 35%; white powder; mp: 133.4-135.7 °C; ^1H NMR (500 MHz, Chloroform-*d*) δ_H 7.76 – 7.70 (m, 2H), 7.68 – 7.61 (m, 2H), 3.64 (t, J = 5.4 Hz, 1H), 3.60 – 3.44 (m, 6H), 3.18 (t, J = 11.6 Hz, 1H), 2.65 (d, J = 11.8 Hz, 1H), 2.59 (dd, J = 11.7, 3.4 Hz, 1H), 2.41 – 2.38 (m, 1H), 2.31 – 2.21 (m, 1H), 2.08 – 2.00 (m, 2H), 2.00 – 1.91 (m, 3H), 1.89 – 1.75 (m, 8H), 1.69 – 1.66 (m, 1H), 1.57 – 1.45 (m, 1H), 1.48 – 1.40 (m, 1H), 1.42 – 1.31 (m, 4H), 1.31 – 1.24 (m, 1H); ^{13}C NMR (126 MHz, Chloroform-*d*) δ_C 171.61, 138.80, 132.02, 131.97, 129.23, 127.13, 62.86, 57.33, 56.65, 47.48, 45.72, 45.26, 41.56, 39.19, 34.48, 33.30, 30.97, 27.89, 20.86, 20.78, 20.70. HRMS (ESI): $C_{25}H_{38}BrN_4O_3S$ (553.1843) $[M+H]^+$=553.1845.

Data for **4l** ($C_{26}H_{39}BrN_4O_3S$): yield: 70%; white powder; mp: 134.9-136.3 °C; ^{1}H NMR (500 MHz, Chloroform-*d*) δ_H 7.73 (d, *J* = 8.9 Hz, 2H), 7.63 (d, *J* = 8.4 Hz, 2H), 3.71 – 3.63 (m, 1H), 3.63 – 3.56 (m, 1H), 3.56 – 3.39 (m, 5H), 3.18 (t, *J* = 11.6 Hz, 1H), 2.64 (d, *J* = 11.0 Hz, 1H), 2.58 (d, *J* = 11.5 Hz, 1H), 2.46 – 2.32 (m, 4H), 2.31 (s, 3H), 2.28 – 2.18 (m, 1H), 2.04 – 1.95 (m, 2H), 1.97 – 1.88 (m, 1H), 1.88 – 1.72 (m, 7H), 1.70 – 1.51 (m, 2H), 1.52 – 1.43 (m, 1H), 1.43 – 1.36 (m, 2H), 1.38 – 1.33 (m, 1H), 1.36 – 1.24 (m, 1H); ^{13}C NMR (126 MHz, Chloroform-*d*) δ_C 171.34, 138.79, 131.96, 129.20, 127.12, 62.88, 57.35, 56.69, 56.64, 55.12, 54.67, 47.48, 45.96, 45.38, 41.33, 39.18, 34.45, 33.34, 31.10, 27.88, 20.80, 20.75. HRMS (ESI): $C_{26}H_{40}BrN_4O_3S$ (567.1999) $[M+H]^+$=567.2003.

Data for **4m** ($C_{25}H_{34}BrN_3O_4S_2$): yield: 51%; white powder; mp: 122.2-124.1 °C; ^{1}H NMR (500 MHz, Chloroform-*d*) δ_H 7.80 – 7.71 (m, 2H), 7.70 – 7.61 (m, 2H), 6.13 (dd, *J* = 18.7, 6.6 Hz, 1H), 4.64 – 4.51 (m, 1H), 3.78 (d, *J* = 1.2 Hz, 0H), 3.64 – 3.45 (m, 1H), 3.54 (s, 1H), 3.41 – 3.31 (m, 1H), 3.30 – 3.22 (m, 1H), 3.24 – 3.13 (m, 1H), 2.92 – 2.82 (m, 1H), 2.63 (d, *J* = 11.2 Hz, 1H), 2.56 (s, 1H), 2.36 – 2.10 (m, 2H), 2.09 – 1.93 (m, 2H), 1.84 (q, *J* = 7.7 Hz, 1H), 1.81 (s, 6H), 1.79 (s, 1H), 1.73 – 1.60 (m, 1H), 1.52 – 1.39 (m, 1H), 1.42 – 1.30 (m, 4H), 1.33 – 1.24 (m, 1H); ^{13}C NMR (126 MHz, Chloroform-*d*) δ_C 205.43, 173.22, 139.26, 131.99, 131.95, 129.25, 129.21, 127.22, 62.78, 59.33, 57.55, 57.14, 56.63, 47.27, 39.34, 36.07, 34.34, 31.70, 30.88, 28.03, 27.88, 27.51, 20.81, 20.70. HRMS (ESI): $C_{25}H_{35}BrN_3O_4S_2$ (584.1247) $[M+H]^+$=584.1240.

Data for **4n** ($C_{24}H_{31}BrN_4O_3S_2$): yield: 38%; white powder; mp: 157.6-159.3 °C; ^{1}H NMR (500 MHz, Chloroform-*d*) δ_H 12.42 (s, 1H), 7.74 – 7.68 (m, 2H), 7.62 – 7.56 (m, 2H), 7.53 (d, *J* = 3.6 Hz, 1H), 7.01 (d, *J* = 3.6 Hz, 1H), 3.59 (dd, *J* = 8.9, 5.0 Hz, 1H), 3.51 (dd, *J* = 12.4, 5.8 Hz, 1H), 3.21 (t, *J* = 11.6 Hz, 1H), 2.67 – 2.59 (m, 2H), 2.59 – 2.50 (m, 2H), 2.08 – 1.96 (m, 2H), 1.99 – 1.90 (m, 1H), 1.92 – 1.82 (m, 1H), 1.85 – 1.77 (m, 3H), 1.57 – 1.44 (m, 1H), 1.47 – 1.36 (m, 2H), 1.39 (s, 3H), 1.38 – 1.34 (m, 1H), 1.36 – 1.24 (m, 3H); ^{13}C NMR (126 MHz, Chloroform-*d*) δ_C 171.01, 159.96, 138.99, 136.51, 131.93, 129.12, 127.14, 113.43, 62.79, 57.20, 56.62, 47.26,

39.34, 35.86, 34.41, 31.06, 29.72, 28.06, 27.87, 20.82, 20.71, 20.62. HRMS (ESI): $C_{24}H_{32}BrN_4O_3S_2$ (567.1094) $[M+H]^+$= 567.1102.

Data for **4o** ($C_{31}H_{47}BrN_4O_3S$): yield: 51%; white powder; mp: 214.2-216.0 ℃; ^{1}H NMR (500 MHz, Chloroform-*d*) δ_H 7.80 – 7.69 (m, 2H), 7.66 – 7.59 (m, 2H), 4.75 – 4.66 (m, 1H), 3.99 – 3.91 (m, 1H), 3.54 – 3.39 (m, 2H), 3.21 – 3.12 (m, 1H), 3.05 – 2.94 (m, 2H), 2.85 – 2.76 (m, 1H), 2.72 – 2.65 (m, 4H), 2.65 (d, *J* = 11.2 Hz, 1H), 2.59 (d, *J* = 11.6 Hz, 1H), 2.56 – 2.46 (m, 1H), 2.41 – 2.18 (m, 1H), 2.07 – 2.01 (m, 1H), 2.00 (s, 2H), 1.98 – 1.88 (m, 2H), 1.90 – 1.75 (m, 3H), 1.78 – 1.67 (m, 2H), 1.70 – 1.57 (m, 1H), 1.57 – 1.51 (m, 1H), 1.51 – 1.48 (m, 1H), 1.48 (s, 4H), 1.48 – 1.36 (m, 2H), 1.36 – 1.21 (m, 8H); ^{13}C NMR (126 MHz, Chloroform-*d*) δ_C 171.16, 138.78, 131.99, 129.24, 127.16, 62.88, 62.52, 58.30, 57.32, 56.61, 49.65, 47.47, 45.02, 41.03, 39.16, 34.42, 33.38, 31.02, 29.70, 27.91, 27.86, 27.79, 26.76, 24.97, 23.96, 23.00, 20.76, 8.85, 8.24. HRMS (ESI): $C_{31}H_{48}BrN_4O_3S$ (635.2625) $[M+H]^+$= 635.2631.

Data for **4p** ($C_{31}H_{41}BrN_4O_3S$): yield: 49%; white powder; mp: 156.8-158.9 ℃; ^{1}H NMR (500 MHz, Chloroform-*d*) δ_H 7.77 – 7.71 (m, 2H), 7.67 – 7.61 (m, 2H), 7.34 – 7.27 (m, 2H), 6.96 (d, *J* = 1.2 Hz, 1H), 6.97 – 6.88 (m, 2H), 3.86 – 3.78 (m, 1H), 3.78 – 3.71 (m, 1H), 3.71 – 3.60 (m, 2H), 3.57 – 3.48 (m, 2H), 3.25 – 3.10 (m, 5H), 2.69 – 2.62 (m, 1H), 2.62 – 2.55 (m, 1H), 2.49 – 2.39 (m, 1H), 2.34 – 2.25 (m, 1H), 2.03 (t, *J* = 3.1 Hz, 1H), 2.01 – 1.91 (m, 2H), 1.89 – 1.76 (m, 6H), 1.75 – 1.66 (m, 1H), 1.58 – 1.48 (m, 1H), 1.51 – 1.43 (m, 1H), 1.45 – 1.38 (m, 2H), 1.37 (s, 1H), 1.37 – 1.29 (m, 1H), 1.29 – 1.26 (m, 1H); ^{13}C NMR (126 MHz, Chloroform-*d*) δ_C 171.45, 151.00, 138.74, 131.98, 129.25, 129.23, 127.15, 120.46, 116.63, 62.86, 57.34, 56.71, 56.66, 49.83, 49.39, 47.49, 45.55, 41.46, 39.20, 34.44, 33.45, 31.14, 27.95, 27.91, 20.88, 20.84, 20.79. HRMS (ESI): $C_{31}H_{42}BrN_4O_3S$ (629.2156) $[M+H]^+$= 629.2160.

Data for **5c** ($C_{25}H_{37}ClN_4O_4S$): yield: 63%; white powder; mp: 160.7-162.5 ℃; ^{1}H NMR (500 MHz, Chloroform-*d*) δ_H 7.80 – 7.73 (m, 2H), 7.55 – 7.46 (m, 2H), 4.74 – 4.71 (m, 1H), 4.37 – 4.34 (m, 1H), 3.65 – 3.51 (m, 3H), 3.50 – 3.39 (m, 2H), 3.25 (s, 1H), 3.23 – 3.18 (m, 5H), 2.90 – 2.78 (m, 3H), 2.56 (s, 1H), 2.40 (dd, *J* = 28.4, 15.4 Hz, 1H), 2.33 – 2.20 (m, 2H), 2.23 – 2.07 (m, 3H), 2.02 – 1.91 (m, 1H), 1.83 – 1.70 (m, 3H), 1.63 – 1.50 (m, 5H), 1.32 – 1.19 (m, 1H); ^{13}C NMR (126 MHz,

Chloroform-*d*) δ_C 171.26, 138.84, 138.70, 129.32, 128.58, 69.02, 68.52, 67.18, 56.67, 49.63, 46.39, 46.06, 45.59, 42.26, 38.49, 34.55, 33.00, 29.66, 25.74, 25.03, 20.07, 17.18, 17.09. HRMS (ESI): $C_{25}H_{38}ClN_4O_4S$ (525.2297) $[M+H]^+$=525.2291.

Data for **5d** ($C_{26}H_{39}ClN_4O_4S$): yield: 95%; light brown powder; mp: 164.4-166.9 °C; ^{1}H NMR (500 MHz, Chloroform-*d*) δ_H 7.79 – 7.72 (m, 2H), 7.53 – 7.45 (m, 2H), 4.65 – 4.61 (m, 1H), 4.29 – 4.26 (m, 1H), 3.66 – 3.52 (m, 3H), 3.50 – 3.38 (m, 2H), 3.31 – 3.27 (m, 1H), 3.25 – 3.22 (m, 4H), 3.27 – 3.18 (m, 1H), 2.50 (d, *J* = 11.5 Hz, 1H), 2.42 – 2.33 (m, 5H), 2.33 (d, *J* = 5.7 Hz, 1H), 2.29 (s, 3H), 2.28 – 2.18 (m, 2H), 2.17 – 2.12 (m, 1H), 2.14 – 2.05 (m, 2H), 1.99 – 1.89 (m, 1H), 1.84 – 1.68 (m, 3H), 1.60 – 1.45 (m, 1H), 1.26 – 1.23 (m, 1H), 1.16 – 1.07 (m, 1H); ^{13}C NMR (126 MHz, Chloroform-*d*) δ_C 171.11, 138.84, 138.72, 129.31, 128.54, 67.20, 56.63, 55.14, 54.70, 49.43, 46.03, 45.35, 44.55, 41.41, 38.36, 34.38, 32.97, 29.79, 25.60, 24.91, 20.03, 17.13, 17.04. HRMS (ESI): $C_{26}H_{40}ClN_4O_4S$ (539.2453) $[M+H]^+$=539.2444.

Data for **5e** ($C_{25}H_{34}ClN_3O_5S_2$): yield: 99%; white powder; mp: 153.4-155.3 °C; ^{1}H NMR (500 MHz, Chloroform-*d*) δ_H 7.79 – 7.72 (m, 2H), 7.52 – 7.45 (m, 2H), 5.06 (s, 1H), 4.46 – 4.36 (m, 1H), 3.78 – 3.63 (m, 1H), 3.42 (s, 1H), 3.40 – 3.29 (m, 1H), 3.28 – 3.21 (m, 1H), 3.16 (dd, *J* = 25.2, 6.0 Hz, 1H), 3.14 – 3.03 (m, 2H), 2.80 – 2.69 (m, 1H), 2.67 – 2.56 (m, 1H), 2.52 – 2.40 (m, 1H), 2.37 – 2.28 (m, 2H), 2.27 (s, 1H), 2.26 – 1.97 (m, 3H), 1.95 – 1.82 (m, 3H), 1.82 – 1.70 (m, 2H), 1.60 (d, *J* = 16.3 Hz, 1H), 1.58 – 1.50 (m, 4H), 1.52 – 1.37 (m, 0H), 1.32 – 1.23 (m, 1H), 1.22 – 1.10 (m, 1H); ^{13}C NMR (126 MHz, Chloroform-*d*) δ_C 205.12, 176.29, 173.37, 139.71, 138.86, 129.30, 128.42, 128.39, 68.51, 68.28, 59.20, 58.89, 56.62, 44.81, 39.18, 35.02, 31.31, 31.22, 27.38, 25.64, 25.20, 22.84, 20.99, 20.73, 17.05. HRMS (ESI): $C_{25}H_{35}ClN_3O_5S_2$ (556.1701) $[M+H]^+$=556.1694.

Data for **5f** ($C_{24}H_{31}ClN_4O_4S_2$): yield: 39%; white powder; mp: 178.8-180.6 °C; ^{1}H NMR (500 MHz, Chloroform-*d*) δ_H 7.77 – 7.69 (m, 2H), 7.48 – 7.42 (m, 3H), 6.96 (d, *J* = 3.6 Hz, 1H), 4.83 (s, 1H), 4.27 – 4.18 (m, 1H), 3.77 – 3.59 (m, 3H), 3.22 (s, 1H), 3.17 – 3.06 (m, 4H), 2.59 – 2.44 (m, 1H), 2.37 – 2.30 (m, 2H), 2.10 – 2.04 (m, 1H), 1.96 – 1.85 (m, 1H), 1.81 – 1.74 (m, 2H), 1.67 – 1.61 (m, 2H), 1.40 (t, *J* = 7.3 Hz,

4H), 1.27 – 1.25 (m, 2H); ^{13}C NMR (126 MHz, Deuterium Oxide) δ_C 173.83, 160.97, 139.64, 134.34, 129.41, 128.82, 126.83, 115.04, 67.25, 66.27, 65.89, 54.84, 46.48, 35.61, 35.02, 31.31, 30.97, 23.60, 23.51, 18.22, 15.81, 15.77. HRMS (ESI): $C_{24}H_{32}ClN_4O_4S_2$ (539.1548) $[M+H]^+$=539.1541.

Data for **5g** ($C_{31}H_{47}ClN_4O_4S$): yield: 75%; white powder; mp: 217.6-219.0 ℃; ^{1}H NMR (500 MHz, Chloroform-*d*) δ_H 7.80 – 7.74 (m, 2H), 7.51 – 7.45 (m, 2H), 4.95 – 4.88 (m, 1H), 4.68 – 4.54 (m, 2H), 3.96 – 3.88 (m, 1H), 3.67 – 3.59 (m, 1H), 3.07 (d, *J* = 6.8 Hz, 6H), 3.00 – 2.91 (m, 1H), 2.70 – 2.59 (m, 1H), 2.54 – 2.39 (m, 9H), 2.39 – 2.31 (m, 1H), 2.25 – 2.07 (m, 5H), 2.03 – 1.91 (m, 1H), 1.91 – 1.79 (m, 1H), 1.77 (s, 1H), 1.77 – 1.66 (m, 2H), 1.66 – 1.53 (m, 5H), 1.56 – 1.45 (m, 2H), 1.48 – 1.32 (m, 3H); ^{13}C NMR (126 MHz, Chloroform-*d*) δ_C 171.14, 138.76, 138.55, 129.29, 128.61, 69.78, 69.23, 66.87, 62.61, 56.69, 50.26, 45.23, 41.35, 38.78, 38.75, 35.00, 33.25, 29.48, 29.41, 28.89, 28.74, 27.79, 26.36, 26.11, 25.28, 24.73, 20.02, 17.26, 17.18. HRMS (ESI): $C_{31}H_{48}ClN_4O_4S$ (607.3079) $[M+H]^+$=607.3070.

Data for **5h** ($C_{31}H_{41}ClN_4O_4S$): yield: 98%; white powder; mp: 183.3~185.9 ℃; ^{1}H NMR (500 MHz, Chloroform-*d*) δ_H 7.80 – 7.73 (m, 2H), 7.54 – 7.46 (m, 2H), 7.34 – 7.25 (m, 2H), 6.96 (d, *J* = 1.2 Hz, 1H), 6.97 – 6.87 (m, 2H), 4.50 (d, *J* = 10.6 Hz, 1H), 4.23 (t, *J* = 12.0 Hz, 1H), 3.85 – 3.77 (m, 1H), 3.77 – 3.66 (m, 1H), 3.69 – 3.59 (m, 4H), 3.26 – 3.19 (m, 1H), 3.19 (s, 2H), 3.19 – 3.12 (m, 1H), 3.14 – 3.02 (m, 3H), 2.46 – 2.34 (m, 2H), 2.29 – 2.19 (m, 2H), 2.19 – 2.12 (m, 2H), 2.12 – 2.01 (m, 1H), 1.86 – 1.72 (m, 4H), 1.65 – 1.58 (m, 2H), 1.58 (s, 1H), 1.55 (d, *J* = 5.3 Hz, 1H), 1.29 (d, *J* = 15.8 Hz, 2H); ^{13}C NMR (126 MHz, Chloroform-*d*) δ_C 171.33, 150.97, 139.08, 129.39, 129.26, 128.65, 120.49, 116.62, 68.03, 67.96, 67.43, 56.59, 49.80, 49.56, 49.36, 45.48, 41.47, 38.28, 34.31, 33.09, 29.86, 25.63, 24.91, 22.64, 19.94, 17.07, 17.01. HRMS (ESI): $C_{31}H_{42}ClN_4O_4S$ (601.2610) $[M+H]^+$=601.2597.

Data for **5i** ($C_{25}H_{36}BrN_3O_4S$): yield: 78%; white powder; mp: 191.0-192.9 ℃; ^{1}H NMR (500 MHz, Chloroform-*d*) δ_H 7.73 – 7.67 (m, 2H), 7.70 – 7.60 (m, 2H), 4.96 – 4.88 (m, 1H), 4.57 (t, *J* = 11.8 Hz, 1H), 3.64 (dd, *J* = 11.1, 5.0 Hz, 1H), 3.48 – 3.38 (m, 3H), 3.38 (dd, *J* = 7.3, 2.8 Hz, 1H), 3.13 – 3.01 (m, 5H), 2.95 – 2.92 (m, 1H), 2.72 – 2.60 (m, 1H), 2.55 – 2.42 (m, 1H), 2.50 – 2.46 (m, 1H), 2.32 – 2.23 (m, 1H), 2.22 –

2.18 (m, 1H), 2.23 – 2.08 (m, 3H), 2.04 – 1.90 (m, 2H), 1.89 – 1.81 (m, 2H), 1.84 – 1.76 (m, 1H), 1.78 – 1.64 (m, 2H), 1.61 – 1.45 (m, 4H); ^{13}C NMR (126 MHz, Chloroform-*d*) δ_C 171.48, 139.23, 132.22, 128.73, 127.23, 69.65, 69.09, 67.04, 56.79, 50.09, 46.61, 45.60, 38.82, 35.05, 34.51, 29.46, 26.14, 26.07, 25.26, 24.44, 19.65, 17.25, 17.17. HRMS (ESI): $C_{25}H_{37}BrN_3O_4S$ (554.1683) $[M+H]^+$=554.1679.

Data for **5j** ($C_{25}H_{36}BrN_3O_5S$): yield: 96%; white powder; mp: 181.4-183.6 °C; ^{1}H NMR (500 MHz, Chloroform-*d*) δ_H 7.69 (d, *J* = 8.8 Hz, 2H), 7.66 (d, *J* = 8.8 Hz, 2H), 4.79 – 4.76 (m, 1H), 4.48 – 4.39 (m, 1H), 3.73 – 3.53 (m, 7H), 3.53 – 3.42 (m, 2H), 3.26 (d, *J* = 11.9 Hz, 2H), 3.13 – 3.10 (m, 3H), 2.62 (d, *J* = 13.9 Hz, 1H), 2.43 (dd, *J* = 16.2, 12.6 Hz, 1H), 2.39 – 2.29 (m, 1H), 2.27 – 2.19 (m, 1H), 2.21 – 2.10 (m, 4H), 2.06 – 1.96 (m, 1H), 1.85 – 1.69 (m, 4H), 1.61 – 1.49 (m, 2H), 1.26 (s, 1H); ^{13}C NMR (126 MHz, Chloroform-*d*) δ_C 171.55, 138.89, 132.31, 128.70, 127.43, 69.10, 68.60, 67.29, 66.90, 66.75, 56.63, 49.87, 45.98, 41.87, 38.59, 34.72, 32.99, 29.56, 25.89, 25.13, 19.93, 17.18, 17.10. HRMS (ESI): $C_{25}H_{37}BrN_3O_5S$ (570.1632) $[M+H]^+$=570.1625.

Data for **5k** ($C_{25}H_{37}BrN_4O_4S$): yield: 69%; white powder; mp: 164.2-165.9 °C; ^{1}H NMR (500 MHz, Chloroform-*d*) δ_H 7.72 – 7.68 (m, 2H), 7.67 – 7.64 (m, 2H), 4.74 (s, 1H), 4.37 (s, 1H), 3.66 – 3.51 (m, 3H), 3.50 – 3.38 (m, 2H), 3.22 – 3.18 (m, 6H), 2.90 – 2.78 (m, 3H), 2.56 (s, 1H), 2.40 (s, 1H), 2.28 – 2.15 (m, 1H), 2.17 – 2.07 (m, 3H), 1.99 – 1.96 (m, 1H), 2.02 – 1.91 (m, 1H), 1.83 – 1.70 (m, 2H), 1.75 (s, 2H), 1.62 – 1.54 (m, 3H), 1.54 – 1.49 (m, 1H), 1.32 – 1.24 (m, 1H); ^{13}C NMR (126 MHz, Chloroform-*d*) δ_C 171.29, 139.21, 132.28, 128.69, 127.34, 68.56, 67.17, 56.69, 53.47, 49.70, 46.46, 46.12, 45.64, 42.32, 38.54, 34.61, 33.02, 29.62, 25.77, 25.05, 20.07, 17.18, 17.09. HRMS (ESI): $C_{25}H_{38}BrN_4O_4S$ (569.1792) $[M+H]^+$=569.1780.

Data for **5l** ($C_{26}H_{39}BrN_4O_4S$): yield: 83%; white powder; mp: 160.8-162.7 °C; ^{1}H NMR (500 MHz, Chloroform-*d*) δ_H 7.72 – 7.66 (m, 2H), 7.68 – 7.62 (m, 2H), 4.74 (s, 1H), 4.37 (s, 1H), 3.69 – 3.59 (m, 1H), 3.62 – 3.53 (m, 2H), 3.51 – 3.40 (m, 2H), 3.25 – 3.22 (m, 2H), 3.25 – 3.13 (m, 3H), 2.58 – 2.50 (m, 1H), 2.45 – 2.32 (m, 4H), 2.30 (s, 3H), 2.29 – 2.19 (m, 1H), 2.19 – 2.07 (m, 3H), 2.00 – 1.90 (m, 1H), 1.82 – 1.68 (m, 3H), 1.62 – 1.48 (m, 5H), 1.32 – 1.18 (m, 1H), 1.17 – 1.08 (m, 1H); ^{13}C NMR (126

MHz, Chloroform-*d*) δ_C 171.18, 139.30, 132.28, 128.66, 127.33, 67.10, 56.68, 55.17, 54.72, 46.05, 45.38, 44.76, 41.42, 38.52, 34.58, 33.03, 29.69, 25.74, 25.03, 20.07, 17.18, 17.09. HRMS (ESI): $C_{26}H_{40}BrN_4O_4S$ (583.1948) $[M+H]^+$=583.1941.

Data for **5m** ($C_{25}H_{34}BrN_3O_5S_2$): yield: 78%; white powder; mp: 149.4-151.6 °C; ^{1}H NMR (500 MHz, Chloroform-*d*) δ_H 7.71 – 7.63 (m, 4H), 4.69 – 4.64 (m, 1H), 4.66 – 4.48 (m, 1H), 4.16 – 4.07 (m, 1H), 3.62 – 3.52 (m, 1H), 3.40 – 3.27 (m, 6H), 3.30 – 3.21 (m, 1H), 2.71 – 2.62 (m, 1H), 2.34 – 2.28 (m, 2H), 2.31 – 2.24 (m, 1H), 2.23 – 2.12 (m, 3H), 2.11 – 2.03 (m, 1H), 1.79 (s, 3H), 1.77 (d, *J* = 13.3 Hz, 1H), 1.68 – 1.59 (m, 3H), 1.55 – 1.40 (m, 2H), 1.36 – 1.23 (m, 1H); ^{13}C NMR (126 MHz, Deuterium Oxide) δ_C 209.50, 176.60, 175.97, 135.67, 132.50, 128.91, 128.18, 67.43, 66.34, 65.97, 59.14, 58.42, 55.19, 46.81, 36.03, 35.24, 31.35, 29.97, 27.50, 23.70, 23.55, 20.47, 20.34, 19.63, 15.94. HRMS (ESI): $C_{25}H_{35}BrN_3O_5S_2$ (600.1196) $[M+H]^+$=600.1191.

Data for **5n** ($C_{24}H_{31}BrN_4O_4S_2$): yield: 49%; white powder; mp: 185.8-188.3 °C; ^{1}H NMR (500 MHz, Chloroform-*d*) δ_H 7.71 – 7.59 (m, 4H), 7.45 (d, *J* = 3.6 Hz, 1H), 6.96 (d, *J* = 3.4 Hz, 1H), 4.99 (s, 1H), 4.30 (s, 1H), 3.71 – 3.64 (m, 1H), 3.61 – 3.42 (m, 2H), 3.23 – 3.19 (m, 1H), 3.15 – 3.12 (m, 2H), 2.60 – 2.50 (m, 1H), 2.44 (dd, *J* = 14.6, 6.6 Hz, 1H), 2.35 (s, 2H), 2.11 – 2.02 (m, 2H), 1.92 – 1.88 (m, 2H), 1.78 (s, 2H), 1.70 – 1.49 (m, 3H), 1.42 – 1.24 (m, 3H); ^{13}C NMR (126 MHz, Deuterium Oxide) δ_C 173.82, 160.92, 135.10, 132.42, 128.83, 128.26, 127.17, 115.04, 67.31, 66.31, 65.93, 58.42, 54.94, 46.54, 35.66, 34.99, 31.19, 31.08, 23.61, 23.50, 18.26, 15.83. HRMS (ESI): $C_{24}H_{32}BrN_4O_4S_2$ (583.1043) $[M+H]^+$=583.1036.

Data for **5o** ($C_{31}H_{47}BrN_4O_4S$): yield: 79%; white powder; mp: 218.4-219.8 °C; ^{1}H NMR (500 MHz, Chloroform-*d*) δ_H 7.73 – 7.62 (m, 4H), 4.84 – 4.80 (m, 1H), 4.67 – 4.60 (m, 1H), 4.47 (t, *J* = 12.0 Hz, 1H), 3.89 (dd, *J* = 12.7, 8.6 Hz, 1H), 3.69 – 3.57 (m, 1H), 3.27 – 3.16 (m, 1H), 3.14 (d, *J* = 19.3 Hz, 4H), 3.03 (s, 3H), 3.00 – 2.91 (m, 1H), 2.64 – 2.58 (m, 1H), 2.54 – 2.48 (m, 4H), 2.48 – 2.44 (m, 1H), 2.47 – 2.40 (m, 1H), 2.36 – 2.27 (m, 1H), 2.27 (s, 1H), 2.25 – 2.15 (m, 1H), 2.15 – 2.08 (m, 2H), 2.02 – 1.92 (m, 1H), 1.92 – 1.85 (m, 1H), 1.88 – 1.74 (m, 1H), 1.76 – 1.71 (m, 2H), 1.74 – 1.65 (m, 1H), 1.67 – 1.48 (m, 7H), 1.47 – 1.32 (m, 3H), 1.13 (t, 1H); ^{13}C NMR (126

MHz, Chloroform-*d*) δ_C 171.03, 139.21, 132.27, 128.70, 127.30, 69.39, 68.86, 67.00, 62.64, 62.62, 56.71, 53.46, 50.25, 50.23, 45.20, 45.04, 41.34, 41.30, 38.64, 35.29, 33.23, 33.14, 29.57, 28.81, 28.66, 27.73, 27.68, 26.26, 26.23, 25.92, 25.14, 24.65, 20.08, 17.22, 17.13, 15.51. HRMS (ESI): $C_{31}H_{48}BrN_4O_4S$ (651.2574) $[M+H]^+$=651.2562.

Data for **5p** ($C_{31}H_{41}BrN_4O_4S$): yield: 89%; white powder; mp: 185.3-187.1 °C; ^{1}H NMR (500 MHz, Chloroform-*d*) δ_H 7.72 – 7.66 (m, 2H), 7.66 (d, J = 8.7 Hz, 2H), 7.33 – 7.24 (m, 2H), 6.99 – 6.90 (m, 3H), 6.95 – 6.87 (m, 1H), 4.67 (d, J = 10.6 Hz, 1H), 4.35 (t, J = 11.5 Hz, 1H), 3.84 – 3.75 (m, 1H), 3.77 – 3.68 (m, 1H), 3.70 – 3.57 (m, 3H), 3.41 – 3.28 (m, 2H), 3.24 – 3.03 (m, 5H), 2.58 (s, 1H), 2.45 – 2.33 (m, 2H), 2.28 – 2.17 (m, 2H), 2.18 – 2.09 (m, 2H), 2.07 – 1.96 (m, 1H), 1.94 (s, 1H), 1.86 – 1.70 (m, 4H), 1.62 – 1.57 (m, 1H), 1.57 (s, 1H), 1.58 – 1.50 (m, 1H), 1.37 – 1.22 (m, 1H); ^{13}C NMR (126 MHz, Chloroform-*d*) δ_C 171.35, 150.97, 138.79, 132.33, 129.25, 128.71, 127.47, 120.46, 116.61, 68.66, 68.14, 67.44, 56.61, 49.80, 49.35, 45.48, 41.45, 38.45, 34.54, 33.15, 29.70, 25.77, 25.03, 22.75, 19.99, 17.15. HRMS (ESI): $C_{31}H_{42}BrN_4O_4S$ (645.2105) $[M+H]^+$=645.2093.

Data for **6c** ($C_{26}H_{36}ClN_5O_3S$): yield: 96%; white powder; mp: 133.9-136.1 °C; ^{1}H NMR (500 MHz, Chloroform-*d*) δ_H 7.84 – 7.77 (m, 2H), 7.54 – 7.50 (m, 2H), 4.08 – 4.00 (m, 1H), 3.70 (h, J = 5.2 Hz, 4H), 3.68 – 3.57 (m, 3H), 3.61 – 3.49 (m, 2H), 3.11 (dd, J = 15.2, 12.2 Hz, 1H), 2.68 – 2.59 (m, 2H), 2.45 – 2.28 (m, 4H), 2.10 – 1.97 (m, 1H), 1.82 – 1.57 (m, 5H), 1.60 – 1.41 (m, 5H), 1.40 – 1.19 (m, 4H); ^{13}C NMR (126 MHz, Chloroform-*d*) δ_C 171.20, 139.31, 139.27, 129.65, 128.52, 116.29, 64.49, 57.17, 51.57, 50.93, 45.41, 44.82, 43.34, 43.14, 42.49, 41.44, 40.87, 32.30, 26.47, 25.17, 24.50, 24.10, 23.85, 22.80. HRMS (ESI): $C_{26}H_{37}ClN_5O_3S$ (534.2300) $[M+H]^+$=534.2294.

Data for **6d** ($C_{27}H_{38}ClN_5O_3S$): yield: 48%; white powder; mp: 133.1-134.7 °C; ^{1}H NMR (500 MHz, Chloroform-*d*) δ_H 7.83 – 7.77 (m, 2H), 7.54 – 7.48 (m, 2H), 4.07 – 3.99 (m, 1H), 3.72 – 3.62 (m, 2H), 3.64 – 3.56 (m, 1H), 3.55 – 3.43 (m, 2H), 3.15 – 3.05 (m, 1H), 2.68 – 2.59 (m, 2H), 2.48 – 2.39 (m, 5H), 2.35 (s, 3H), 2.39 – 2.29 (m, 3H), 2.32 – 2.22 (m, 1H), 2.07 – 1.93 (m, 2H), 1.84 – 1.76 (m, 1H), 1.79 – 1.37 (m,

8H), 1.37 – 1.23 (m, 2H); ^{13}C NMR (126 MHz, Chloroform-*d*) δ_C 170.87, 139.27, 139.23, 129.62, 128.53, 116.27, 64.52, 57.18, 54.62, 54.27, 51.57, 45.79, 45.43, 45.32, 44.67, 43.12, 42.52, 40.72, 32.22, 26.47, 25.14, 24.48, 24.09, 23.84, 22.93. HRMS (ESI): $C_{27}H_{39}ClN_5O_3S$ (548.2457) $[M+H]^+$=548.2448.

Data for **6e** ($C_{26}H_{33}ClN_4O_4S_2$): yield: 78%; white powder; mp: 122.5-124.8 °C; ^{1}H NMR (500 MHz, Chloroform-*d*) δ_H 7.83 – 7.78 (m, 2H), 7.55 – 7.50 (m, 2H), 6.08 (dd, *J* = 15.2, 6.4 Hz, 1H), 4.63 – 4.49 (m, 1H), 4.07 – 3.98 (m, 1H), 3.66 – 3.58 (m, 1H), 3.41 – 3.32 (m, 1H), 3.30 – 3.23 (m, 1H), 3.16 – 3.06 (m, 1H), 2.96 – 2.82 (m, 1H), 2.67 – 2.61 (m, 1H), 2.64 – 2.52 (m, 1H), 2.45 – 2.14 (m, 5H), 2.13 – 1.88 (m, 2H), 1.83 – 1.70 (m, 1H), 1.73 – 1.65 (m, 1H), 1.65 – 1.56 (m, 1H), 1.59 – 1.50 (m, 4H), 1.52 – 1.47 (m, 2H), 1.49 – 1.23 (m, 3H); ^{13}C NMR (126 MHz, Chloroform-*d*) δ_C 205.41, 172.98, 139.32, 139.22, 129.67, 128.51, 116.22, 64.49, 59.34, 56.89, 51.57, 50.92, 45.39, 43.16, 42.51, 35.39, 31.82, 27.54, 25.94, 25.11, 24.51, 24.09, 23.29, 23.08. HRMS (ESI): $C_{26}H_{34}ClN_4O_4S_2$ (565.1705) $[M+H]^+$=565.1697.

Data for **6f** ($C_{25}H_{30}ClN_5O_3S_2$): yield: 98%; white powder; mp: 155.2-157.3 °C; ^{1}H NMR (500 MHz, Chloroform-*d*) δ_H 12.12 (s, 1H), 7.83 – 7.75 (m, 2H), 7.52 – 7.47 (m, 2H), 7.46 (d, *J* = 3.6 Hz, 1H), 7.02 (d, *J* = 3.6 Hz, 1H), 4.09 – 4.01 (m, 1H), 3.68 – 3.61 (m, 1H), 3.10 (dd, *J* = 15.3, 11.9 Hz, 1H), 2.67 – 2.59 (m, 3H), 2.56 (t, *J* = 7.2 Hz, 2H), 2.43 – 2.29 (m, 2H), 2.17 – 2.05 (m, 1H), 1.85 – 1.75 (m, 2H), 1.71 (s, 1H), 1.77 – 1.66 (m, 1H), 1.68 – 1.59 (m, 1H), 1.61 – 1.52 (m, 2H), 1.53 (s, 1H), 1.54 – 1.42 (m, 2H), 1.41 – 1.23 (m, 2H); ^{13}C NMR (126 MHz, Chloroform-*d*) δ_C 170.51, 159.85, 139.33, 139.23, 136.35, 129.63, 128.47, 116.26, 113.52, 64.51, 56.97, 51.57, 50.94, 45.38, 43.16, 42.58, 35.13, 26.06, 25.14, 24.50, 24.09, 23.86, 22.71. HRMS(ESI): $C_{25}H_{31}ClN_5O_3S_2$ (546.1406) $[M-H]^-$=546.1407.

Data for **6g** ($C_{32}H_{46}ClN_5O_3S$): yield: 98%; white powder; mp: 206.7-208.4 °C; ^{1}H NMR (500 MHz, Chloroform-*d*) δ_H 7.82 – 7.76 (m, 2H), 7.55 – 7.47 (m, 2H), 4.78 – 4.69 (m, 1H), 4.05 – 3.98 (m, 1H), 3.97 – 3.89 (m, 1H), 3.64 – 3.56 (m, 1H), 3.14 – 3.04 (m, 2H), 3.06 – 2.95 (m, 1H), 2.93 – 2.86 (m, 1H), 2.89 (s, 1H), 2.78 (s, 5H), 2.67 – 2.58 (m, 2H), 2.57 – 2.47 (m, 1H), 2.38 (dd, *J* = 12.5, 3.4 Hz, 1H), 2.36 – 2.22 (m, 3H), 2.13 (s, 1H), 2.07 – 1.93 (m, 3H), 1.83 (s, 5H), 1.80 – 1.68 (m, 1H), 1.68 –

1.53 (m, 4H), 1.56 – 1.46 (m, 3H), 1.40 – 1.23 (m, 4H); ^{13}C NMR (126 MHz, Chloroform-*d*) δ_C 170.72, 139.30, 139.25, 129.61, 128.57, 128.53, 116.27, 64.53, 63.13, 57.22, 51.58, 50.93, 49.89, 45.72, 45.47, 45.41, 44.75, 43.14, 42.54, 40.88, 32.35, 27.79, 26.49, 25.15, 24.50, 24.10, 23.86, 23.63, 23.06, 22.97, 8.78. HRMS (ESI): $C_{32}H_{45}ClN_5O_3S$ (614.2937) $[M-H]^-$=614.2938.

Data for **6h** ($C_{32}H_{40}ClN_5O_3S$): yield: 55%; white powder; mp: 159.1-161.9 °C; ^{1}H NMR (500 MHz, Chloroform-*d*) δ_H 7.85 – 7.78 (m, 2H), 7.55 – 7.48 (m, 2H), 7.30 (dd, *J* = 8.8, 7.2 Hz, 2H), 6.96 (s, 1H), 6.96 – 6.89 (m, 2H), 4.09 – 4.01 (m, 1H), 3.84 – 3.72 (m, 2H), 3.68 – 3.56 (m, 3H), 3.21 – 3.14 (m, 5H), 3.12 (dd, *J* = 15.2, 12.2 Hz, 1H), 2.68 – 2.60 (m, 2H), 2.45 – 2.28 (m, 4H), 2.08 – 1.98 (m, 2H), 1.86 – 1.78 (m, 1H), 1.78 – 1.46 (m, 8H), 1.41 – 1.23 (m, 2H); ^{13}C NMR (126 MHz, Chloroform-*d*) δ_C 170.92, 150.98, 139.26, 129.61, 129.24, 128.58, 120.50, 116.67, 116.31, 64.57, 57.22, 51.60, 50.94, 49.77, 49.43, 45.50, 45.48, 43.15, 42.60, 41.51, 32.40, 26.54, 25.18, 24.52, 24.11, 23.88, 23.00. HRMS (ESI): $C_{32}H_{39}ClN_5O_3S$ (608.2468) $[M-H]^-$=608.2469.

Data for **6i** ($C_{26}H_{35}BrN_4O_3S$): yield: 58%; light brown powder; mp: 162.1-164.6 °C; ^{1}H NMR (500 MHz, Chloroform-*d*) δ_H 7.77 – 7.71 (m, 2H), 7.70 – 7.63 (m, 2H), 4.07 – 3.99 (m, 1H), 3.64 – 3.56 (m, 1H), 3.46 (t, *J* = 6.9 Hz, 3H), 3.42 – 3.35 (m, 1H), 3.38 – 3.31 (m, 1H), 3.10 (dd, *J* = 15.2, 12.1 Hz, 1H), 2.68 – 2.60 (m, 2H), 2.43 – 2.29 (m, 2H), 2.32 – 2.16 (m, 2H), 2.05 – 1.91 (m, 4H), 1.90 – 1.81 (m, 4H), 1.77 – 1.40 (m, 7H), 1.43 – 1.29 (m, 1H), 1.32 – 1.24 (m, 1H; ^{13}C NMR (126 MHz, Chloroform-*d*) δ_C 170.88, 139.80, 132.50, 128.74, 127.65, 116.32, 64.62, 57.34, 51.61, 50.95, 46.59, 45.63, 45.61, 43.14, 42.76, 33.88, 26.54, 26.12, 25.16, 24.53, 24.41, 24.12, 23.90, 22.77. HRMS (ESI): $C_{26}H_{36}BrN_4O_3S$ (563.1686) $[M+H]^+$=563.1677.

Data for **6j** ($C_{26}H_{35}BrN_4O_4S$): yield: 74%; white powder; mp: 158.4-160.5 °C; ^{1}H NMR (500 MHz, Chloroform-*d*) δ_H 7.76 – 7.70 (m, 2H), 7.70 – 7.64 (m, 2H), 4.07 – 4.00 (m, 1H), 3.72 – 3.64 (m, 5H), 3.66 – 3.50 (m, 3H), 3.50 – 3.38 (m, 2H), 3.10 (dd, *J* = 15.2, 12.2 Hz, 1H), 2.68 – 2.60 (m, 2H), 2.43 – 2.21 (m, 4H), 2.08 – 1.94 (m, 1H), 1.85 – 1.77 (m, 1H), 1.76 – 1.68 (m, 2H), 1.71 – 1.63 (m, 2H), 1.66 – 1.44 (m, 5H),

1.40 – 1.23 (m, 2H); ^{13}C NMR (126 MHz, Chloroform-*d*) δ_C 171.04, 139.80, 132.57, 128.66, 127.74, 116.29, 66.93, 66.67, 64.55, 57.23, 51.59, 50.94, 45.96, 45.51, 43.14, 42.61, 41.91, 32.19, 26.52, 25.17, 24.51, 24.11, 23.87, 22.92. HRMS (ESI): $C_{26}H_{36}BrN_4O_4S$ (579.1635) $[M+H]^+$=579.1626.

Data for **6k** ($C_{26}H_{36}BrN_5O_3S$): yield: 39%; white powder; mp: 155.1-156.9 °C; ^{1}H NMR (500 MHz, Chloroform-*d*) δ_H 7.76 – 7.71 (m, 2H), 7.71 – 7.65 (m, 2H), 4.08 – 4.00 (m, 1H), 3.76 – 3.65 (m, 5H), 3.63 (t, *J* = 4.7 Hz, 2H), 3.63 – 3.49 (m, 3H), 3.11 (dd, *J* = 15.3, 12.2 Hz, 1H), 2.68 – 2.60 (m, 2H), 2.45 – 2.27 (m, 4H), 2.08 – 1.97 (m, 1H), 1.82 – 1.75 (m, 1H), 1.77 – 1.65 (m, 6H), 1.68 – 1.56 (m, 1H), 1.58 – 1.42 (m, 3H), 1.40 – 1.25 (m, 2H); ^{13}C NMR (126 MHz, Chloroform-*d*) δ_C 171.18, 139.80, 132.62, 128.61, 127.78, 116.29, 64.49, 57.18, 51.57, 50.93, 45.44, 44.82, 43.34, 43.14, 42.52, 41.44, 40.88, 32.30, 26.48, 25.17, 24.49, 24.10, 23.85, 22.82. HRMS (ESI): $C_{26}H_{35}BrN_5O_3S$ (576.1649) $[M-H]^-$=576.1651.

Data for **6l** ($C_{27}H_{38}BrN_5O_3S$): yield: 46%; white powder; mp: 134.1-136.7 °C; ^{1}H NMR (500 MHz, Chloroform-*d*) δ_H 7.77 – 7.70 (m, 2H), 7.73 – 7.63 (m, 2H), 4.07 – 3.99 (m, 1H), 3.67 (s, 2H), 3.66 (s, 1H), 3.66 – 3.56 (m, 1H), 3.56 – 3.44 (m, 2H), 3.16 – 3.05 (m, 1H), 2.68 – 2.59 (m, 2H), 2.50 – 2.22 (m, 9H), 2.35 (s, 3H), 2.07 – 1.92 (m, 1H), 1.85 – 1.77 (m, 1H), 1.77 – 1.66 (m, 1H), 1.69 – 1.53 (m, 5H), 1.55 – 1.50 (m, 1H), 1.49 (d, *J* = 4.1 Hz, 1H), 1.47 – 1.30 (m, 1H), 1.32 – 1.24 (m, 1H); ^{13}C NMR (126 MHz, Chloroform-*d*) δ_C 170.81, 139.78, 132.57, 128.67, 127.74, 116.28, 64.55, 57.21, 54.99, 54.60, 51.59, 50.93, 45.81, 45.52, 45.17, 43.14, 42.61, 41.23, 32.29, 26.52, 25.16, 24.51, 24.10, 23.87, 22.99. HRMS (ESI): $C_{27}H_{39}BrN_5O_3S$ (592.1951) $[M+H]^+$=592.1942.

Data for **6m** ($C_{26}H_{33}BrN_4O_4S_2$): yield: 74%; white powder; mp: 130.3-132.7 °C; ^{1}H NMR (500 MHz, Chloroform-*d*) δ_H 7.76 – 7.71 (m, 2H), 7.71 – 7.66 (m, 2H), 6.05 (dd, *J* = 16.8, 6.4 Hz, 1H), 4.62 – 4.49 (m, 1H), 4.07 – 3.98 (m, 1H), 3.66 – 3.57 (m, 1H), 3.42 – 3.32 (m, 1H), 3.31 – 3.23 (m, 1H), 3.16 – 3.06 (m, 1H), 2.96 – 2.85 (m, 1H), 2.68 – 2.60 (m, 2H), 2.43 – 2.14 (m, 5H), 2.13 – 1.90 (m, 3H), 1.84 – 1.62 (m, 3H), 1.66 – 1.43 (m, 6H), 1.46 – 1.23 (m, 2H); ^{13}C NMR (126 MHz, Chloroform-*d*) δ_C 205.40, 172.96, 139.75, 132.64, 128.61, 127.81, 116.22, 64.49, 59.36, 56.90, 51.57,

50.93, 45.42, 43.16, 42.54, 35.40, 31.85, 27.55, 25.95, 25.12, 24.51, 24.09, 23.31, 23.09. HRMS (ESI): $C_{26}H_{34}BrN_4O_4S_2$ (609.1199) $[M+H]^+$=609.1190.

Data for **6n** ($C_{25}H_{30}BrN_5O_3S_2$): yield: 73%; white powder; mp: 161.3-163.9 ℃; ^{1}H NMR (500 MHz, Chloroform-*d*) δ_H 12.06 (s, 1H), 7.75 – 7.69 (m, 2H), 7.67 – 7.63 (m, 2H), 7.46 (d, *J* = 3.6 Hz, 1H), 7.02 (d, *J* = 3.6 Hz, 1H), 4.09 – 4.01 (m, 1H), 3.70 – 3.60 (m, 1H), 3.15 – 3.05 (m, 1H), 2.68 – 2.60 (m, 2H), 2.56 (t, *J* = 7.2 Hz, 2H), 2.44 – 2.24 (m, 2H), 2.17 – 2.03 (m, 1H), 1.86 – 1.69 (m, 2H), 1.72 – 1.41 (m, 8H), 1.41 – 1.21 (m, 3H); ^{13}C NMR (126 MHz, Chloroform-*d*) δ_C 170.49, 159.81, 139.75, 136.38, 132.62, 128.57, 127.82, 116.26, 113.53, 64.51, 56.97, 51.57, 50.94, 45.40, 43.16, 42.60, 35.13, 26.06, 25.15, 24.49, 24.10, 23.85, 22.71. HRMS (ESI): $C_{25}H_{31}BrN_5O_3S_2$ (592.1046) $[M+H]^+$=592.1040.

Data for **6o** ($C_{32}H_{46}BrN_5O_3S$): yield: 73%; white powder; mp: 214.7-215.9 ℃; ^{1}H NMR (500 MHz, Chloroform-*d*) δ_H 7.78 – 7.70 (m, 2H), 7.70 – 7.63 (m, 2H), 4.72 – 4.65 (m, 1H), 4.07 – 3.96 (m, 1H), 3.93 – 3.84 (m, 1H), 3.63 – 3.55 (m, 1H), 3.09 (dd, *J* = 15.2, 12.1 Hz, 2H), 3.03 – 2.89 (m, 1H), 2.67 – 2.60 (m, 1H), 2.63 – 2.57 (m, 7H), 2.57 – 2.47 (m, 1H), 2.42 – 2.22 (m, 4H), 1.99 (s, 1H), 1.99 – 1.92 (m, 1H), 1.95 – 1.85 (m, 1H), 1.84 – 1.77 (m, 1H), 1.76 – 1.69 (m, 1H), 1.69 – 1.62 (m, 7H), 1.62 – 1.49 (m, 3H), 1.51 – 1.39 (m, 5H), 1.39 – 1.23 (m, 2H); ^{13}C NMR (126 MHz, Chloroform-*d*) δ_C 170.61, 139.80, 132.56, 128.68, 127.71, 116.29, 64.56, 62.75, 57.23, 51.60, 50.94, 50.07, 45.56, 45.49, 45.10, 45.08, 43.16, 43.12, 42.63, 41.26, 32.42, 32.38, 28.42, 26.53, 25.76, 25.16, 24.52, 24.36, 24.11, 23.88, 23.16, 23.02. HRMS (ESI): $C_{32}H_{47}BrN_5O_3S$ (658.2432) $[M-H]^-$=658.2438.

Data for **6p** ($C_{32}H_{40}BrN_5O_3S$): yield: 98%; light yellow powder; mp: 164.4-66.2 ℃; ^{1}H NMR (500 MHz, Chloroform-*d*) δ_H 7.77 – 7.70 (m, 2H), 7.70 – 7.65 (m, 2H), 7.30 (dd, *J* = 8.8, 7.4 Hz, 2H), 6.96 (d, *J* = 7.9 Hz, 2H), 6.96 – 6.89 (m, 1H), 4.09 – 4.02 (m, 1H), 3.85 – 3.77 (m, 1H), 3.78 (d, *J* = 5.4 Hz, 1H), 3.68 – 3.56 (m, 3H), 3.21 – 3.14 (m, 5H), 3.12 (dd, *J* = 15.2, 12.1 Hz, 1H), 2.69 – 2.60 (m, 2H), 2.45 – 2.28 (m, 4H), 2.08 – 1.96 (m, 1H), 1.86 – 1.79 (m, 1H), 1.77 – 1.64 (m, 3H), 1.64 – 1.46 (m, 6H), 1.41 – 1.29 (m, 1H), 1.32 – 1.23 (m, 1H); ^{13}C NMR (126 MHz, Chloroform-*d*) δ_C 170.91, 150.96, 139.80, 132.58, 129.25, 128.68, 127.75, 120.51,

116.68, 116.30, 64.57, 57.22, 51.60, 50.95, 49.78, 49.44, 45.53, 43.16, 42.62, 41.51, 32.40, 26.55, 25.18, 24.51, 24.11, 23.88, 23.01. HRMS (ESI): $C_{32}H_{41}BrN_5O_3S$ (654.2108) $[M+H]^+$=654.2100.

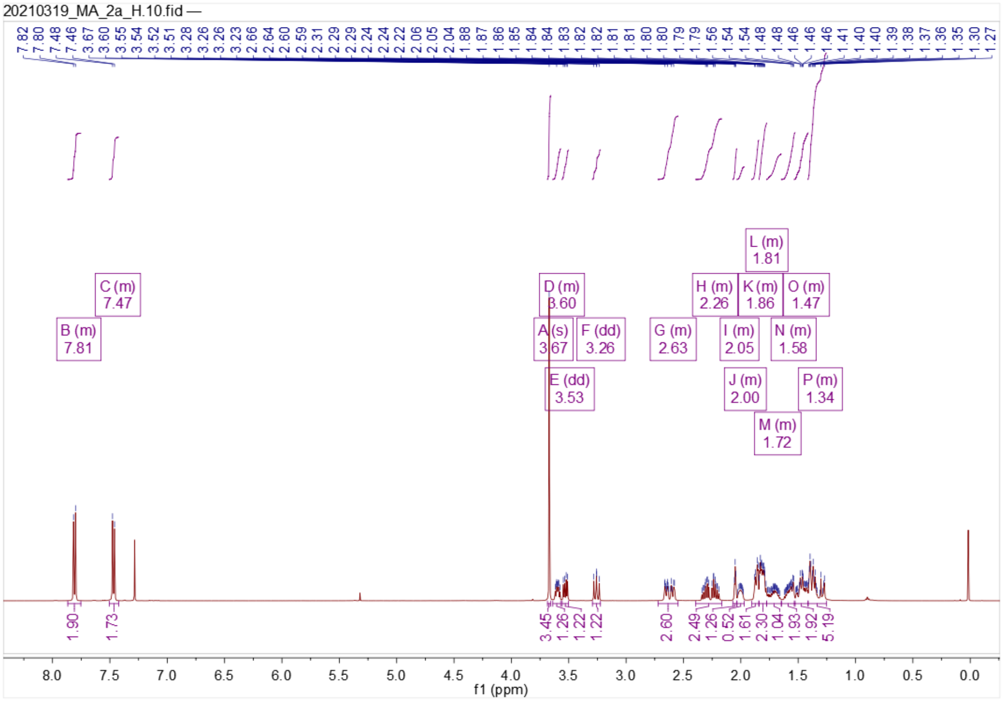

Figure S1. ^{1}H NMR spectrum (Chloroform-*d*, 500 MHz) of **2a**.

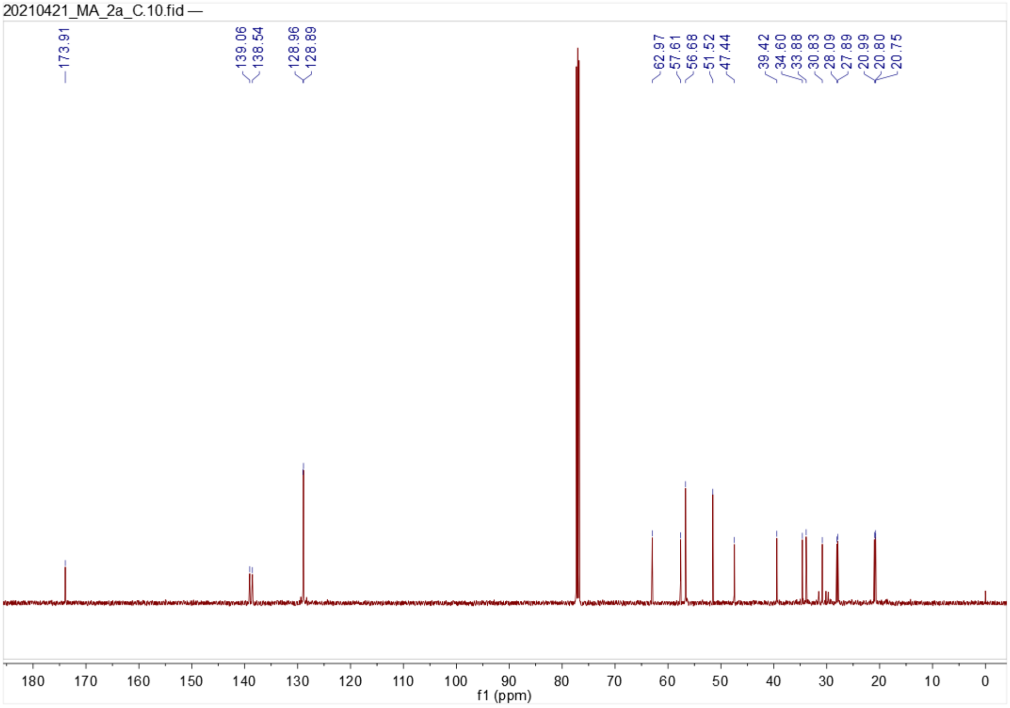

Figure S2. ^{13}C NMR spectrum (Chloroform-*d*, 126 MHz) of **2a**.

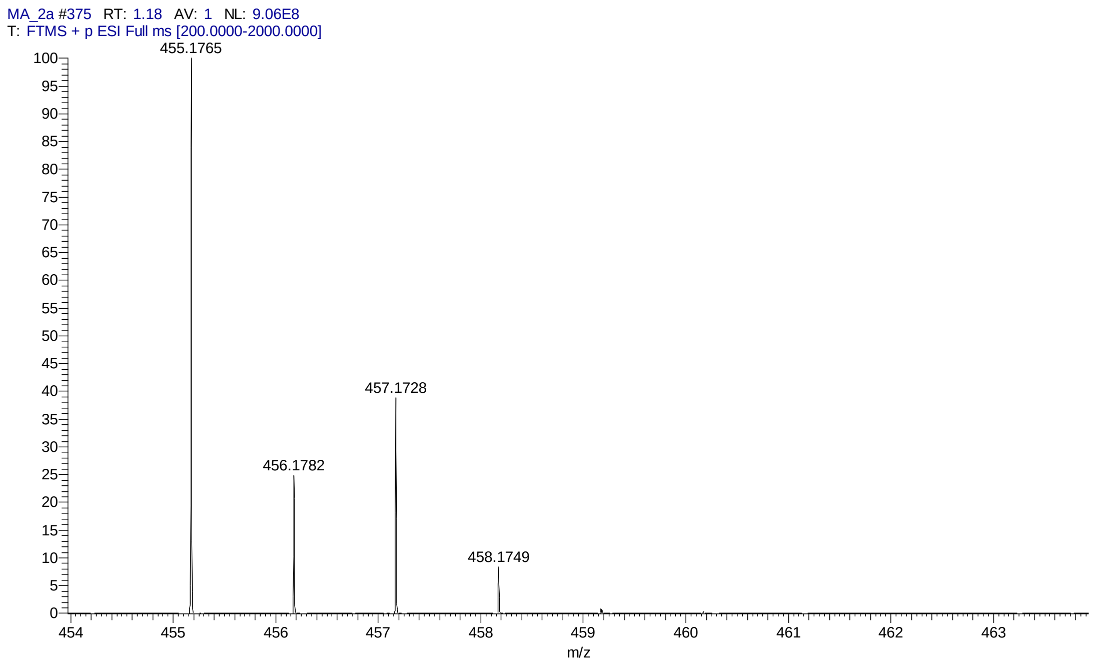

Figure S3. HRMS spectrum of **2a**.

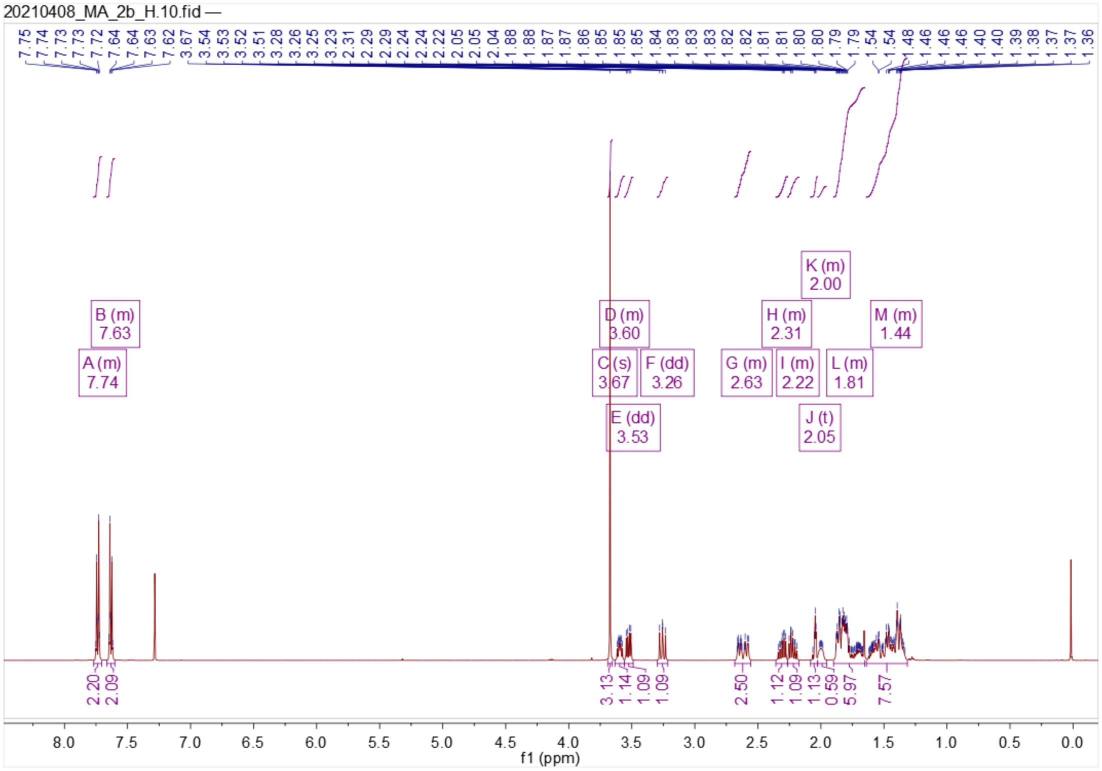

Figure S4. ^{1}H NMR spectrum (Chloroform-*d*, 500 MHz) of **2b**.

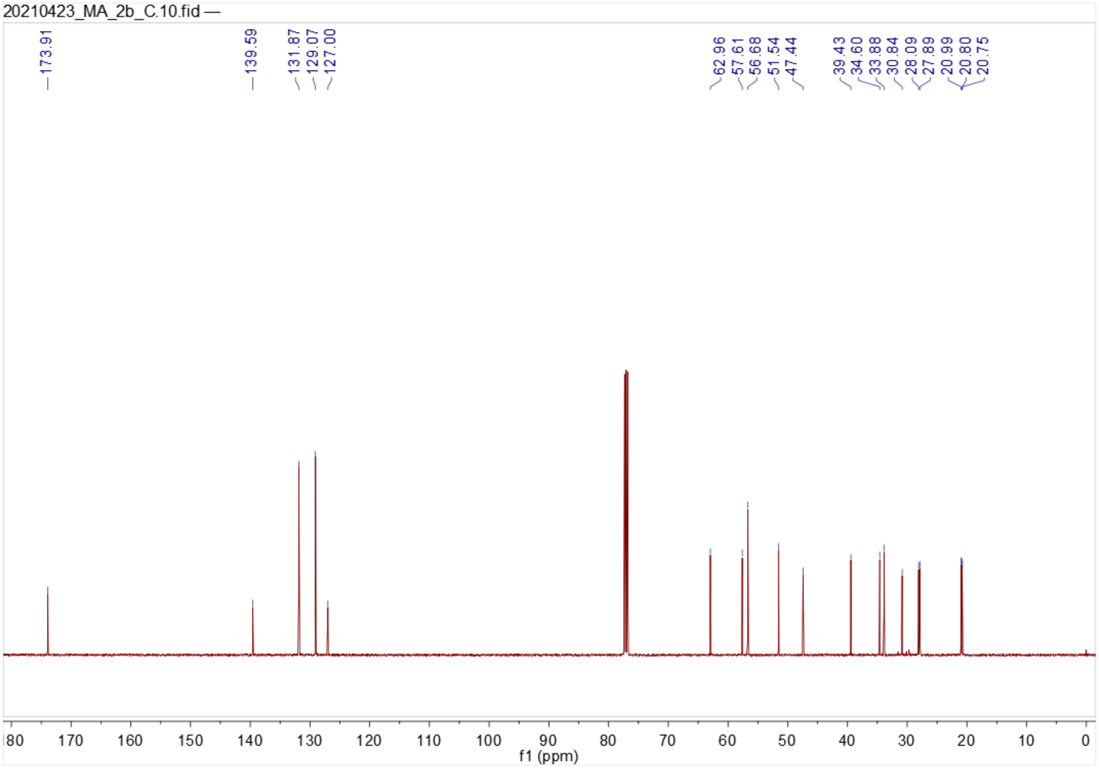

Figure S5. ^{13}C NMR spectrum (Chloroform-*d*, 126 MHz) of **2b**.

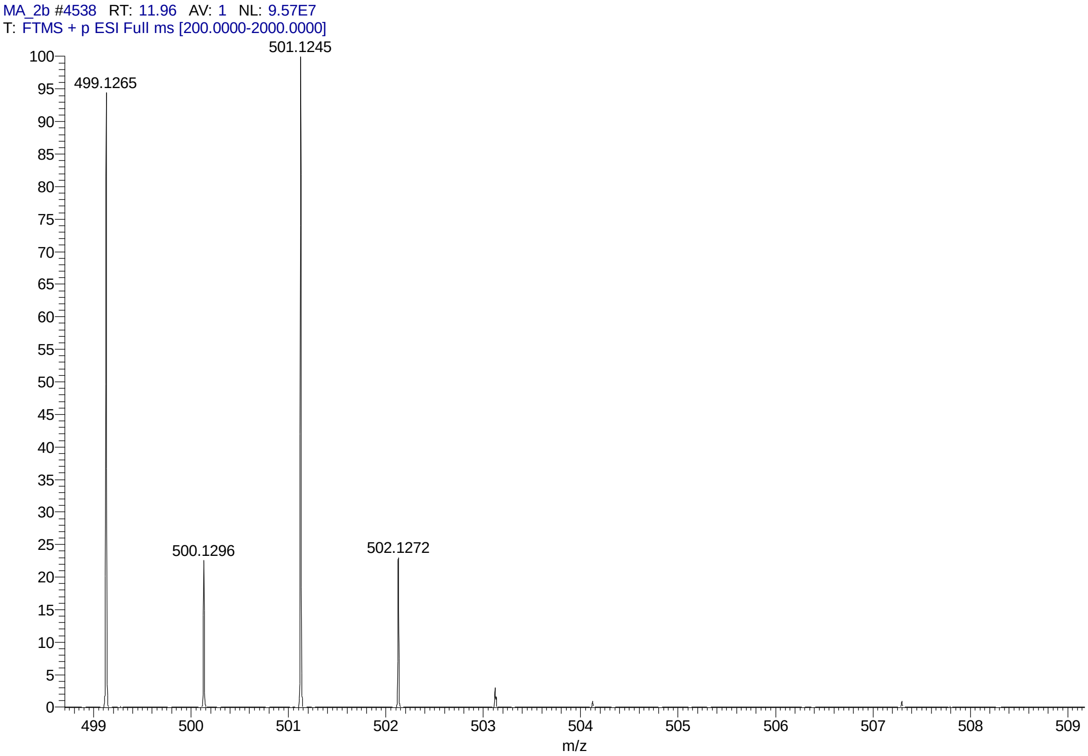

Figure S6. HRMS spectrum of **2b**.

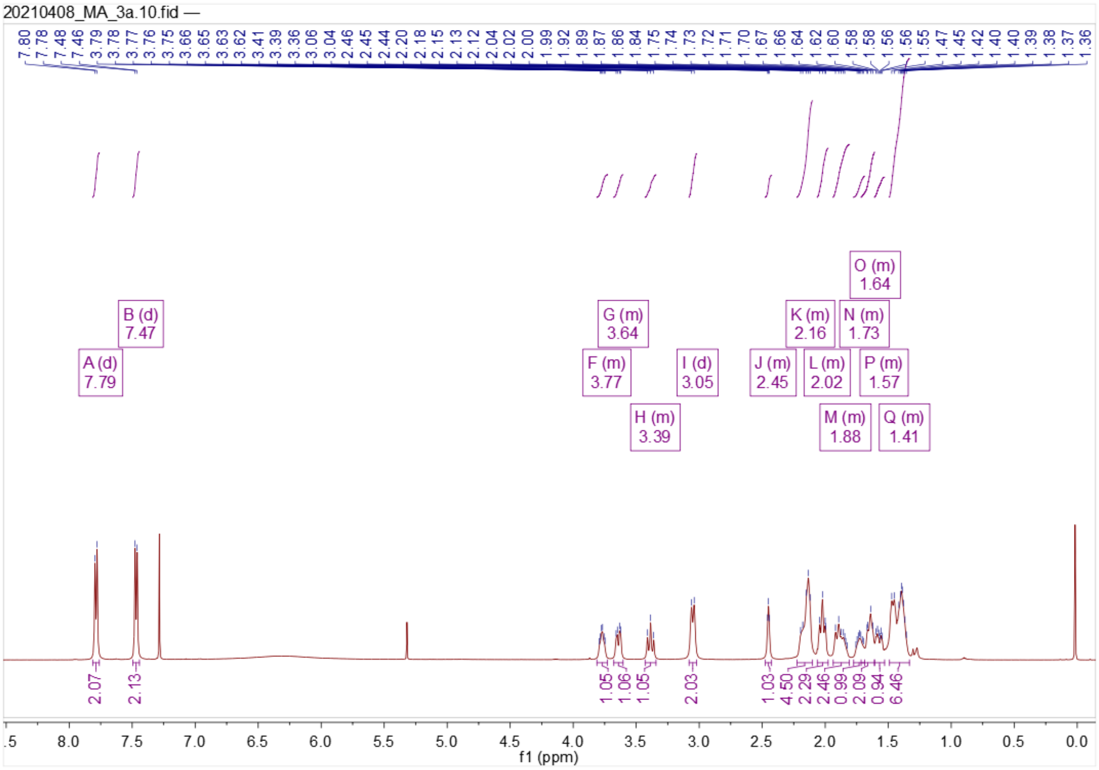

Figure S7. ^{1}H NMR spectrum (Chloroform-*d*, 500 MHz) of **3a**.

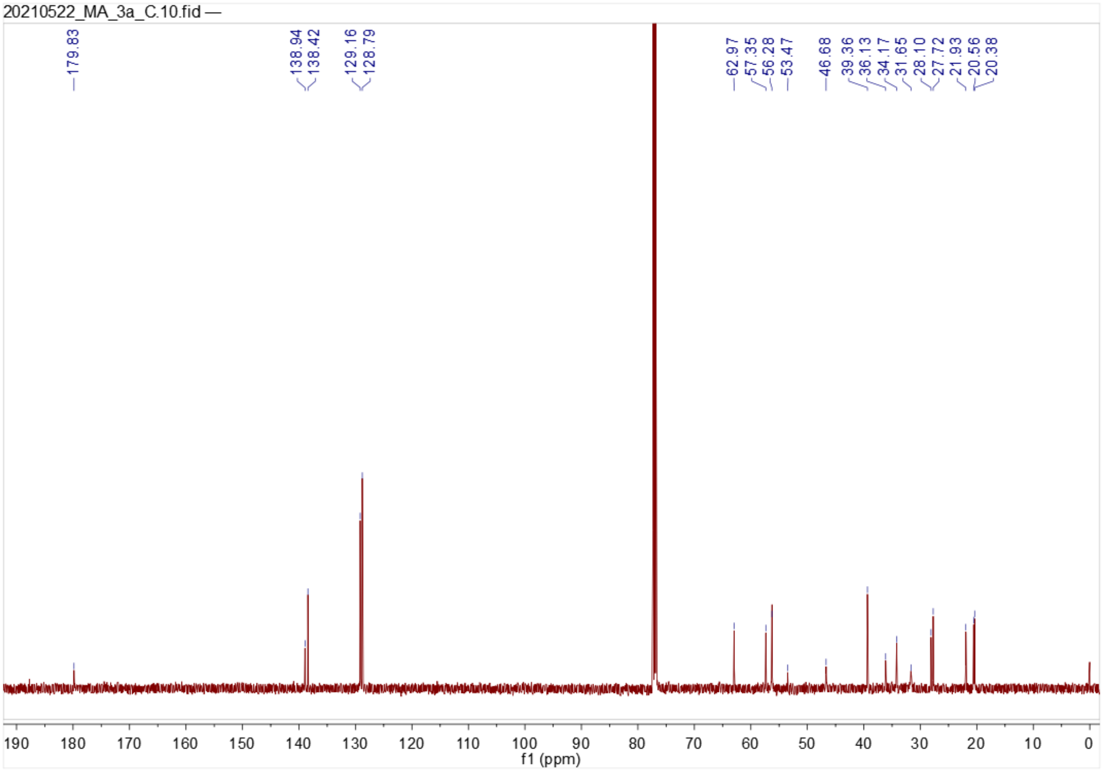

Figure S8. ^{13}C NMR spectrum (Chloroform-*d*, 126 MHz) of **3a**.

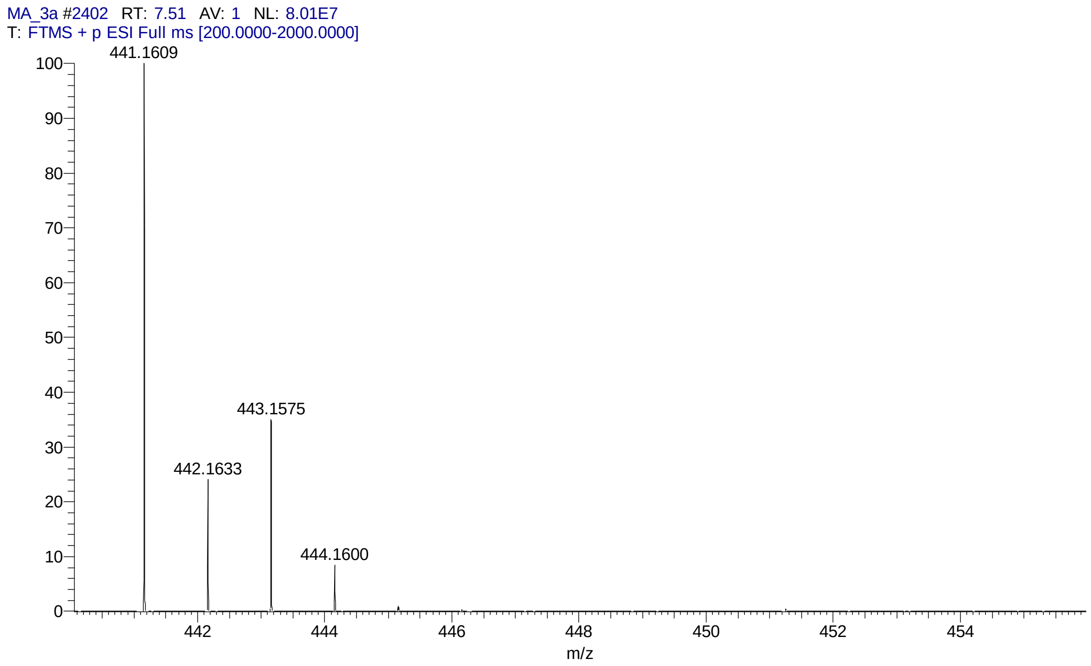

Figure S9. HRMS spectrum of **3a**.

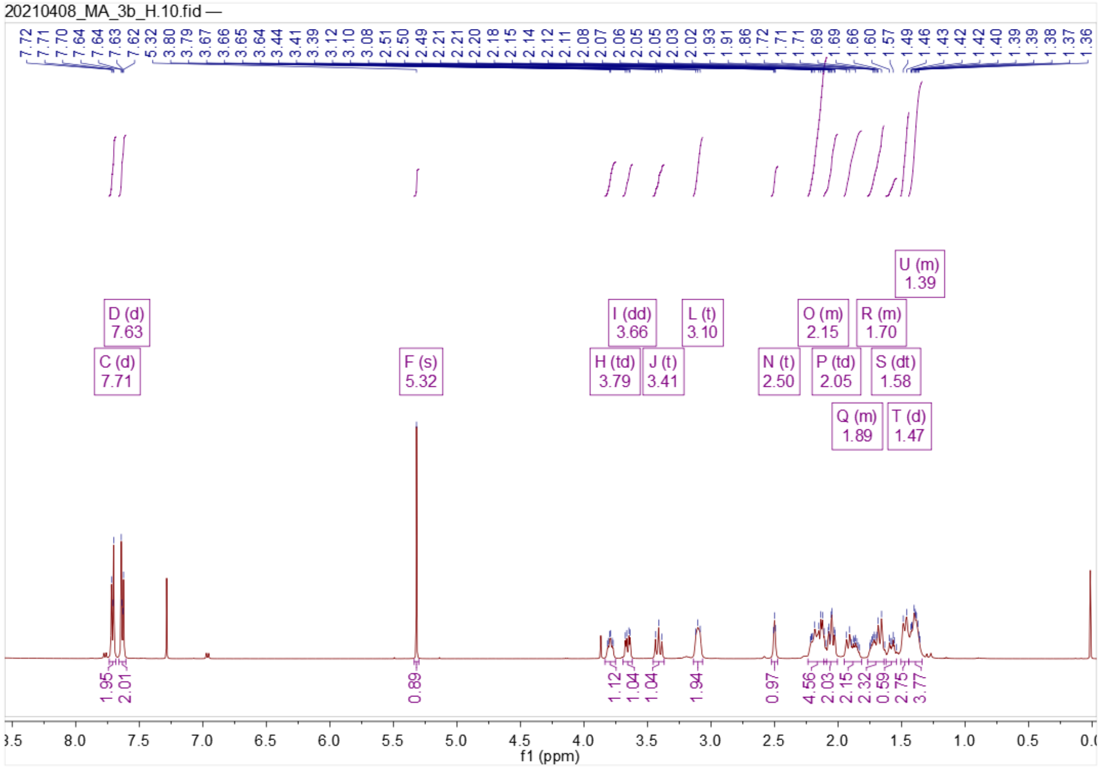

Figure S10. ^{1}H NMR spectrum (Chloroform-*d*, 500 MHz) of **3b**.

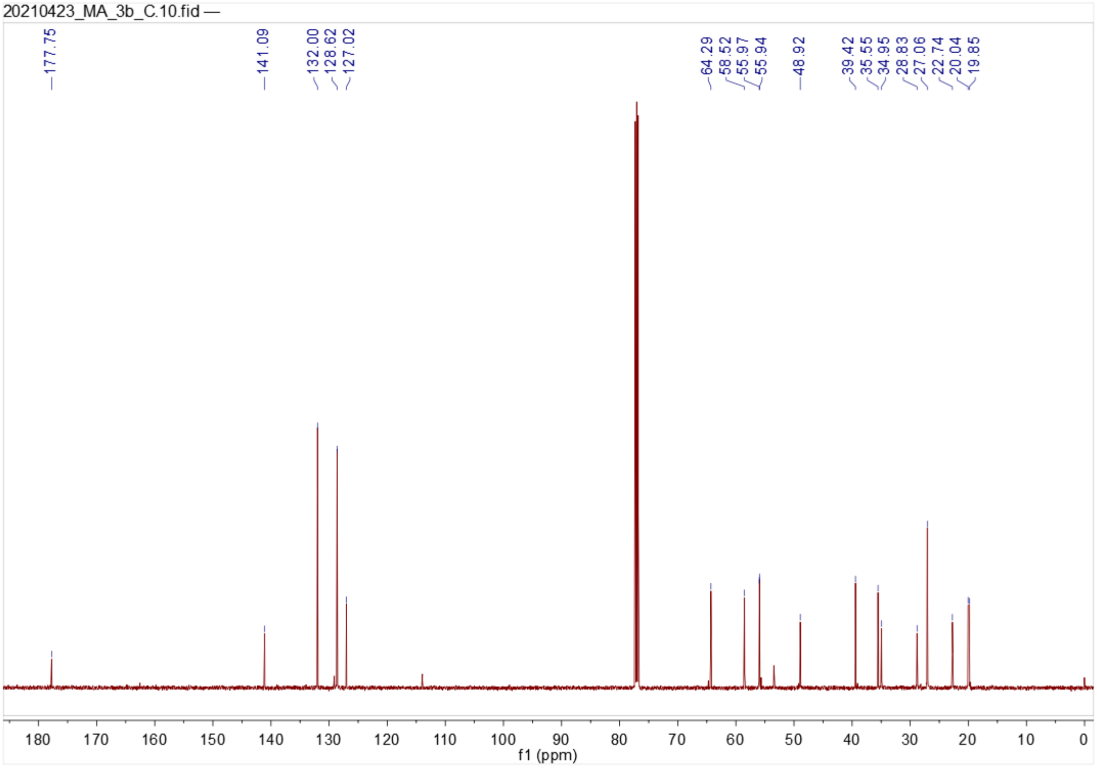

Figure S11. ^{13}C NMR spectrum (Chloroform-*d*, 126 MHz) of **3b**.

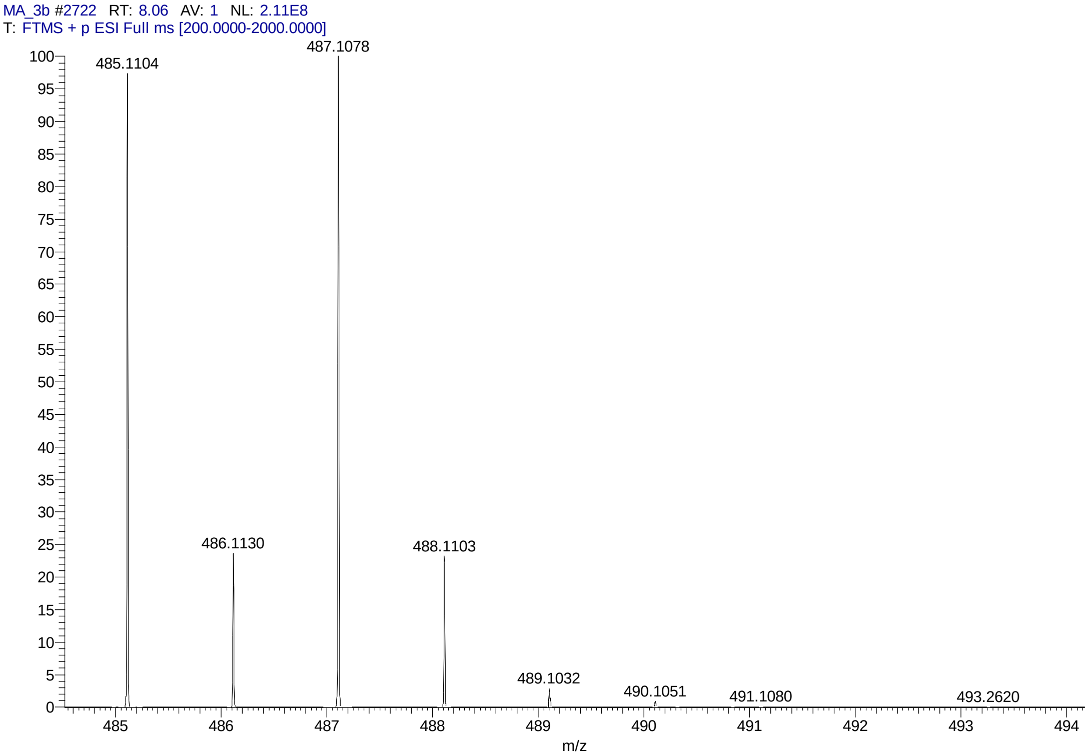

Figure S12. HRMS spectrum of **3b**.

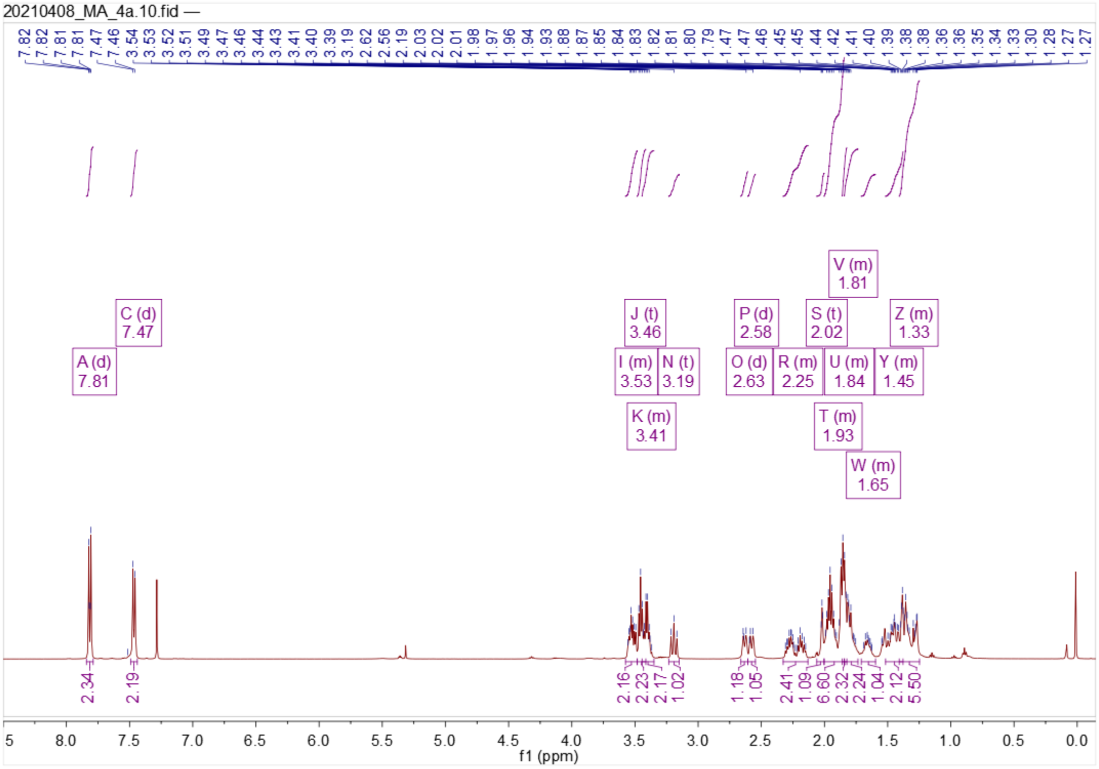

Figure S13. ^{1}H NMR spectrum (Chloroform-*d*, 500 MHz) of **4a**.

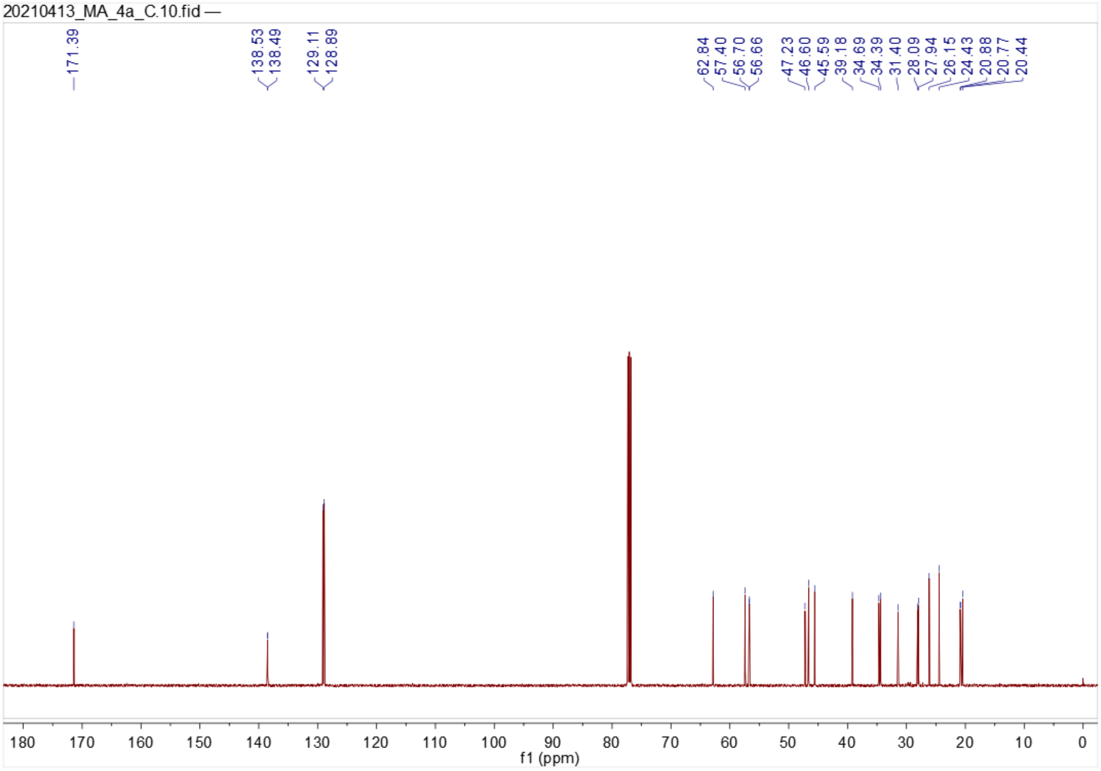

Figure S14. ^{13}C NMR spectrum (Chloroform-*d*, 126 MHz) of **4a**.

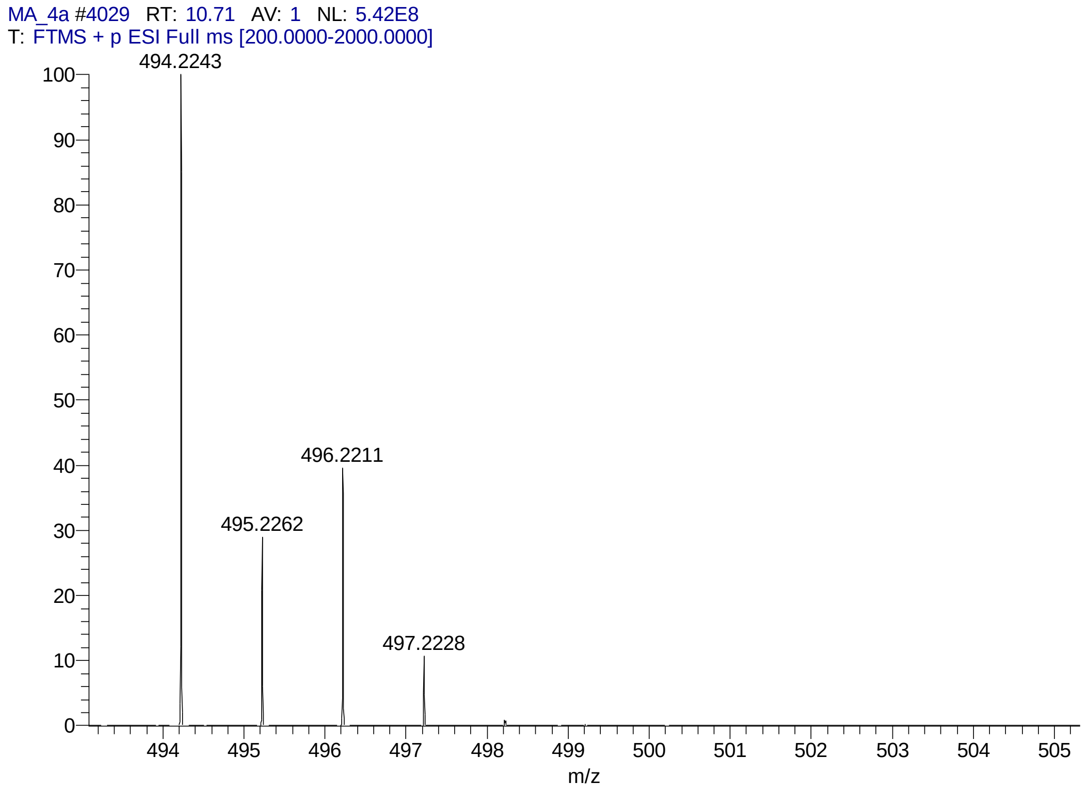

Figure S15. HRMS spectrum of **4a**.

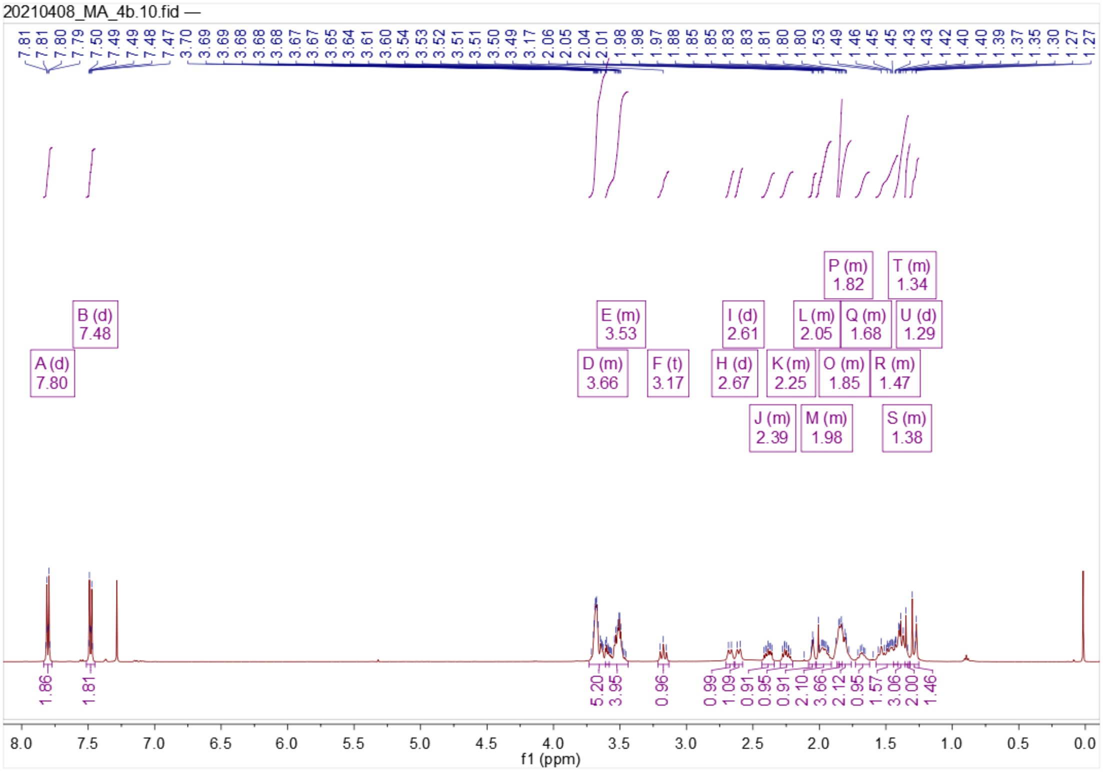

Figure S16. ^{1}H NMR spectrum (Chloroform-*d*, 500 MHz) of **4b**.

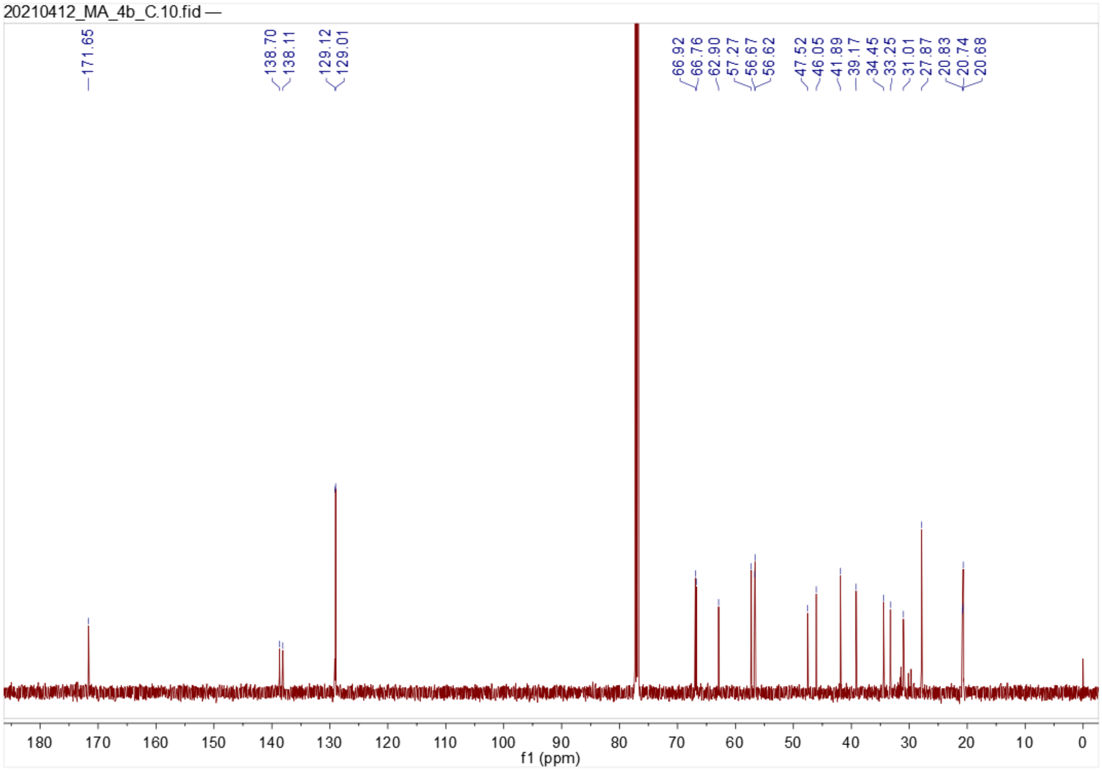

Figure S17. ^{13}C NMR spectrum (Chloroform-*d*, 126 MHz) of **4b**.

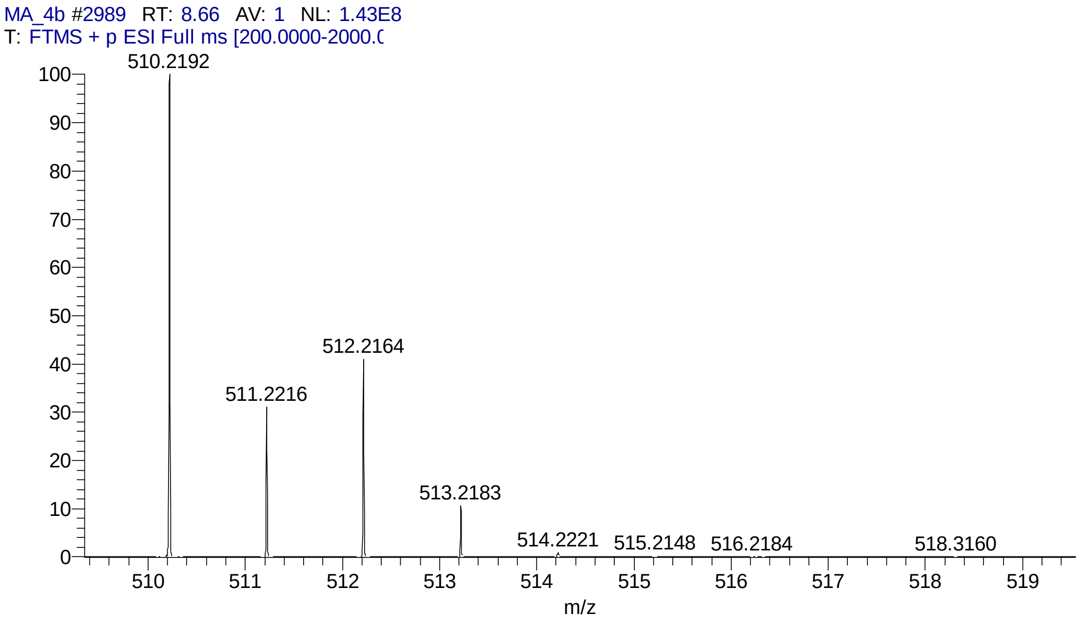

Figure S18. HRMS spectrum of **4b**.

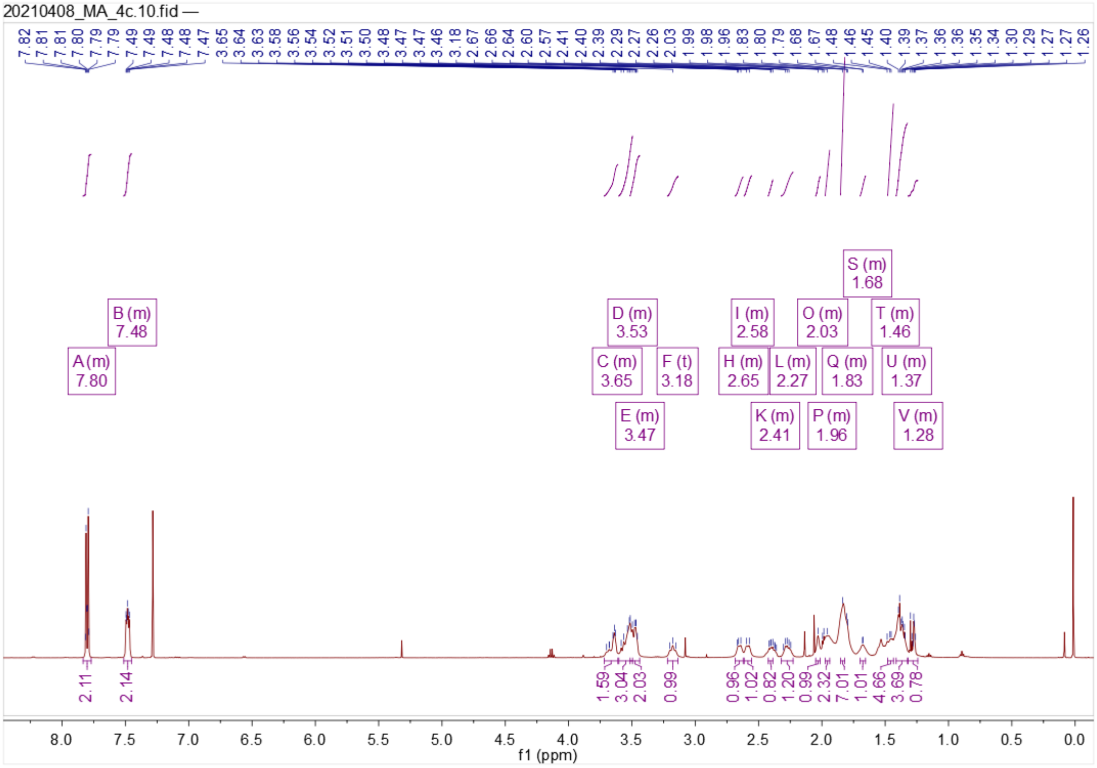

Figure S19. ^{1}H NMR spectrum (Chloroform-*d*, 500 MHz) of **4c**.

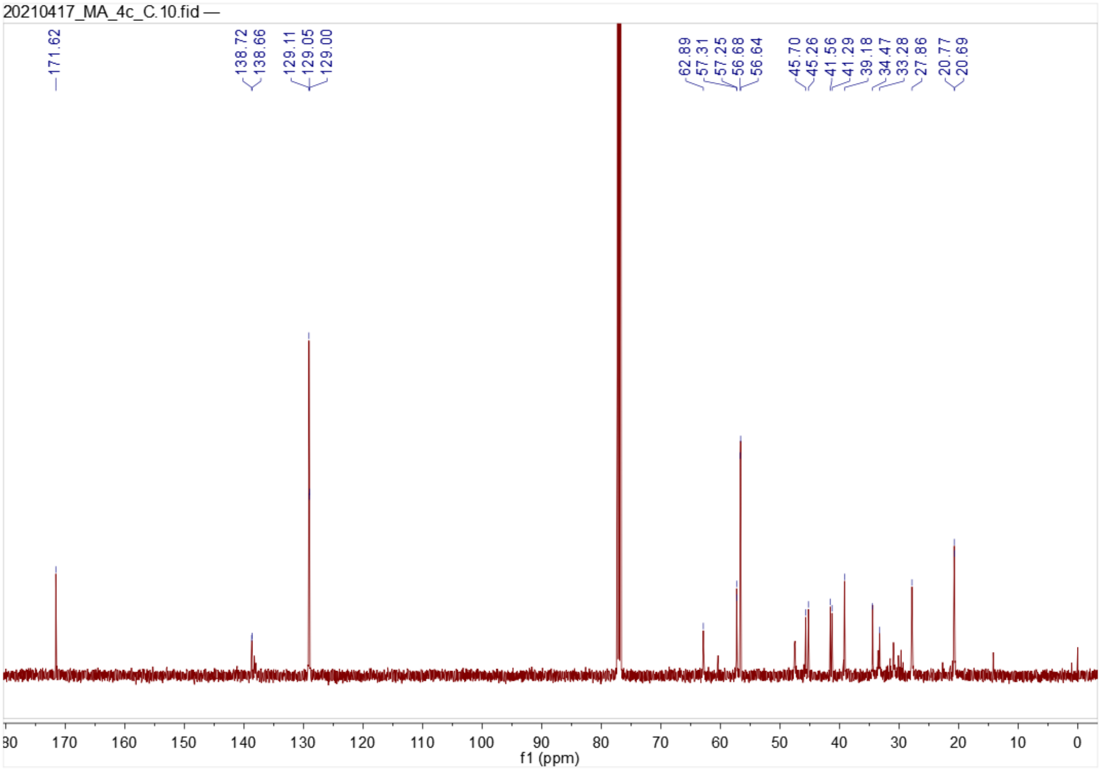

Figure S20. ^{13}C NMR spectrum (Chloroform-*d*, 126 MHz) of **4c**.

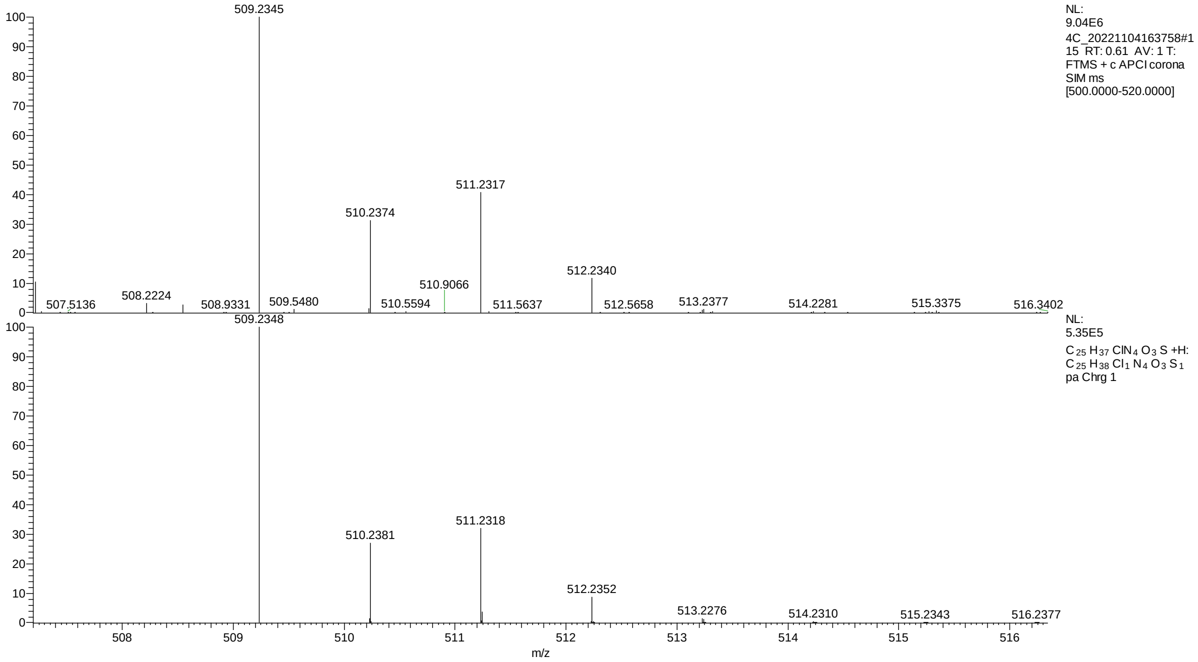

Figure S21. HRMS spectrum of **4c**.

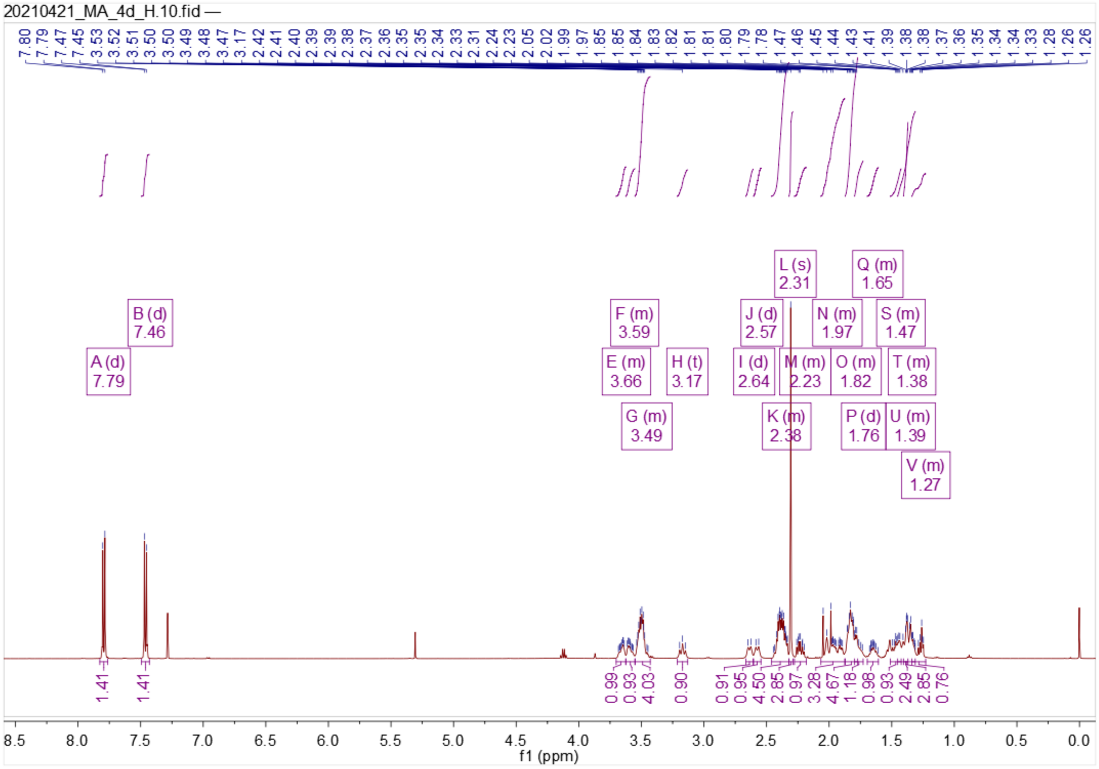

Figure S22. ^{1}H NMR spectrum (Chloroform-*d*, 500 MHz) of **4d**.

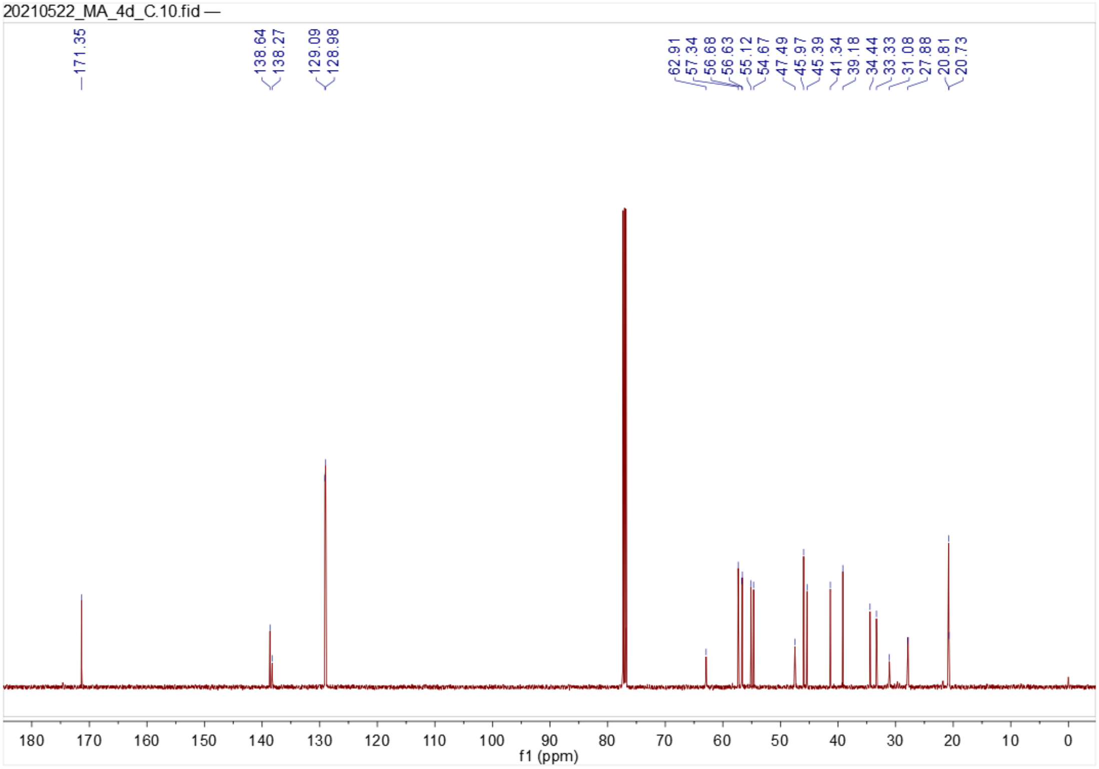

Figure S23. ^{13}C NMR spectrum (Chloroform-*d*, 126 MHz) of **4d**.

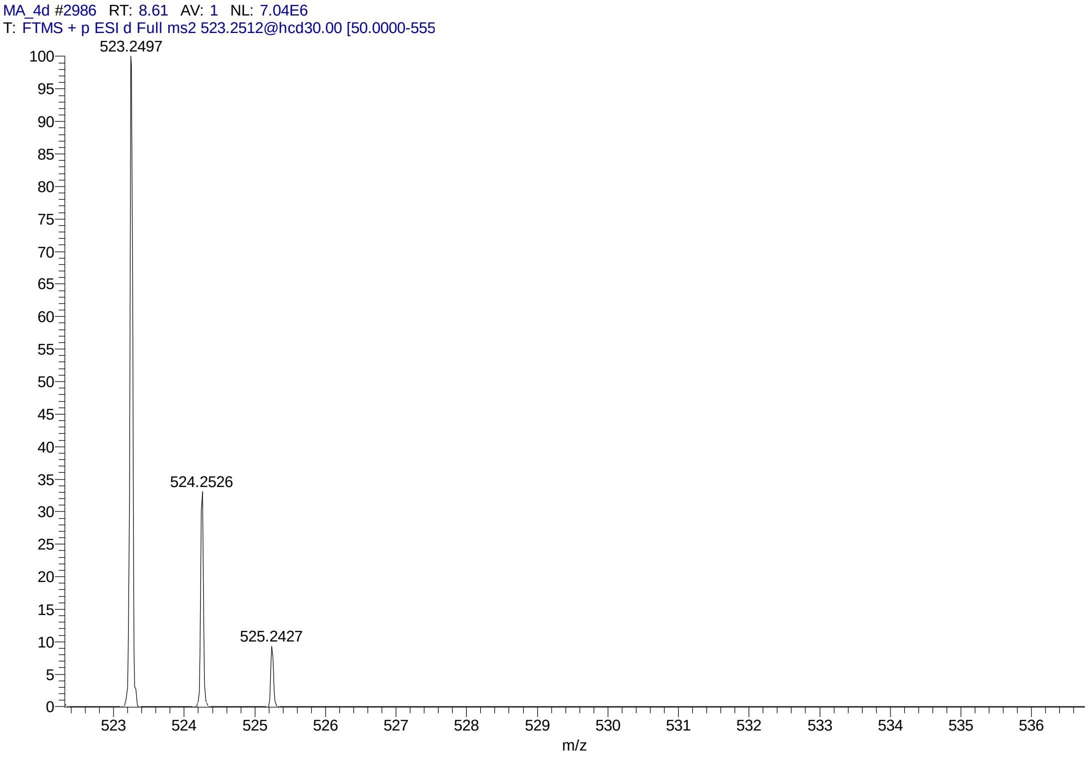

Figure S24. HRMS spectrum of **4d**.

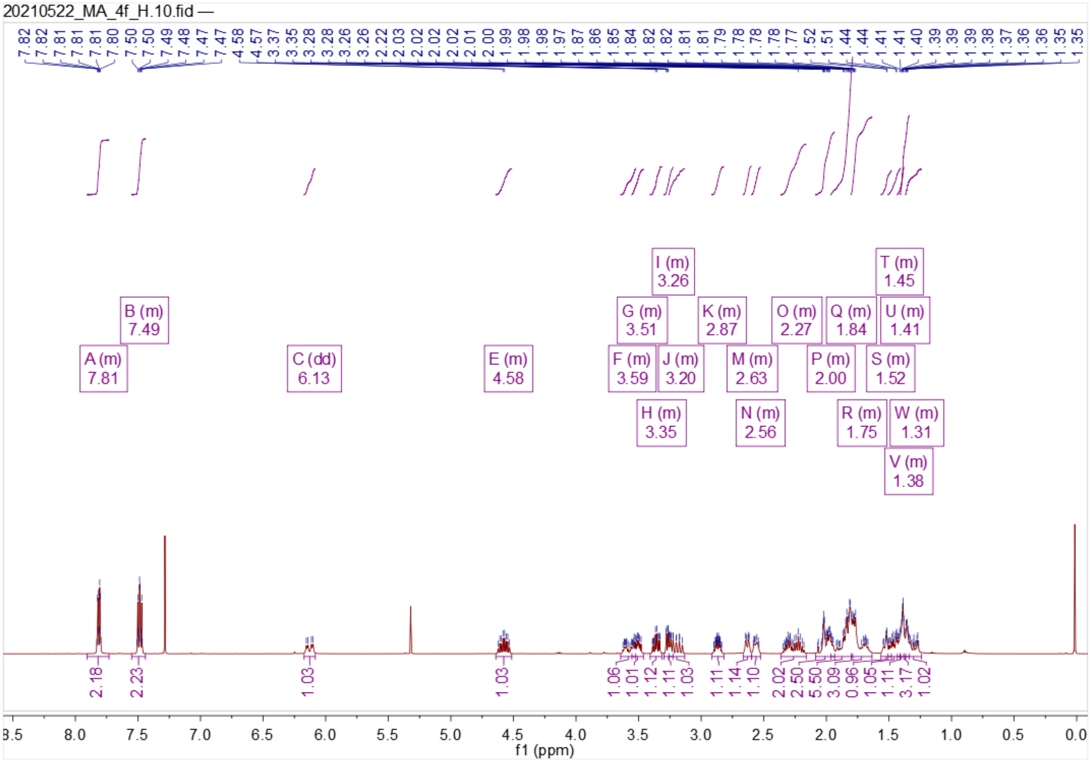

Figure S25. ^{1}H NMR spectrum (Chloroform-*d*, 500 MHz) of **4e**.

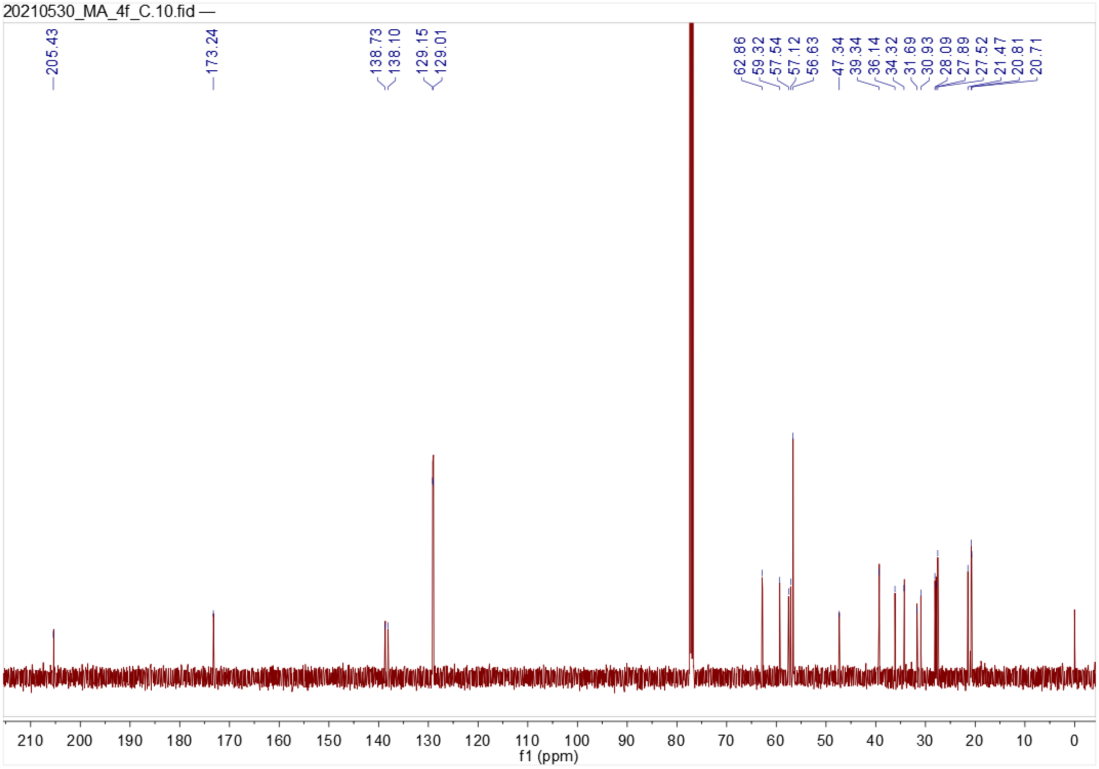

Figure S26. ^{13}C NMR spectrum (Chloroform-*d*, 126 MHz) of **4e**.

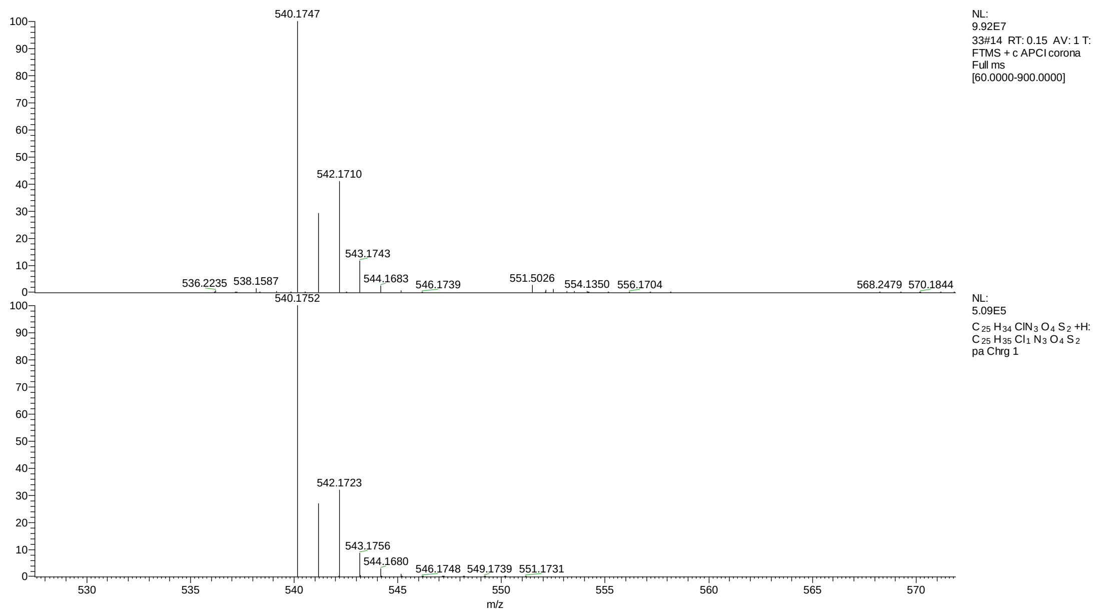

Figure S27. HRMS spectrum of **4e**.

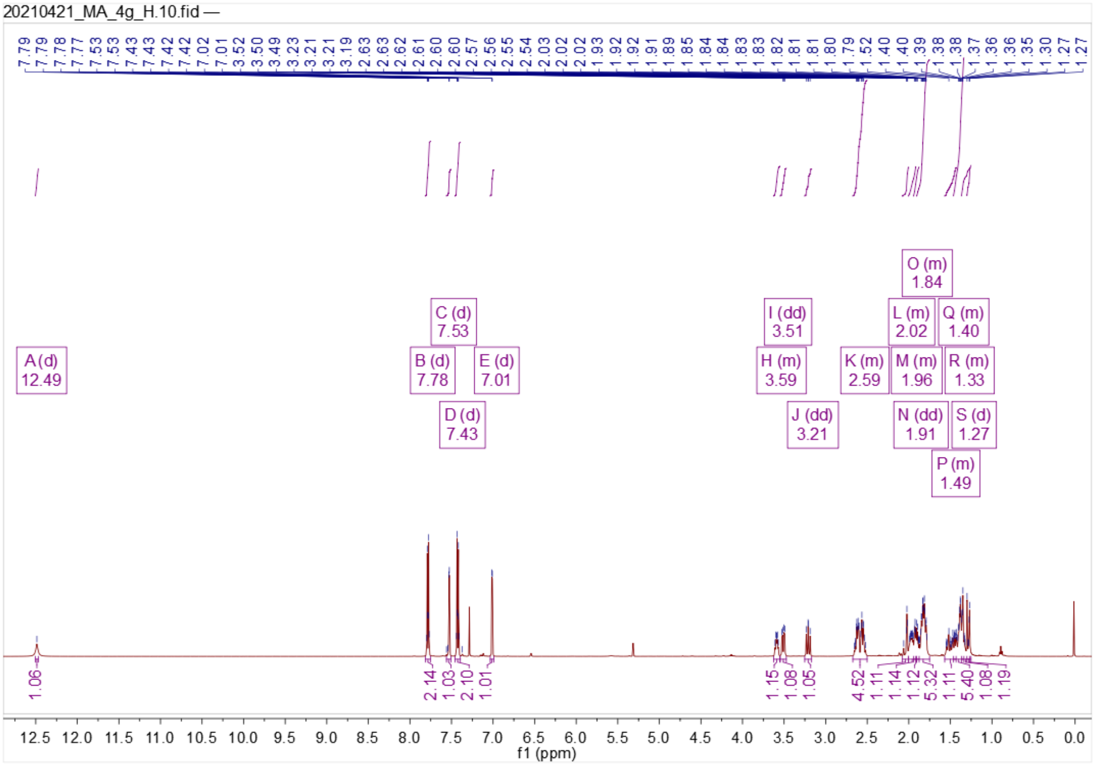

Figure S28. ^{1}H NMR spectrum (Chloroform-*d*, 500 MHz) of **4f**.

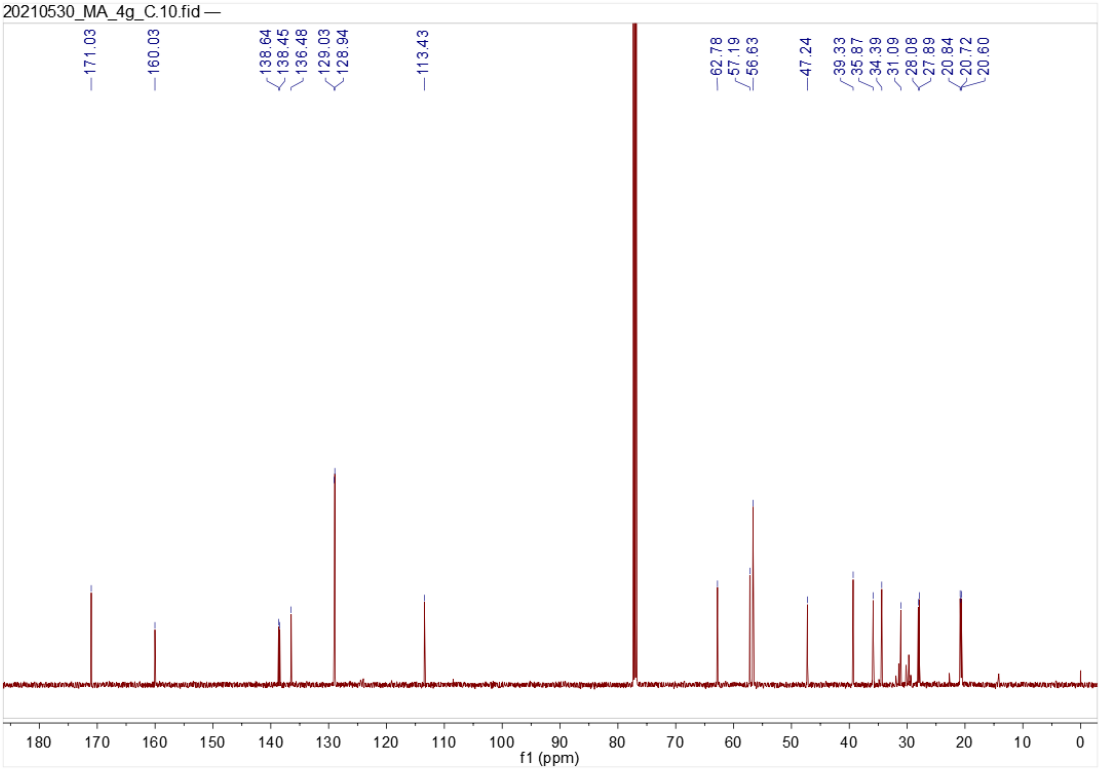

Figure S29. ^{13}C NMR spectrum (Chloroform-*d*, 126 MHz) of **4f**.

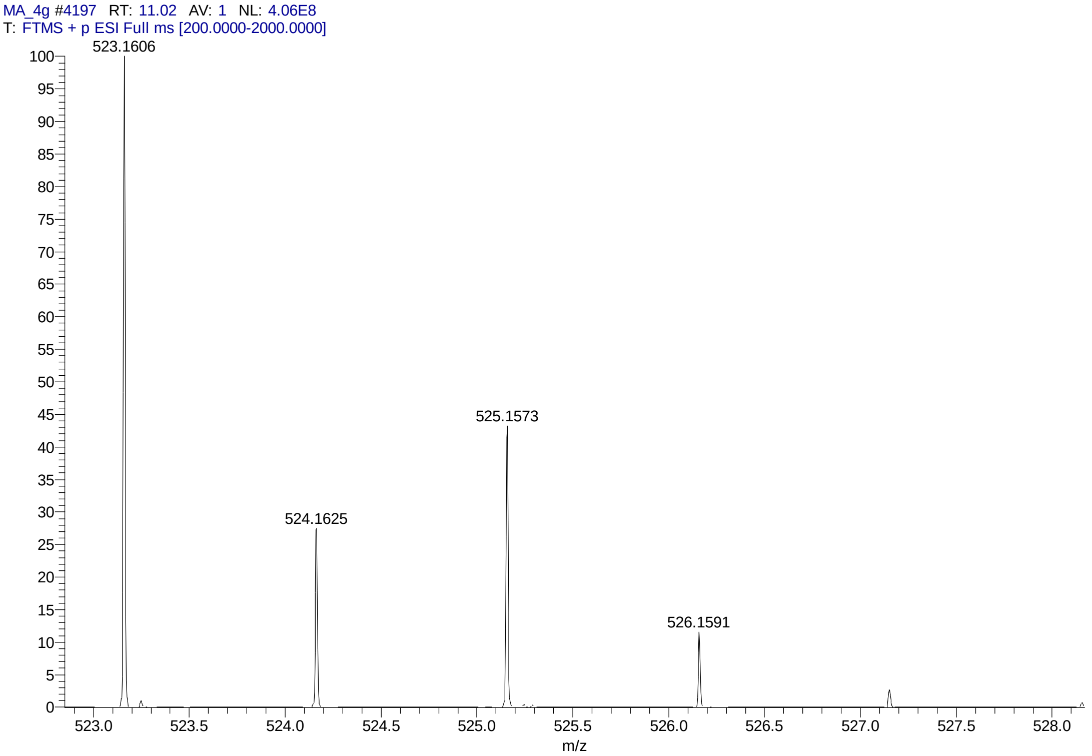

Figure S30. HRMS spectrum of **4f**.

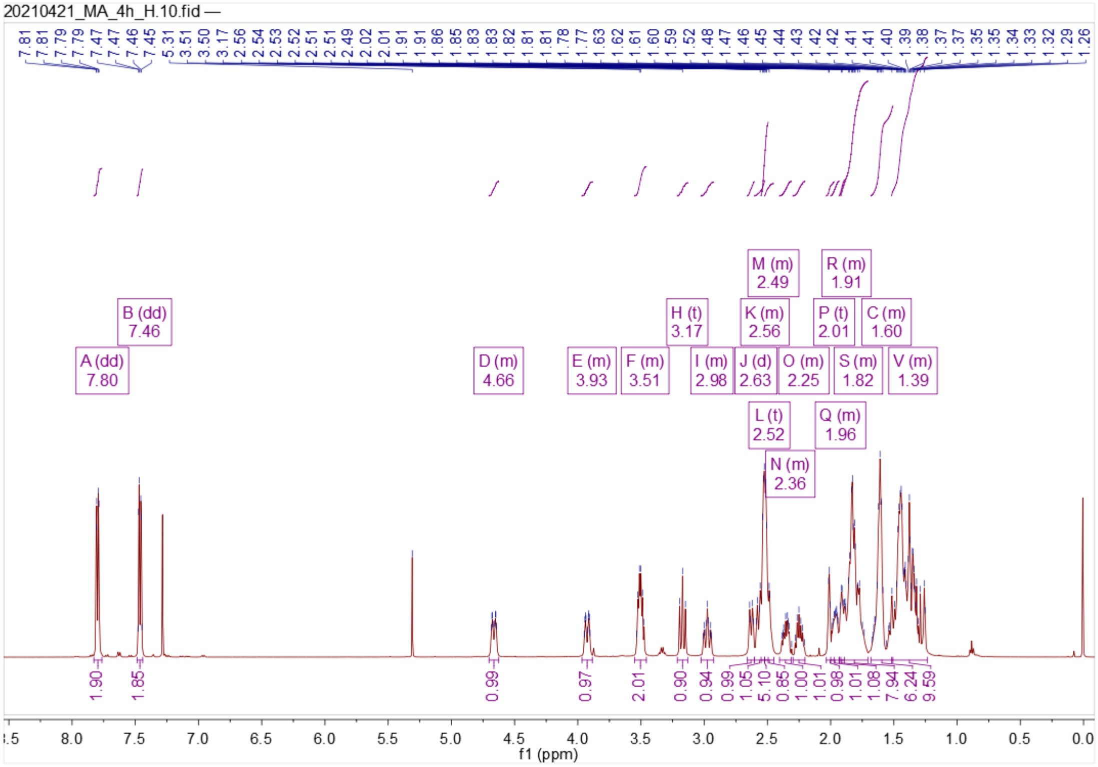

Figure S31. ^{1}H NMR spectrum (Chloroform-*d*, 500 MHz) of **4g**.

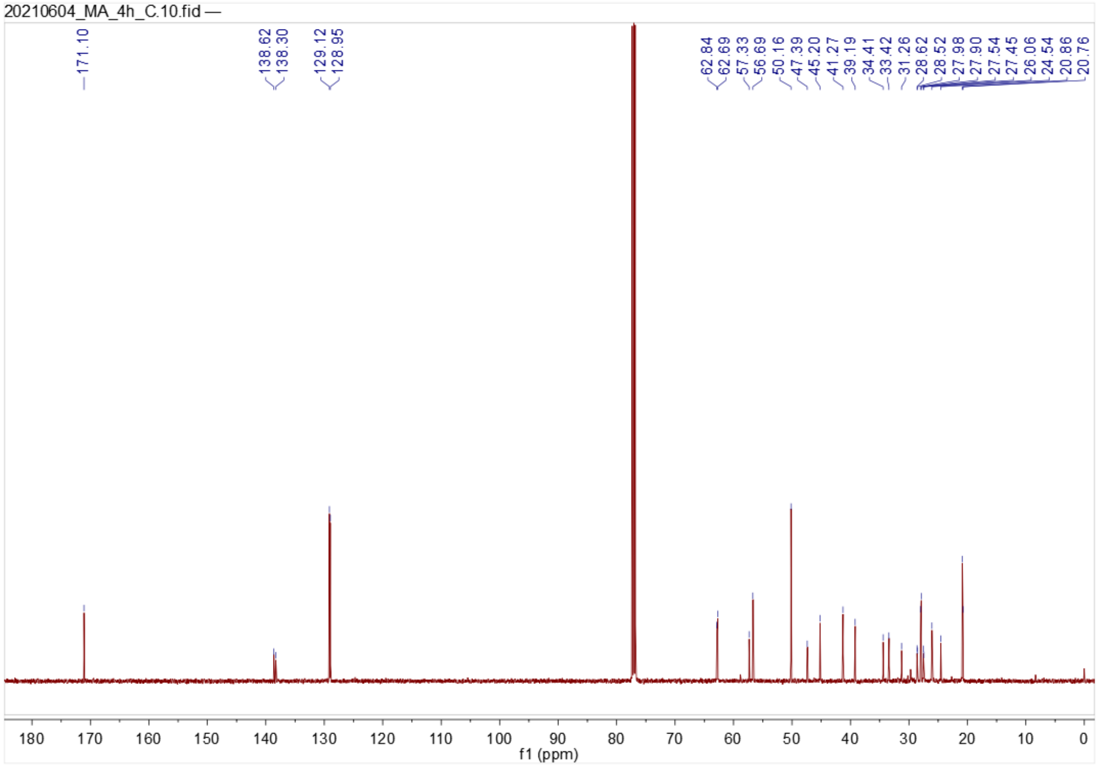

Figure S32. ^{13}C NMR spectrum (Chloroform-*d*, 126 MHz) of **4g**.

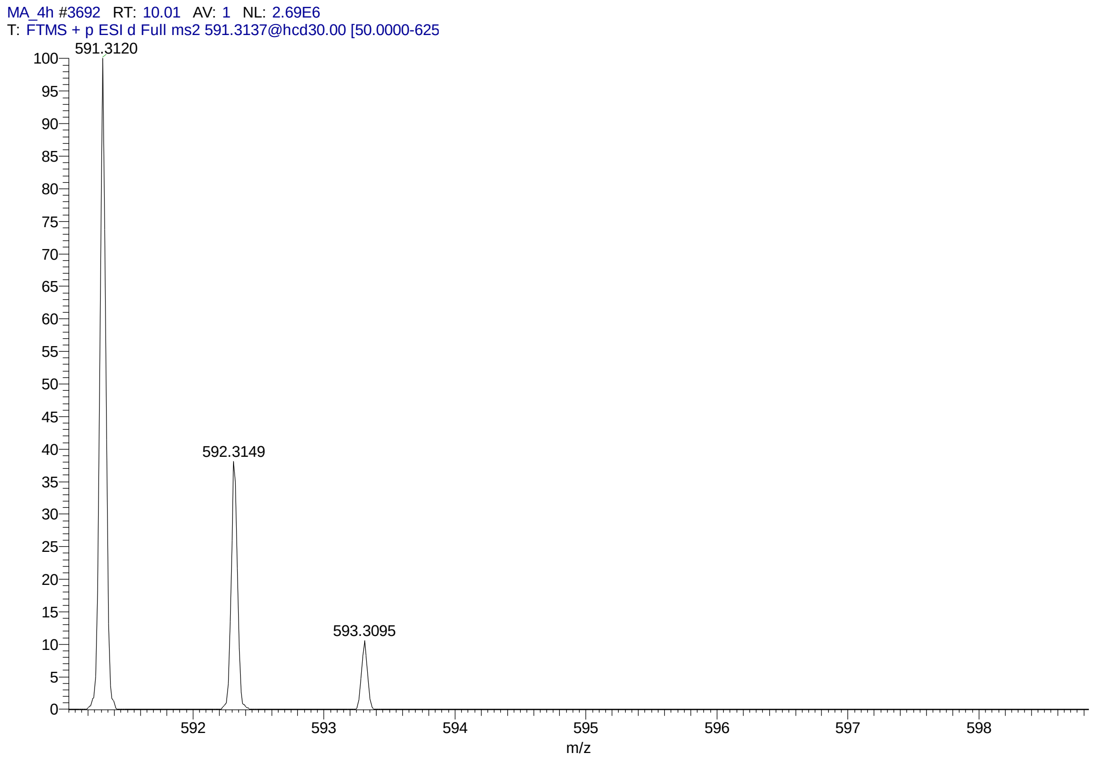

Figure S33. HRMS spectrum of **4g**.

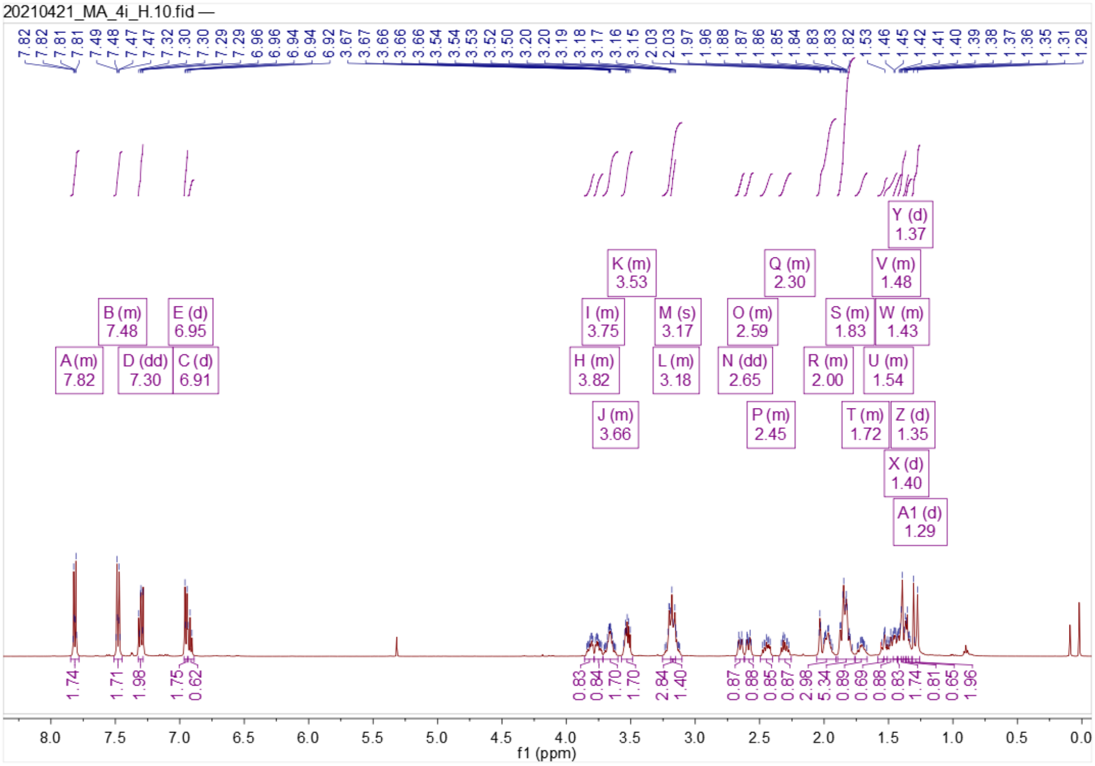

Figure S34. ^{1}H NMR spectrum (Chloroform-*d*, 500 MHz) of **4h**.

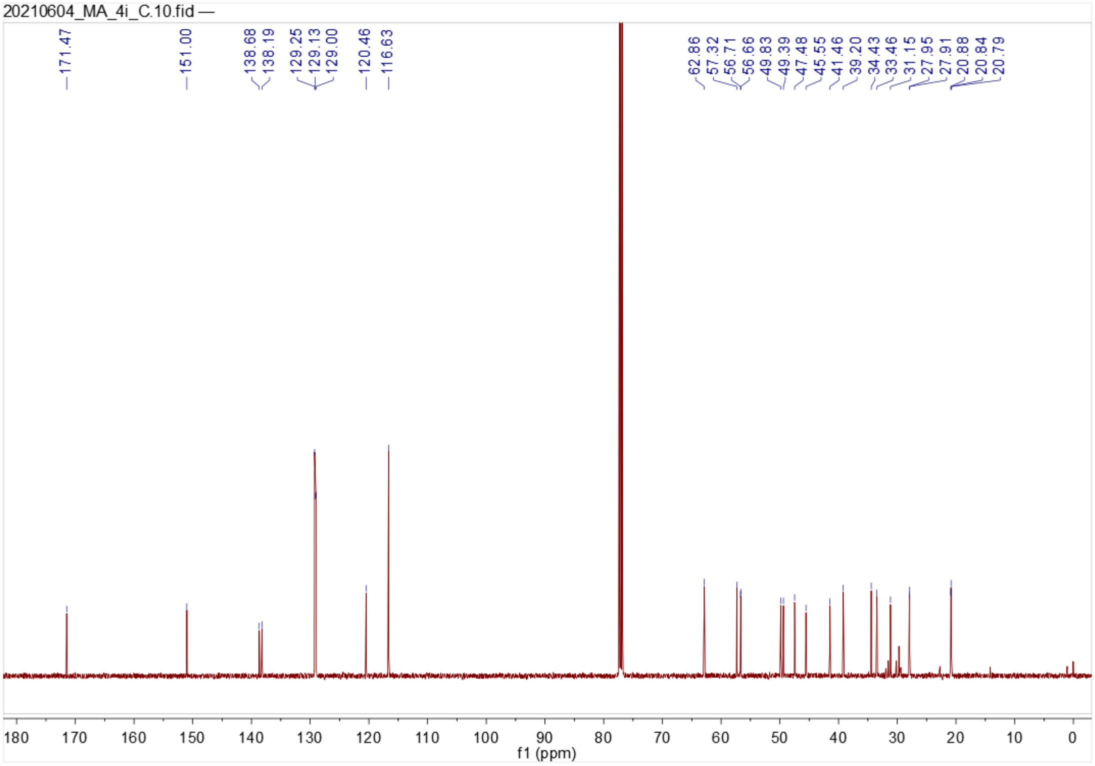

Figure S35. ^{13}C NMR spectrum (Chloroform-*d*, 126 MHz) of **4h**.

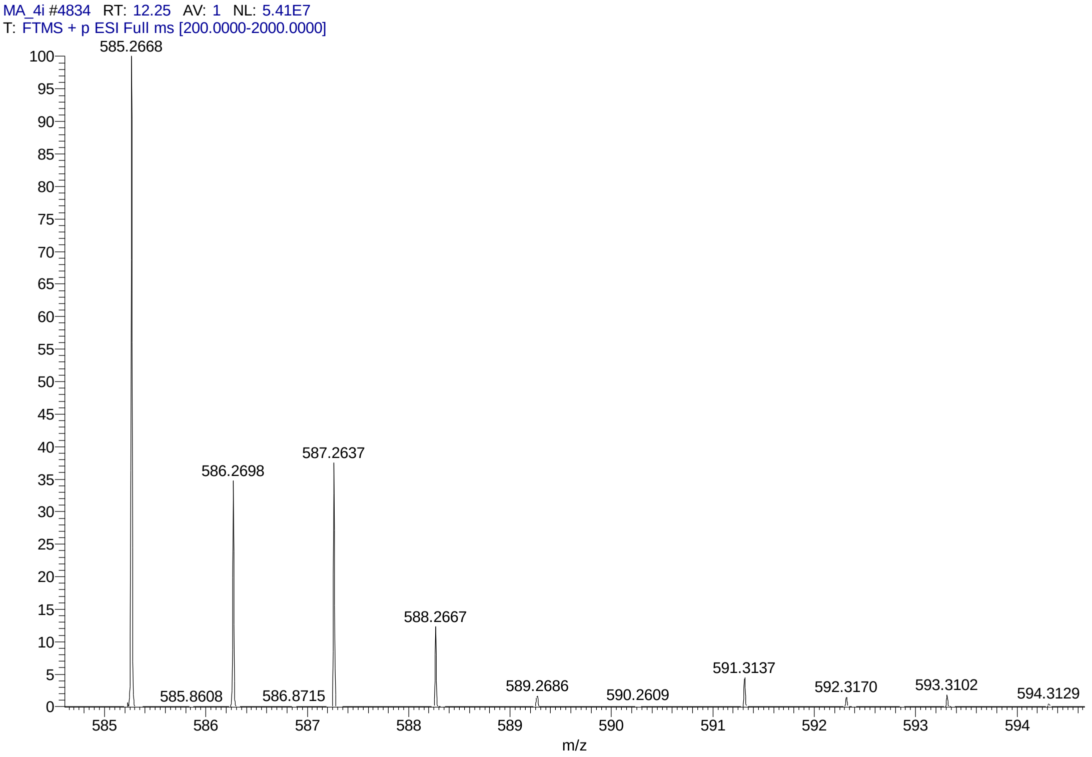

Figure S36. HRMS spectrum of **4h**.

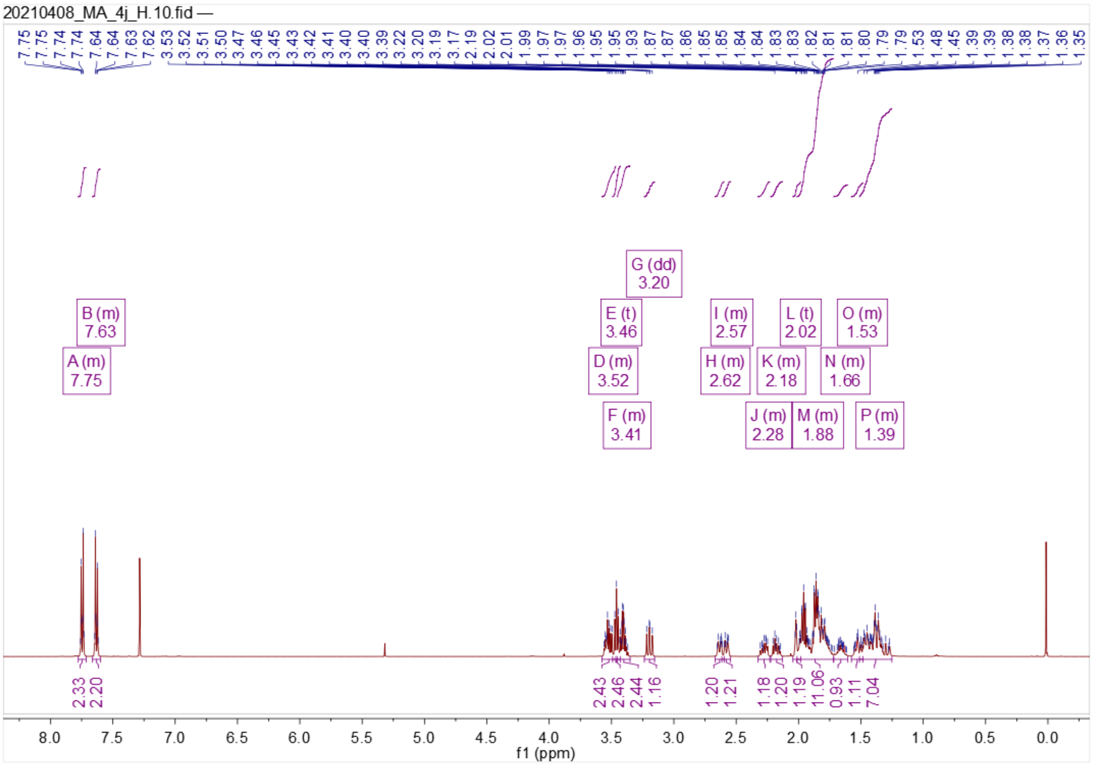

Figure S37. ^{1}H NMR spectrum (Chloroform-*d*, 500 MHz) of **4i**.

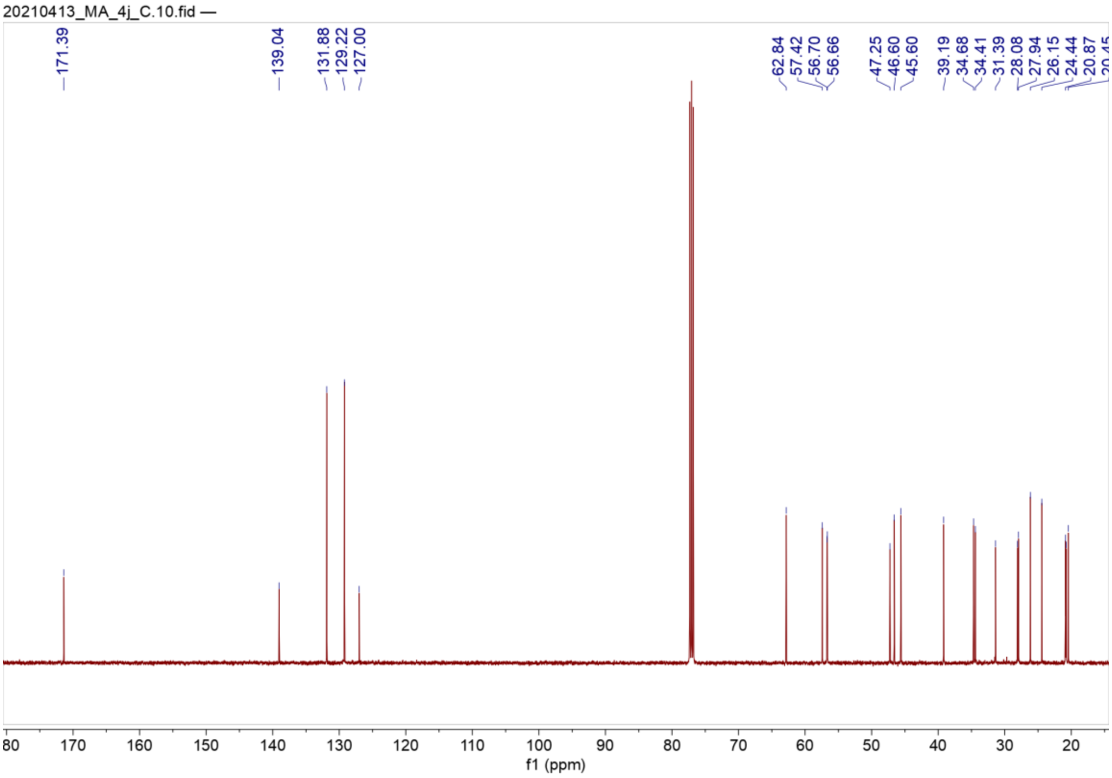

Figure S38. ^{13}C NMR spectrum (Chloroform-*d*, 126 MHz) of **4i**.

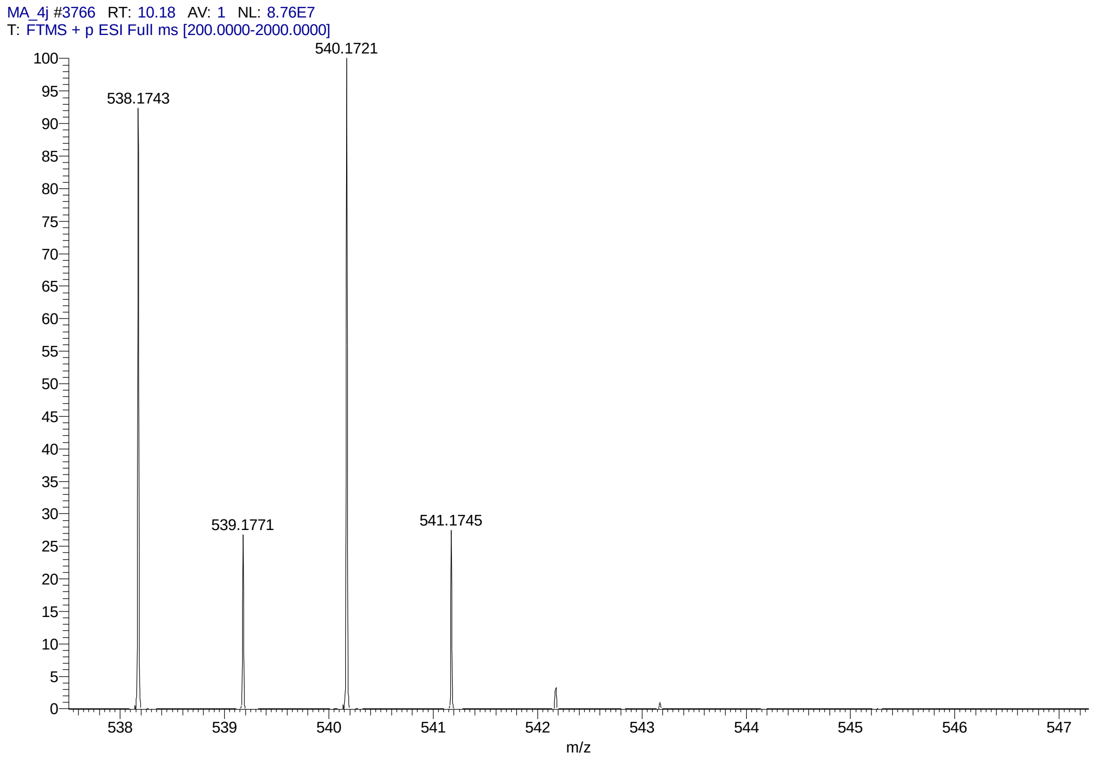

Figure S39. HRMS spectrum of **4i**.

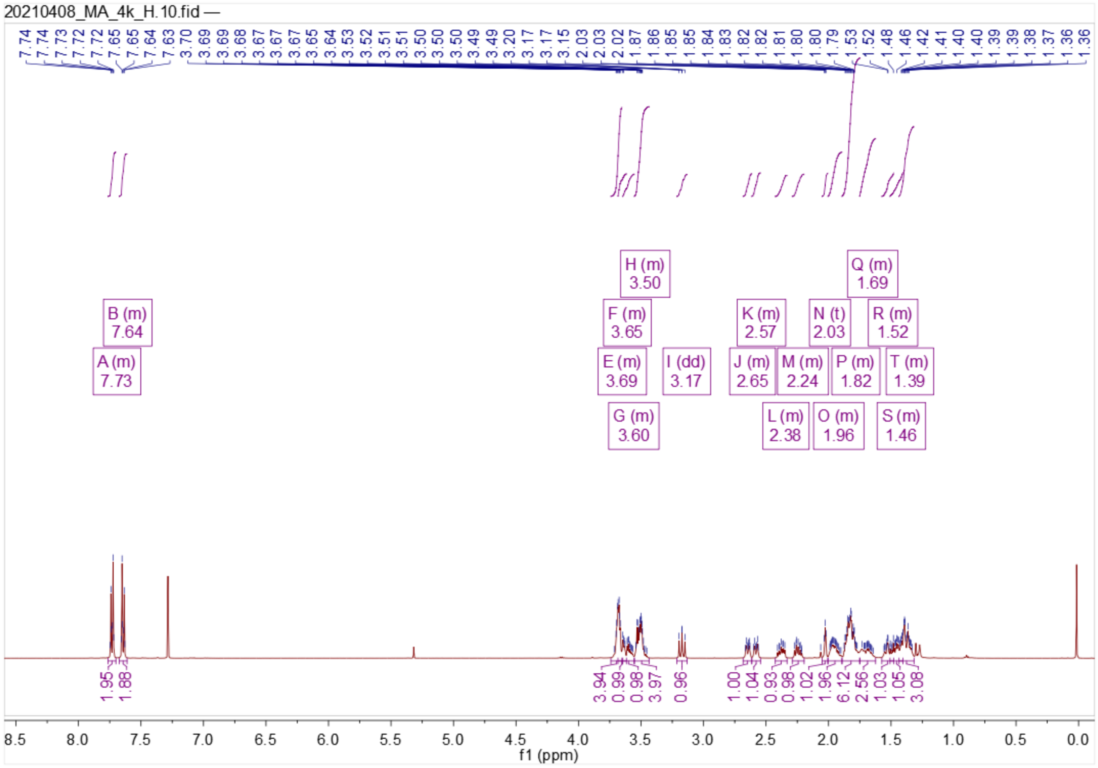

Figure S40. ^{1}H NMR spectrum (Chloroform-*d*, 500 MHz) of **4j**.

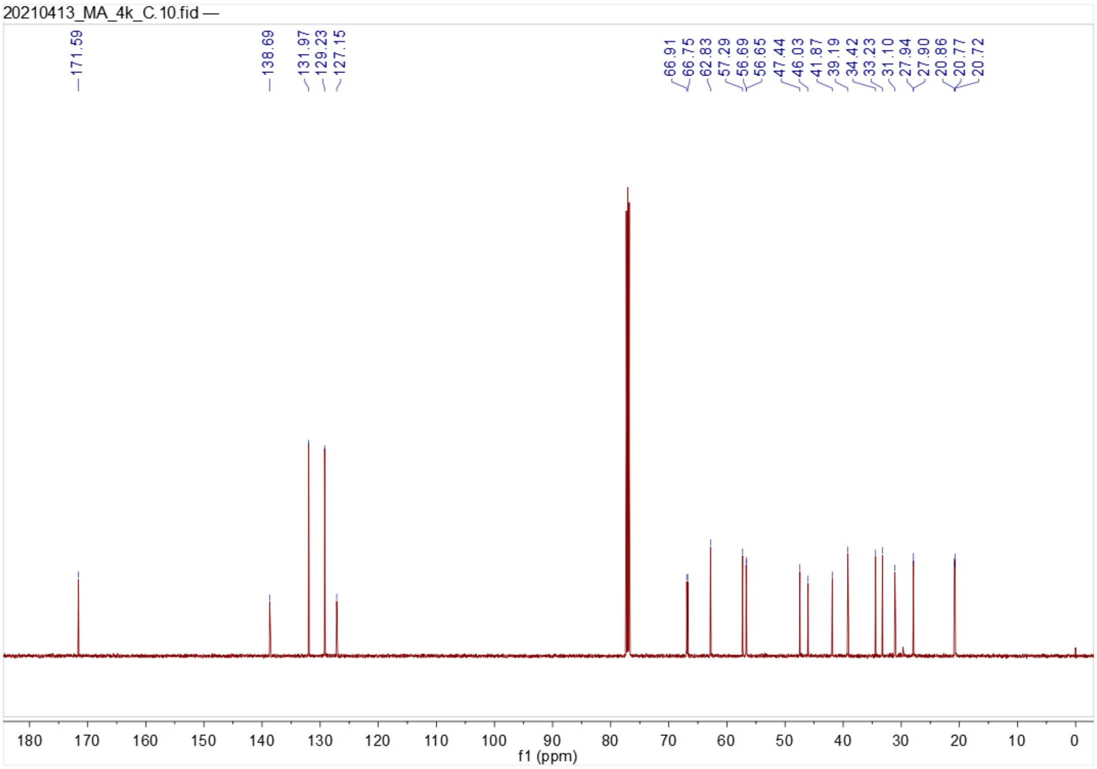

Figure S41. ^{13}C NMR spectrum (Chloroform-*d*, 126 MHz) of **4j**.

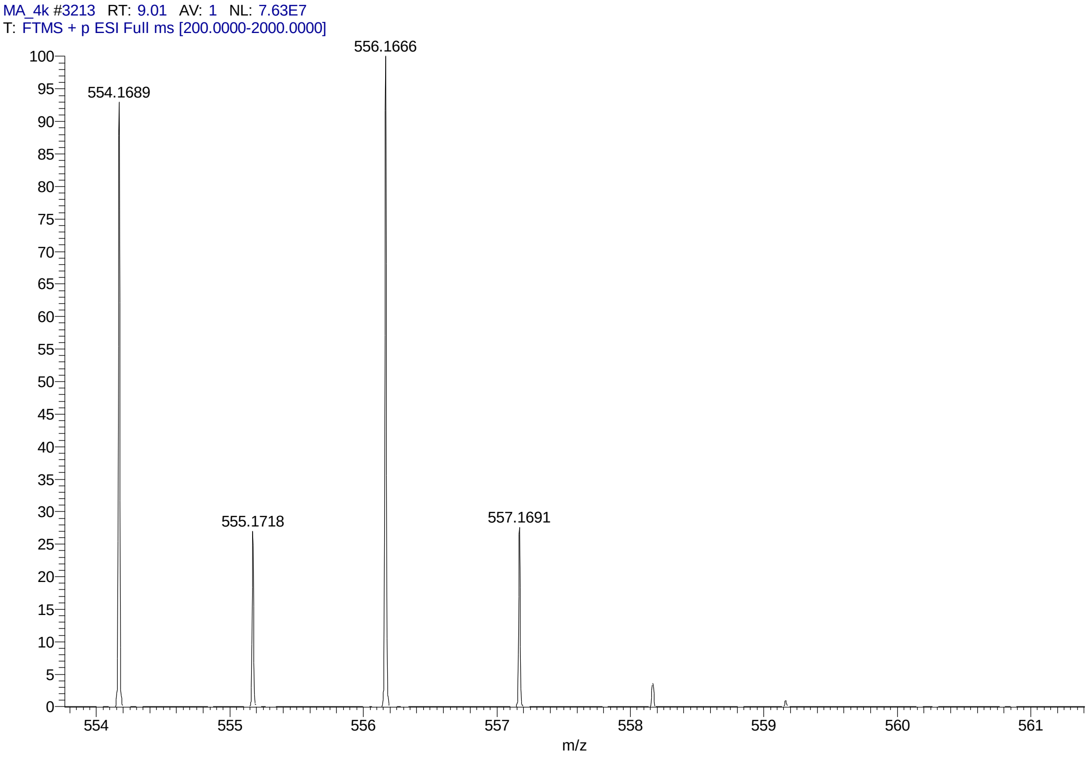

Figure S42. HRMS spectrum of **4j**.

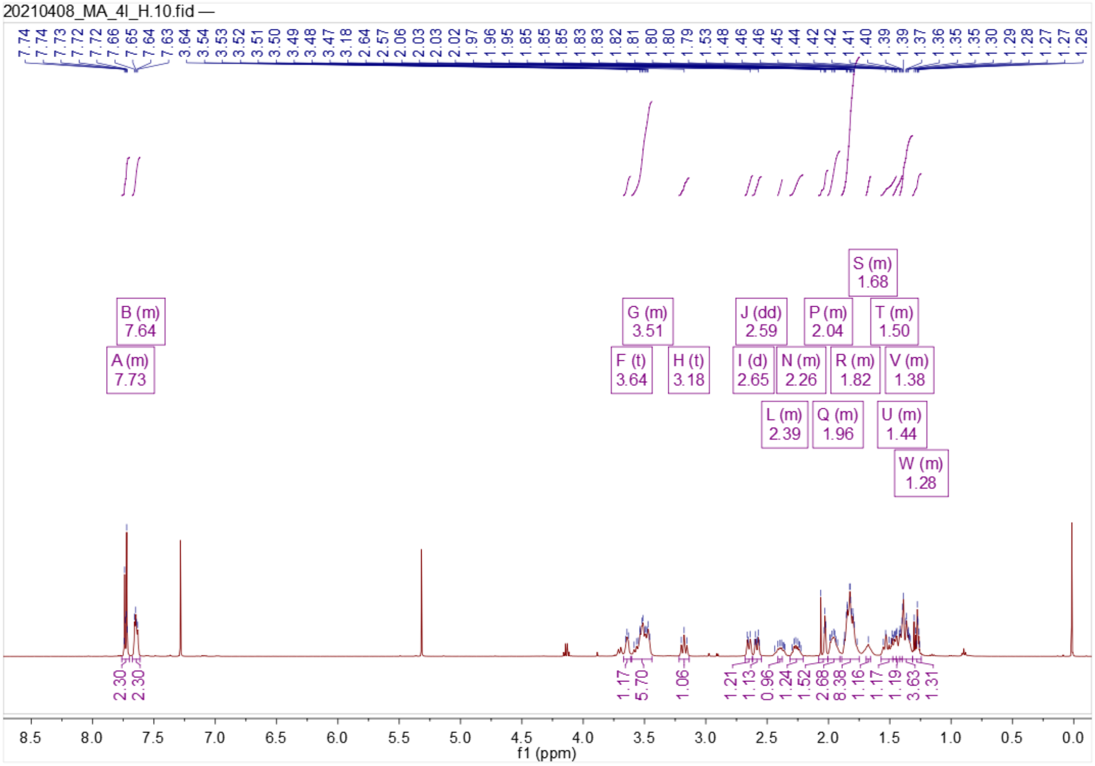

Figure S43. ^{1}H NMR spectrum (Chloroform-*d*, 500 MHz) of **4k**.

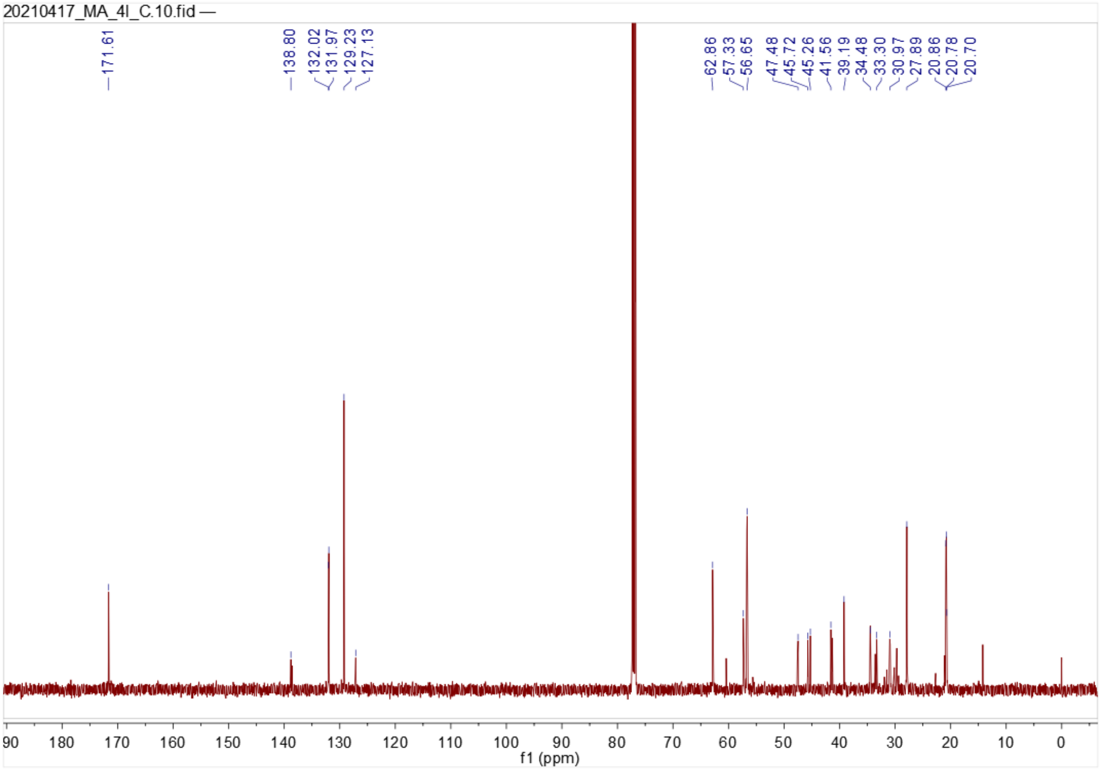

Figure S44. ^{13}C NMR spectrum (Chloroform-*d*, 126 MHz) of **4k**.

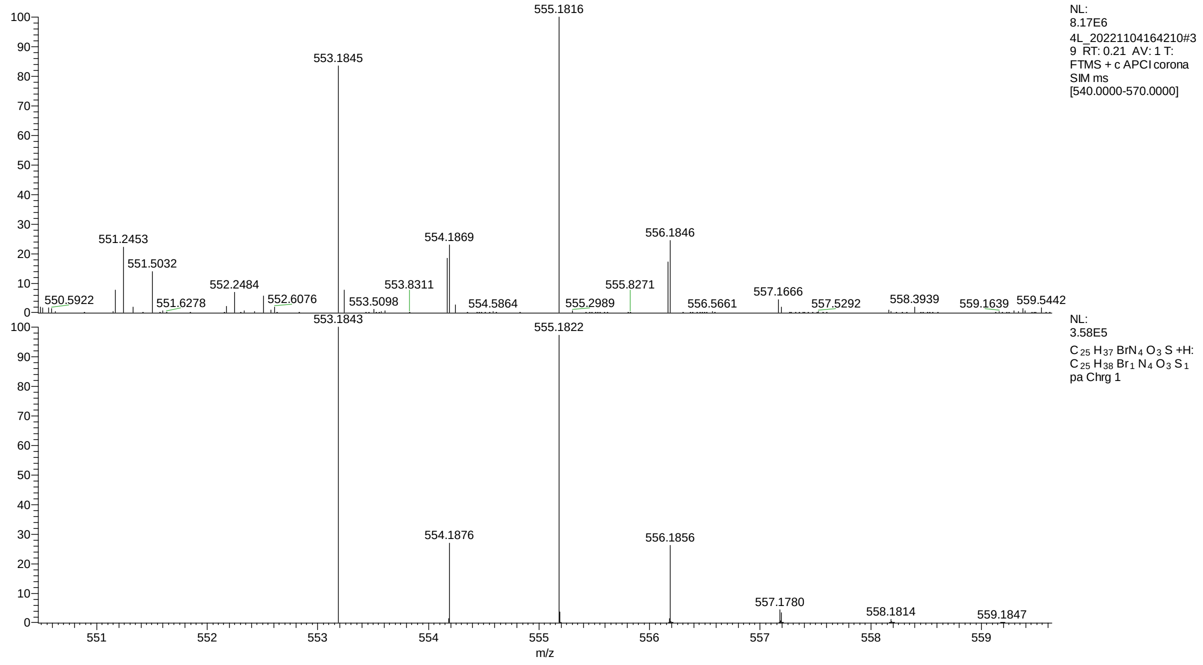

Figure S45. HRMS spectrum of **4k**.

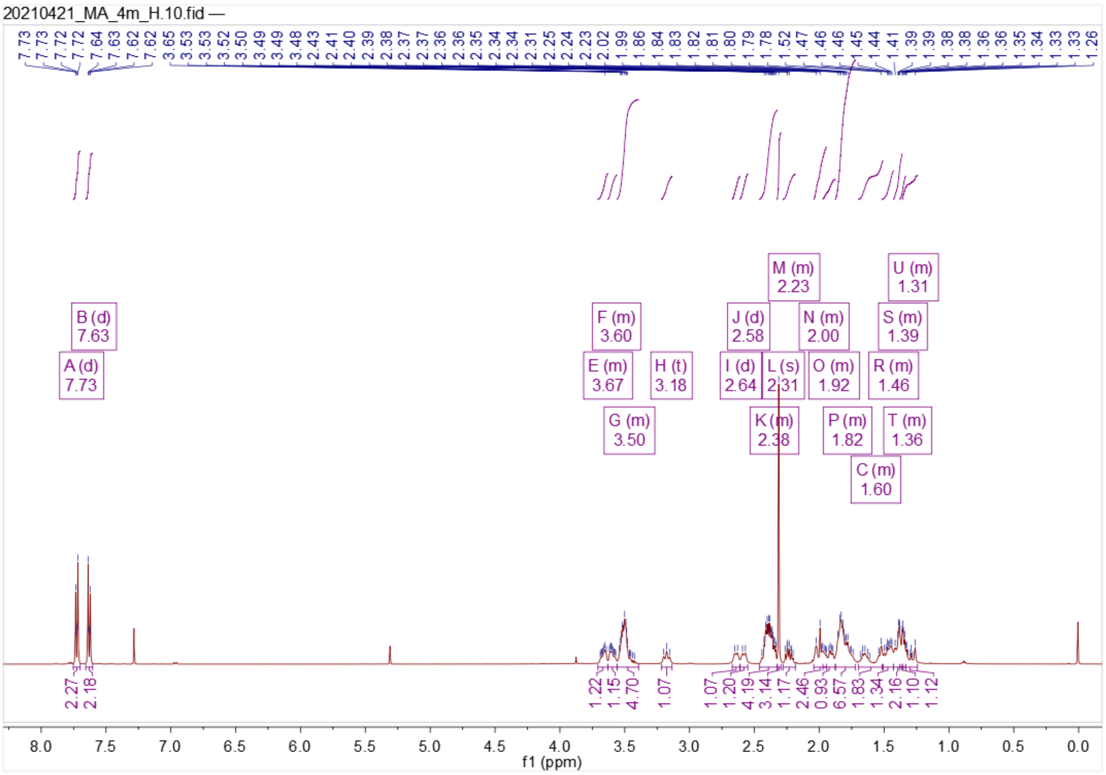

Figure S46. ¹H NMR spectrum (Chloroform-*d*, 500 MHz) of **4l**.

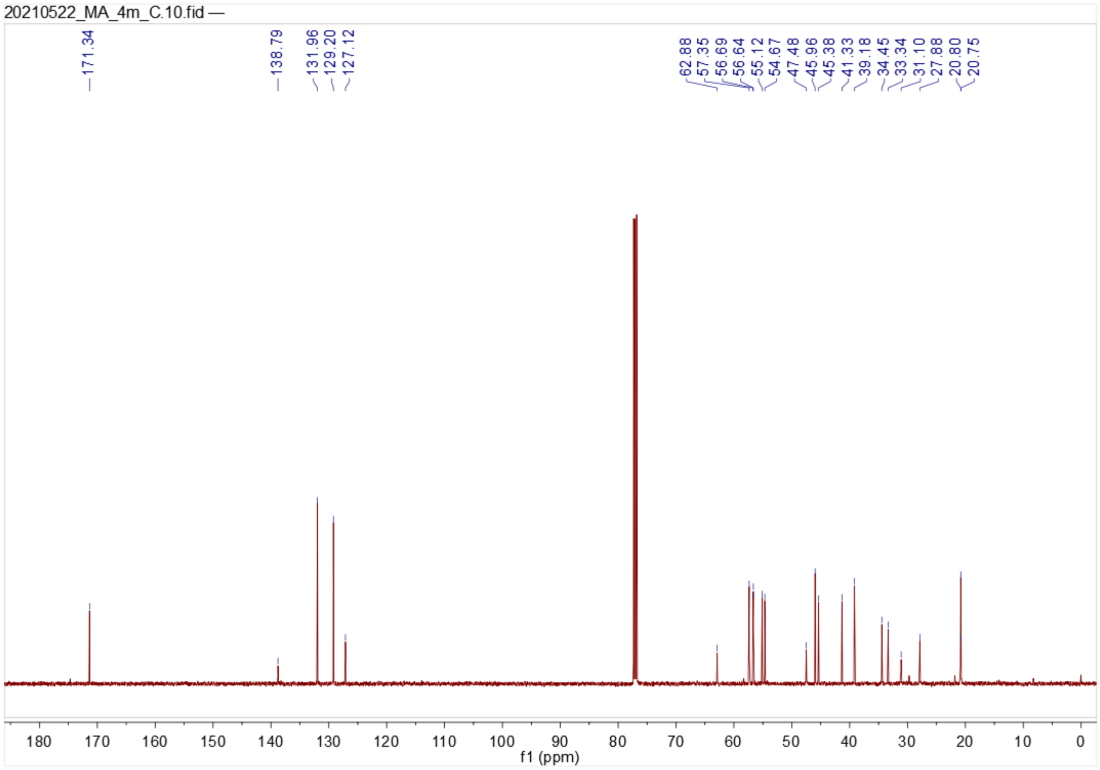

Figure S47. ^{13}C NMR spectrum (Chloroform-*d*, 126 MHz) of **4l**.

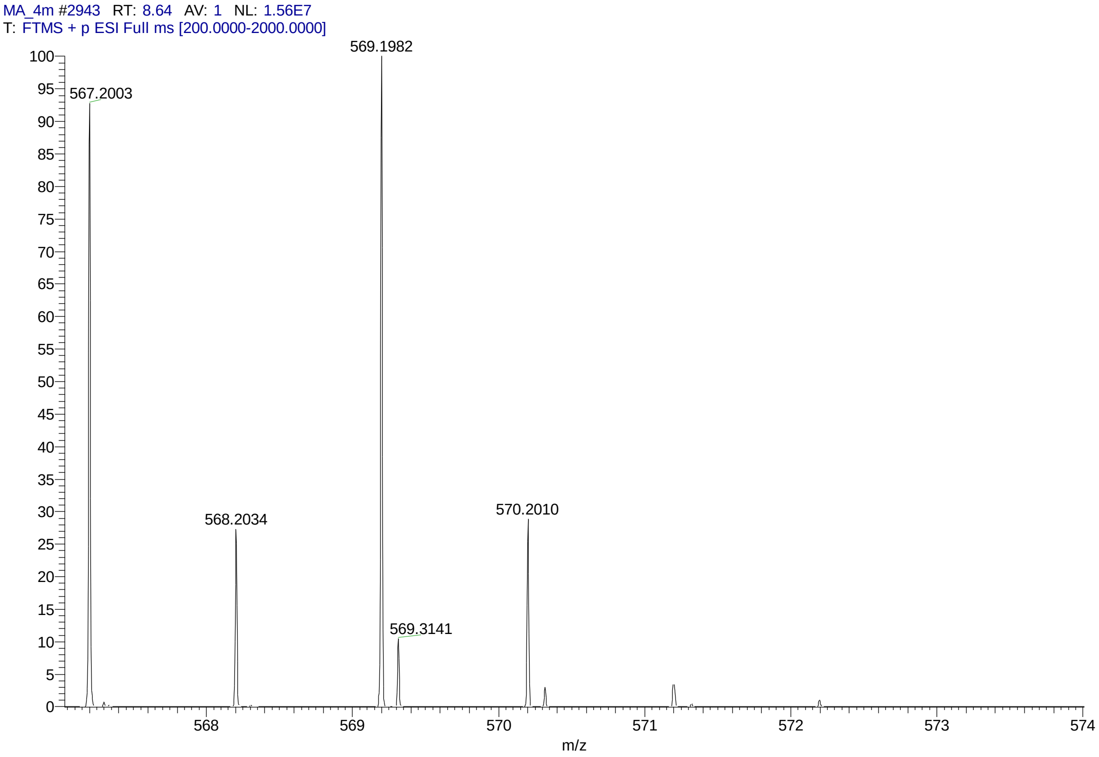

Figure S48. HRMS spectrum of **4l**.

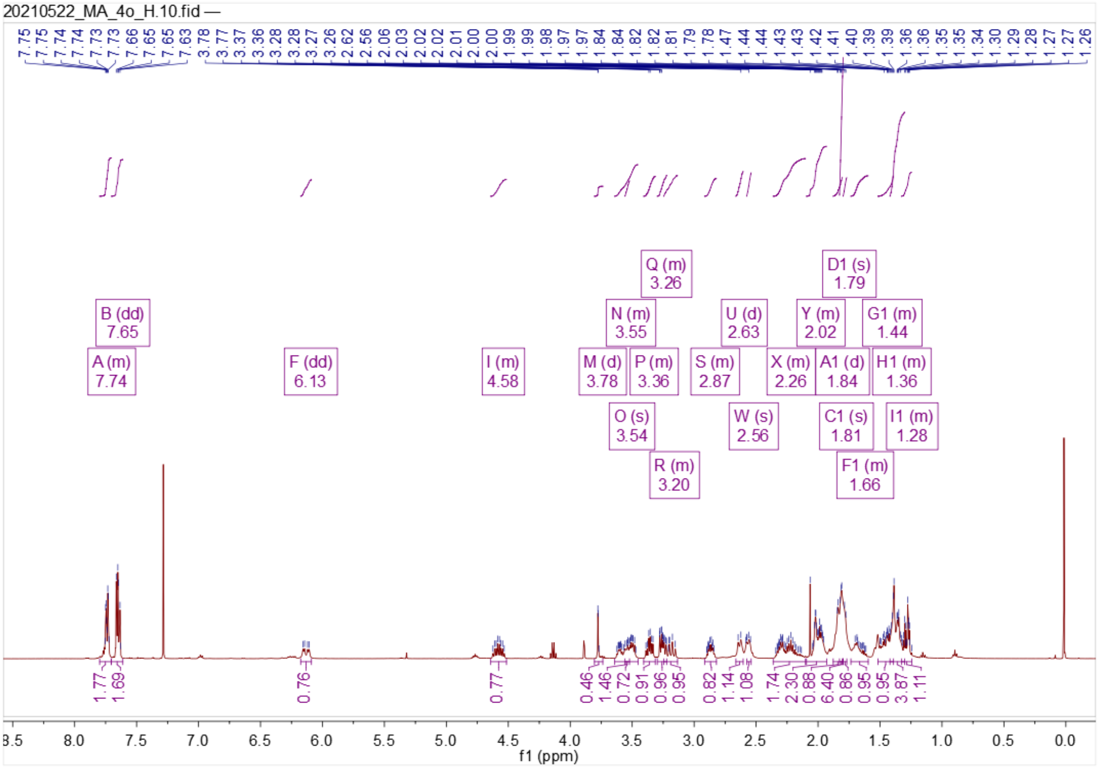

Figure S49. ^{1}H NMR spectrum (Chloroform-*d*, 500 MHz) of **4m**.

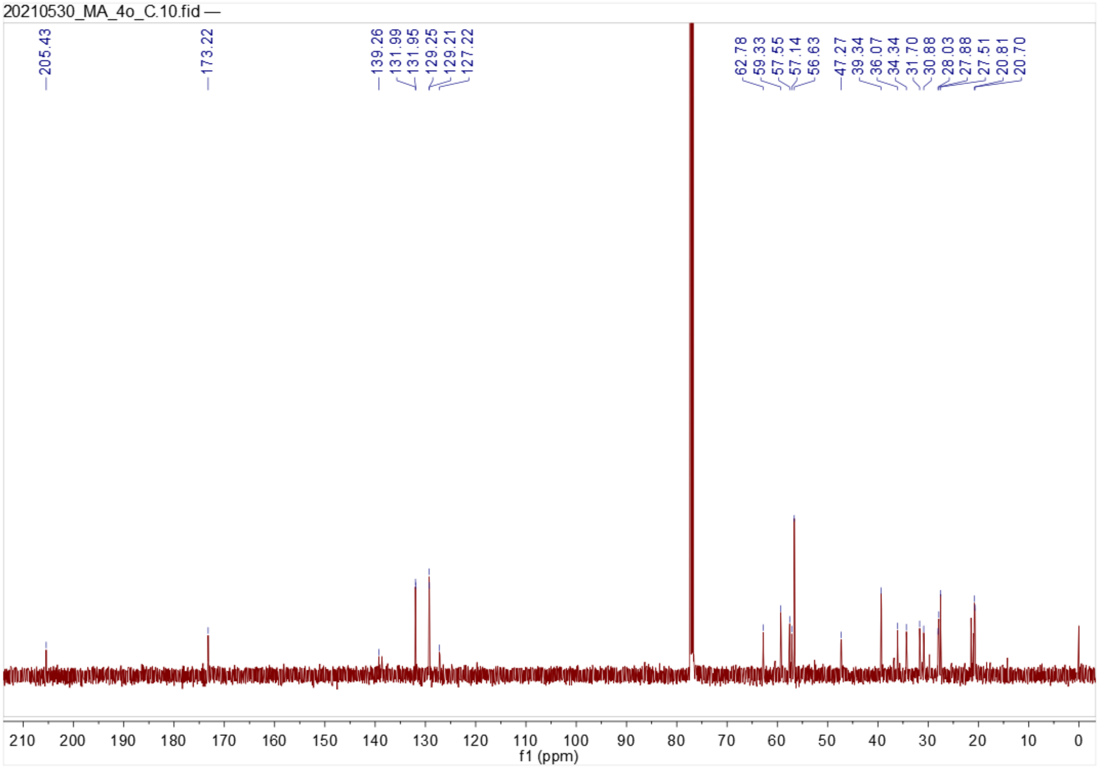

Figure S50. ^{13}C NMR spectrum (Chloroform-*d*, 126 MHz) of **4m**.

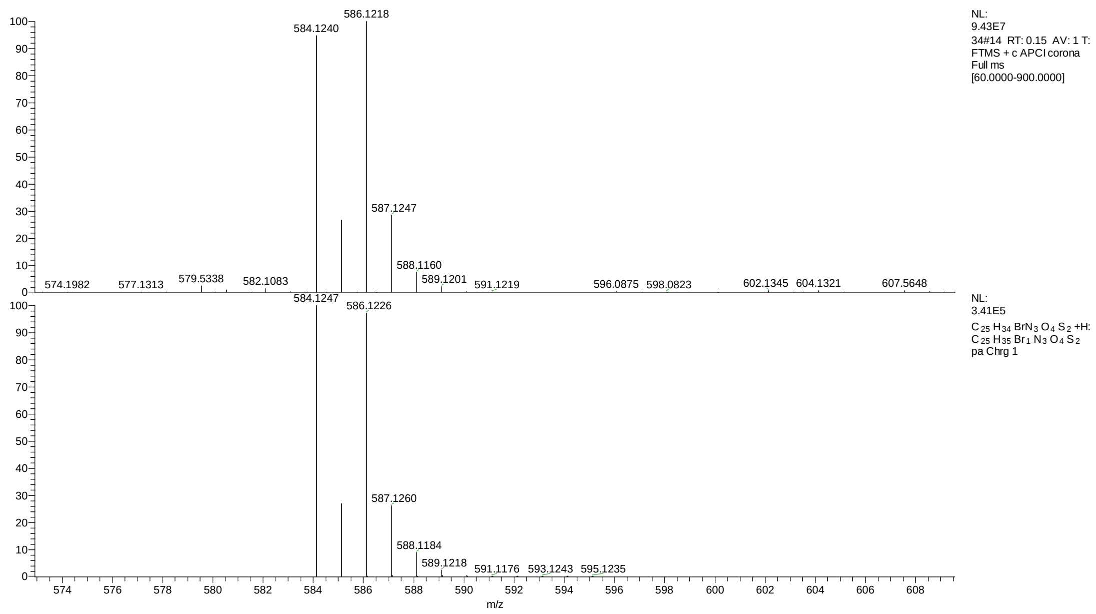

Figure S51. HRMS spectrum of **4m**.

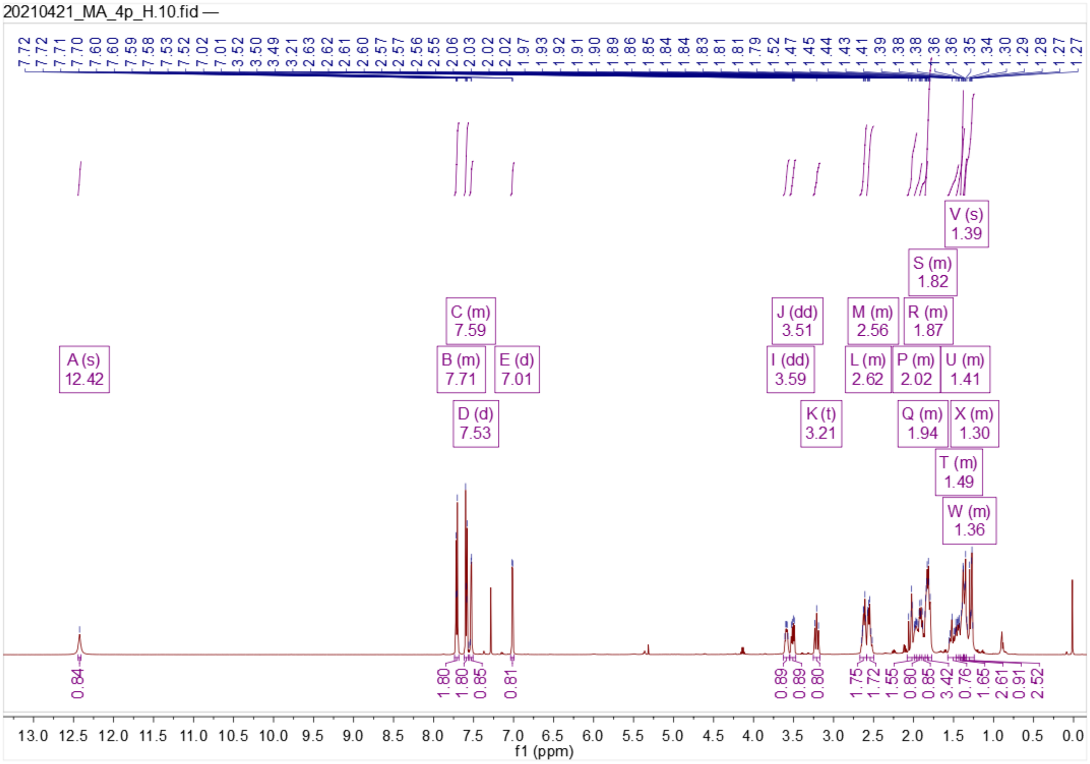

Figure S52. ^{1}H NMR spectrum (Chloroform-*d*, 500 MHz) of **4n**.

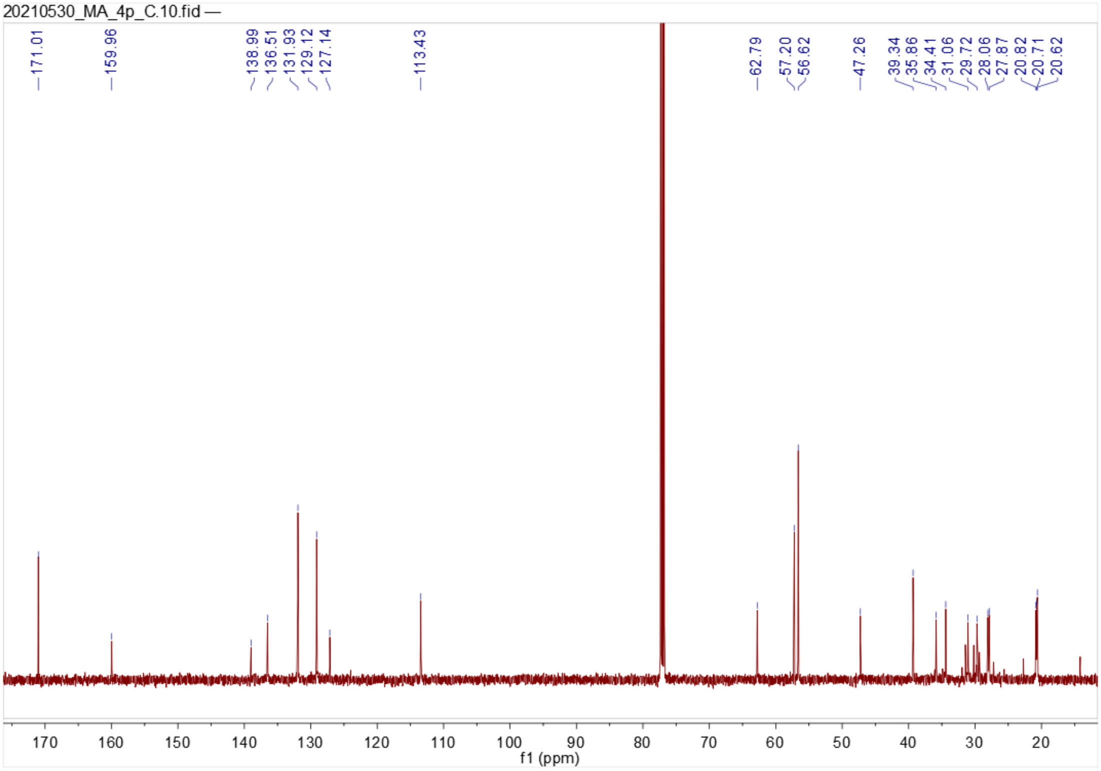

Figure S53. ^{13}C NMR spectrum (Chloroform-*d*, 126 MHz) of **4n**.

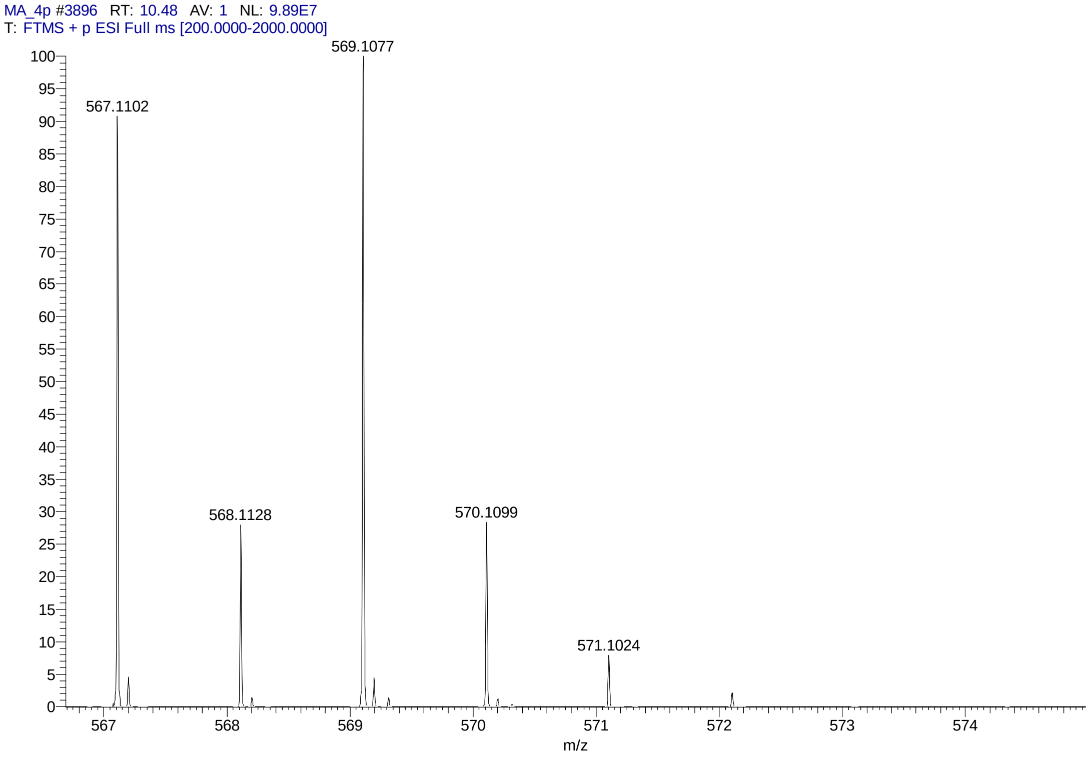

Figure S54. HRMS spectrum of **4n**.

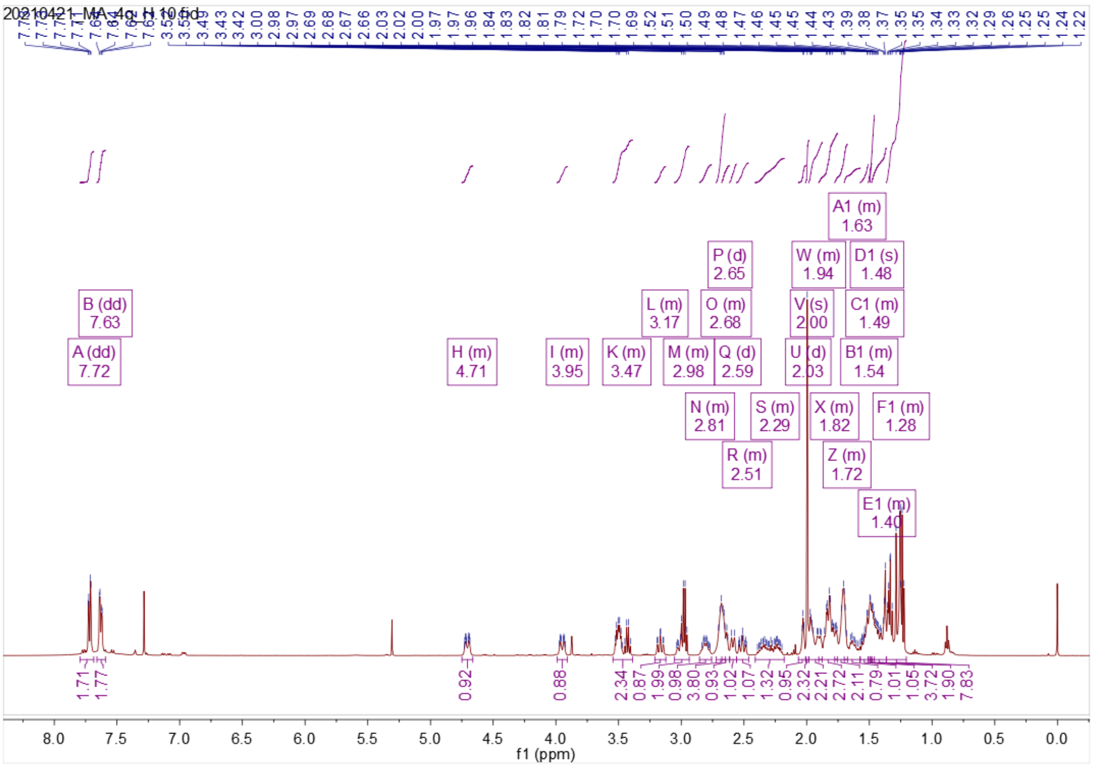

Figure S55. ^{1}H NMR spectrum (Chloroform-*d*, 500 MHz) of **4o**.

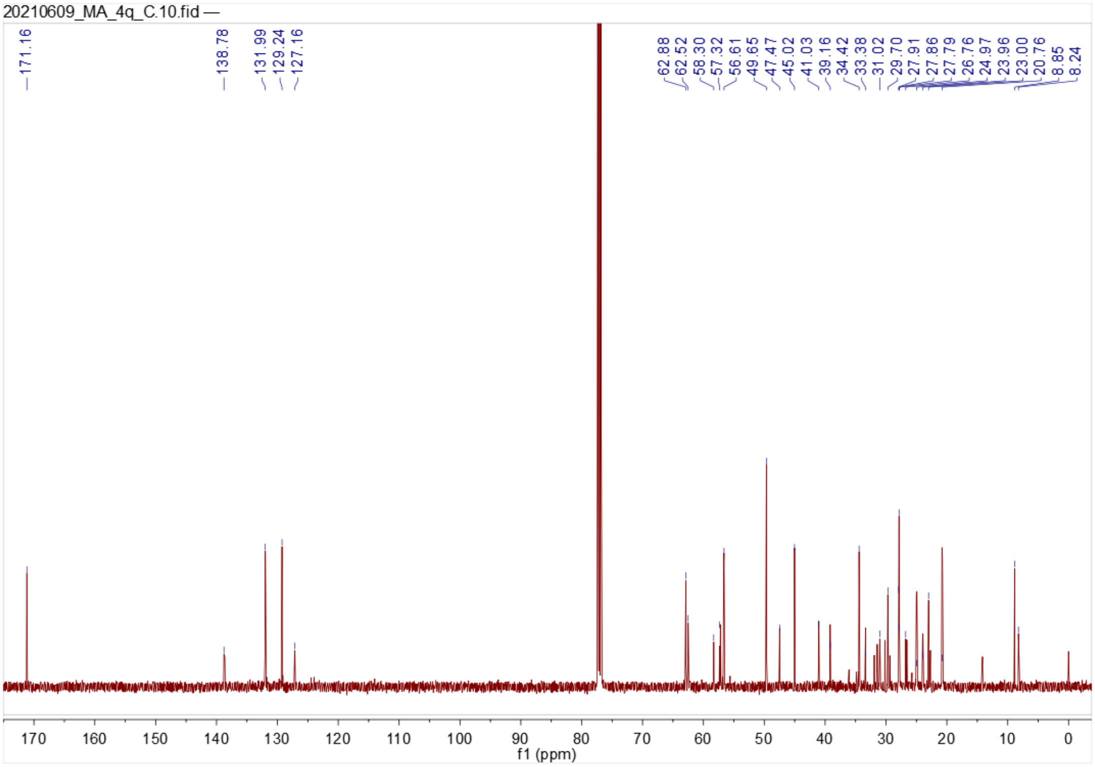

Figure S56. ^{13}C NMR spectrum (Chloroform-*d*, 126 MHz) of **4o**.

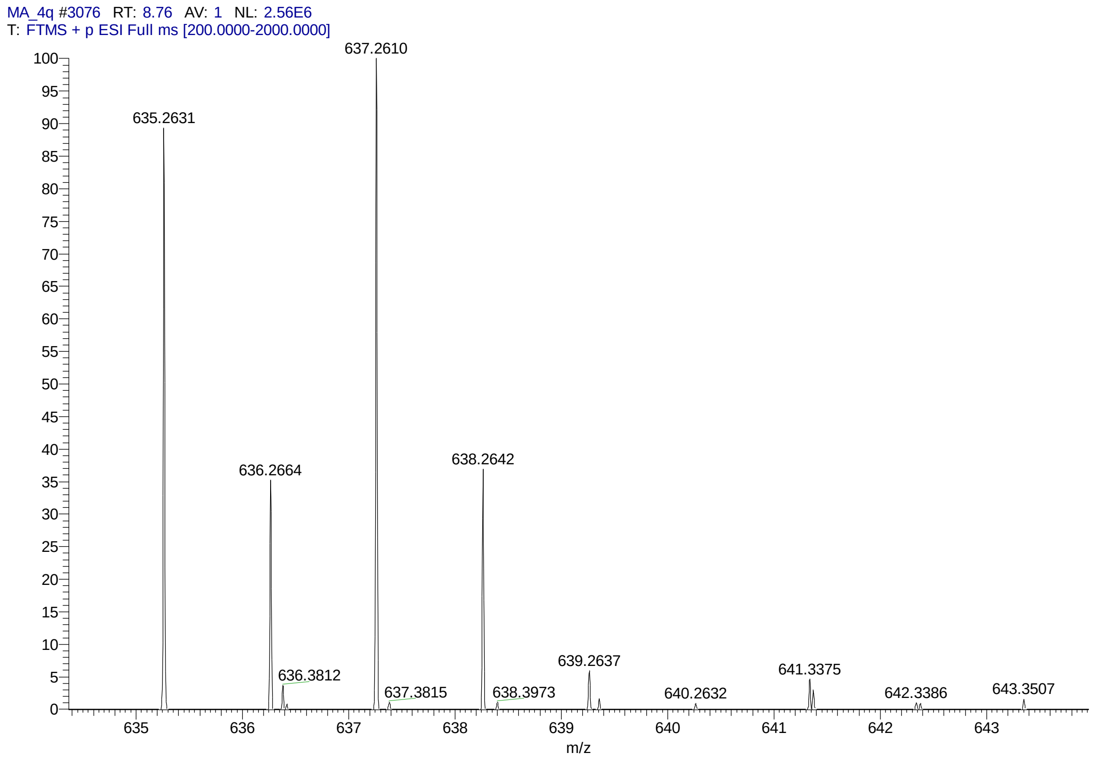

Figure S57. HRMS spectrum of **4o**.

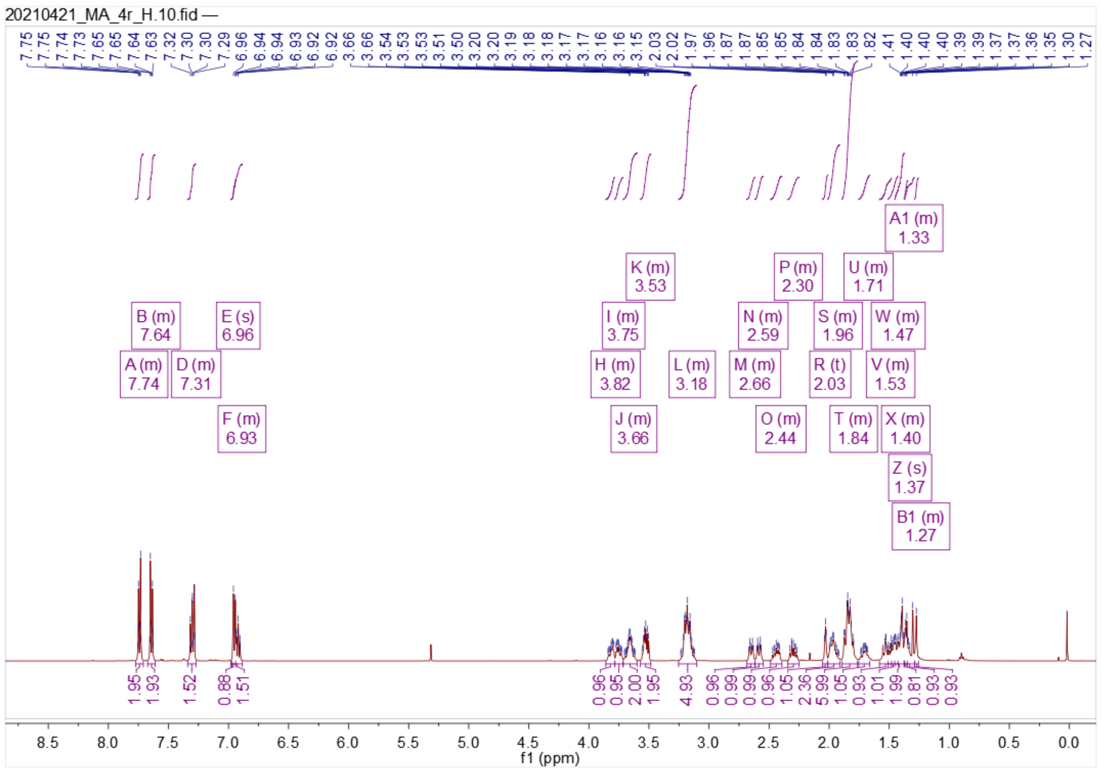

Figure S58. ^{1}H NMR spectrum (Chloroform-*d*, 500 MHz) of **4p**.

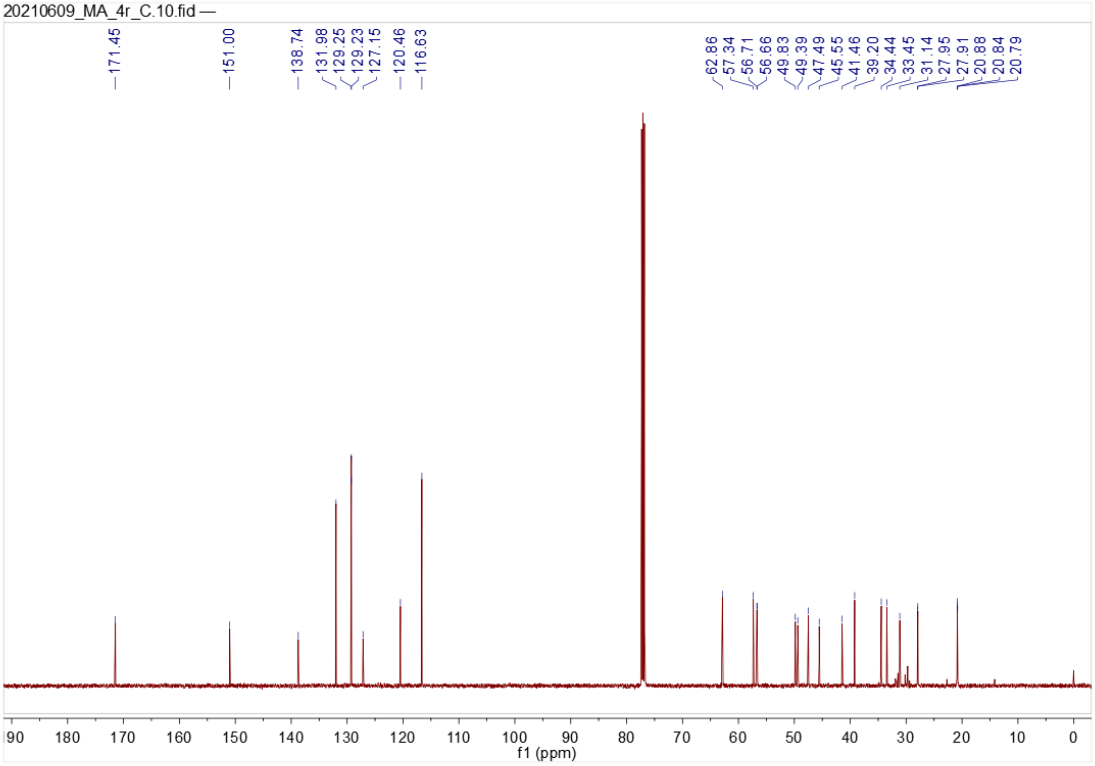

Figure S59. ^{13}C NMR spectrum (Chloroform-*d*, 126 MHz) of **4p**.

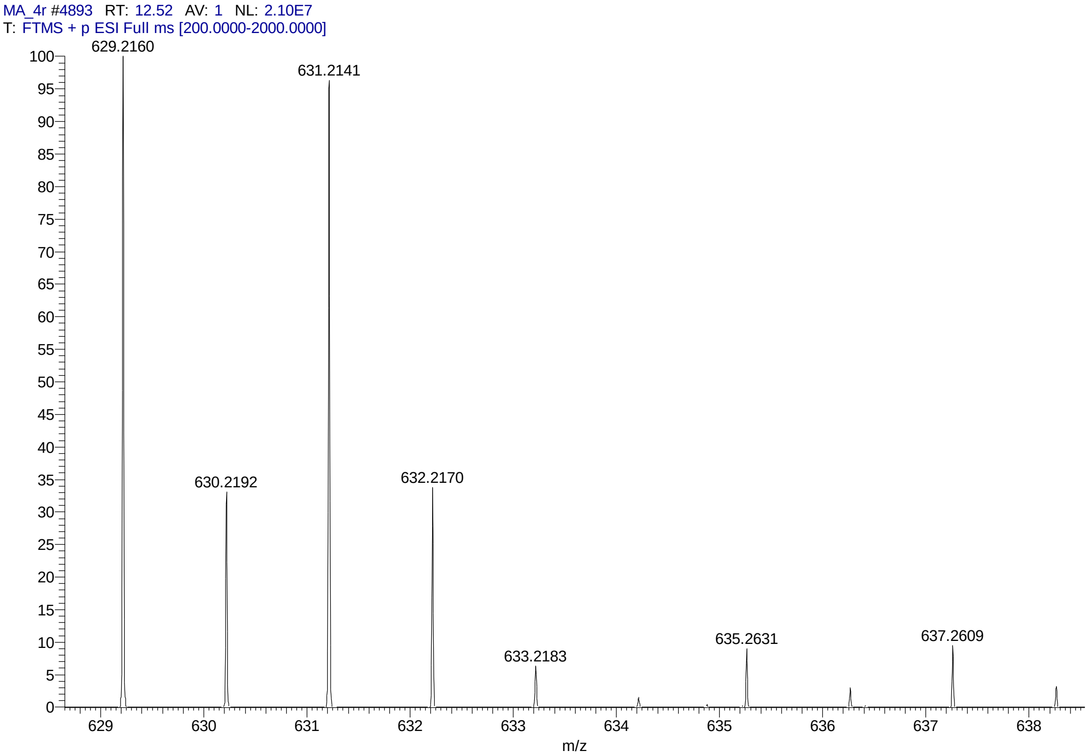

Figure S60. HRMS spectrum of **4p**.

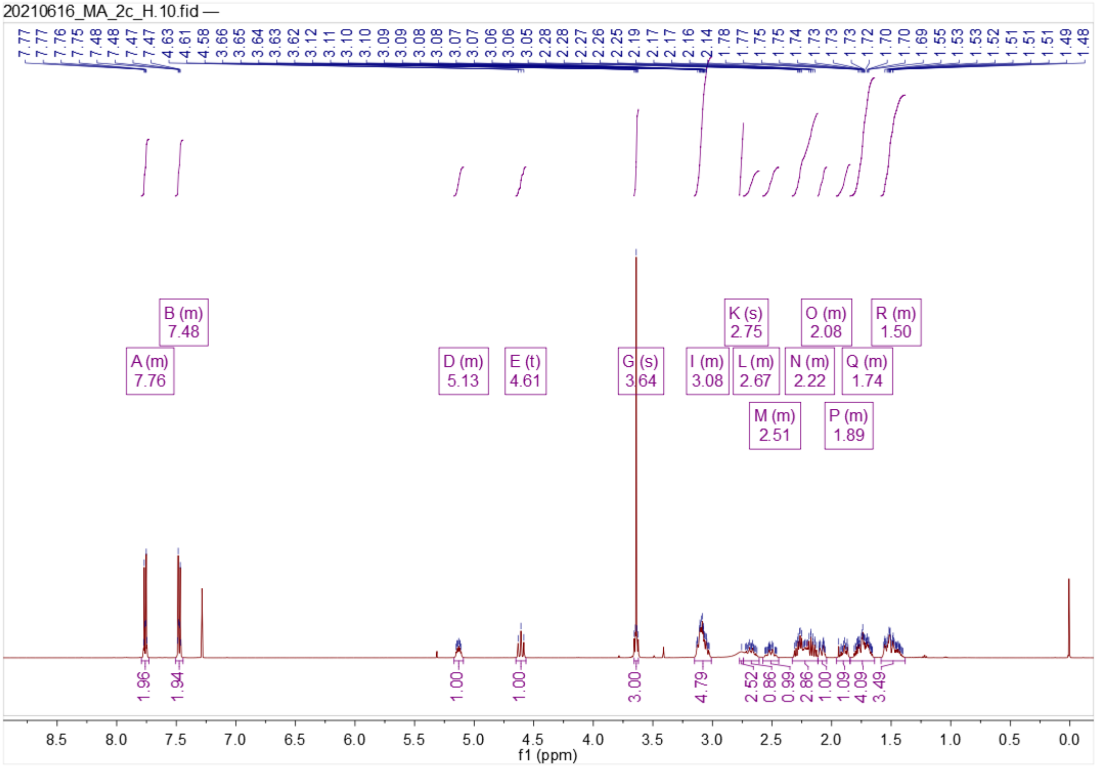

Figure S61. ^{1}H NMR spectrum (Chloroform-*d*, 500 MHz) of **2c**.

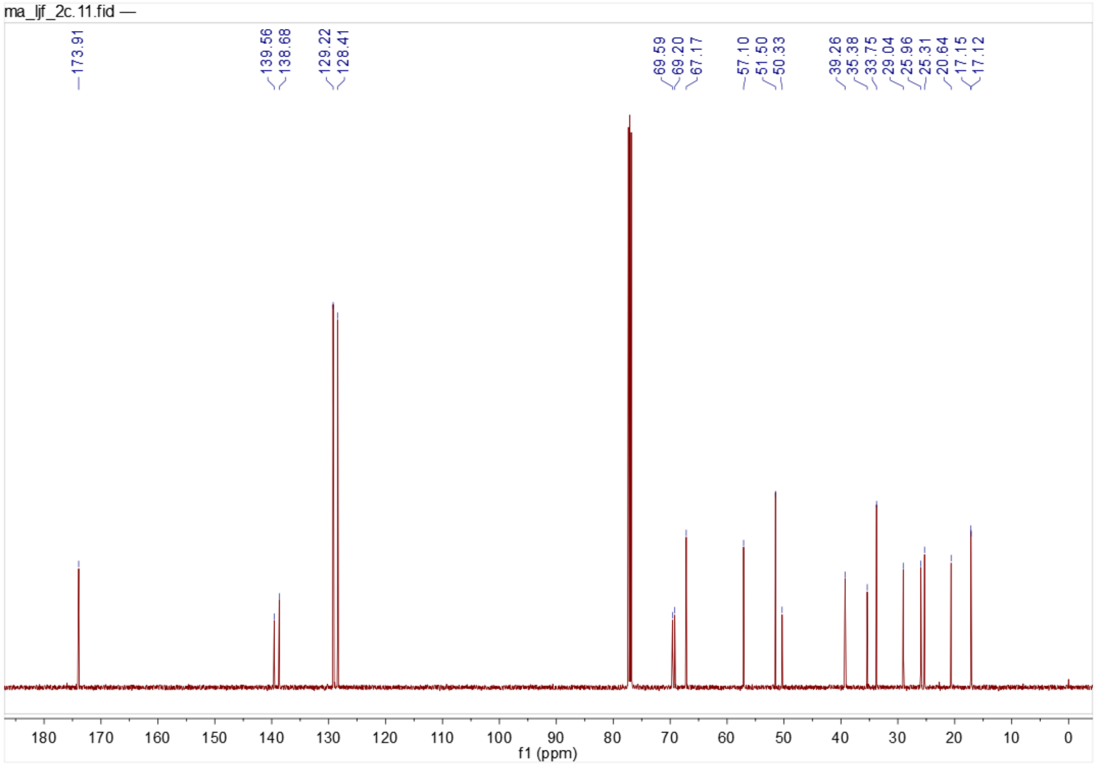

Figure S62. ^{13}C NMR spectrum (Chloroform-*d*, 126 MHz) of **2c**.

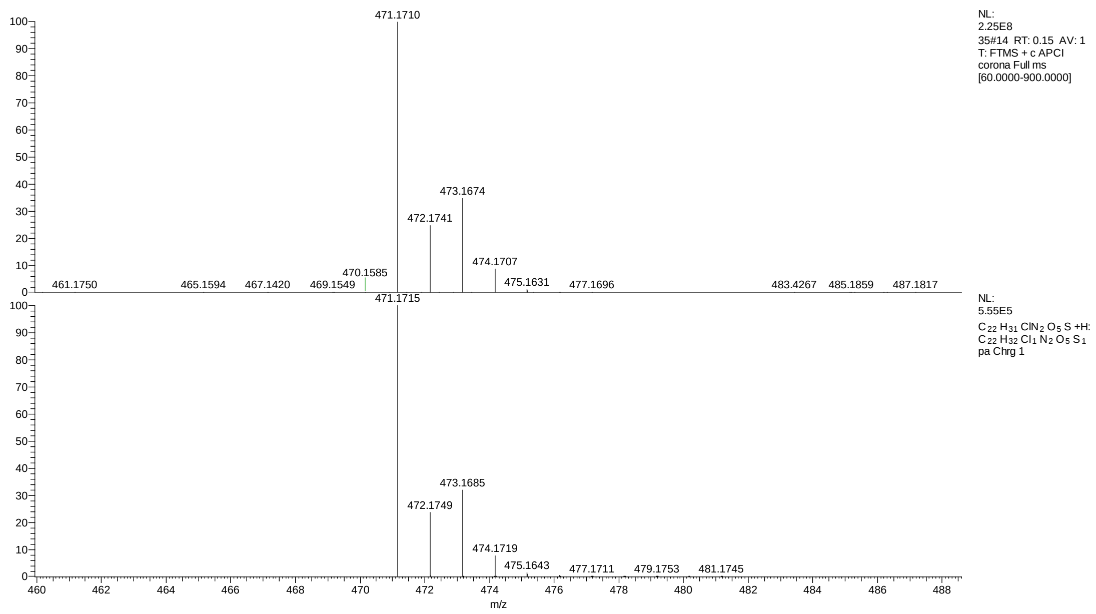

Figure S63. HRMS spectrum of **2c**.

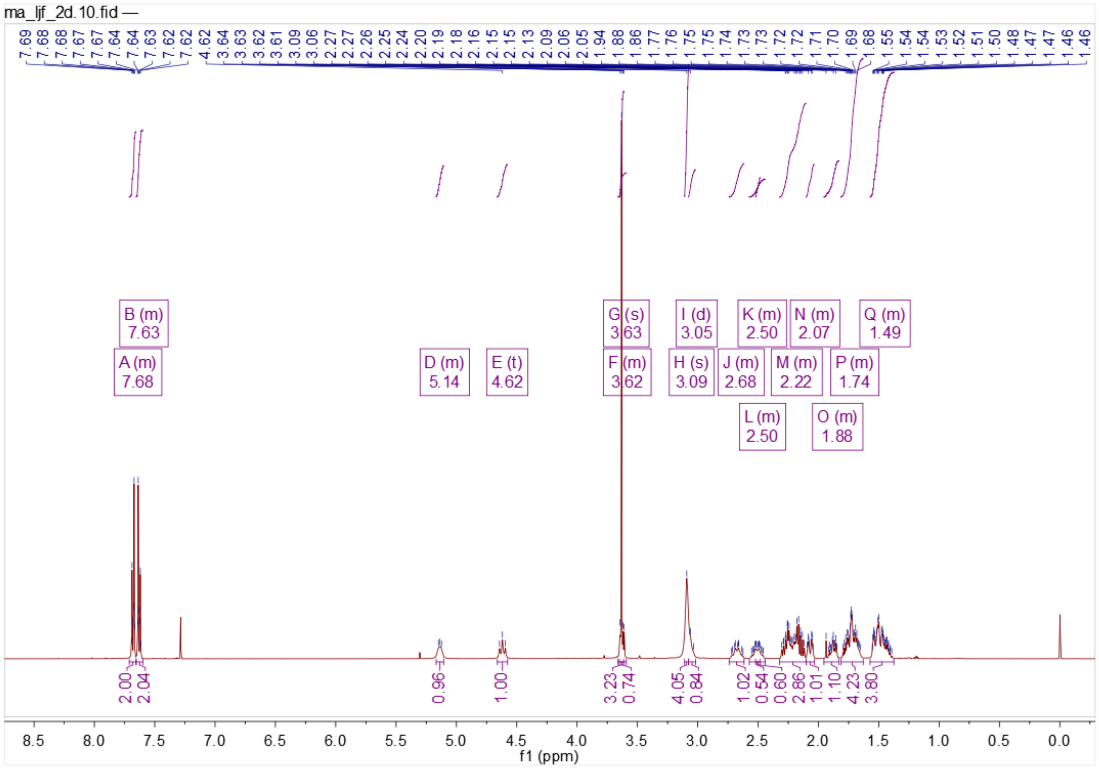

Figure S64. ^{1}H NMR spectrum (Chloroform-*d*, 500 MHz) of **2d**.

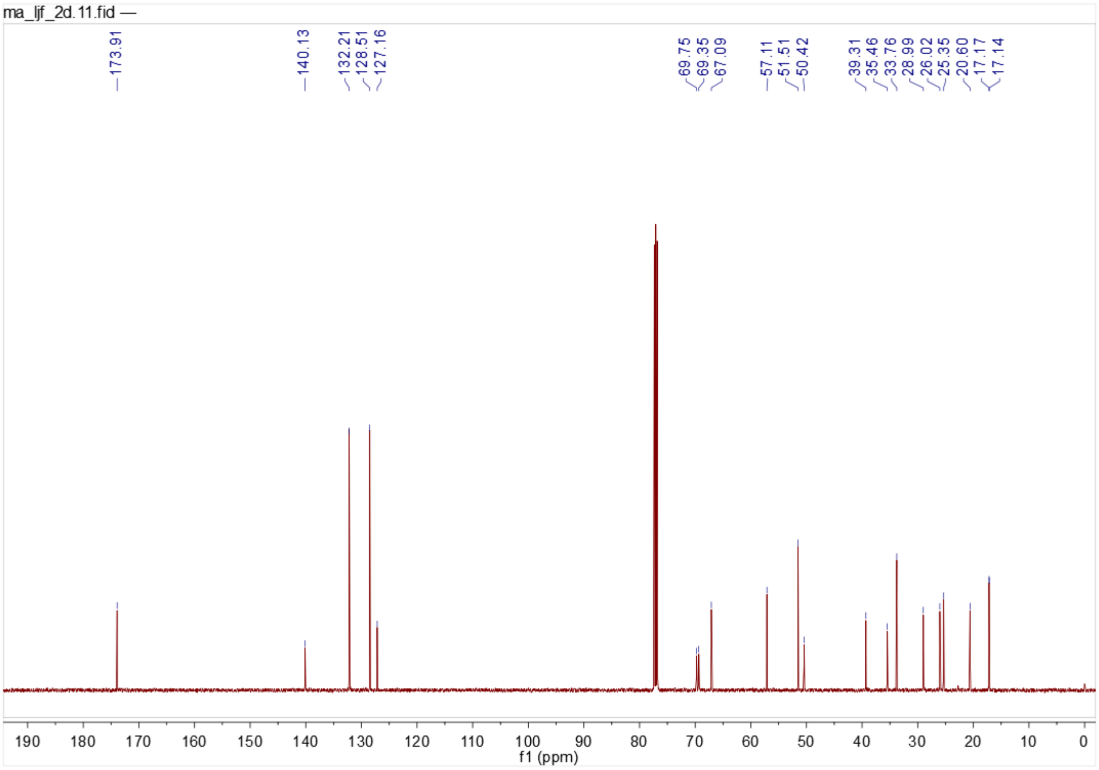

Figure S65. ^{13}C NMR spectrum (Chloroform-*d*, 126 MHz) of **2d**.

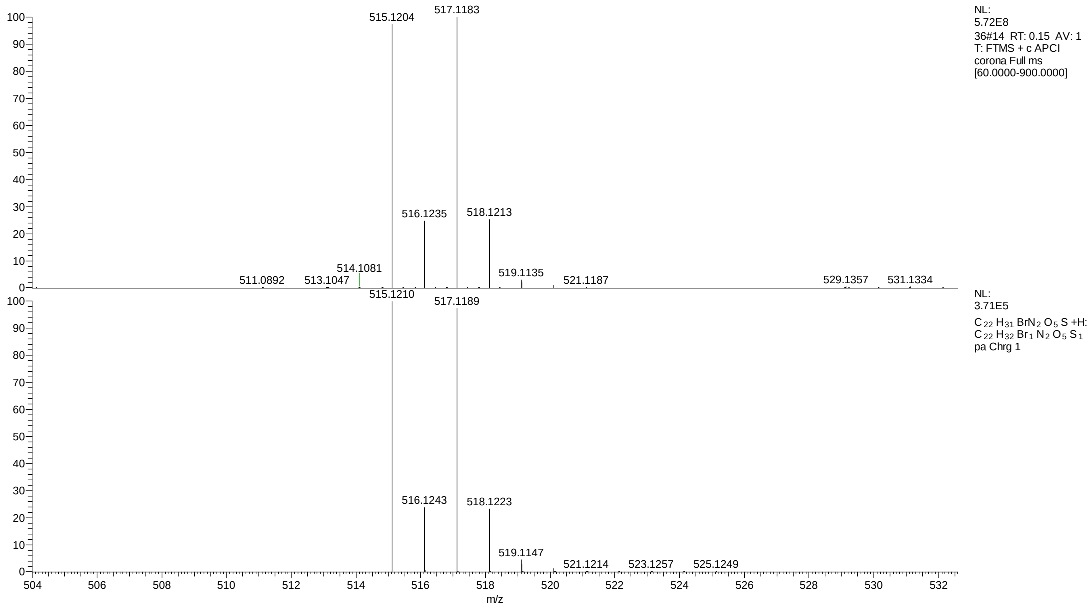

Figure S66. HRMS spectrum of **2d**.

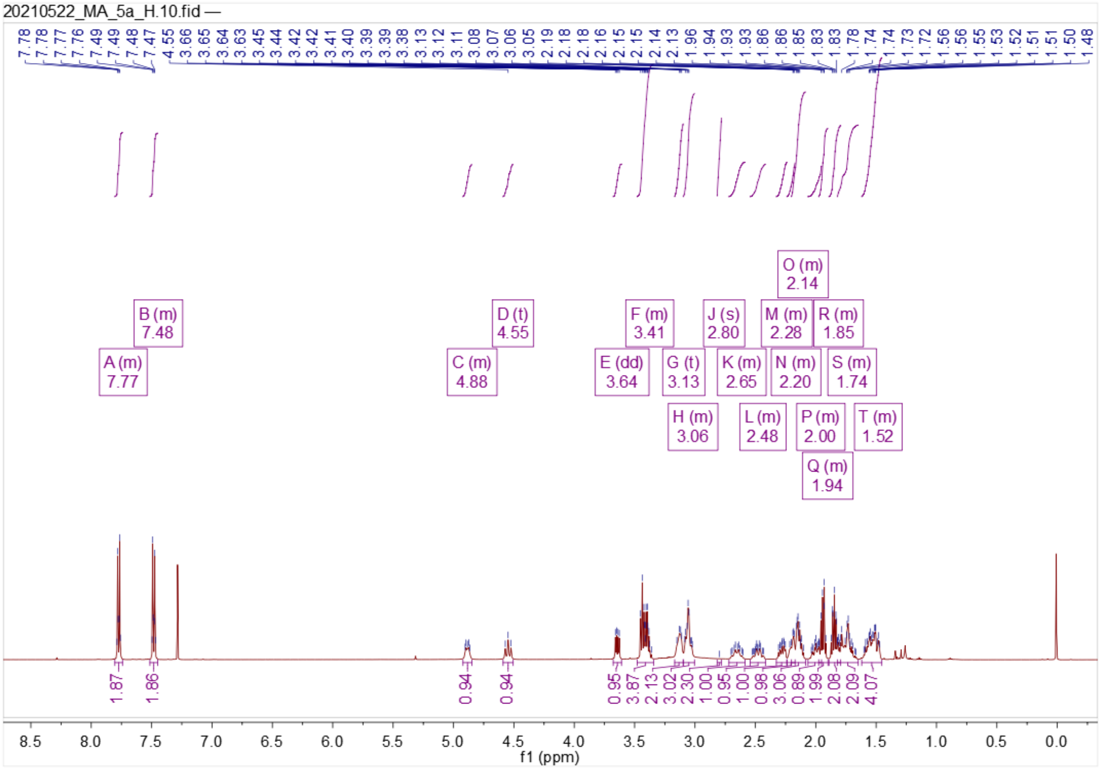

Figure S67. ^{1}H NMR spectrum (Chloroform-*d*, 500 MHz) of **3c.**

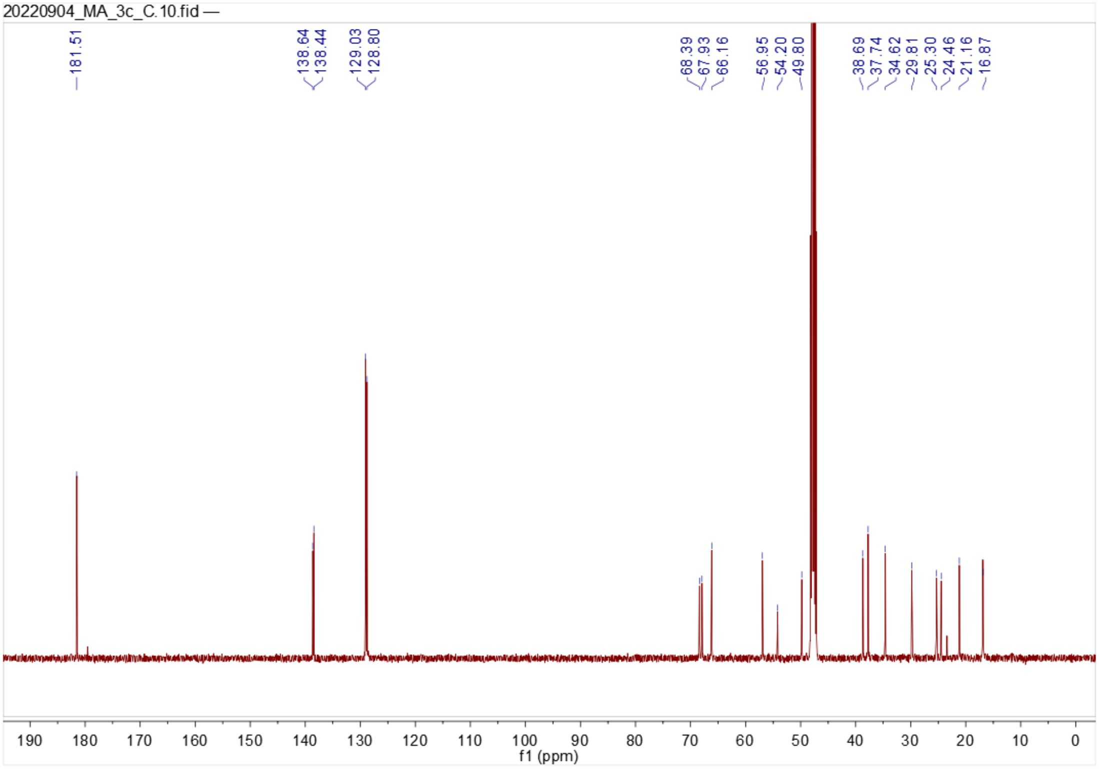

Figure S68. ^{13}C NMR spectrum (Methanol-d_4, 126 MHz) of **3c**.

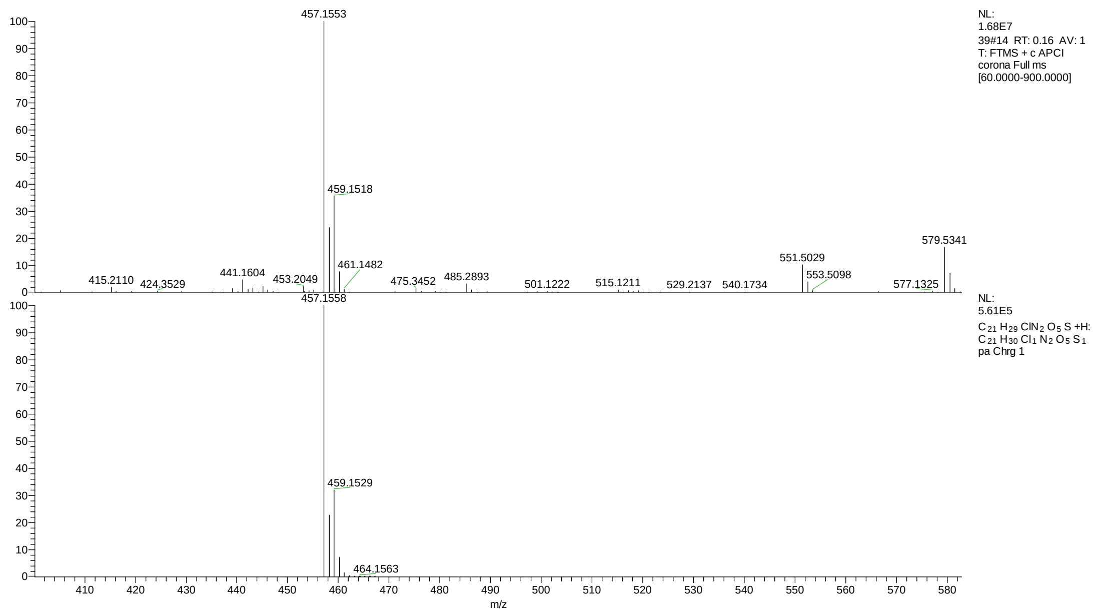

Figure S69. HRMS spectrum of **3c**.

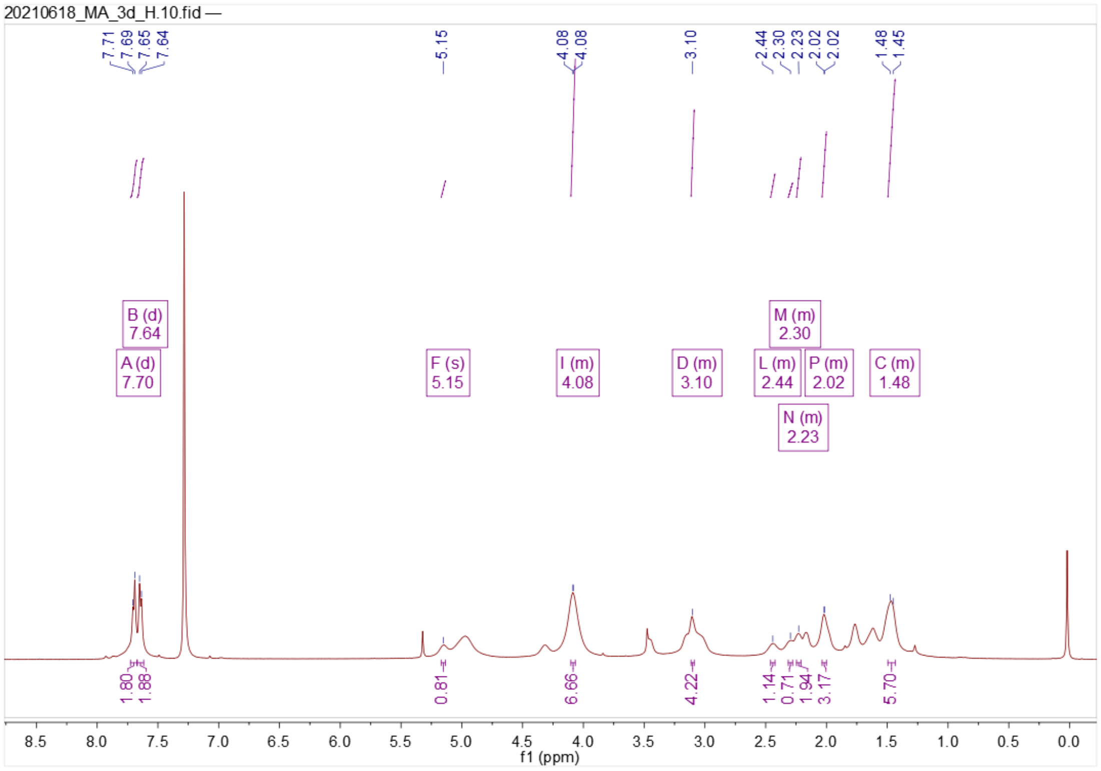

Figure S70. ^{1}H NMR spectrum (Chloroform-*d*, 500 MHz) of **3d**.

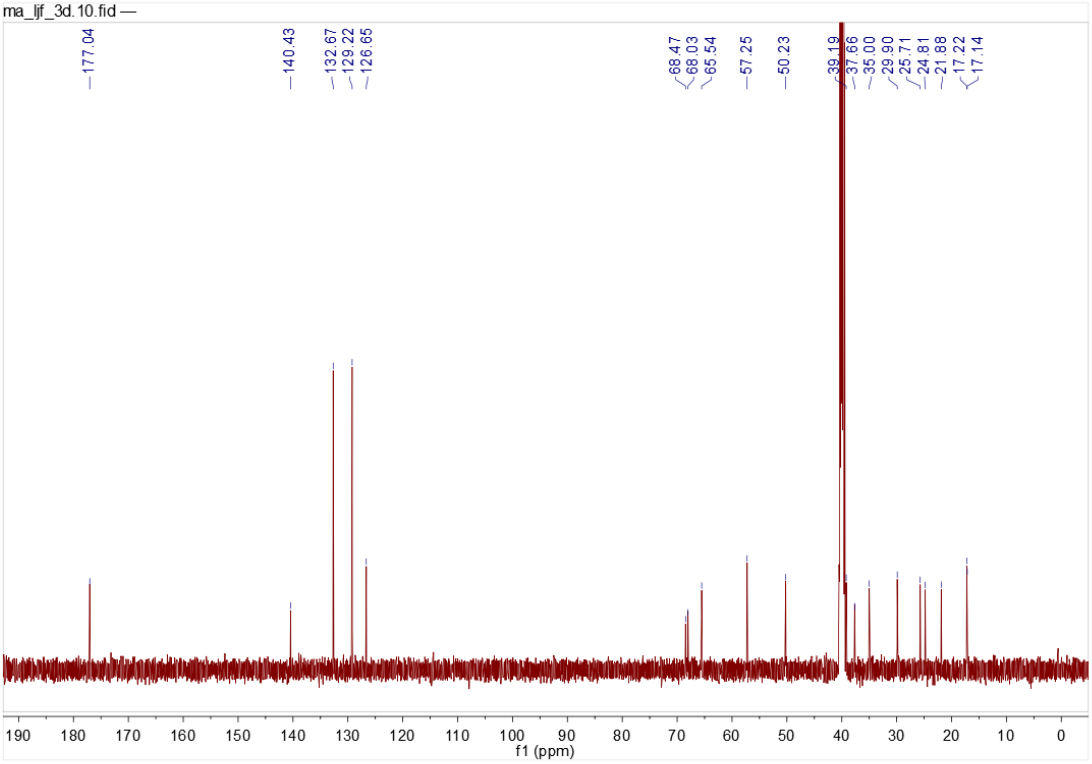

Figure S71. ^{13}C NMR spectrum (Dimethyl sulfoxide-d_6, 126 MHz) of **3d**.

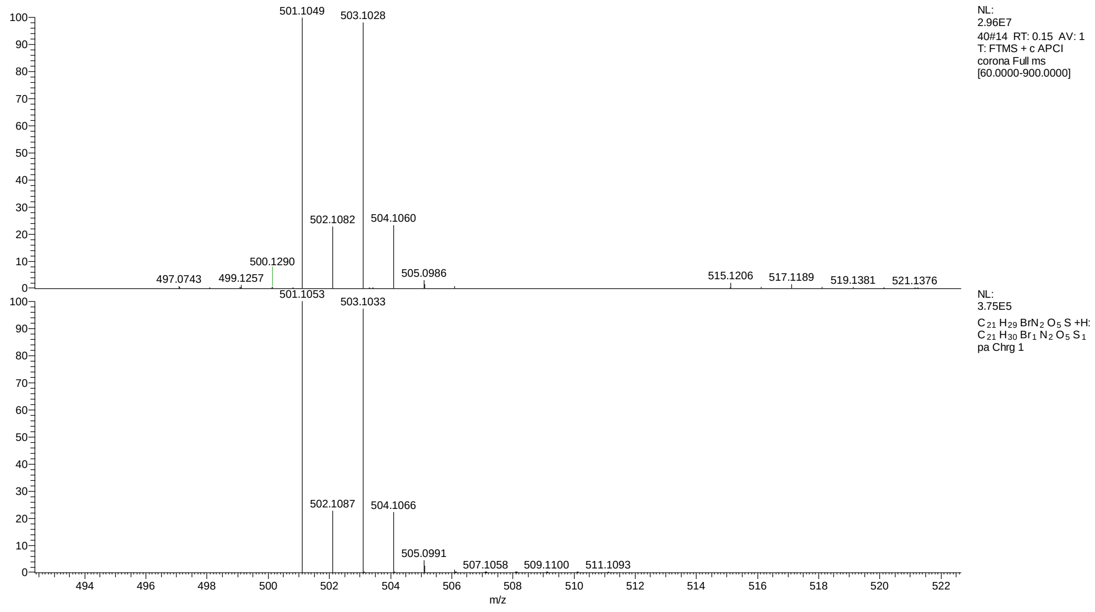

Figure S72. HRMS spectrum of **3d**.

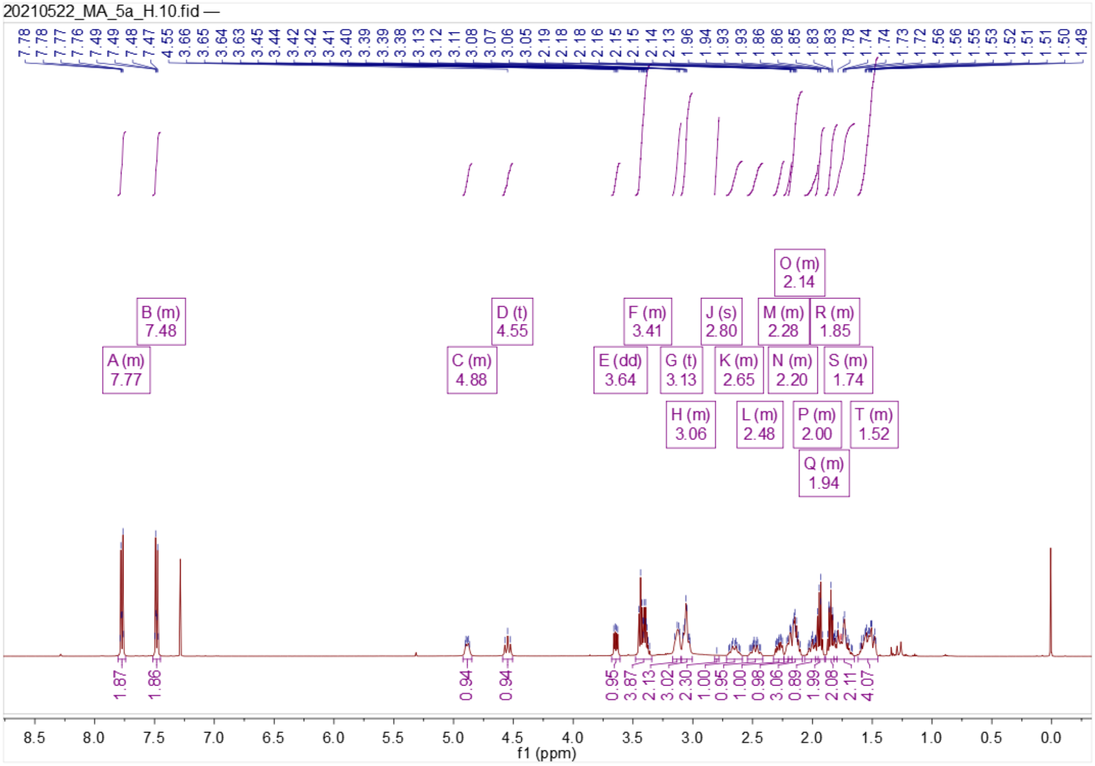

Figure S73. ^{1}H NMR spectrum (Chloroform-*d*, 500 MHz) of **5a**.

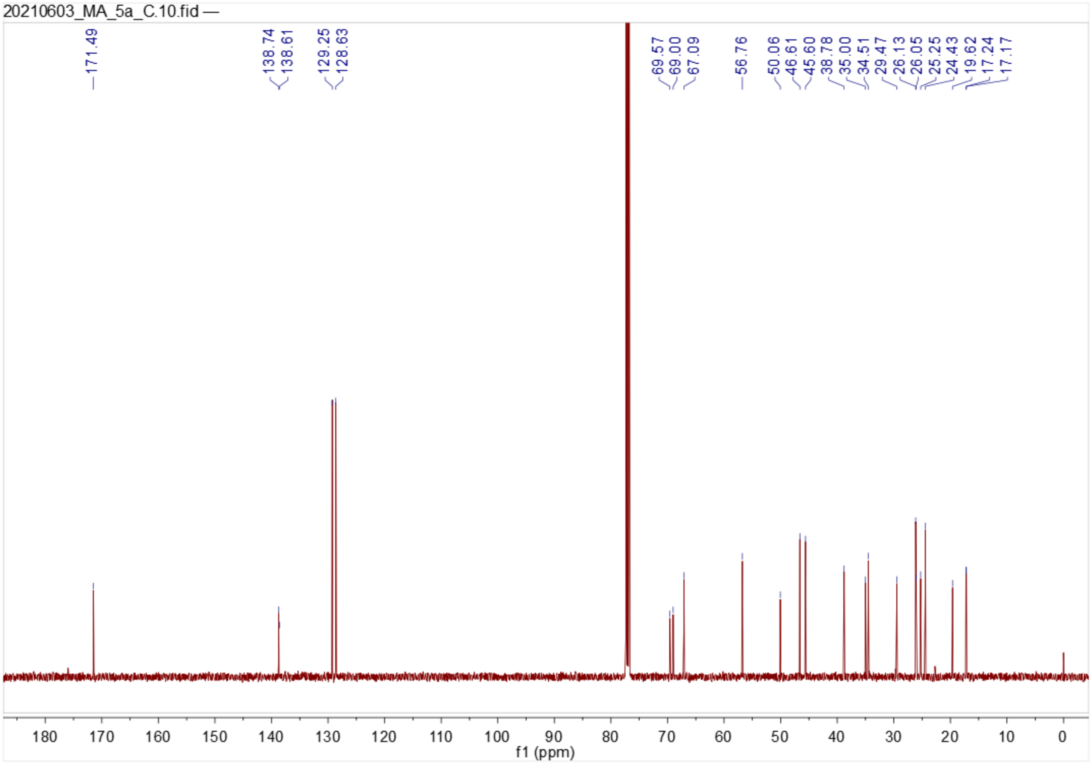

Figure S74. ^{13}C NMR spectrum (Chloroform-*d*, 126 MHz) of **5a**.

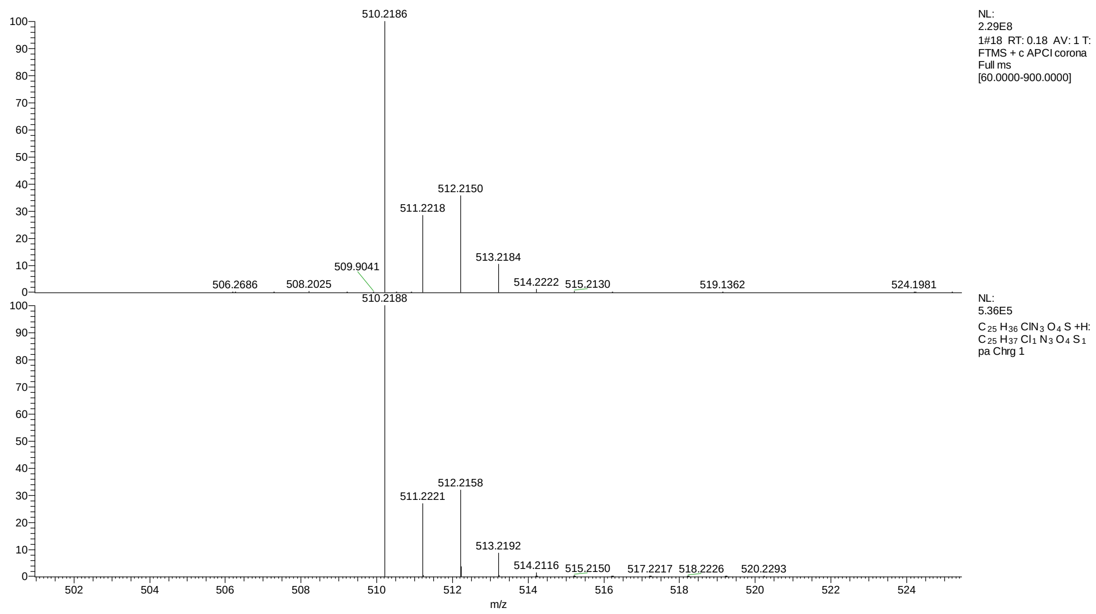

Figure S75. HRMS spectrum of **5a**.

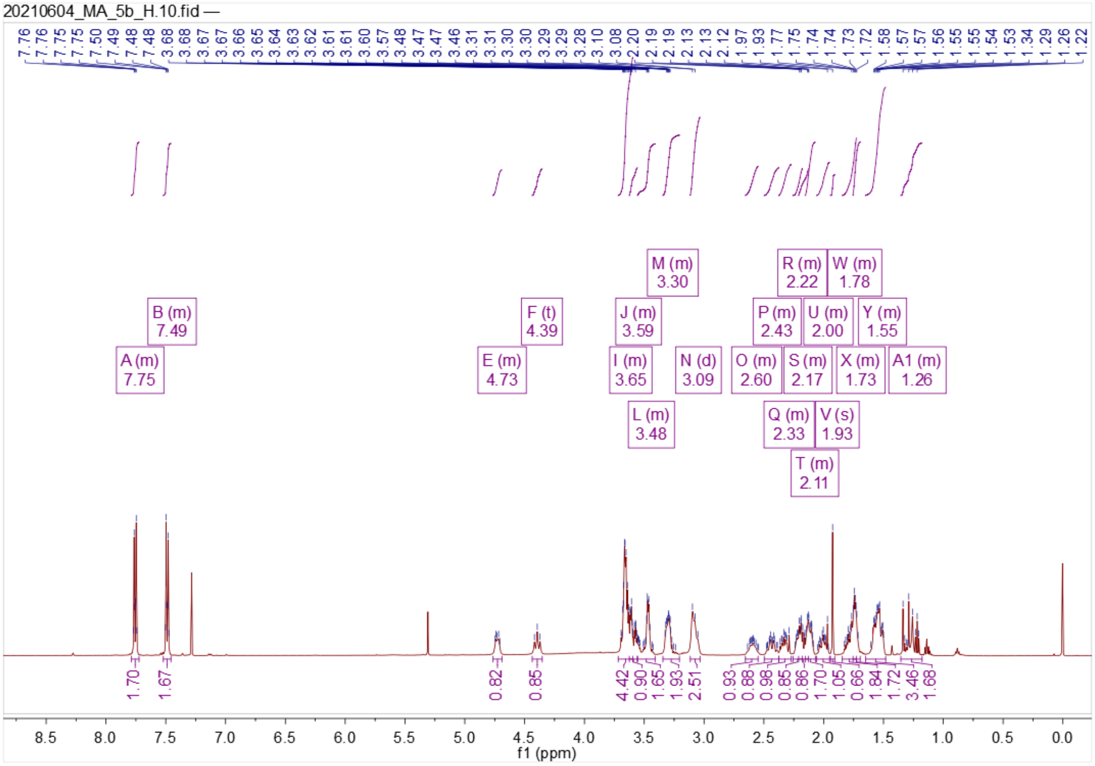

Figure S76. ^{1}H NMR spectrum (Chloroform-*d*, 500 MHz) of **5b**.

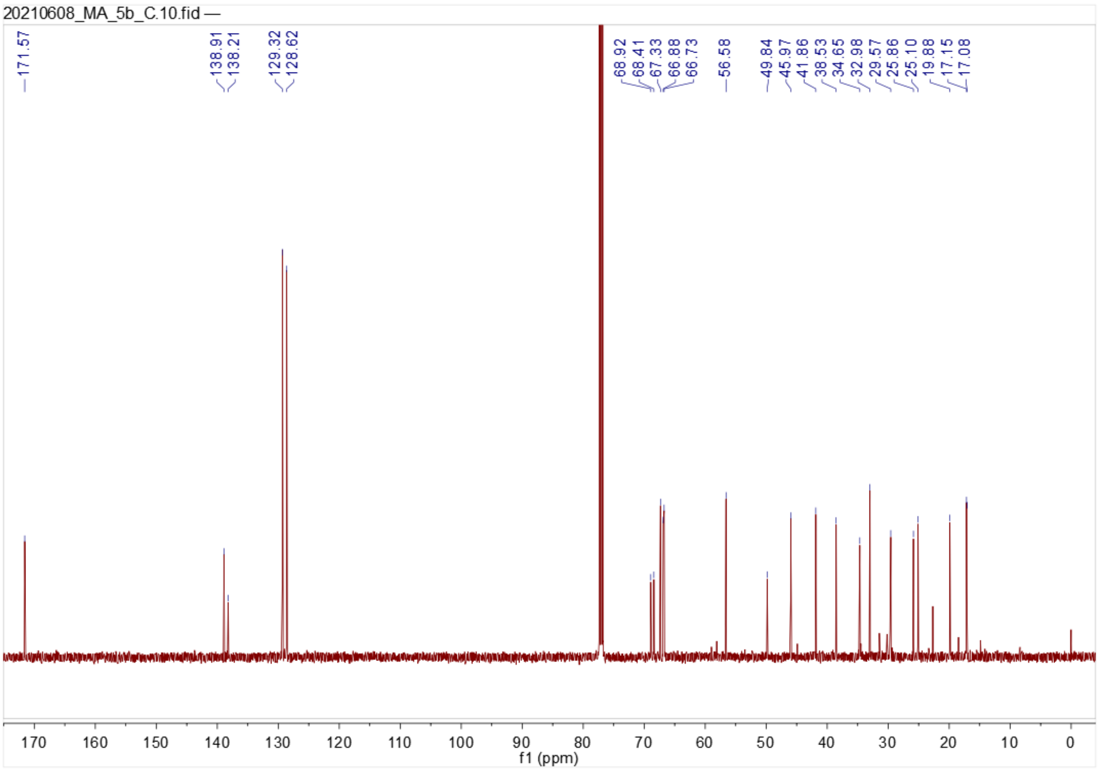

Figure S77. ^{13}C NMR spectrum (Chloroform-*d*, 126 MHz) of **5b**.

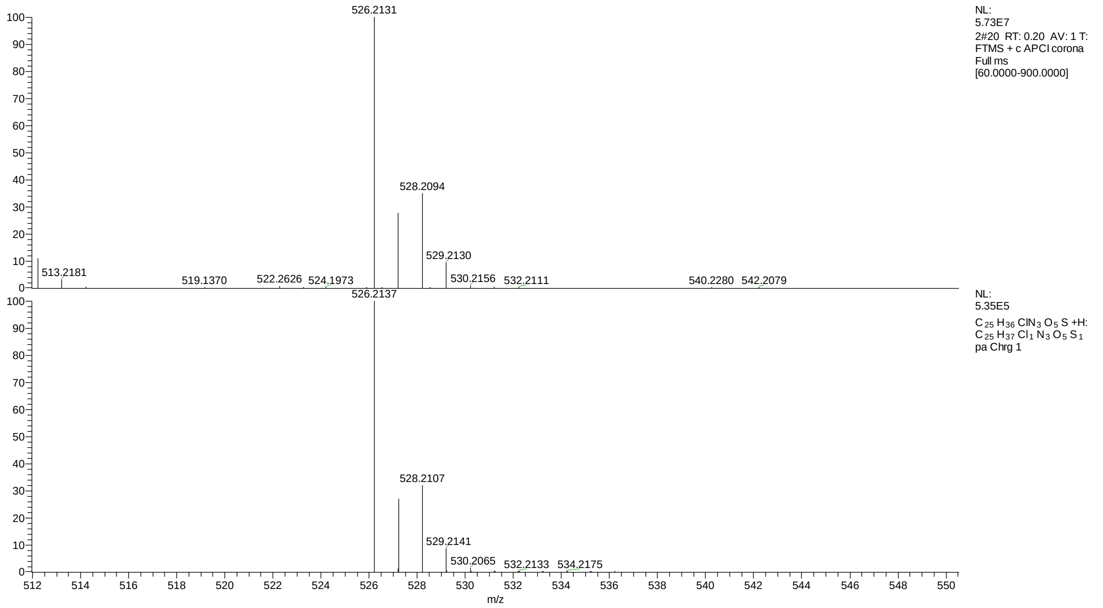

Figure S78. HRMS spectrum of **5b**.

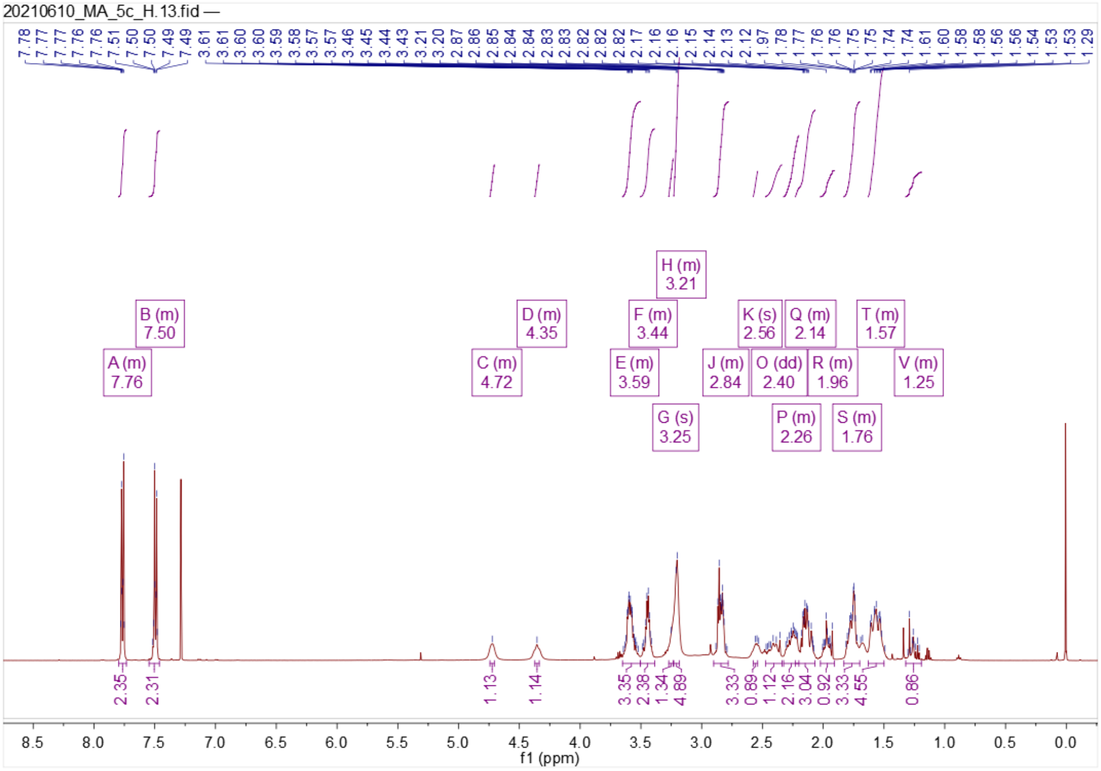

Figure S79. ^{1}H NMR spectrum (Chloroform-*d*, 500 MHz) of **5c**.

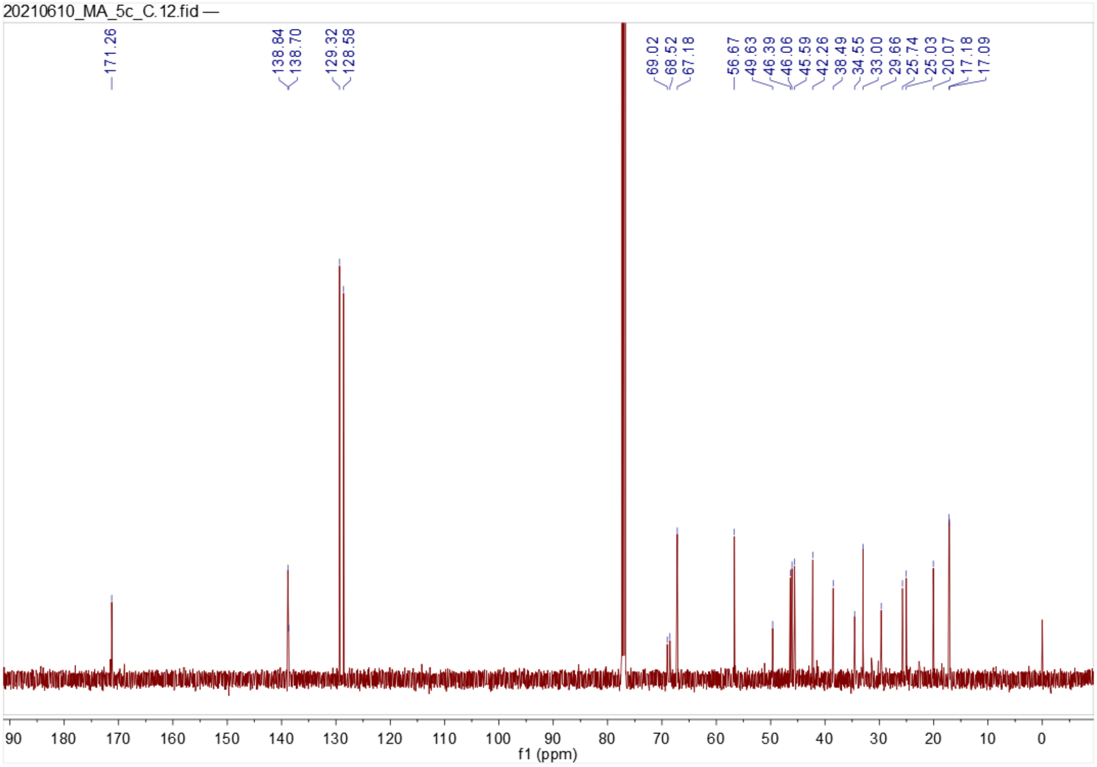

Figure S80. ^{13}C NMR spectrum (Chloroform-*d*, 126 MHz) of **5c**.

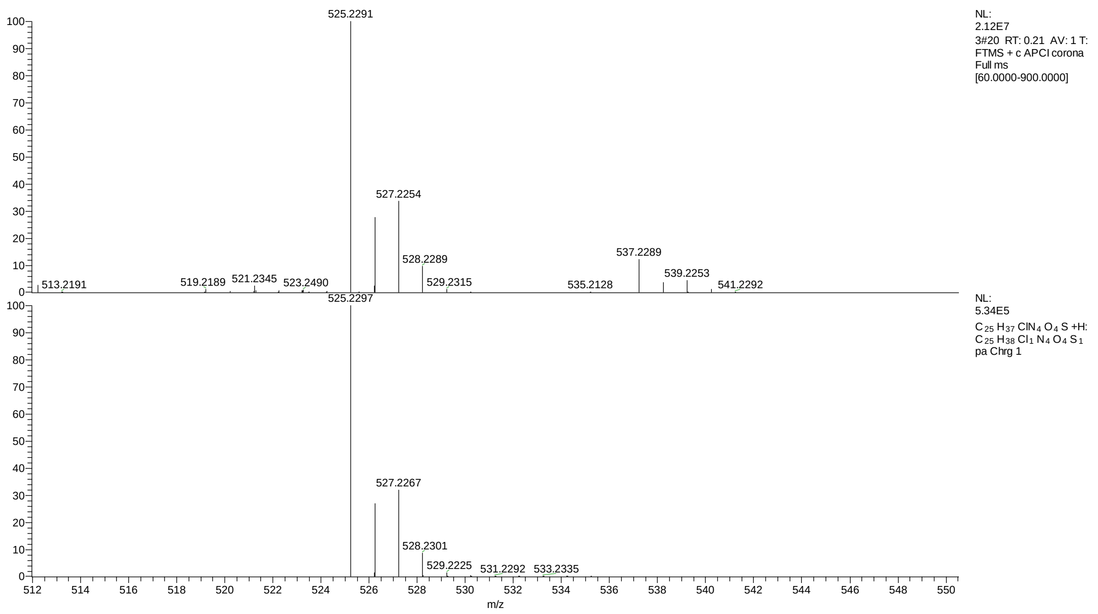

Figure S81. HRMS spectrum of **5c**.

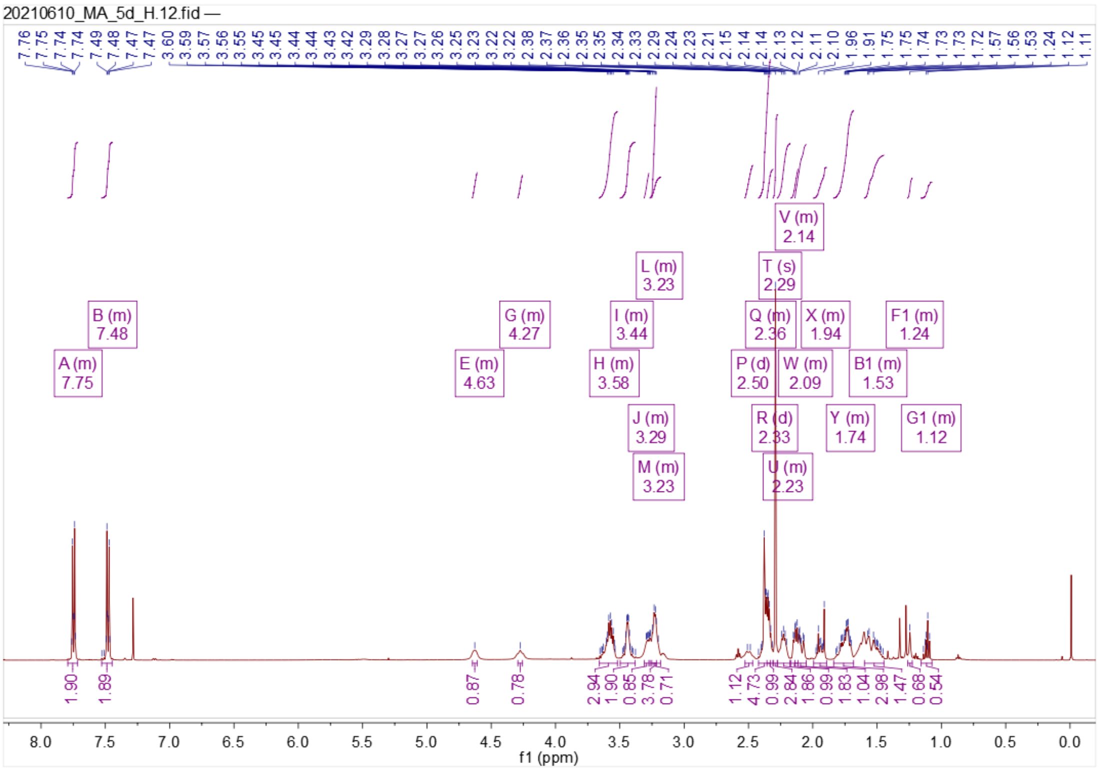

Figure S82. ^{1}H NMR spectrum (Chloroform-*d*, 500 MHz) of **5d**.

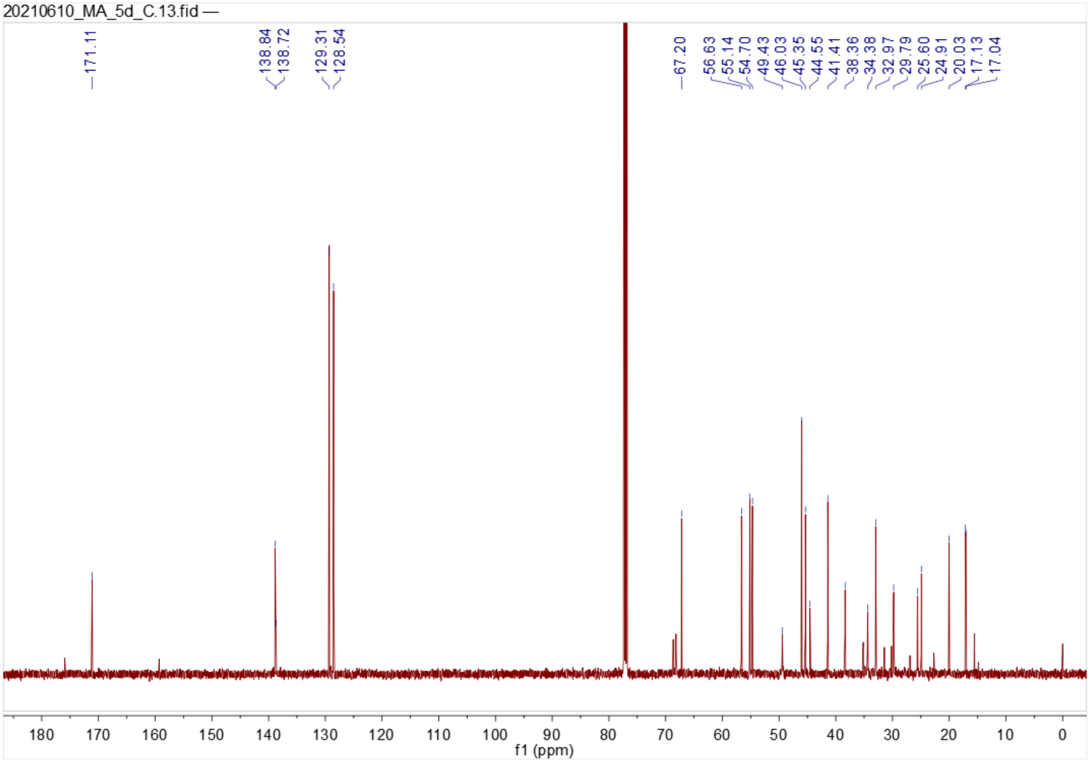

Figure S83. ^{13}C NMR spectrum (Chloroform-*d*, 126 MHz) of **5d**.

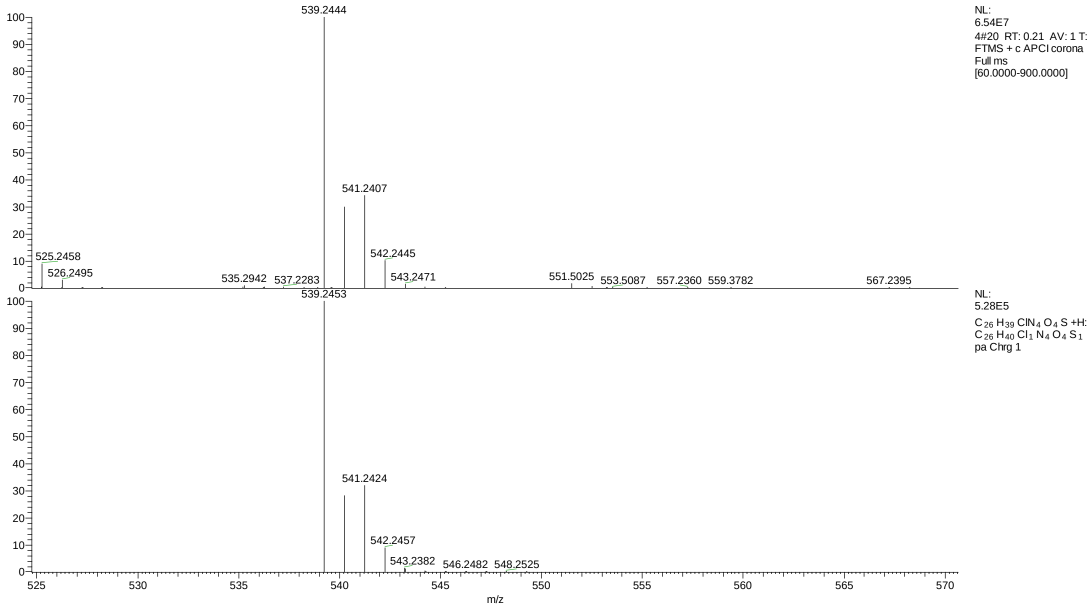

Figure S84. HRMS spectrum of **5d**.

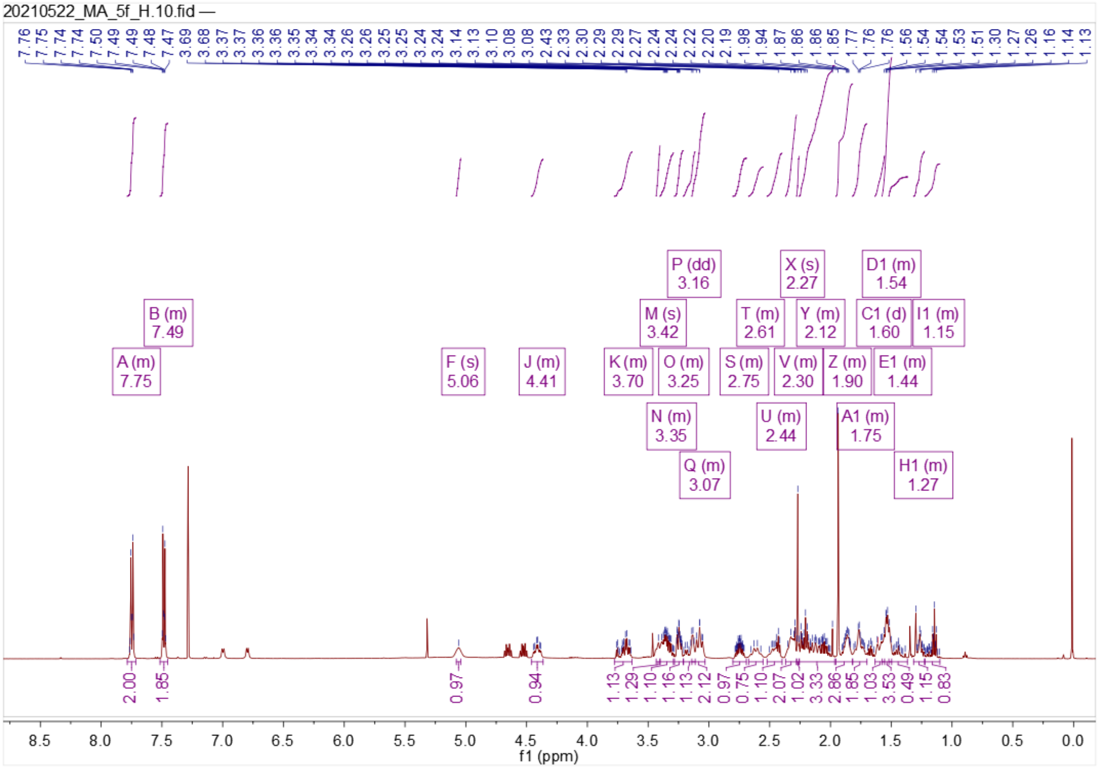

Figure S85. ^{1}H NMR spectrum (Chloroform-*d*, 500 MHz) of **5e**.

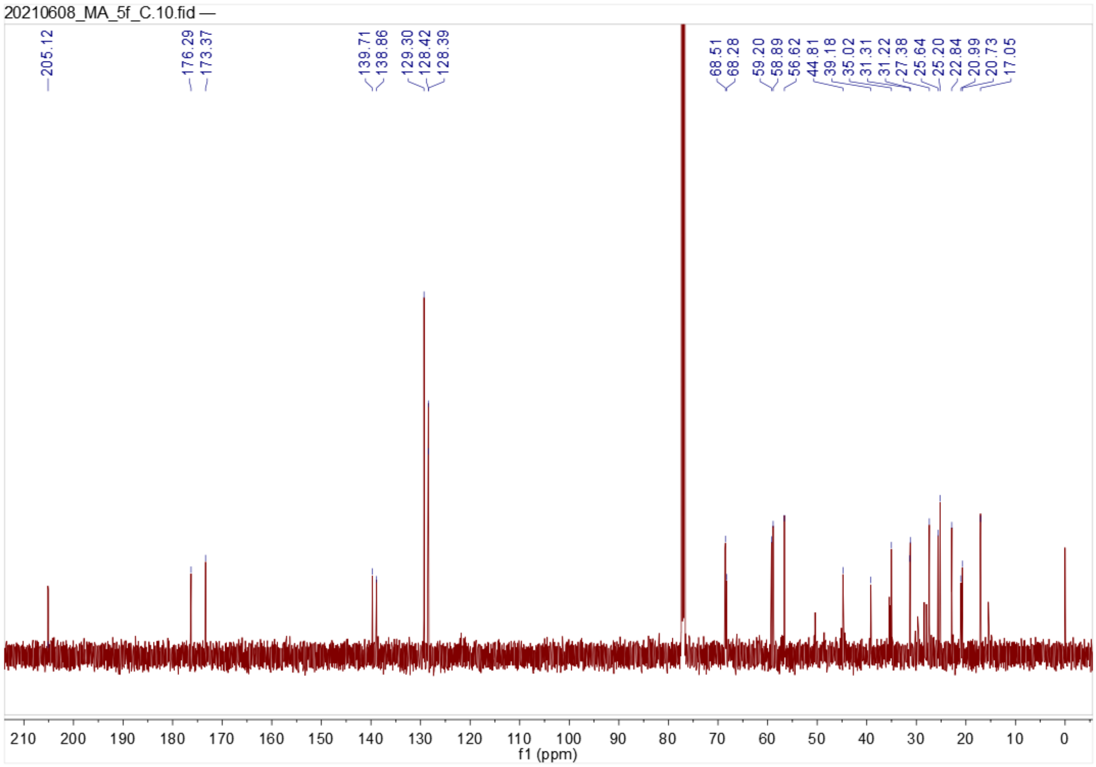

Figure S86. ^{13}C NMR spectrum (Chloroform-*d*, 126 MHz) of **5e**.

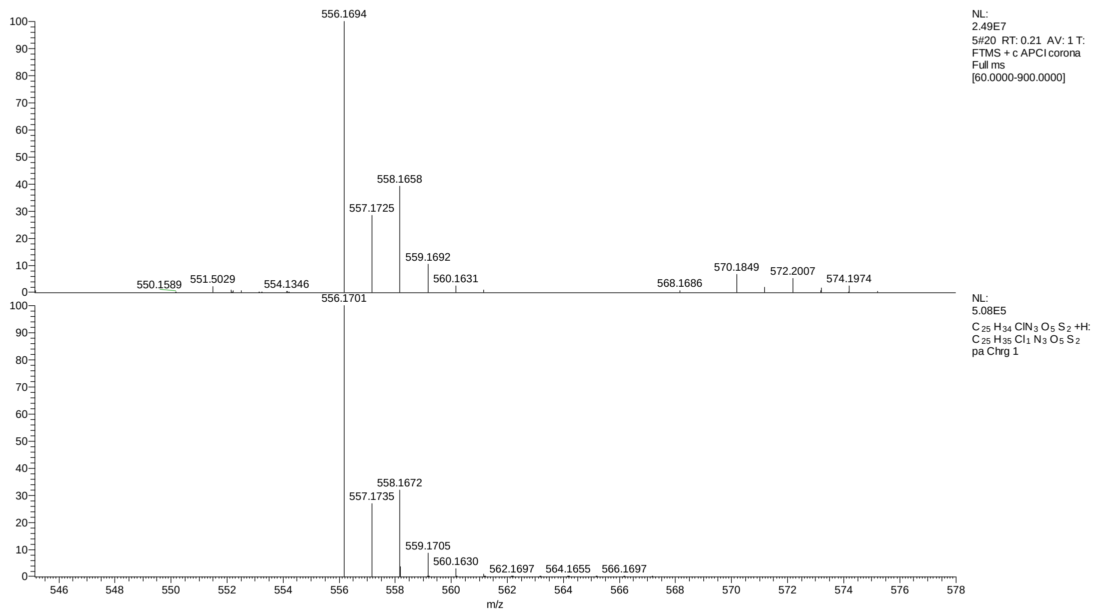

Figure S87. HRMS spectrum of **5e**.

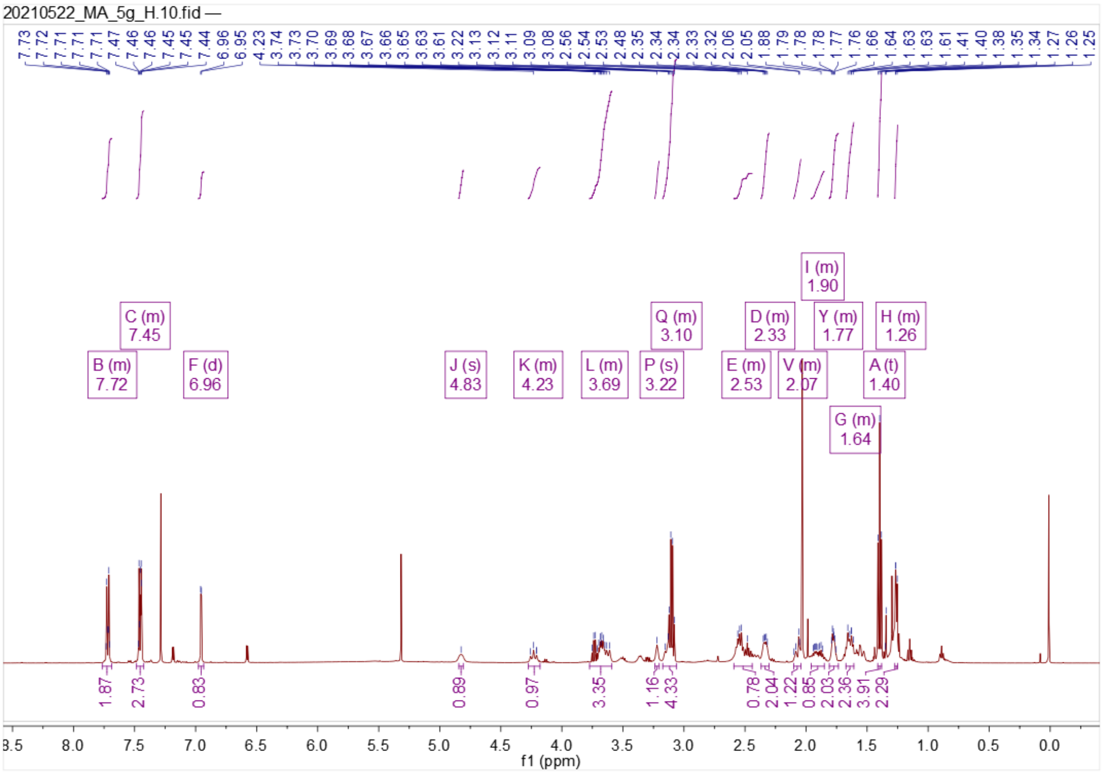

Figure S88. ^{1}H NMR spectrum (Chloroform-*d*, 500 MHz) of **5f**.

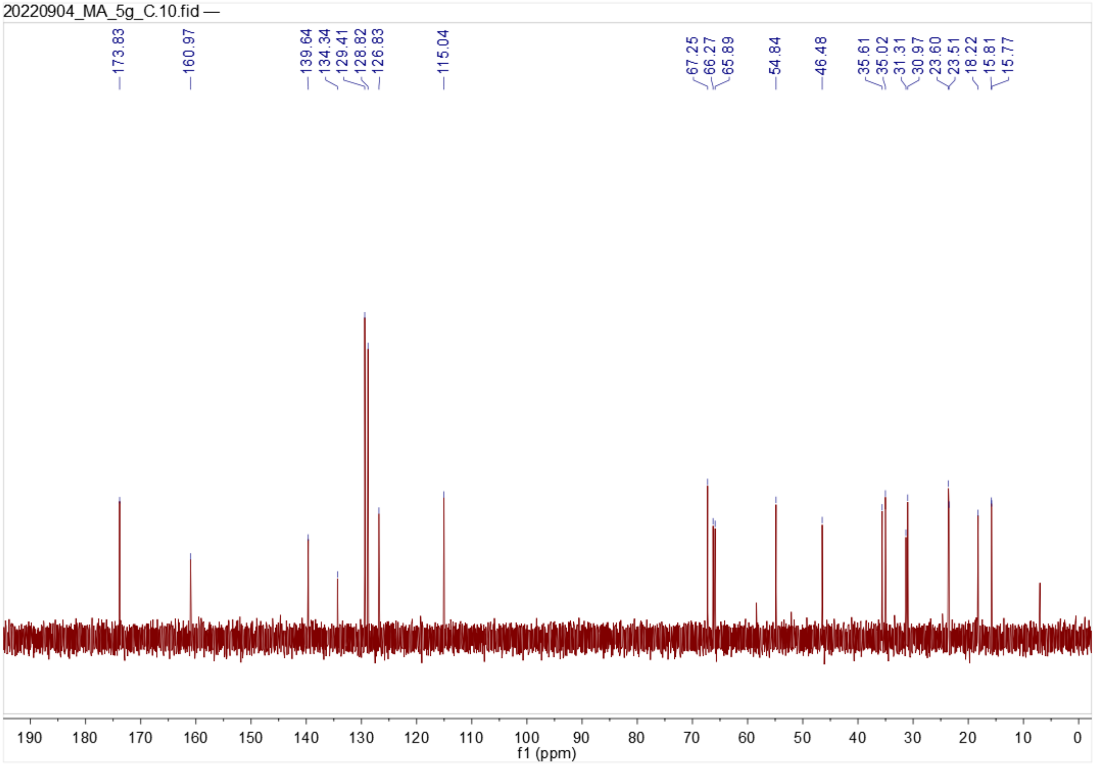

Figure S89. ^{13}C NMR spectrum (Deuterium Oxide, 126 MHz) of **5f**.

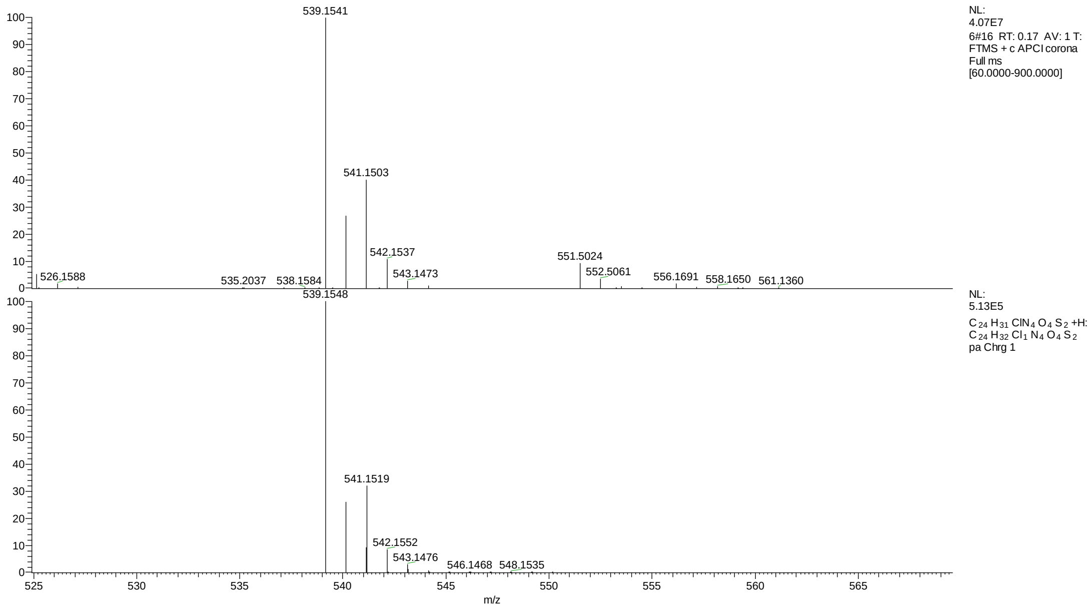

Figure S90. HRMS spectrum of **5f**.

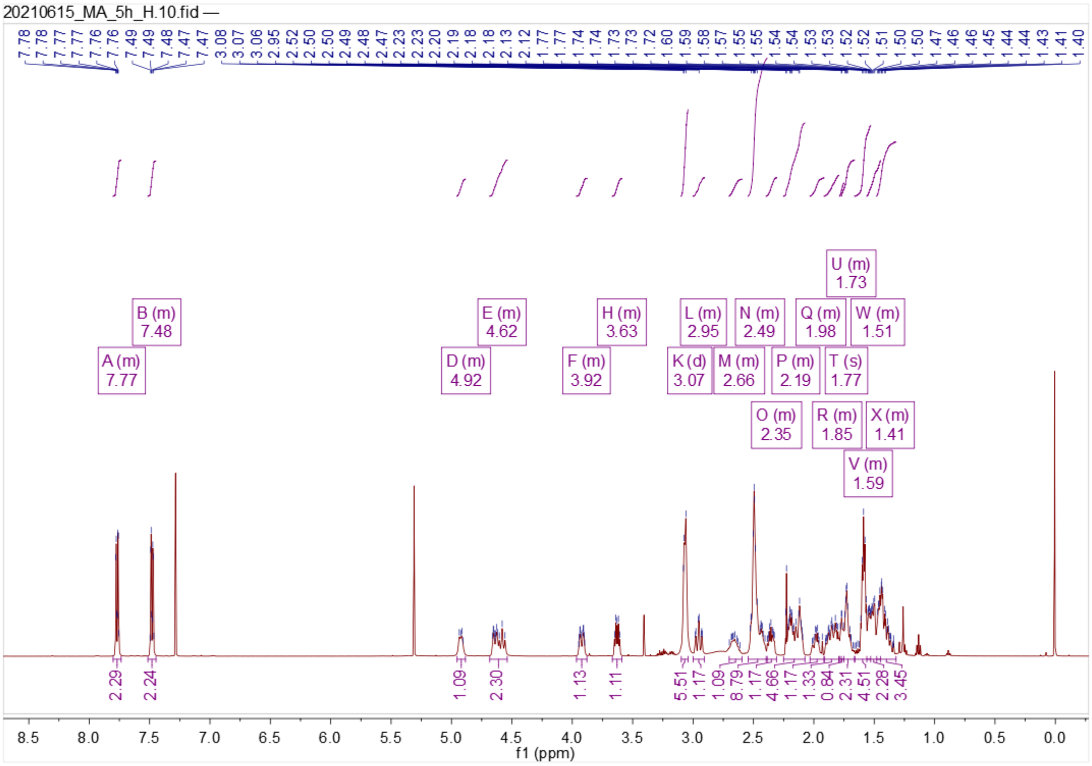

Figure S91. ^{1}H NMR spectrum (Chloroform-*d*, 500 MHz) of **5g**.

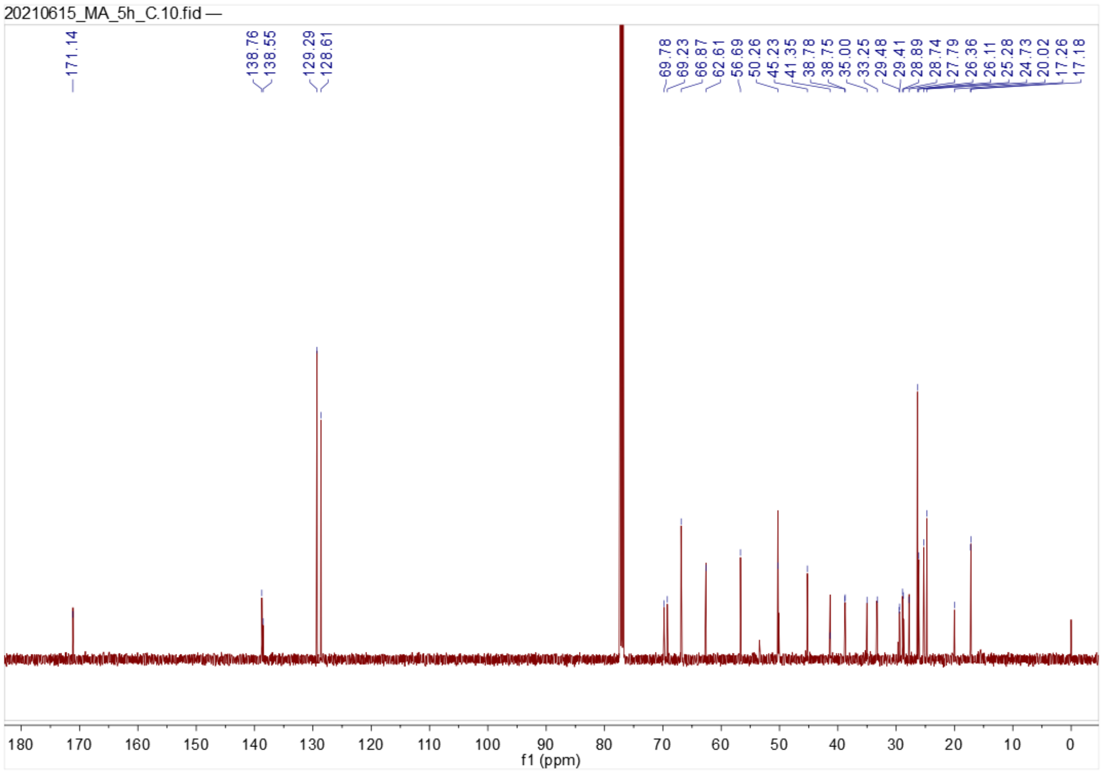

Figure S92. ^{13}C NMR spectrum (Chloroform-*d*, 126 MHz) of **5g**.

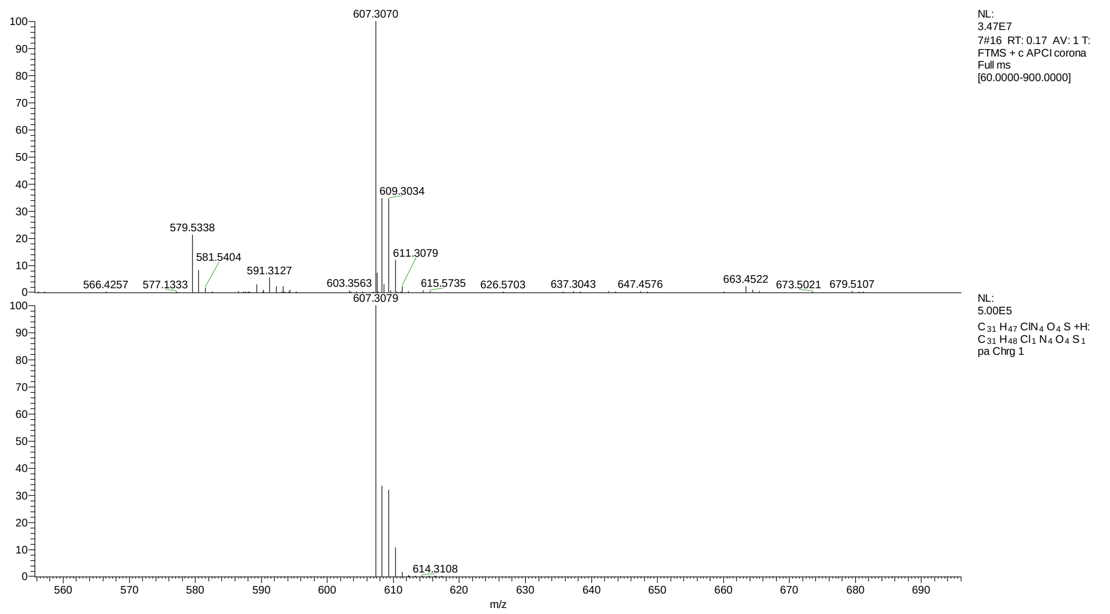

Figure S93. HRMS spectrum of **5g**.

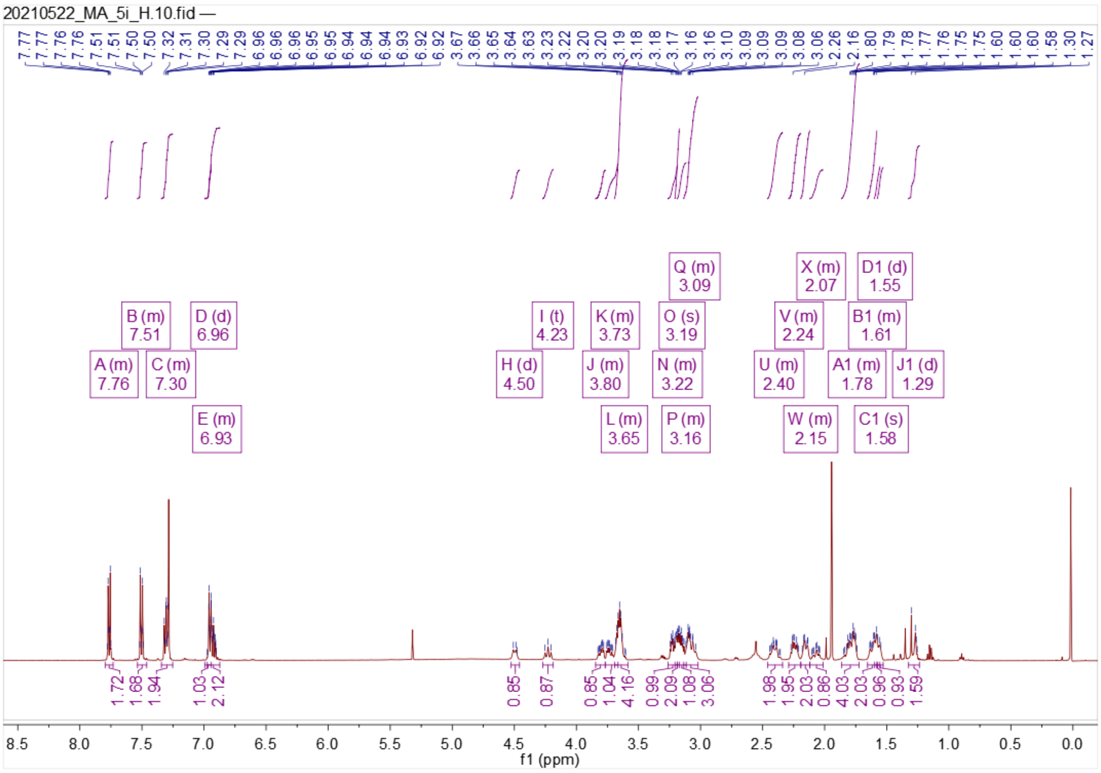

Figure S94. ^{1}H NMR spectrum (Chloroform-*d*, 500 MHz) of **5h**.

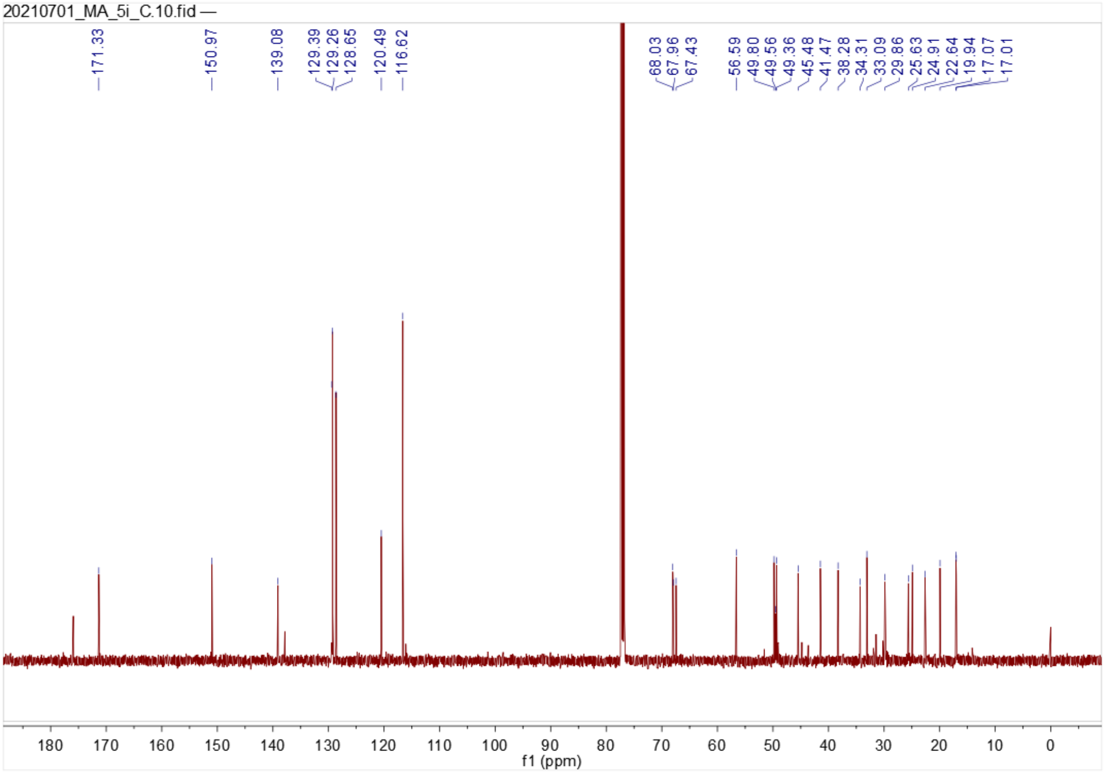

Figure S95. ^{13}C NMR spectrum (Chloroform-*d*, 126 MHz) of **5h**.

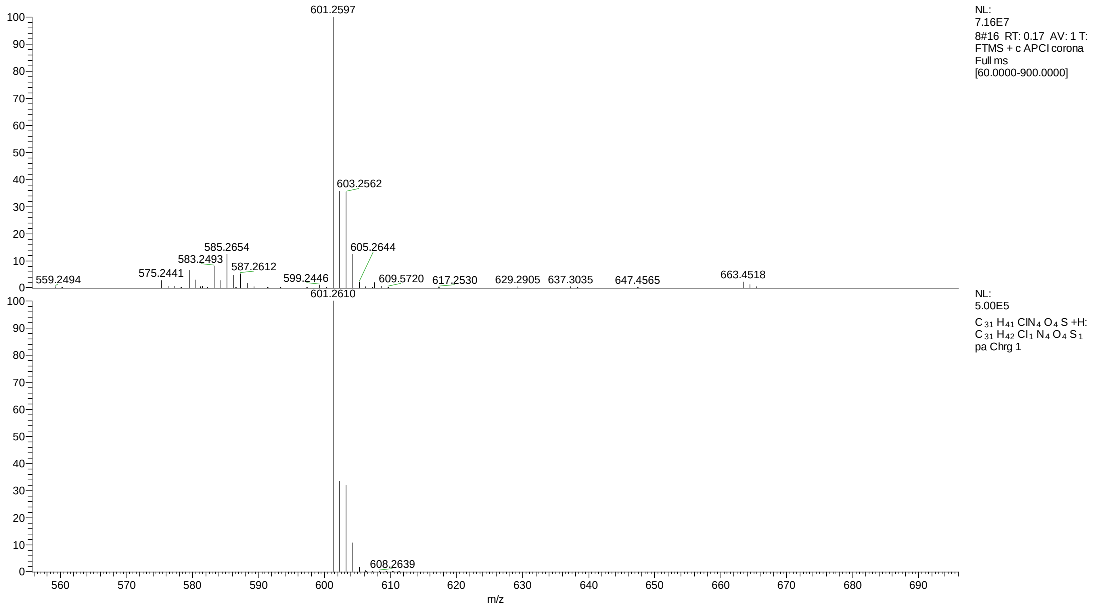

Figure S96. HRMS spectrum of **5h**.

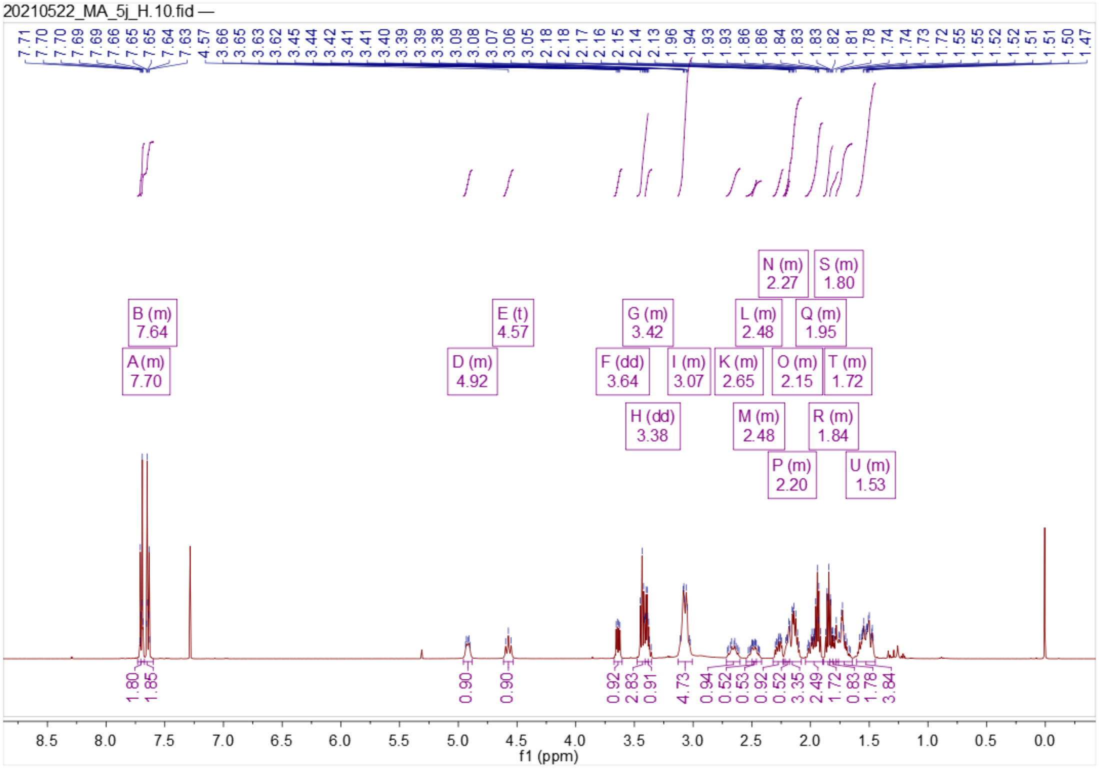

Figure S97. ^{1}H NMR spectrum (Chloroform-*d*, 500 MHz) of **5i**.

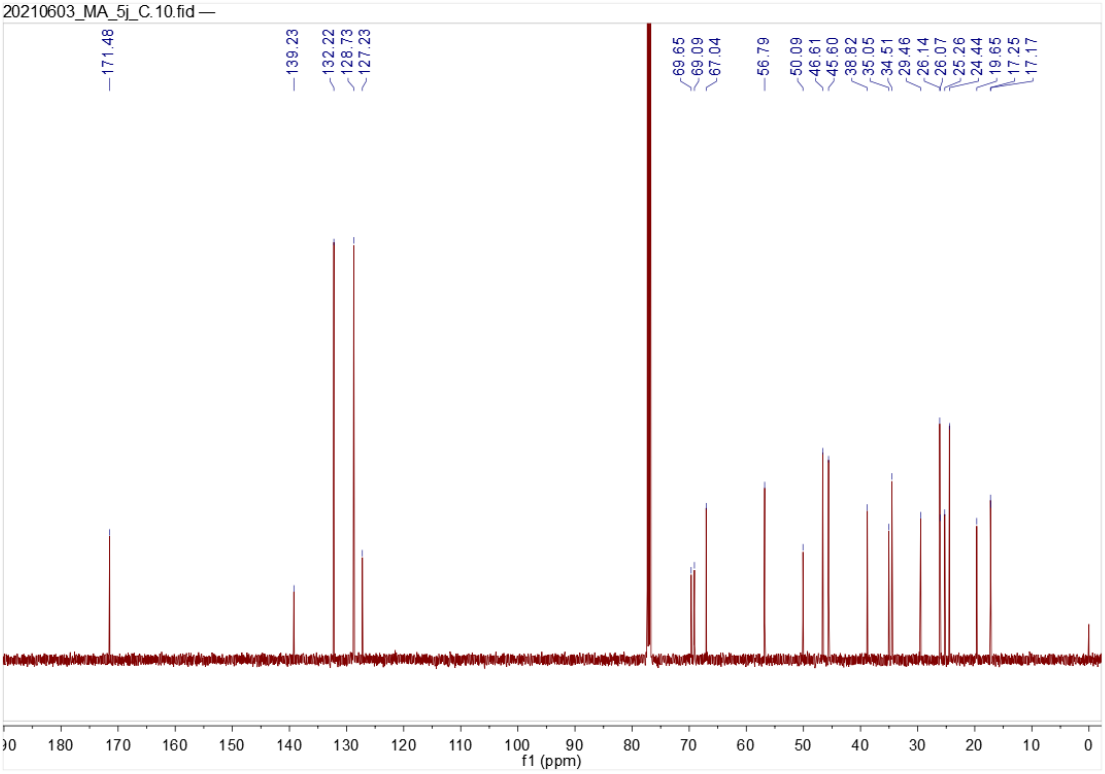

Figure S98. ^{13}C NMR spectrum (Chloroform-*d*, 126 MHz) of **5i**.

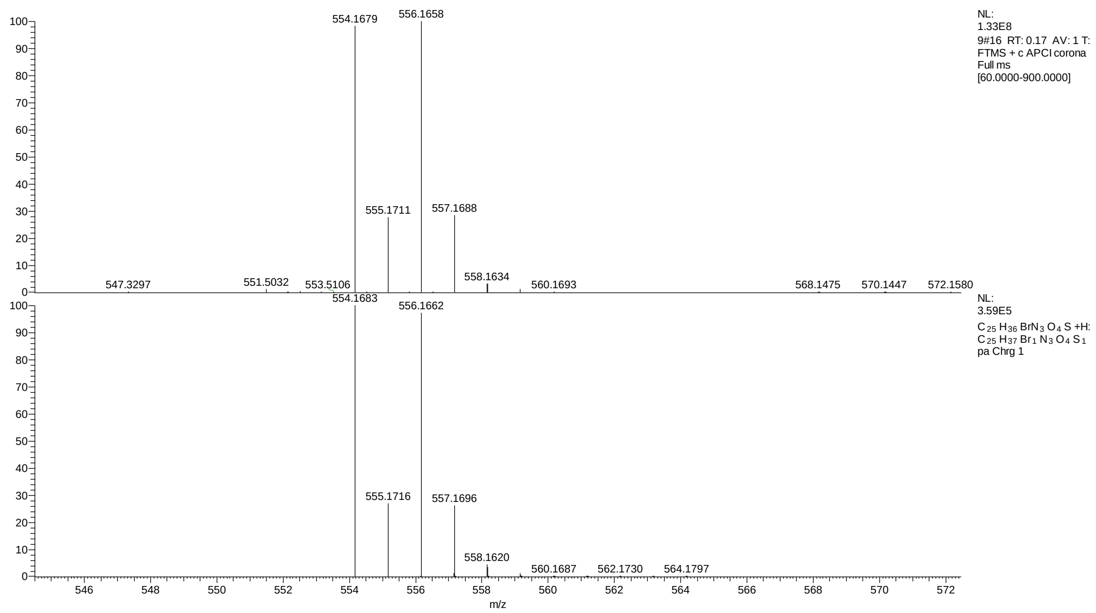

Figure S99. HRMS spectrum of **5i**.

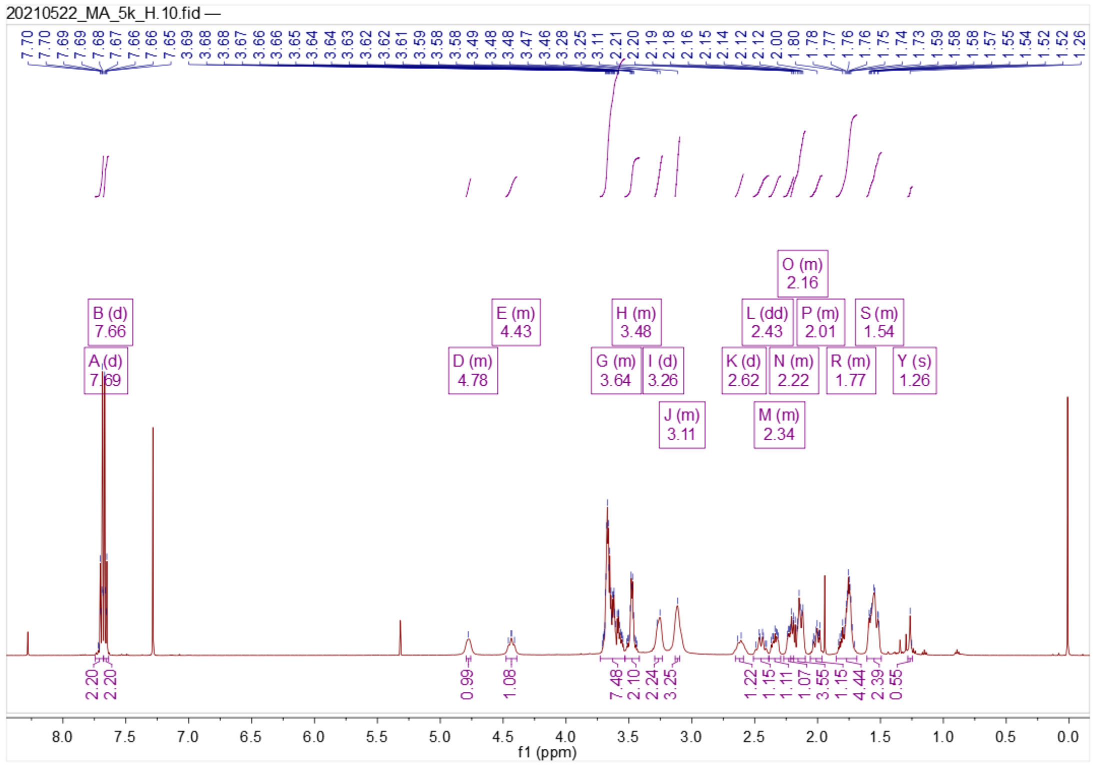

Figure S100. ^{1}H NMR spectrum (Chloroform-*d*, 500 MHz) of **5j**.

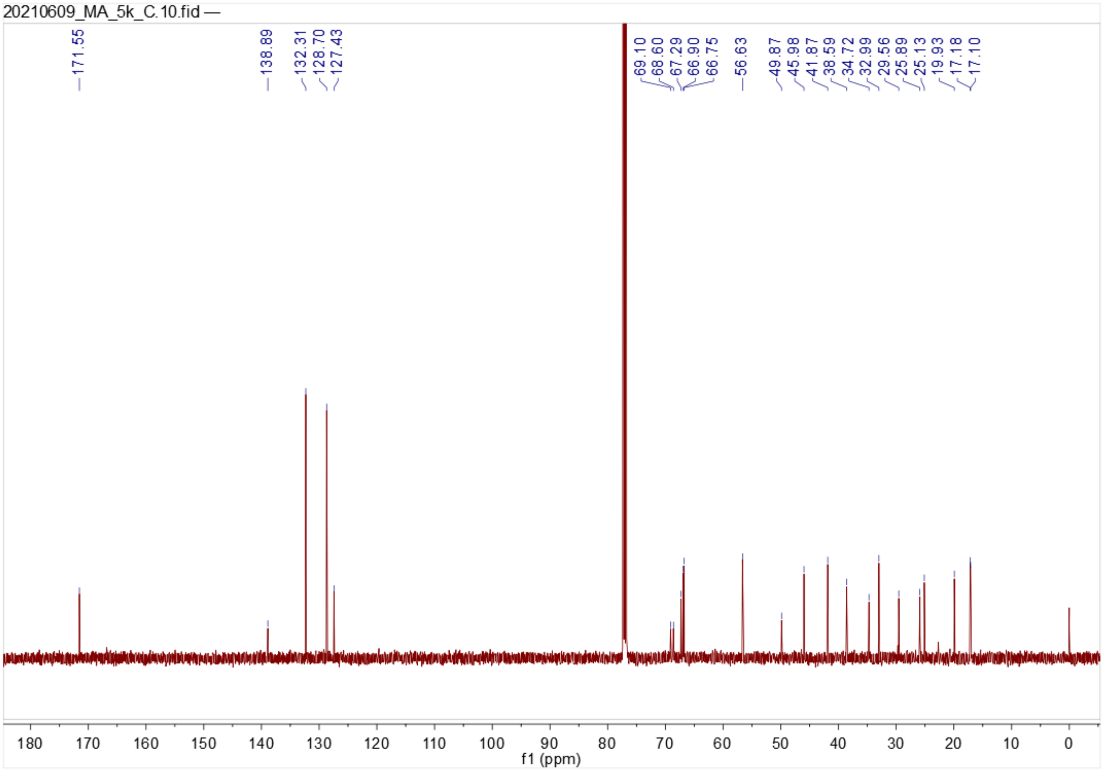

Figure S101. ^{13}C NMR spectrum (Chloroform-*d*, 126 MHz) of **5j**.

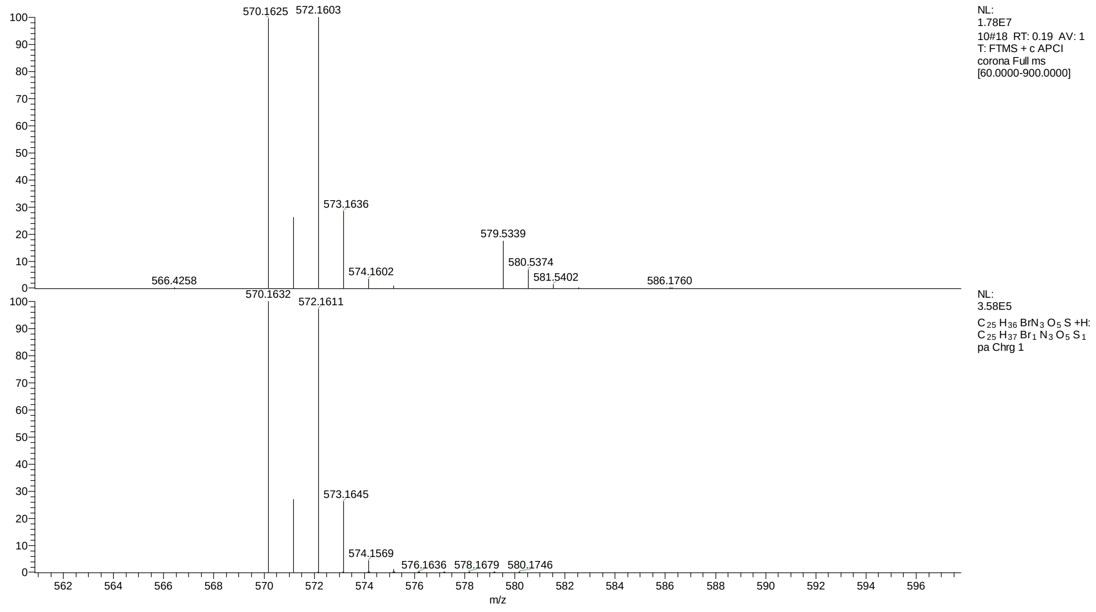

Figure S102. HRMS spectrum of **5j**.

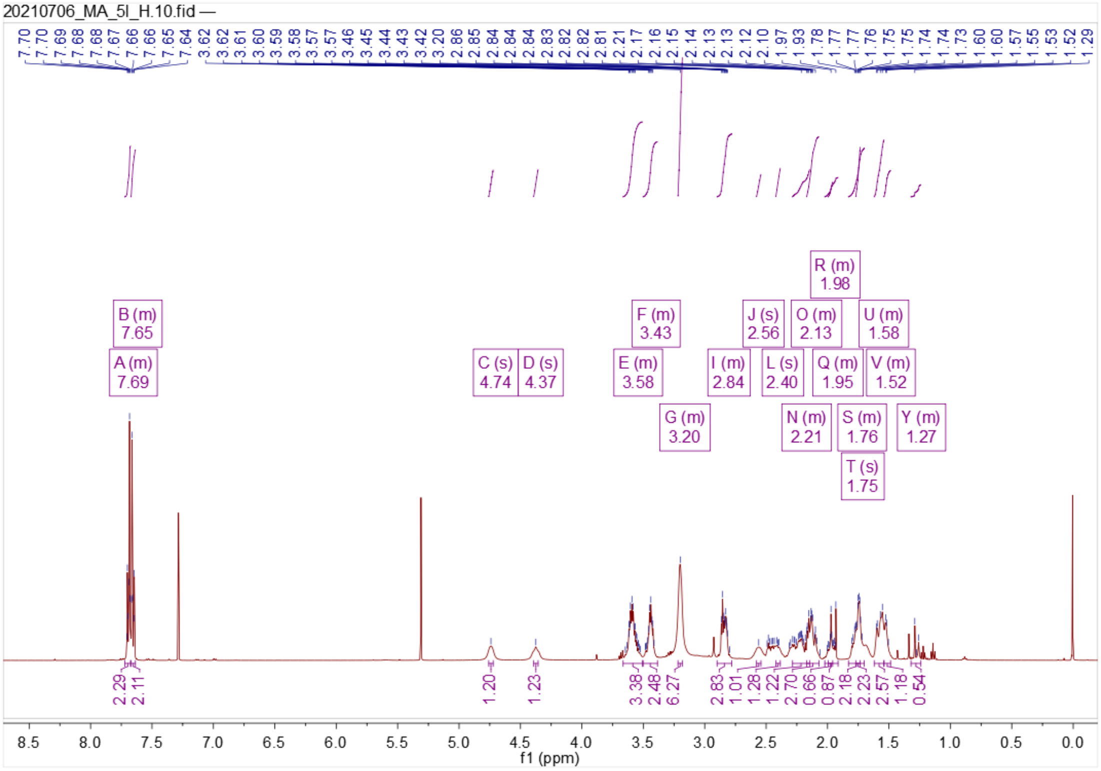

Figure S103. ^{1}H NMR spectrum (Chloroform-*d*, 500 MHz) of **5k**.

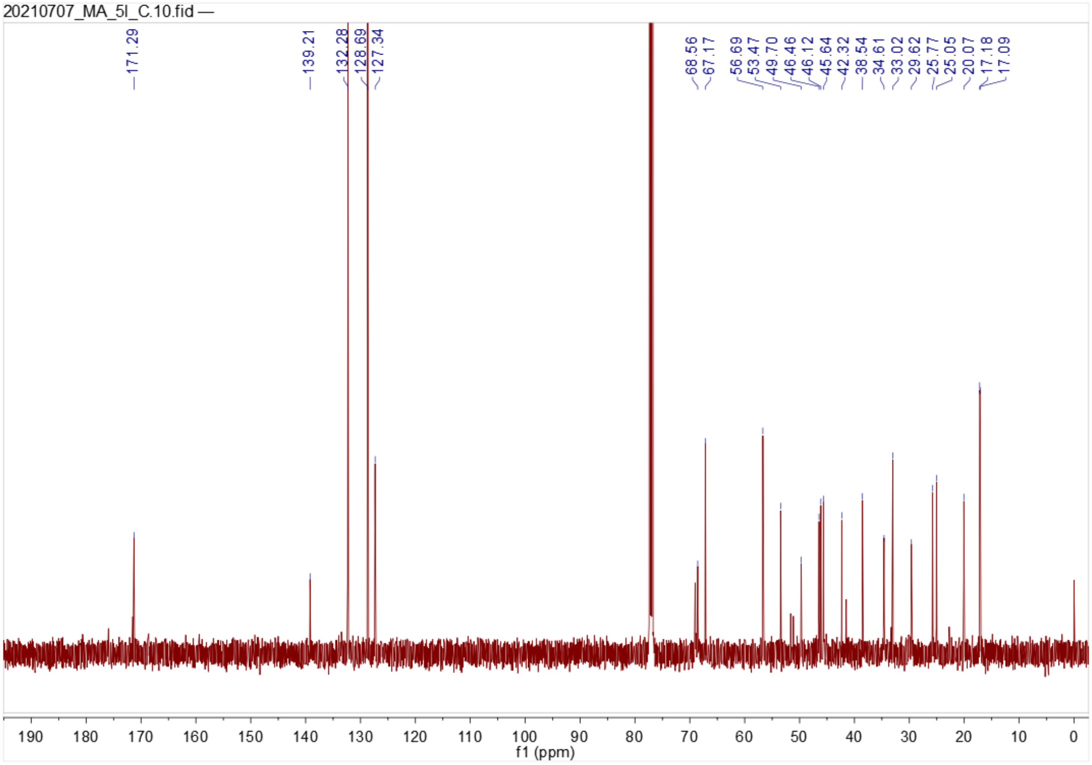

Figure S104. ^{13}C NMR spectrum (Chloroform-*d*, 126 MHz) of **5k**.

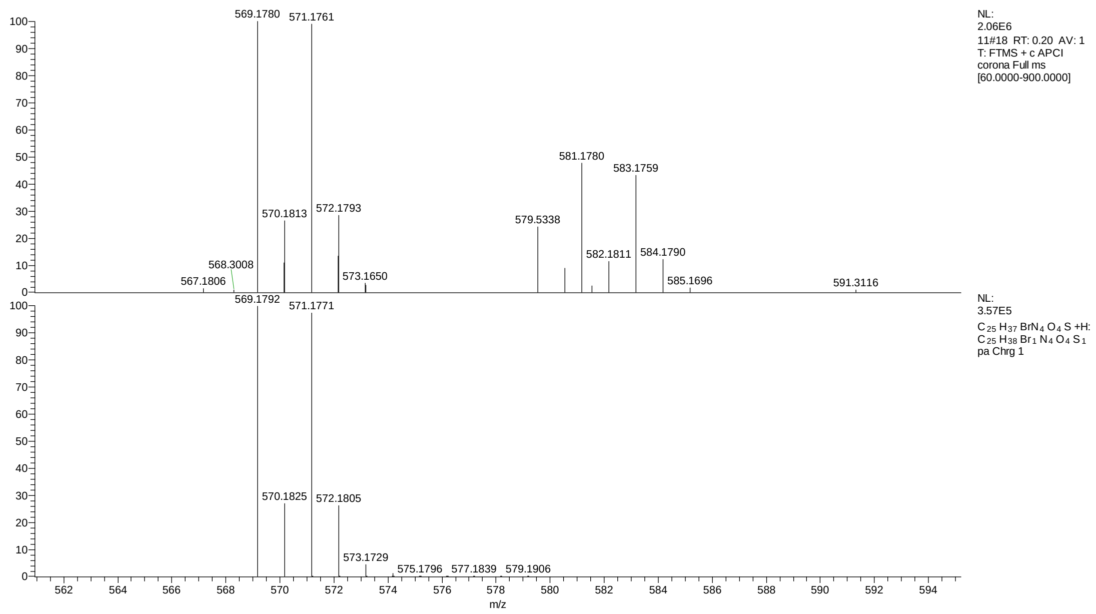

Figure S105. HRMS spectrum of **5k**.

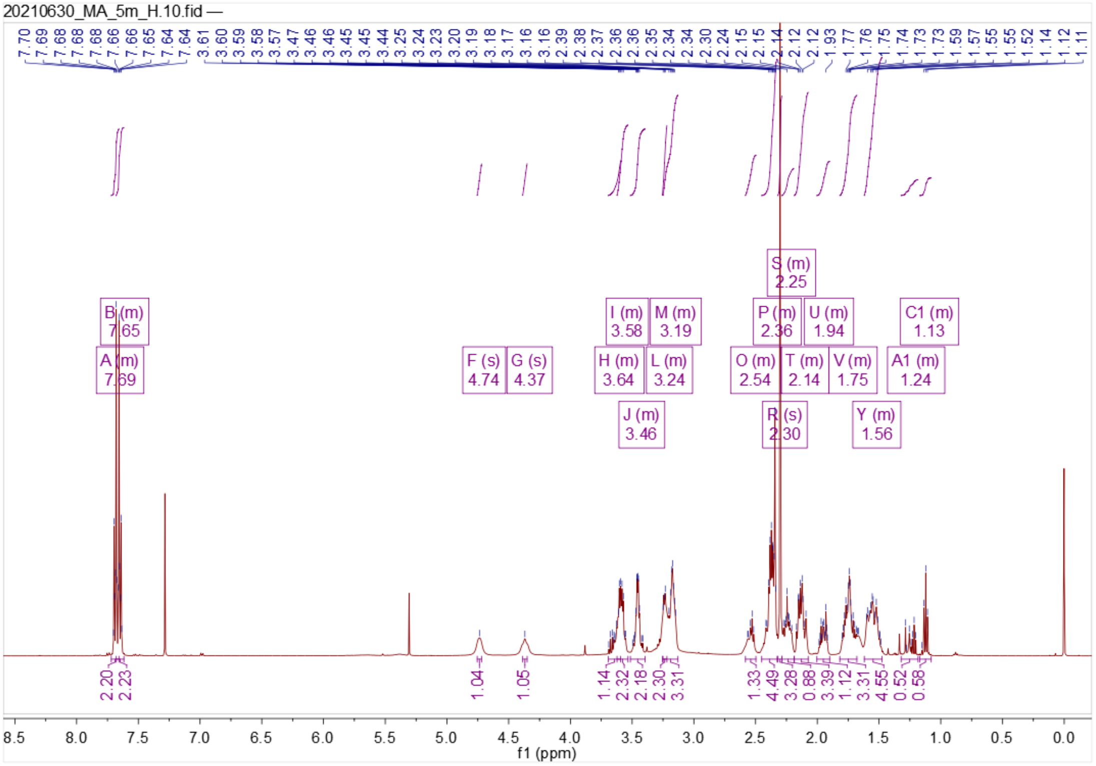

Figure S106. ^{1}H NMR spectrum (Chloroform-*d*, 500 MHz) of **5l**.

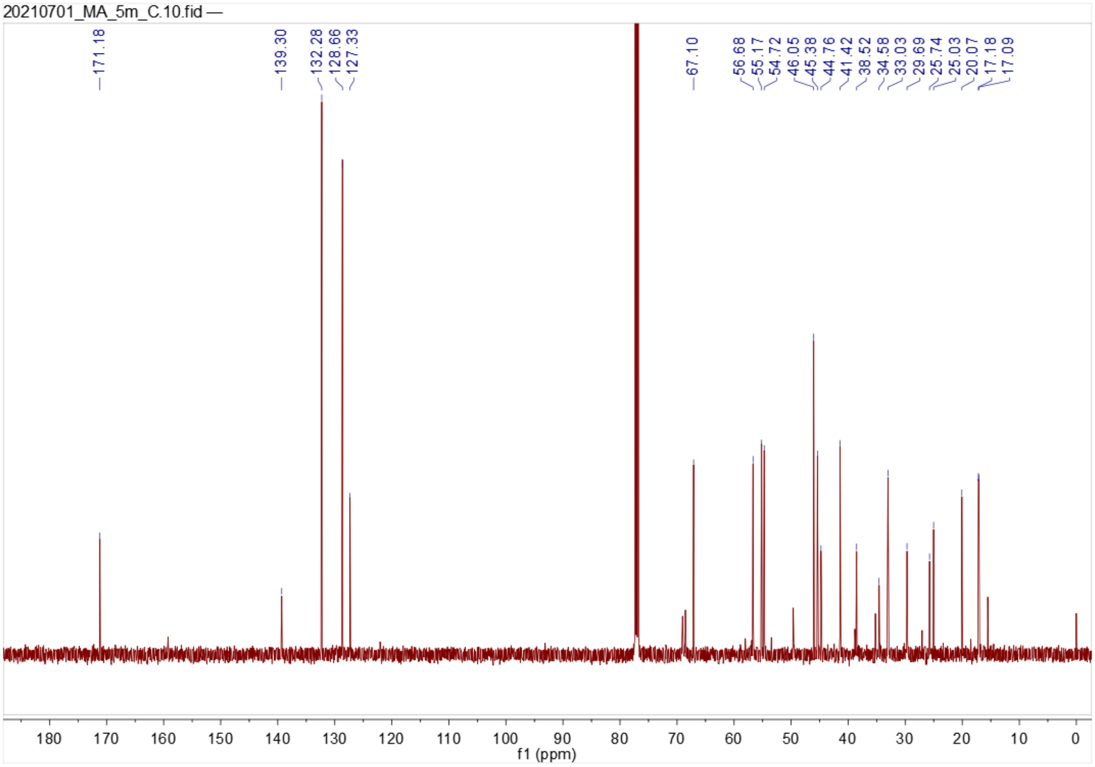

Figure S107. ^{13}C NMR spectrum (Chloroform-*d*, 126 MHz) of **5l**.

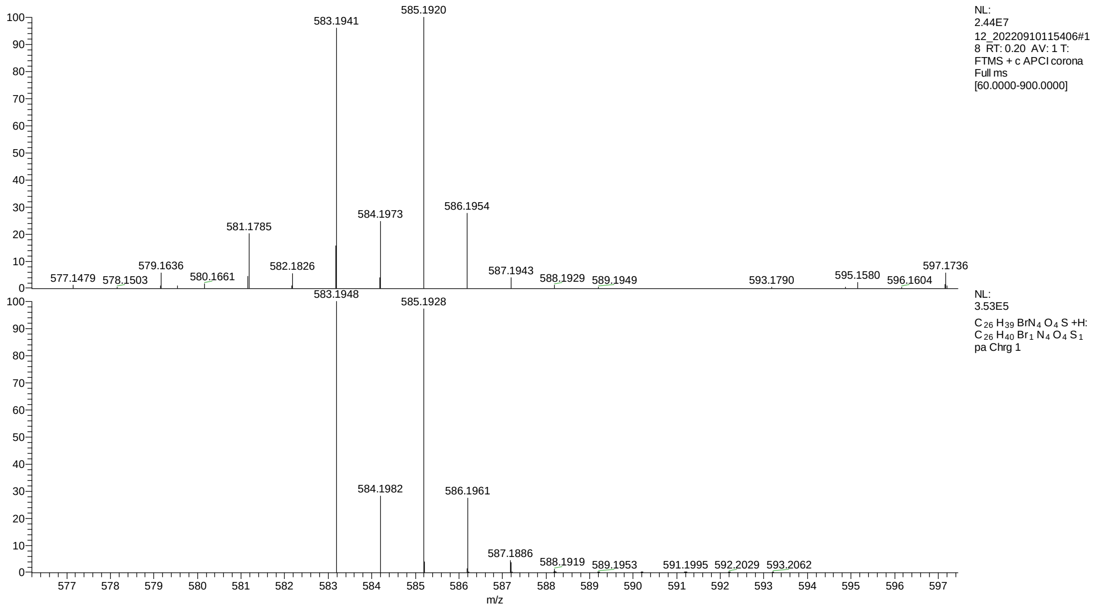

Figure S108. HRMS spectrum of **5l**.

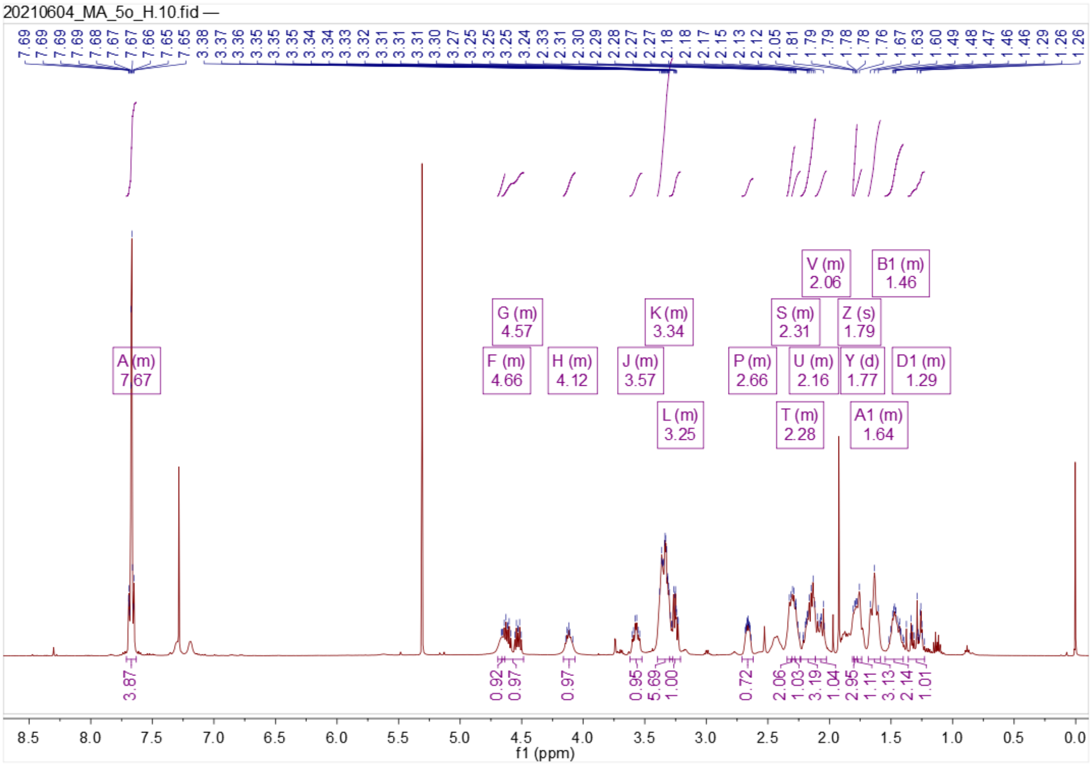

Figure S109. ^{1}H NMR spectrum (Chloroform-*d*, 500 MHz) of **5m**.

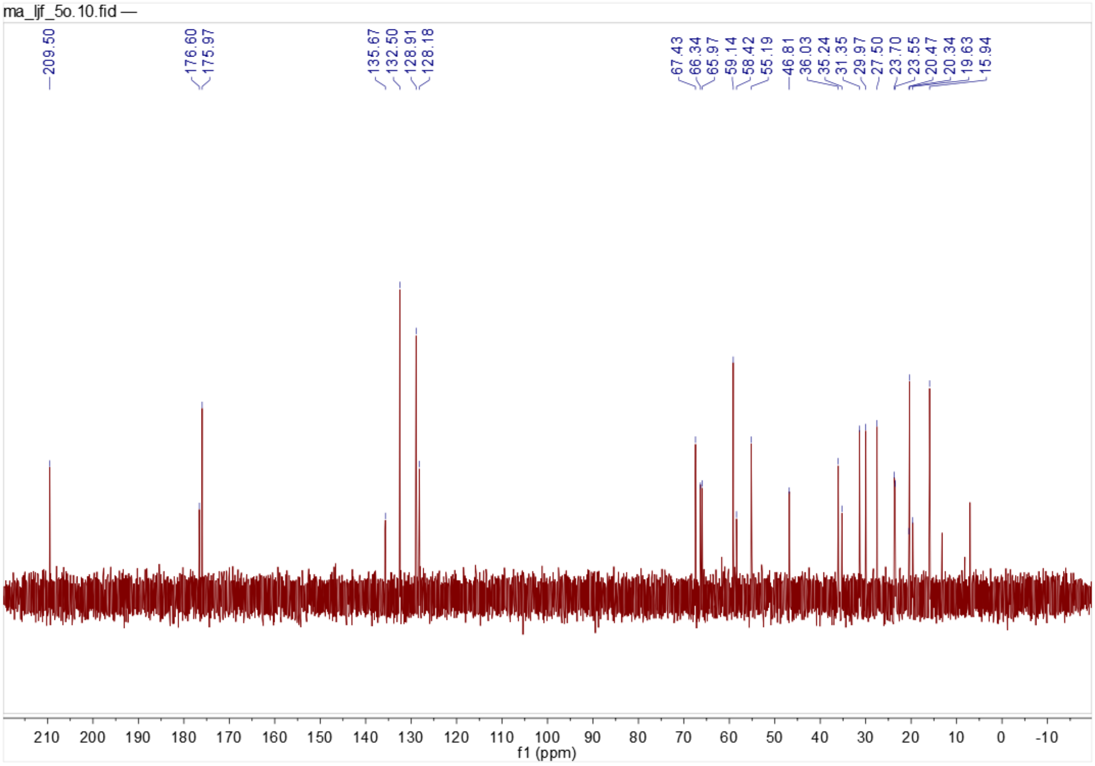

Figure S110. ^{13}C NMR spectrum (Chloroform-*d*, 126 MHz) of **5m**.

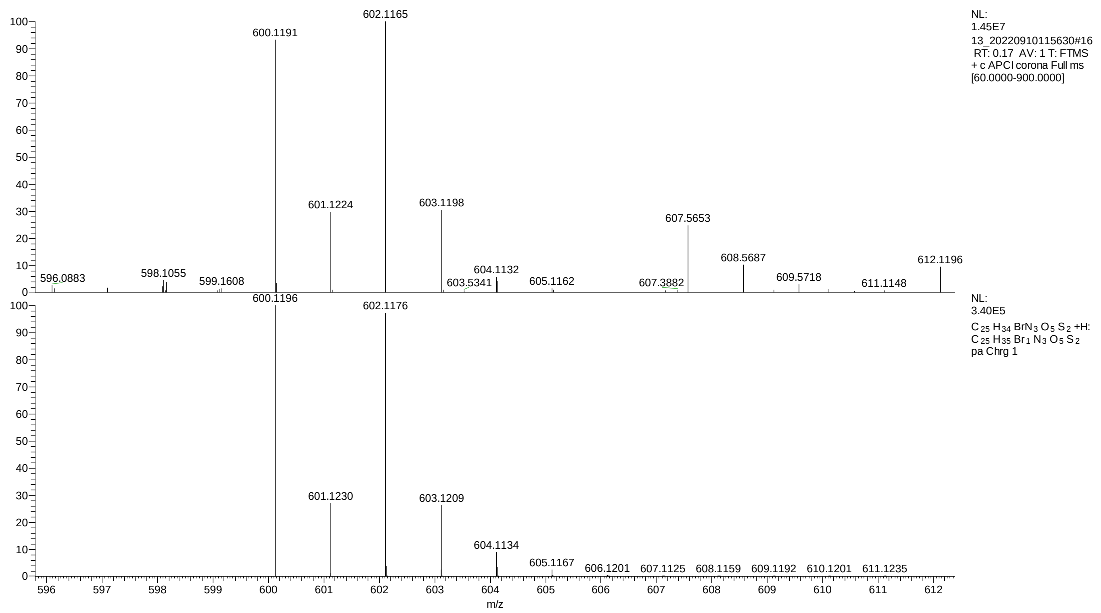

Figure S111. HRMS spectrum of **5m**.

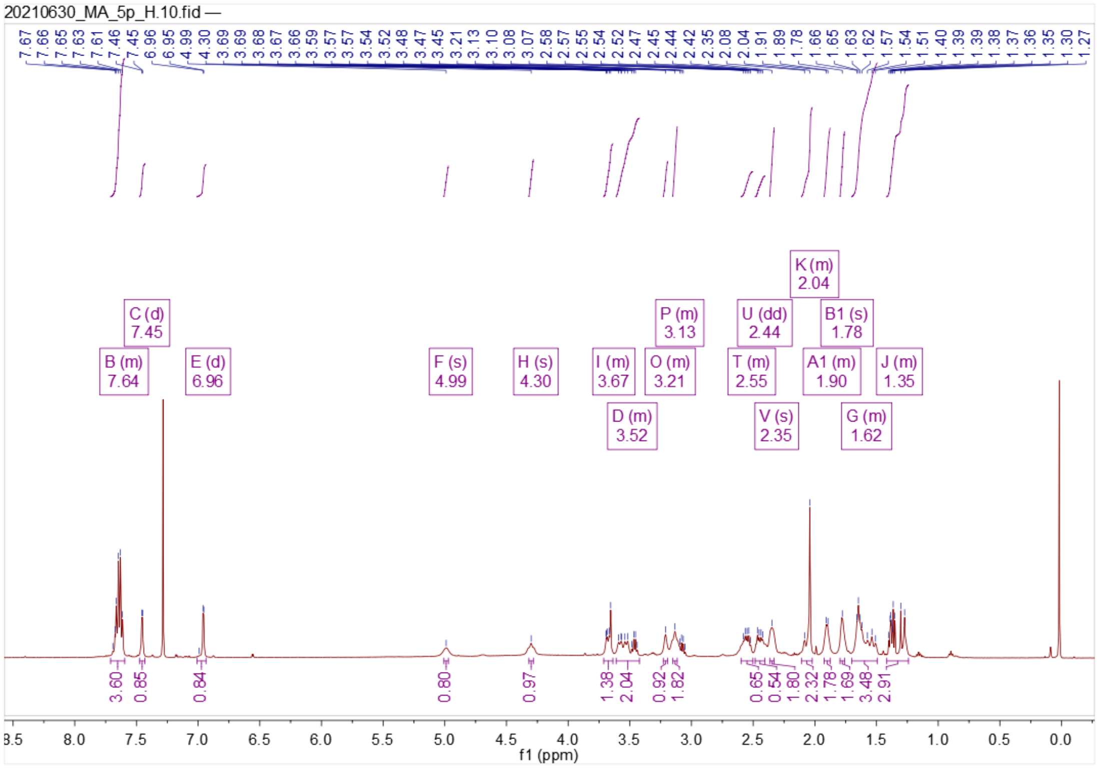

Figure S112. ^{1}H NMR spectrum (Chloroform-*d*, 500 MHz) of **5n**.

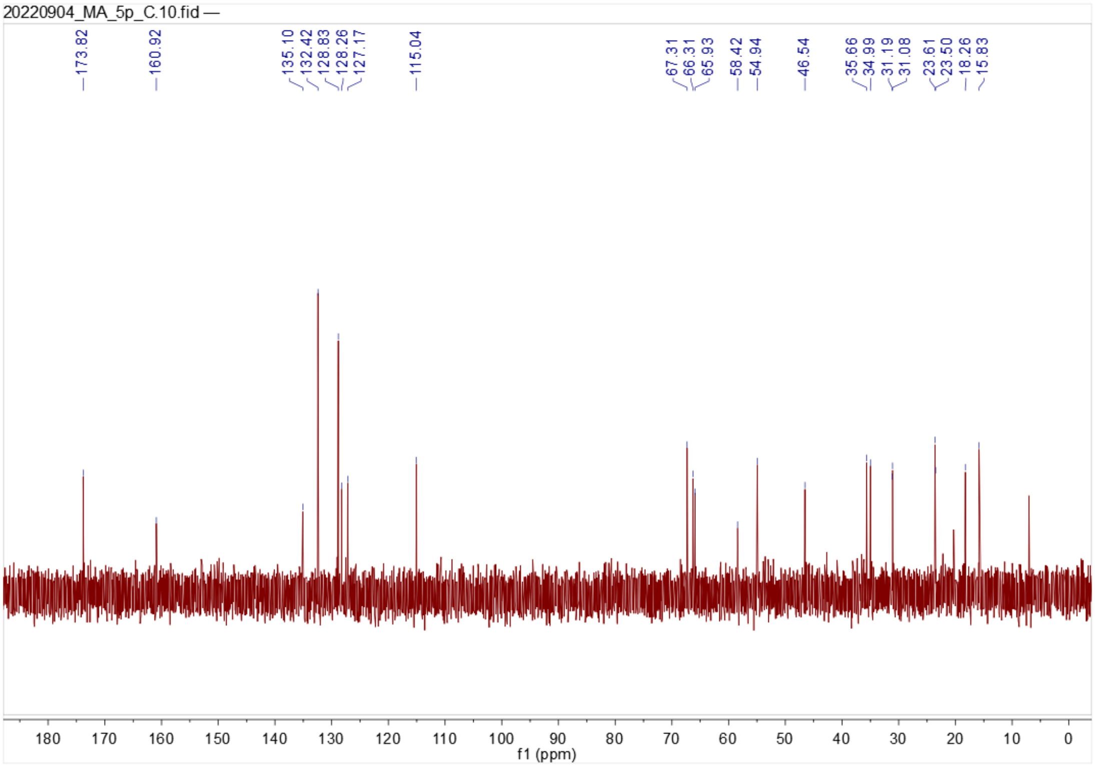

Figure S113. ^{13}C NMR spectrum (Deuterium Oxide, 126 MHz) of **5n**.

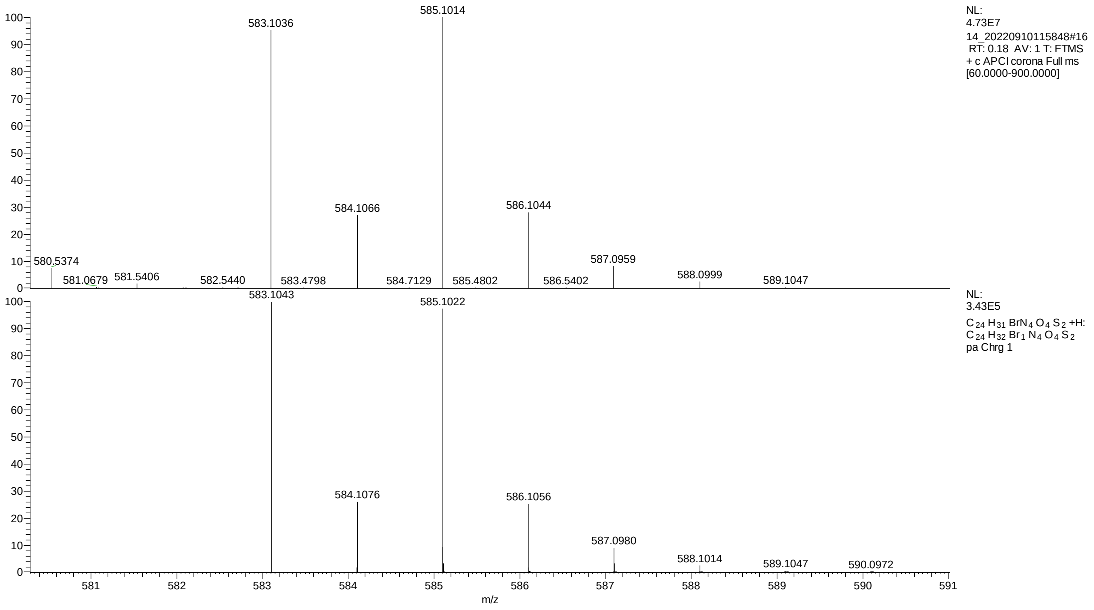

Figure S114. HRMS spectrum of **5n**.

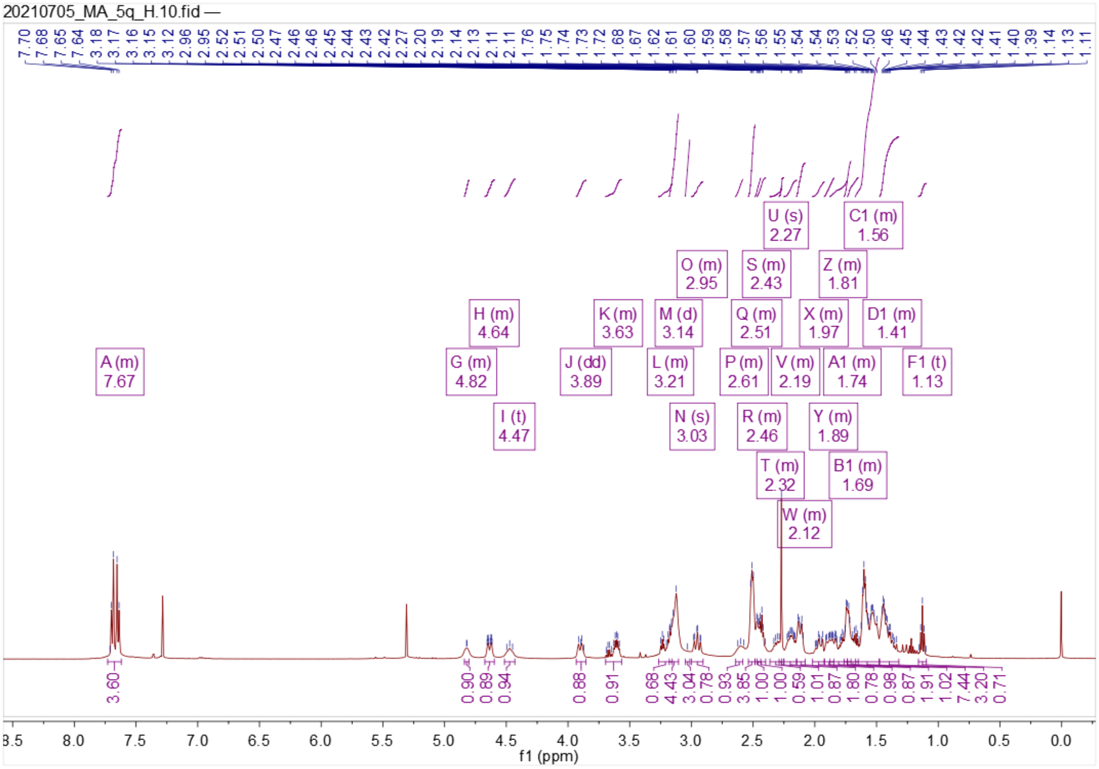

Figure S115. ^{1}H NMR spectrum (Chloroform-*d*, 500 MHz) of **5o**.

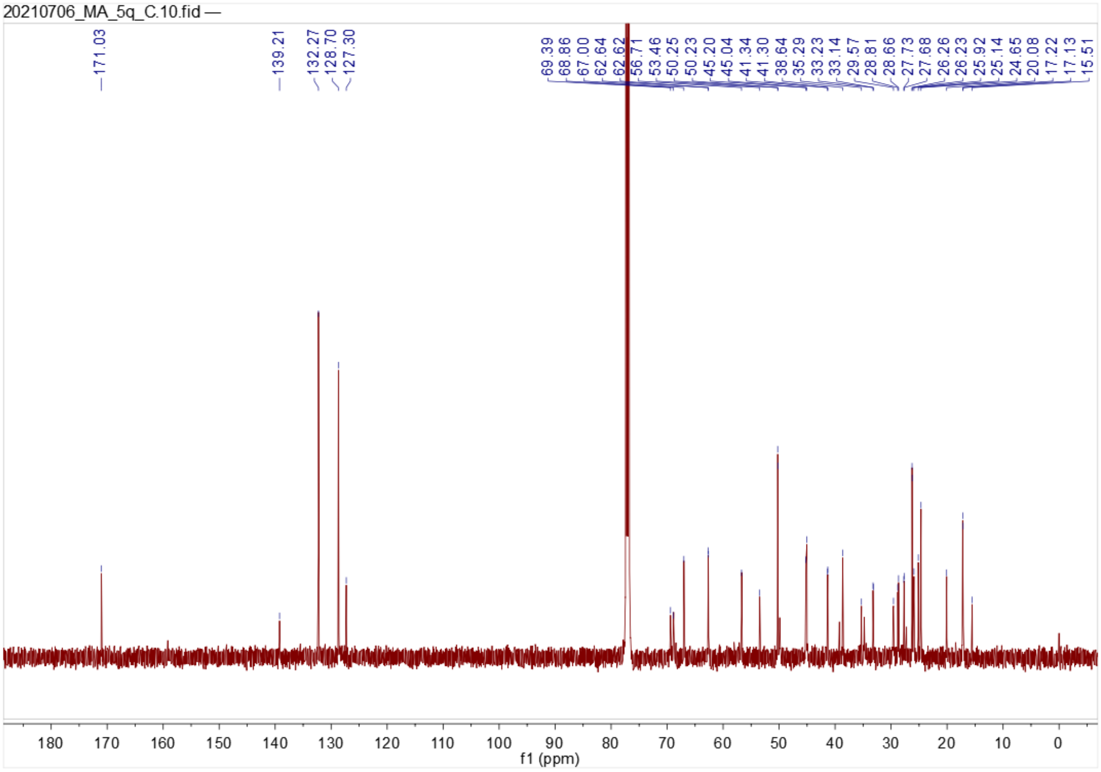

Figure S116. ^{13}C NMR spectrum (Chloroform-*d*, 126 MHz) of **5o**.

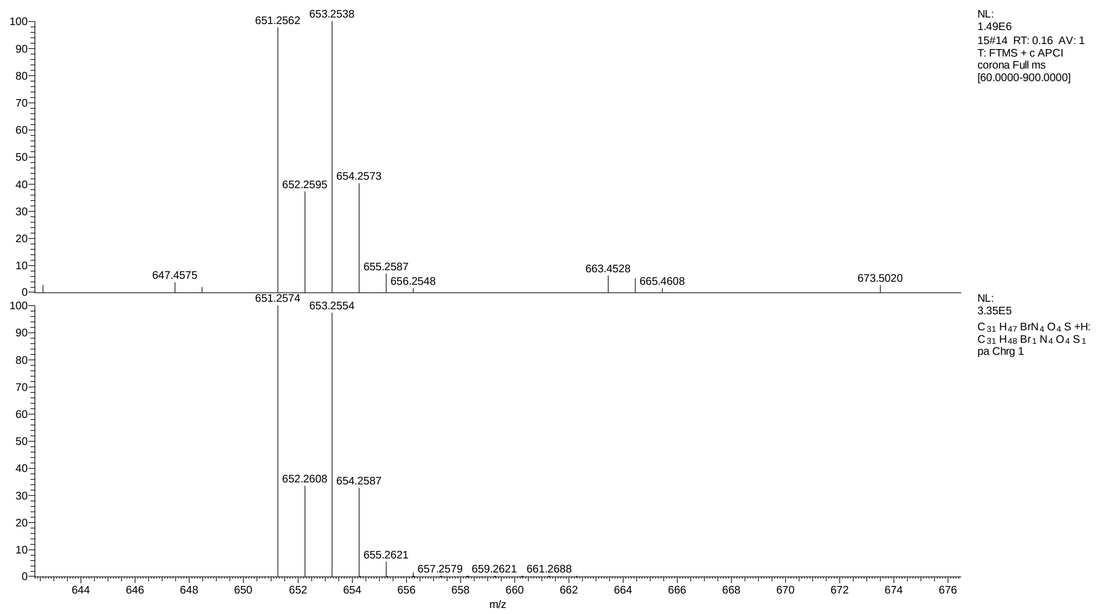

Figure S117. HRMS spectrum of **5o**.

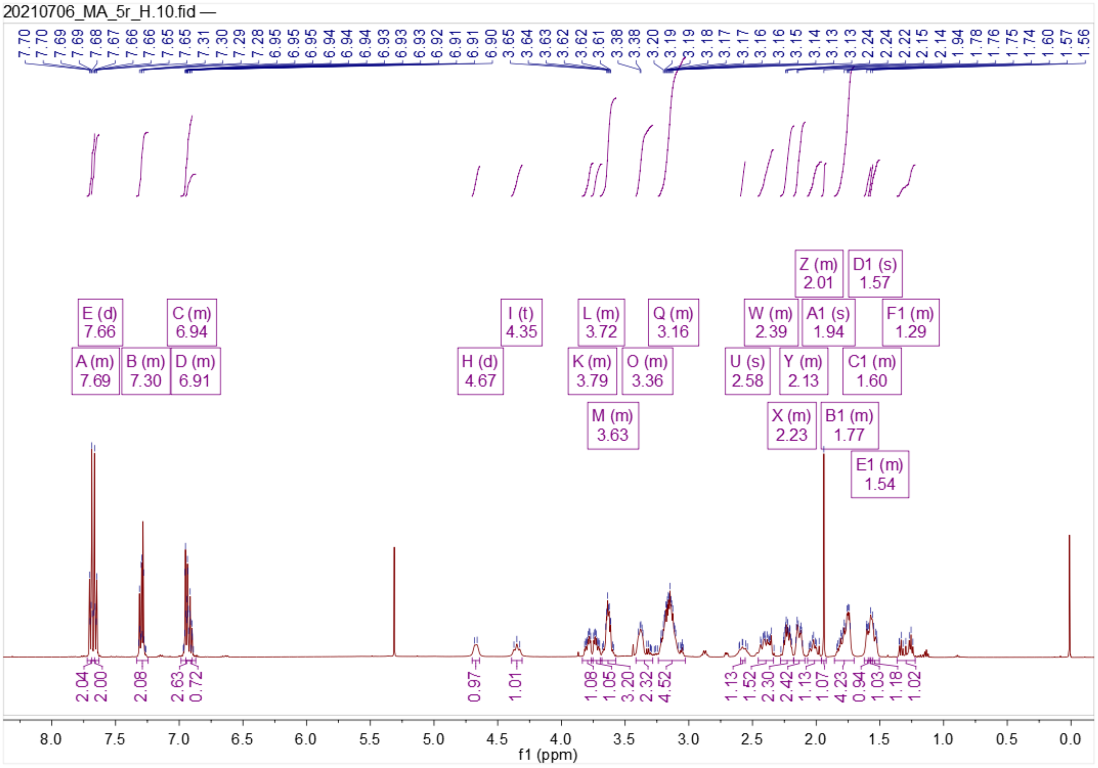

Figure S118. ^{1}H NMR spectrum (Chloroform-d, 500 MHz) of **5p**.

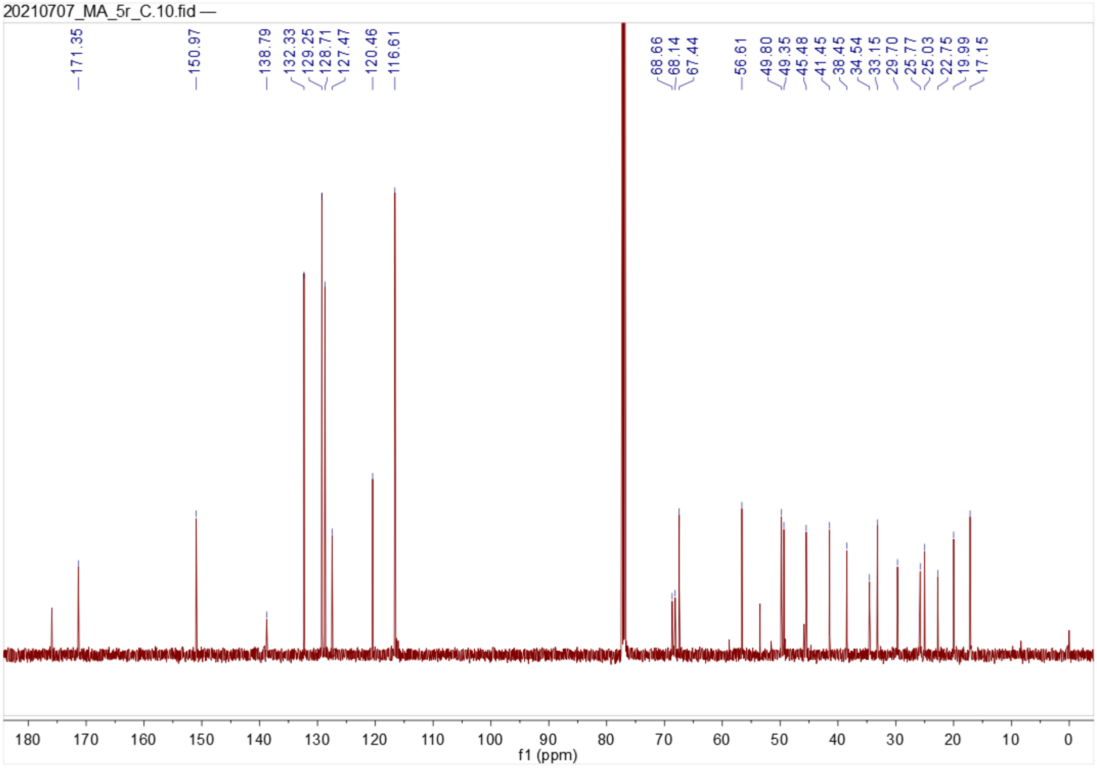

Figure S119. ^{13}C NMR spectrum (Chloroform-*d*, 126 MHz) of **5p**.

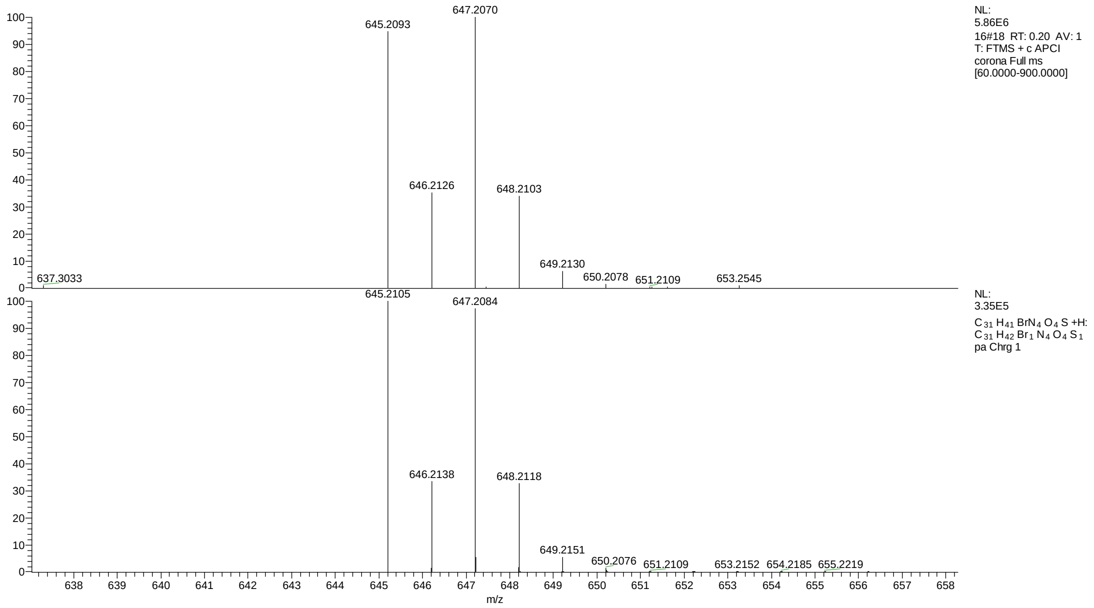

Figure S120. HRMS spectrum of **5p**.

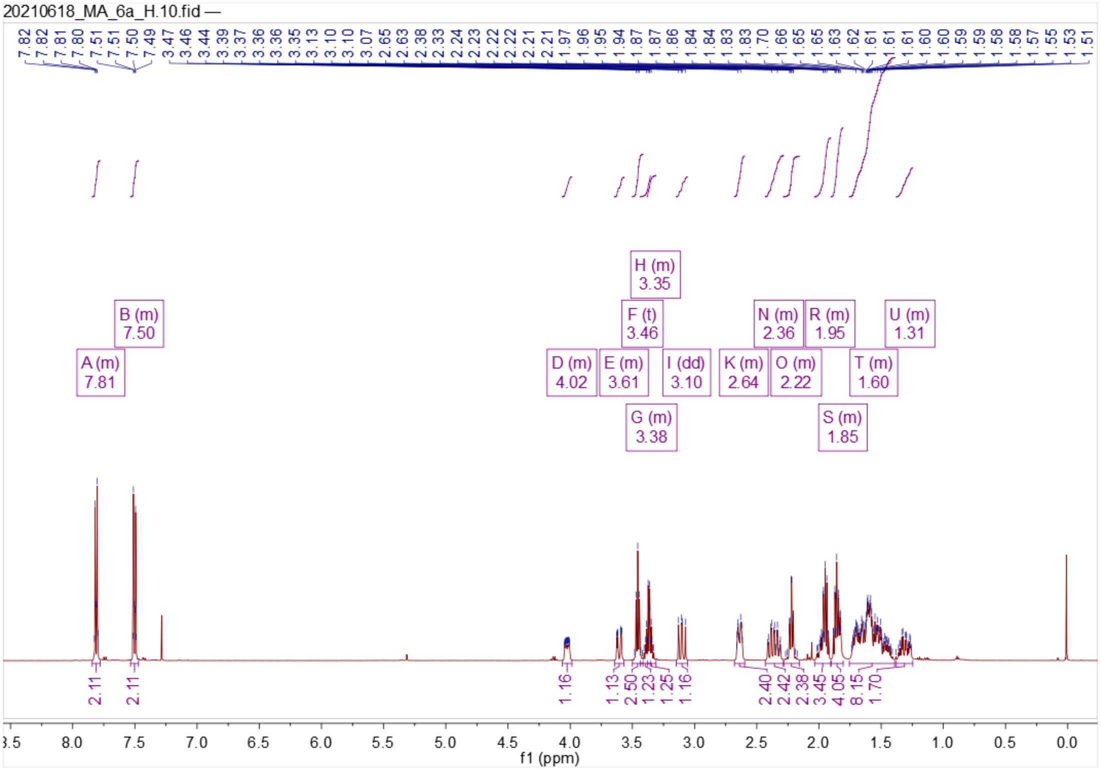

Figure S121. ^{1}H NMR spectrum (Chloroform-*d*, 500 MHz) of **6a**.

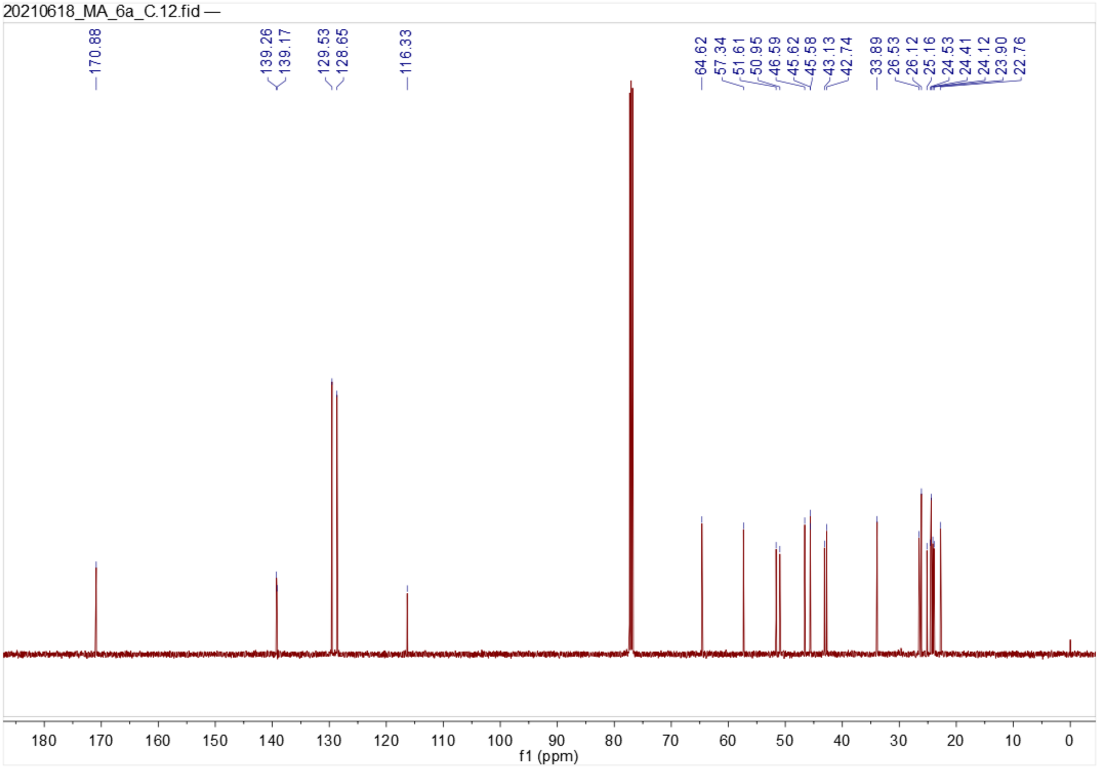

Figure S122. ^{13}C NMR spectrum (Chloroform-*d*, 126 MHz) of **6a**.

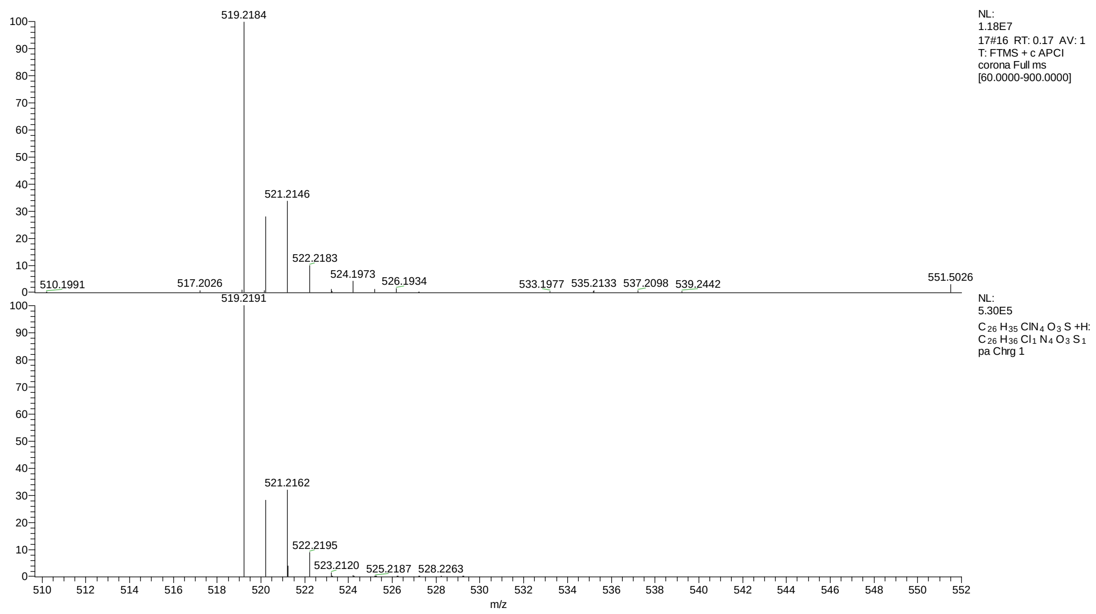

Figure S123. HRMS spectrum of **6a**.

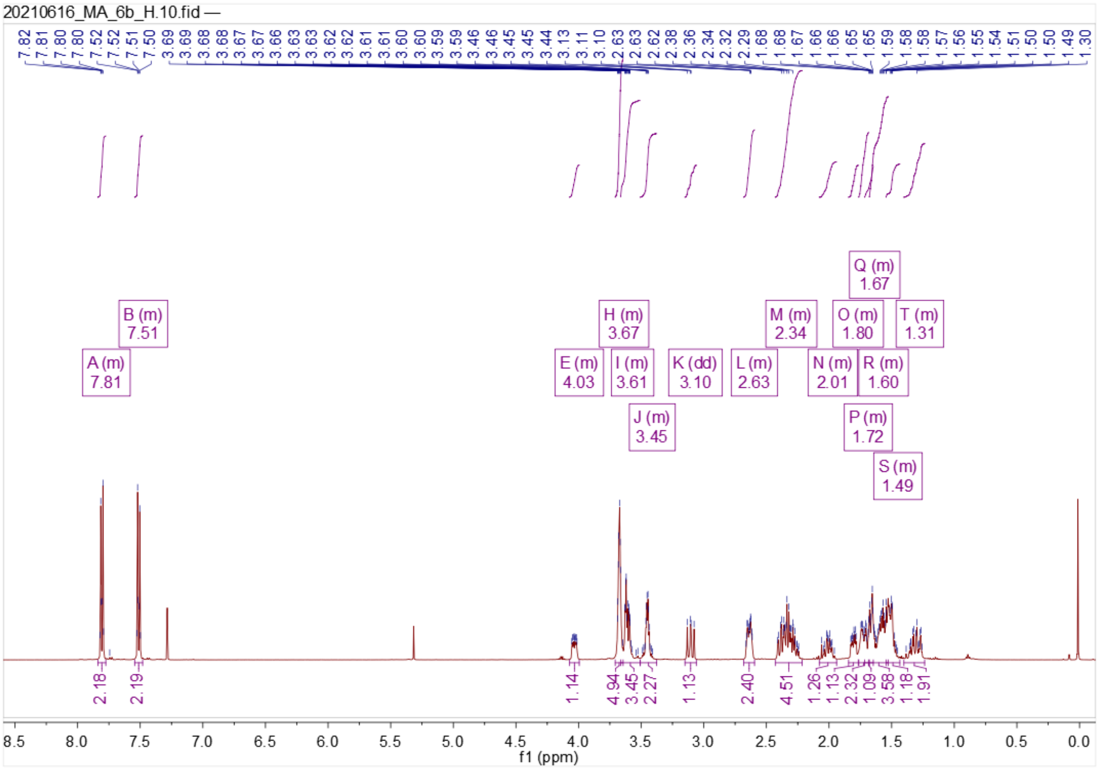

Figure S124. ^{1}H NMR spectrum (Chloroform-*d*, 500 MHz) of **6b**.

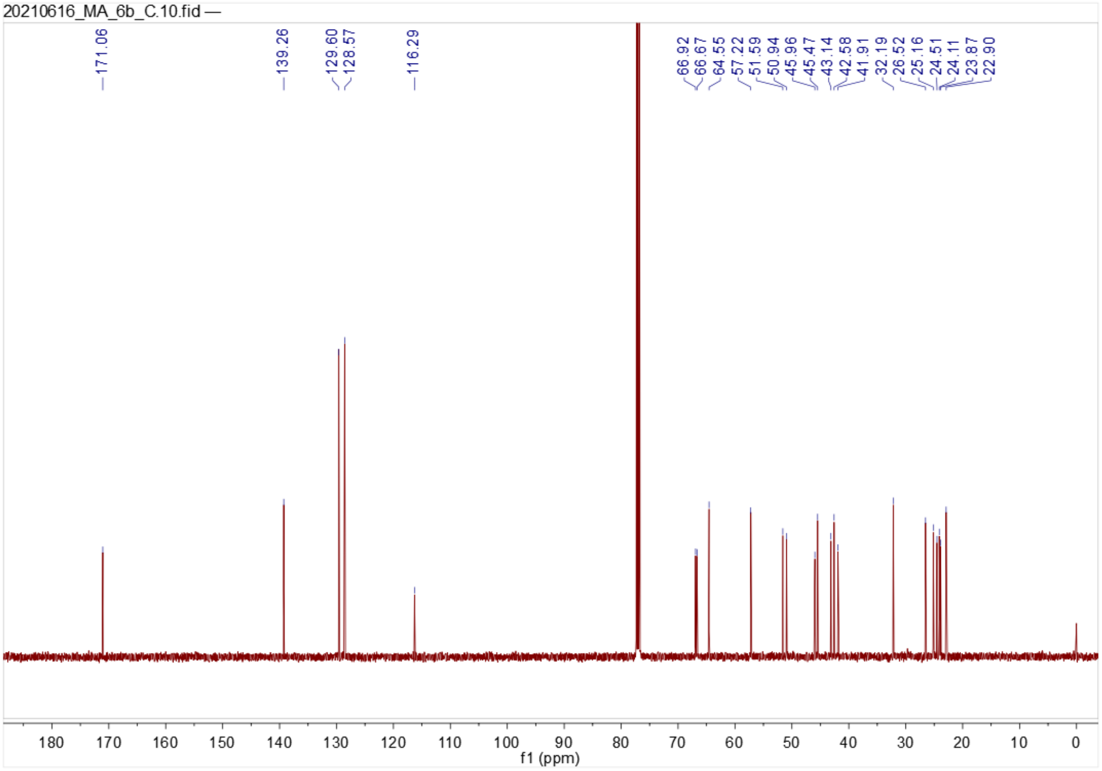

Figure S125. ^{13}C NMR spectrum (Chloroform-*d*, 126 MHz) of **6b**.

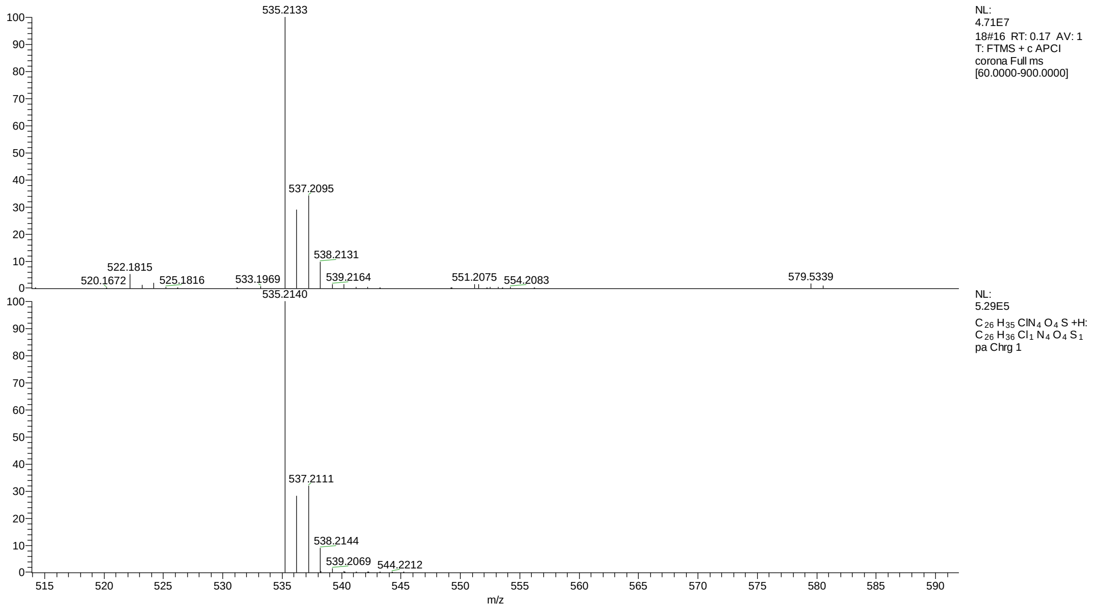

Figure S126. HRMS spectrum of **6b**.

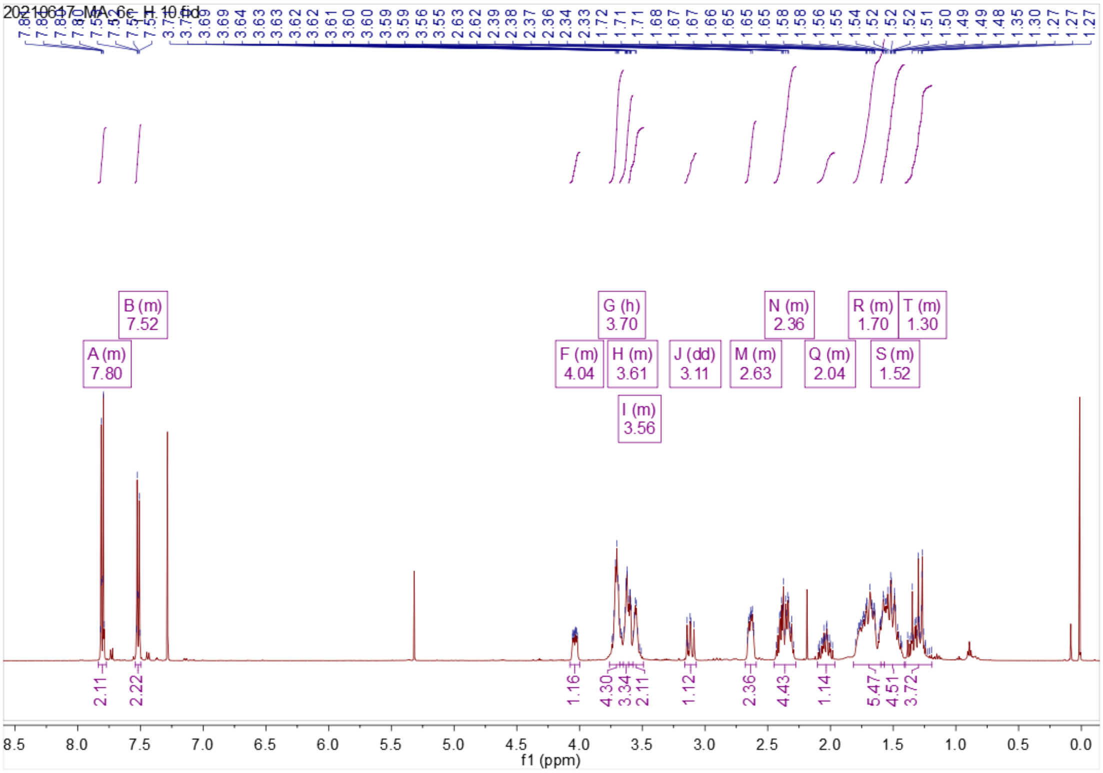

Figure S127. ^{1}H NMR spectrum (Chloroform-*d*, 500 MHz) of **6c**.

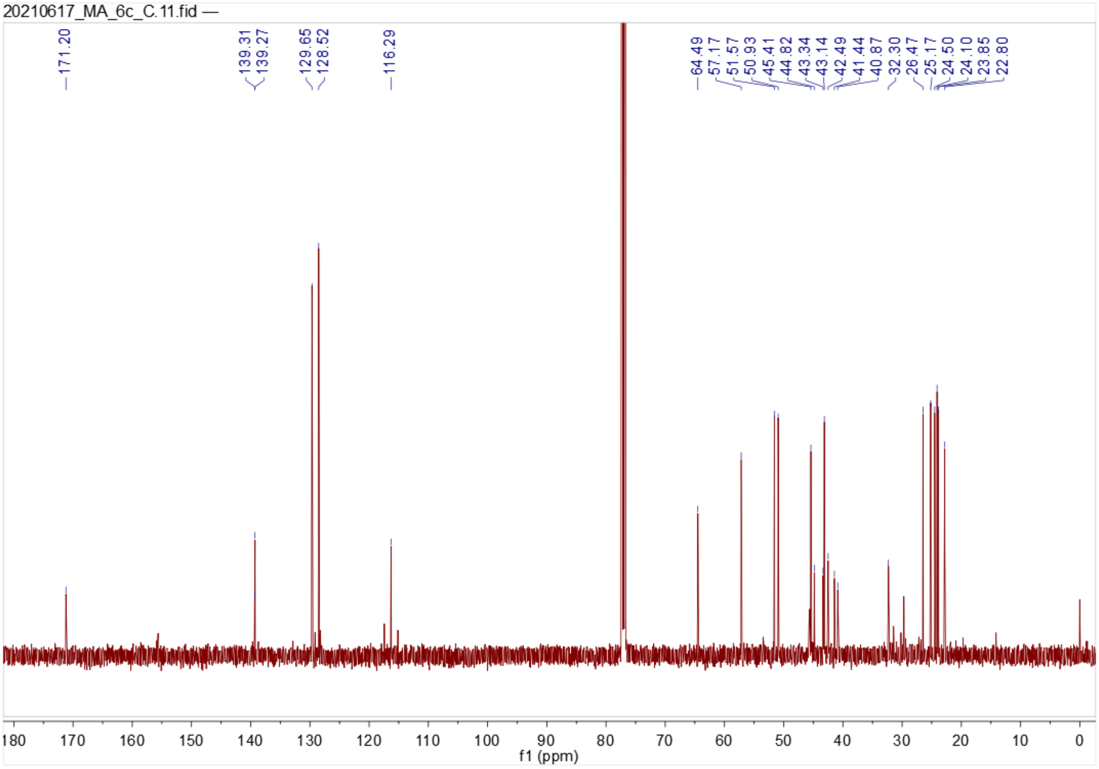

Figure S128. ^{13}C NMR spectrum (Chloroform-*d*, 126 MHz) of **6c**.

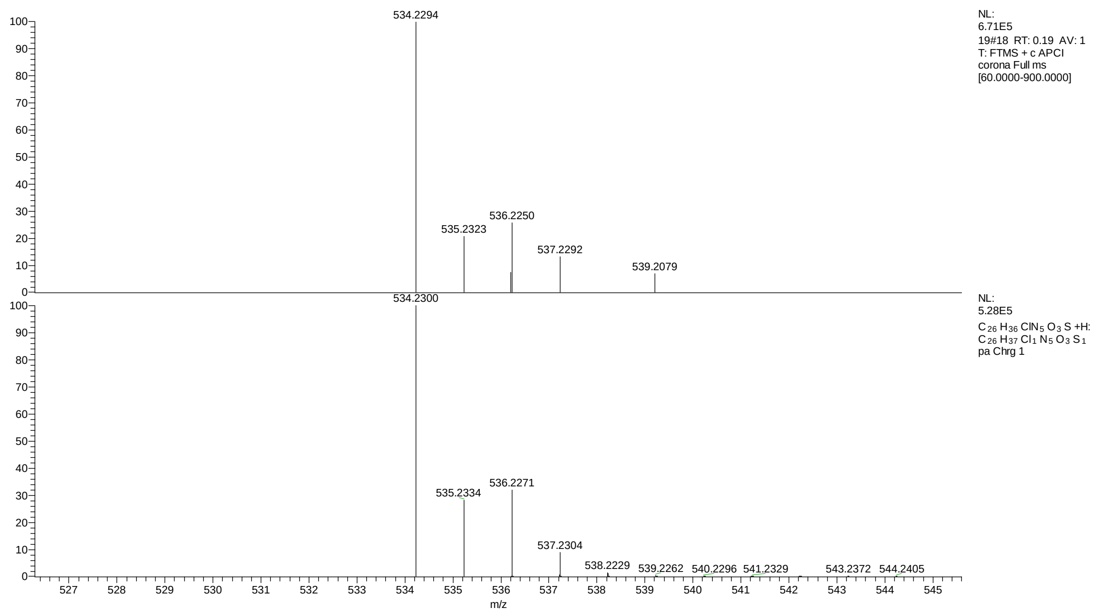

Figure S129. HRMS spectrum of **6c**.

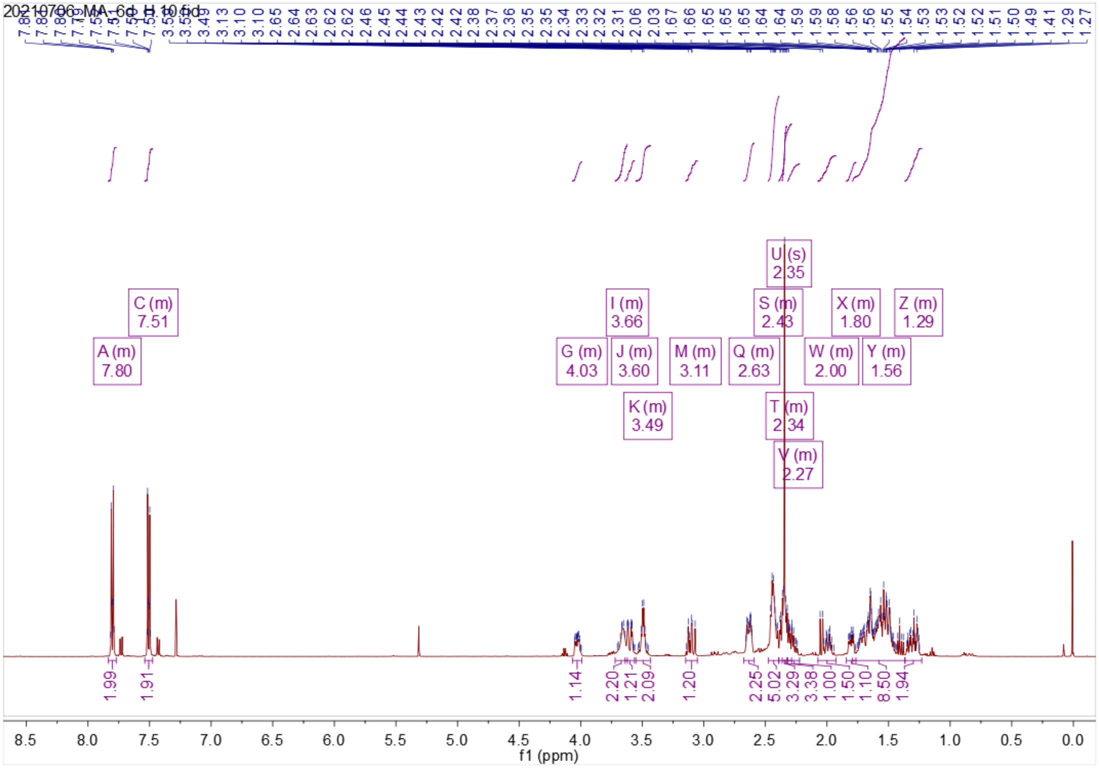

Figure S130. ^{1}H NMR spectrum (Chloroform-*d*, 500 MHz) of **6d**.

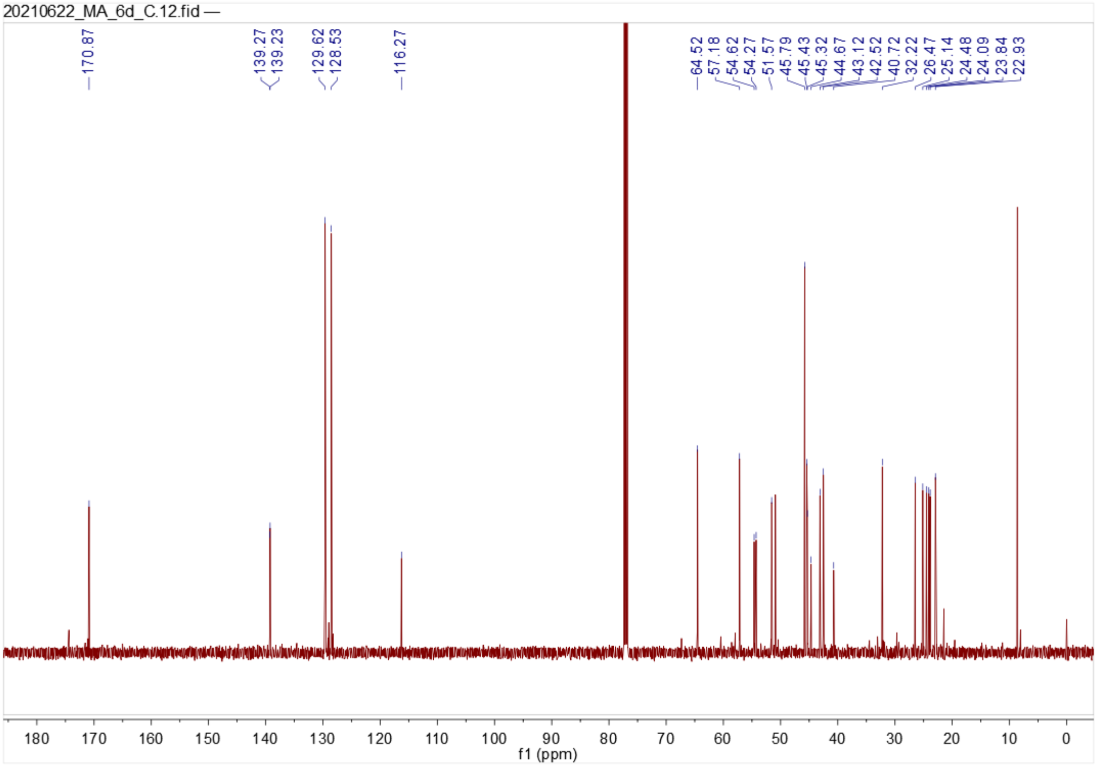

Figure S131. ^{13}C NMR spectrum (Chloroform-*d*, 126 MHz) of **6d**.

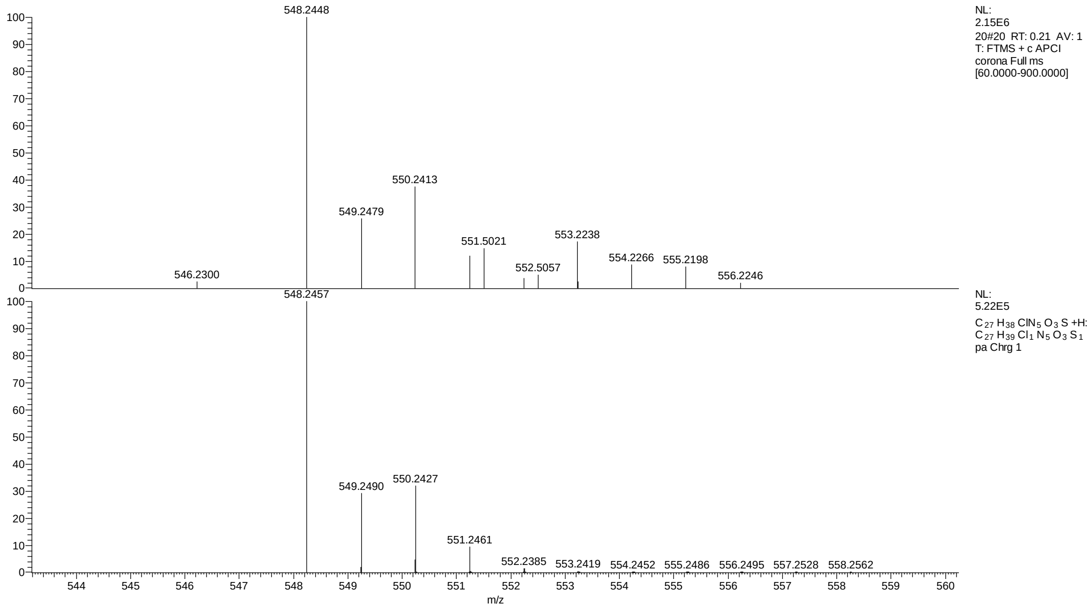

Figure S132. HRMS spectrum of **6d**.

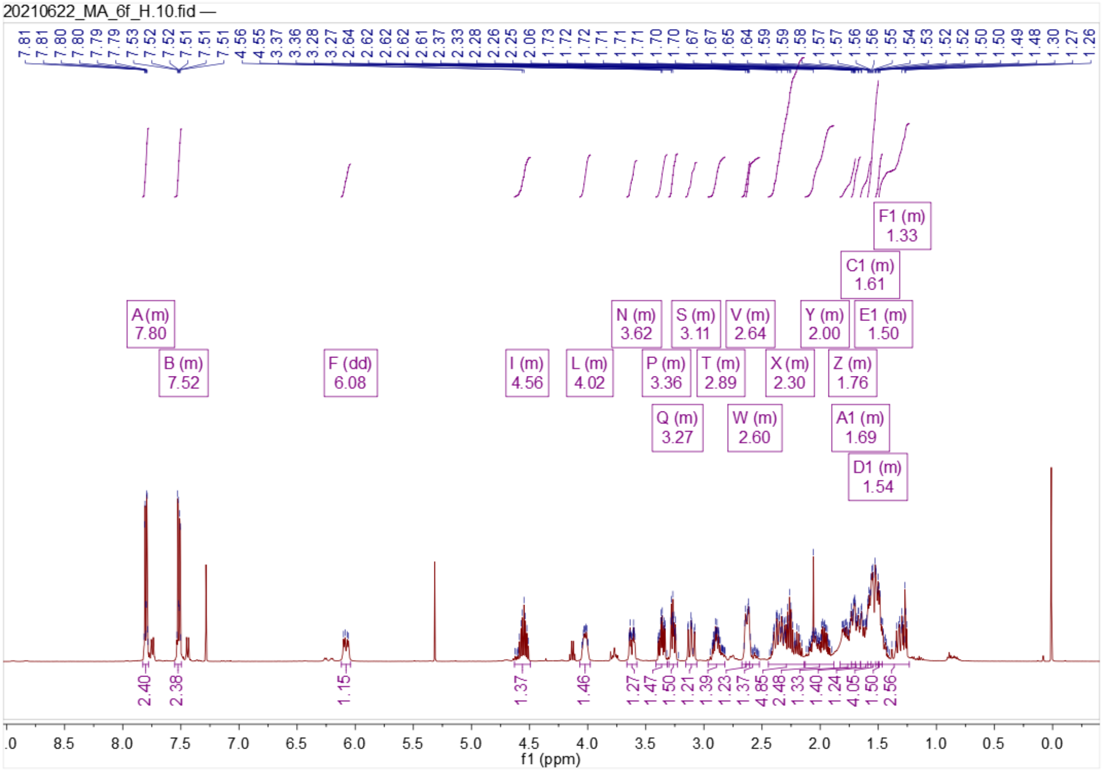

Figure S133. ^{1}H NMR spectrum (Chloroform-*d*, 500 MHz) of **6e**.

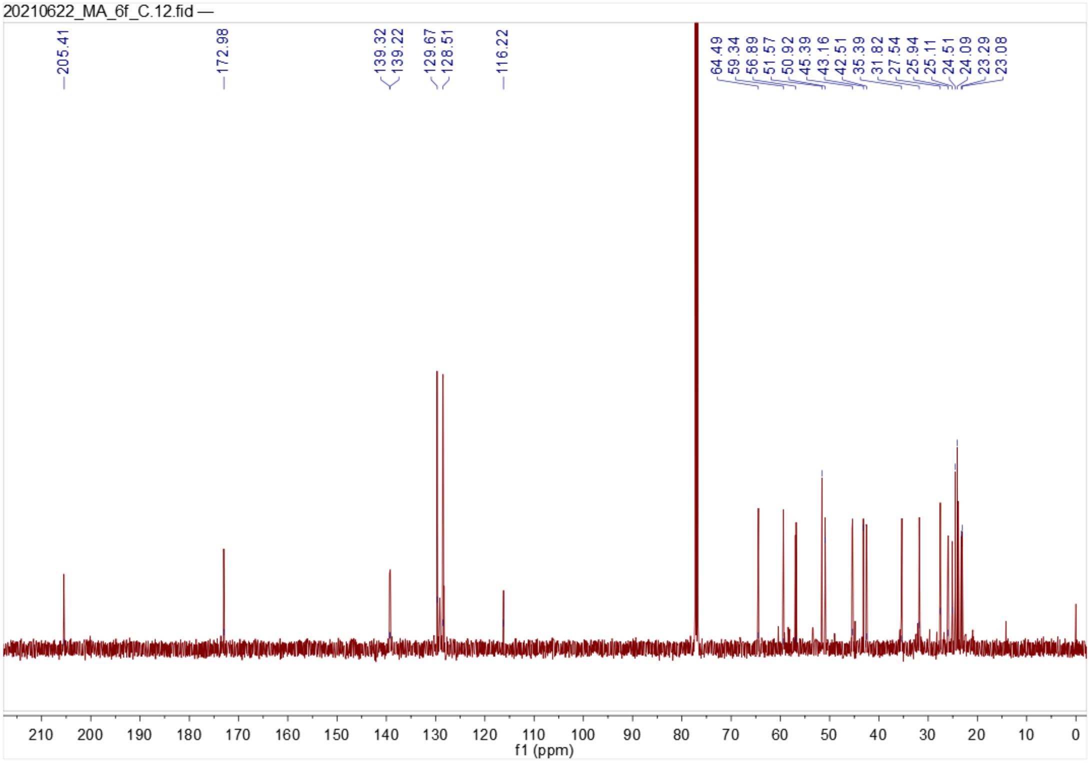

Figure S134. ^{13}C NMR spectrum (Chloroform-*d*, 126 MHz) of **6e**.

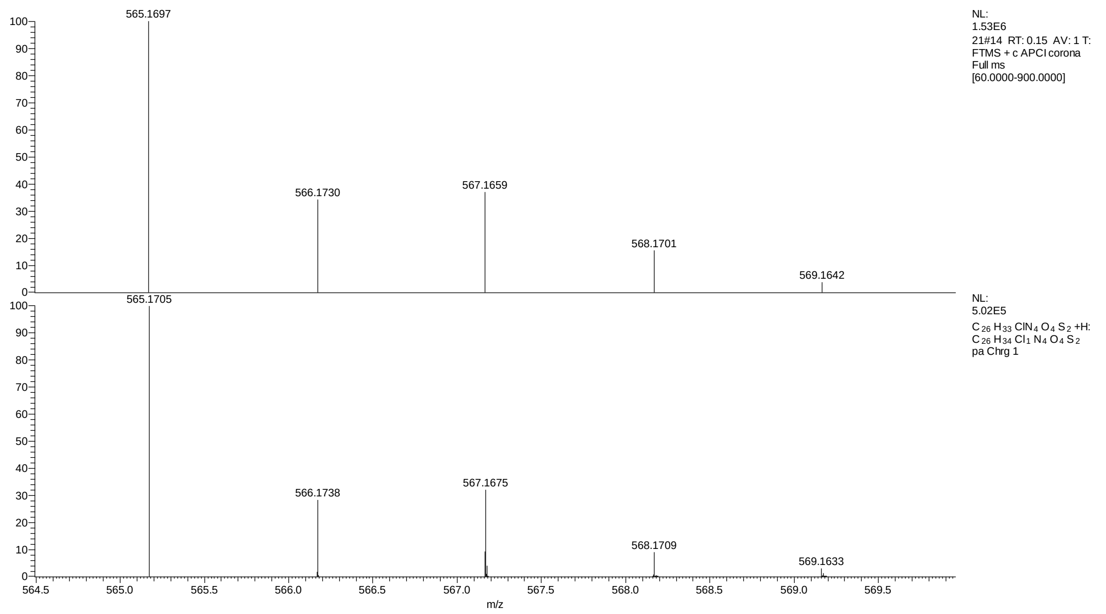

Figure S135. HRMS spectrum of **6e**.

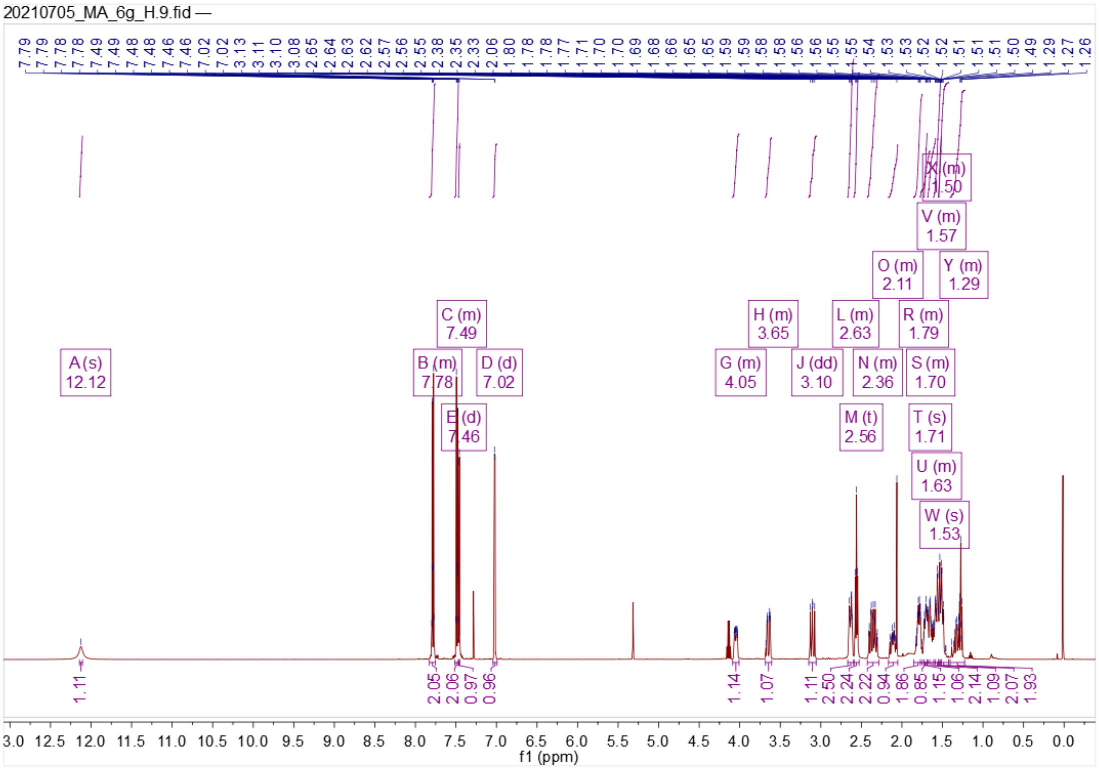

Figure S136. ^{1}H NMR spectrum (Chloroform-*d*, 500 MHz) of **6f**.

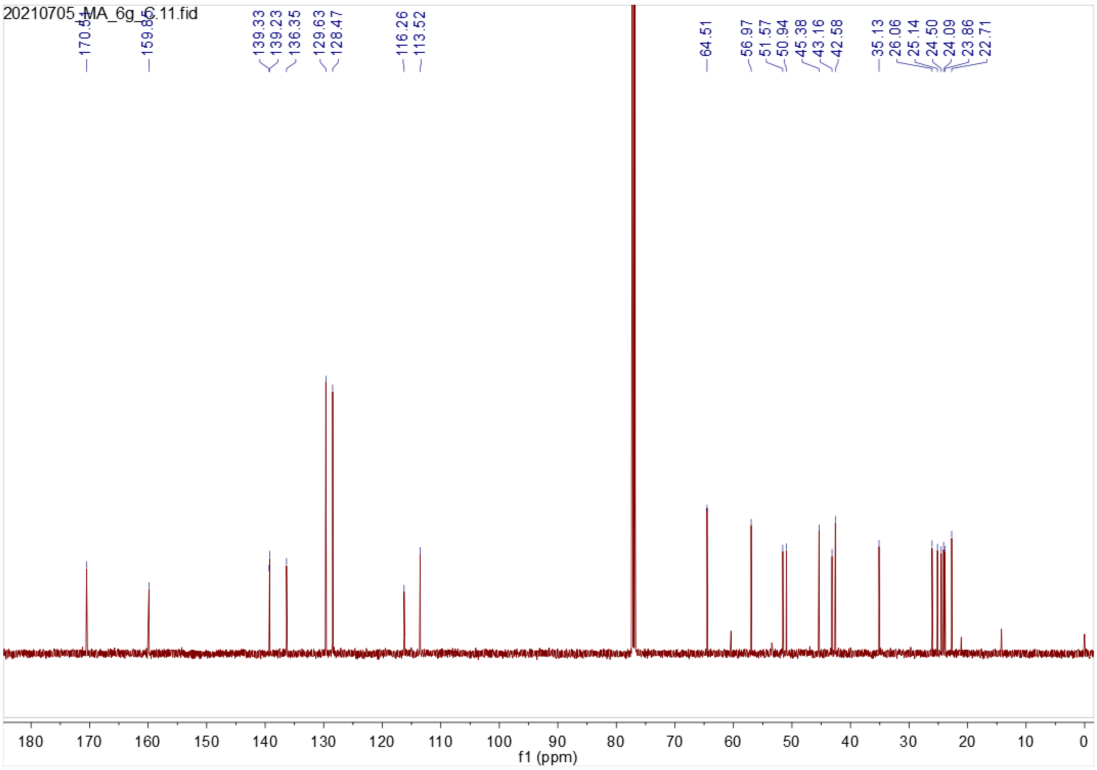

Figure S137. ^{13}C NMR spectrum (Chloroform-*d*, 126 MHz) of **6f**.

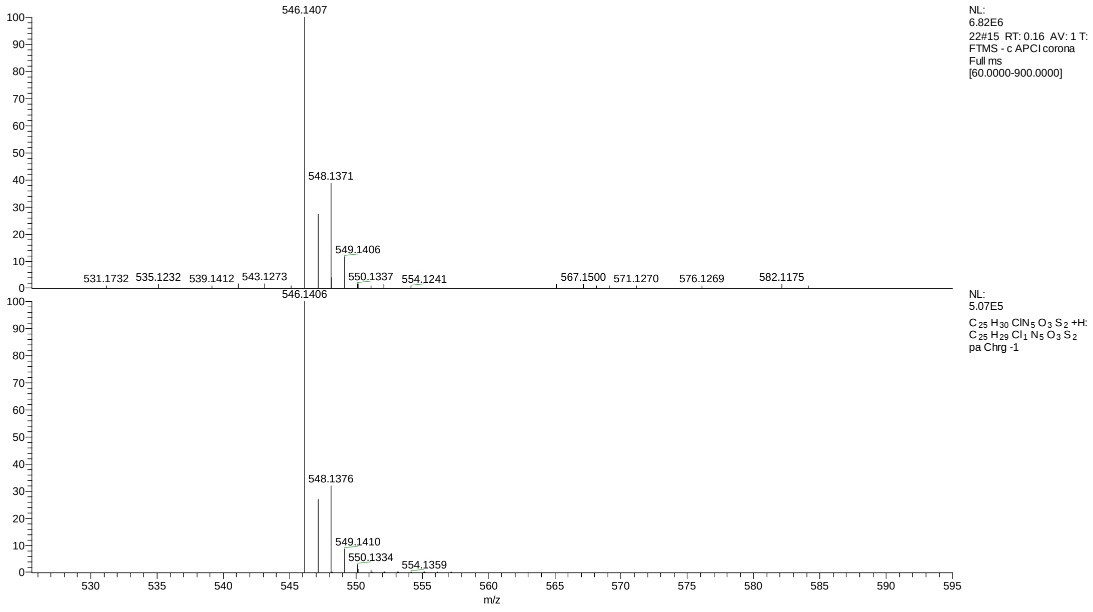

Figure S138. HRMS spectrum of **6f**.

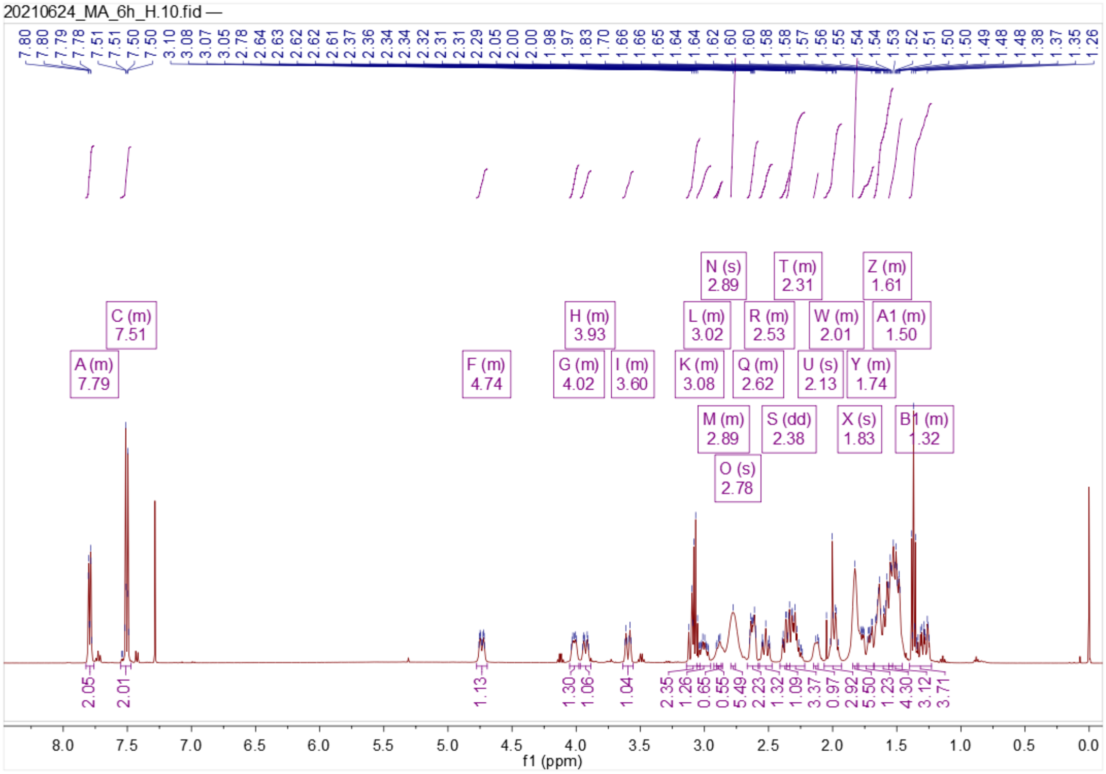

Figure S139. ^{1}H NMR spectrum (Chloroform-*d*, 500 MHz) of **6g**.

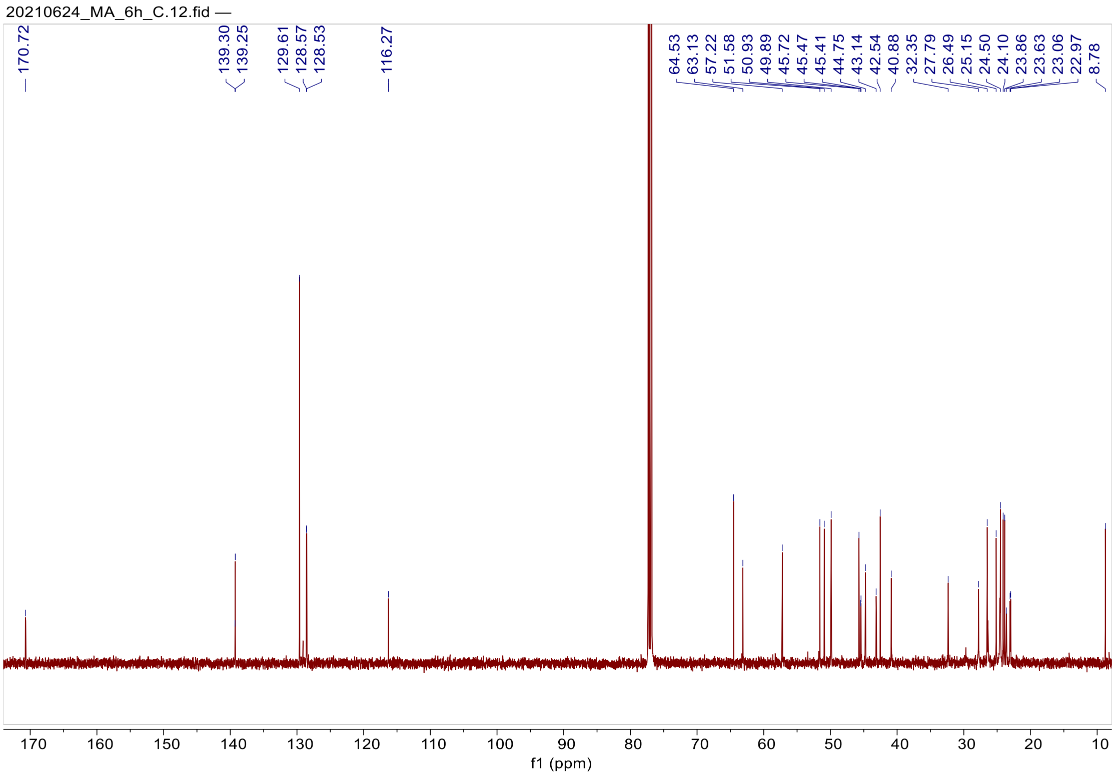

Figure S140. ^{13}C NMR spectrum (Chloroform-*d*, 126 MHz) of **6g**.

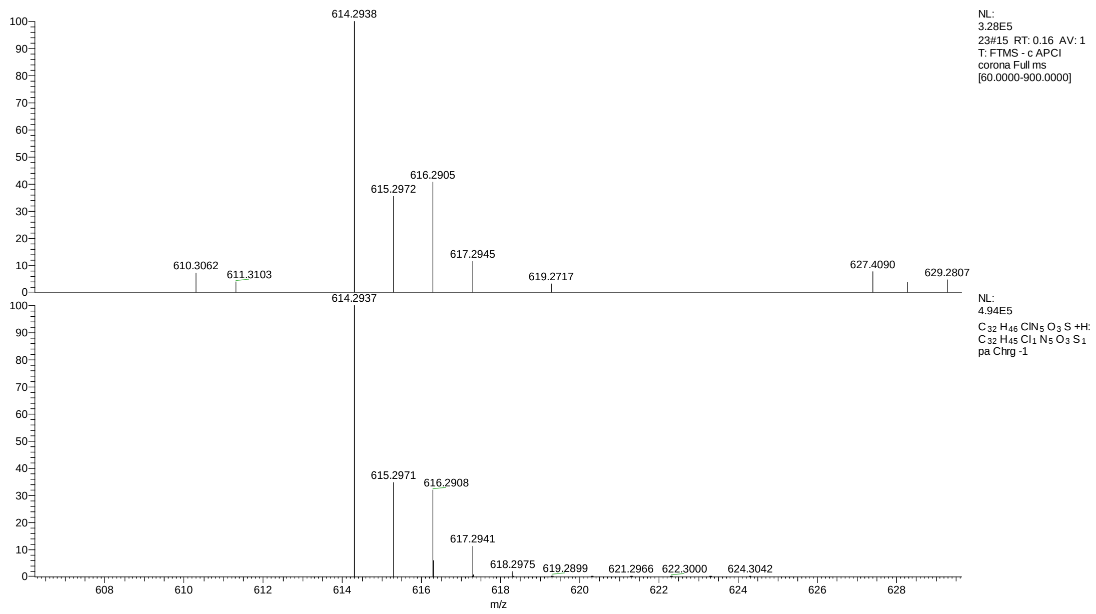

Figure S141. HRMS spectrum of **6g**.

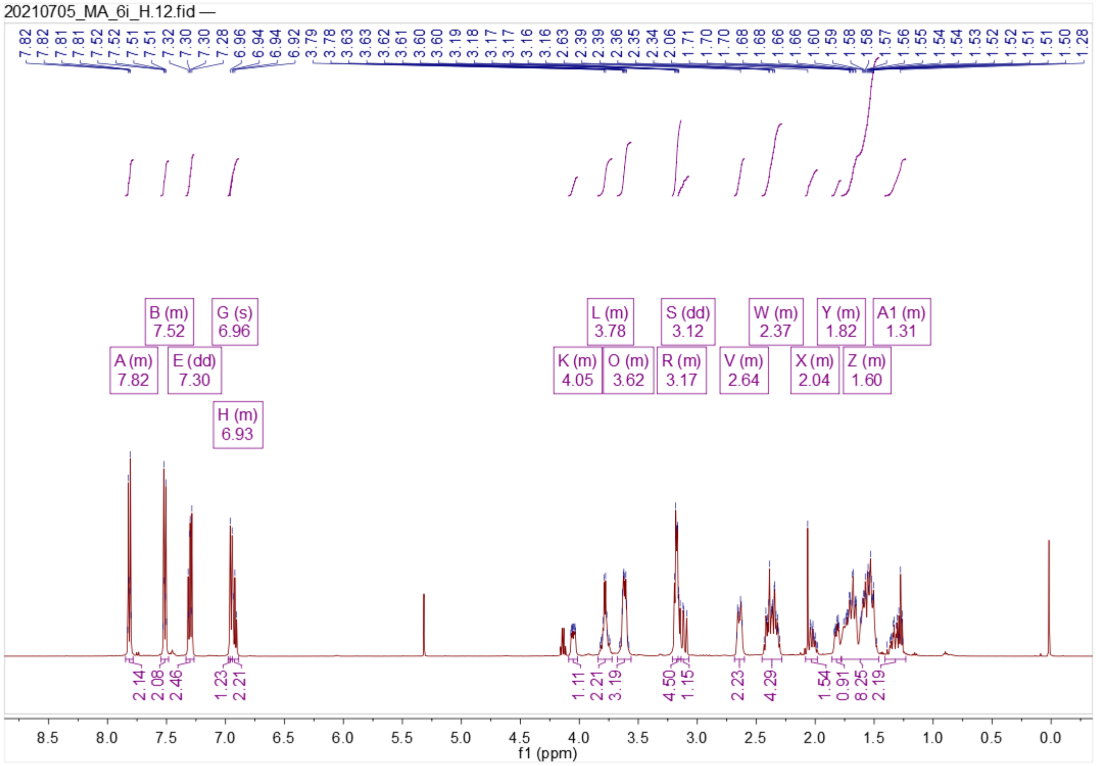

Figure S142. ^{1}H NMR spectrum (Chloroform-*d*, 500 MHz) of **6h**.

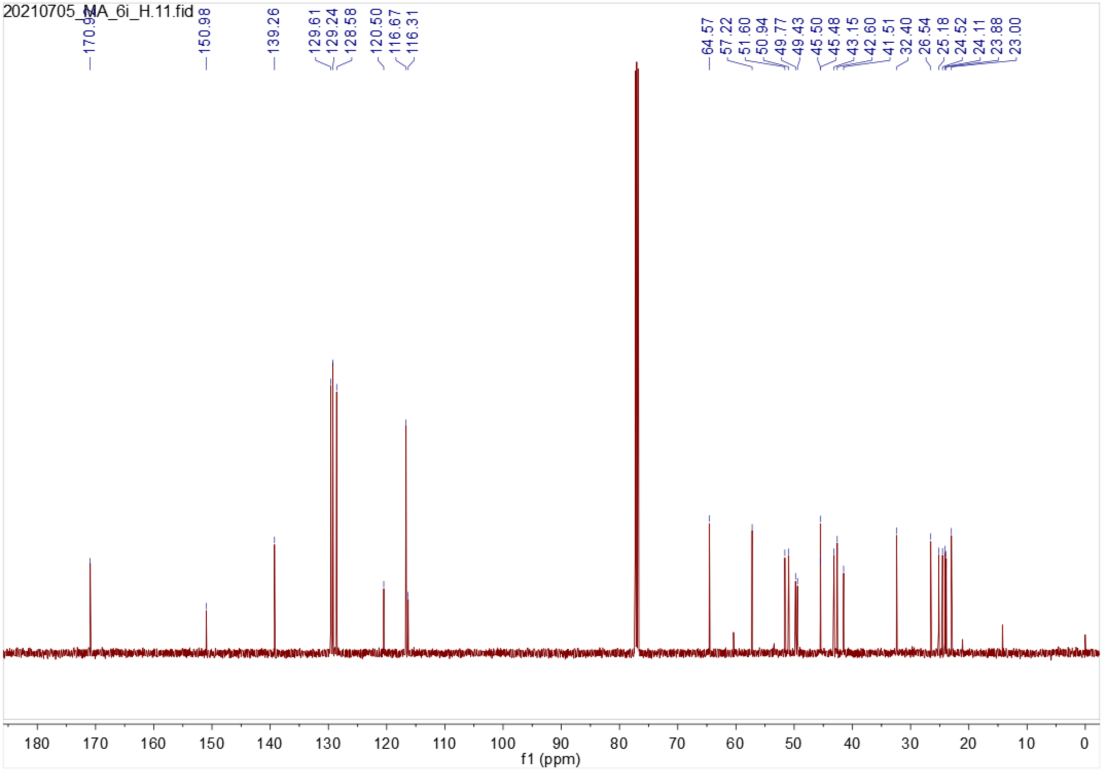

Figure S143. ^{13}C NMR spectrum (Chloroform-*d*, 126 MHz) of **6h**.

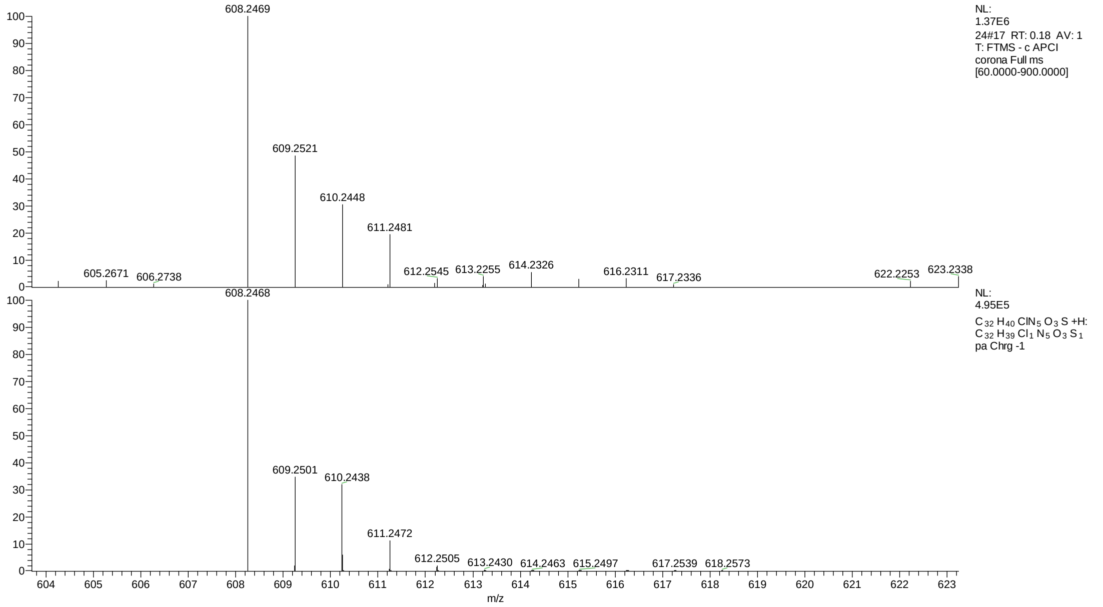

Figure S144. HRMS spectrum of **6h**.

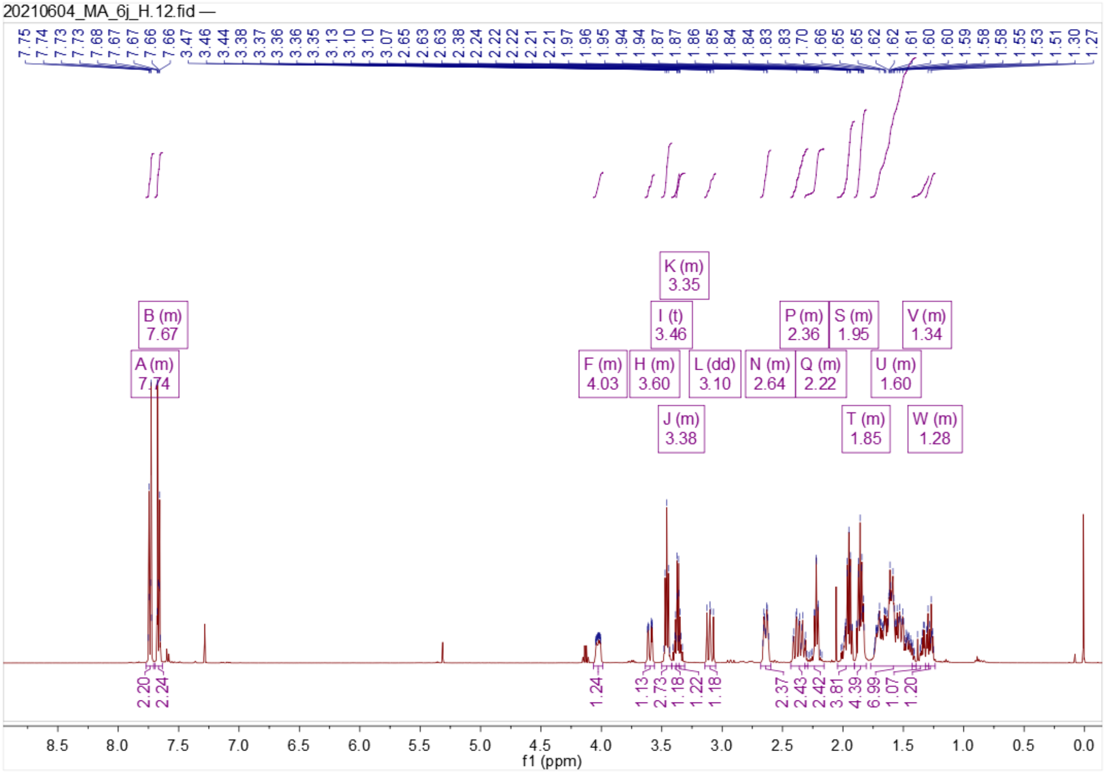

Figure S145. ^{1}H NMR spectrum (Chloroform-*d*, 500 MHz) of **6i**.

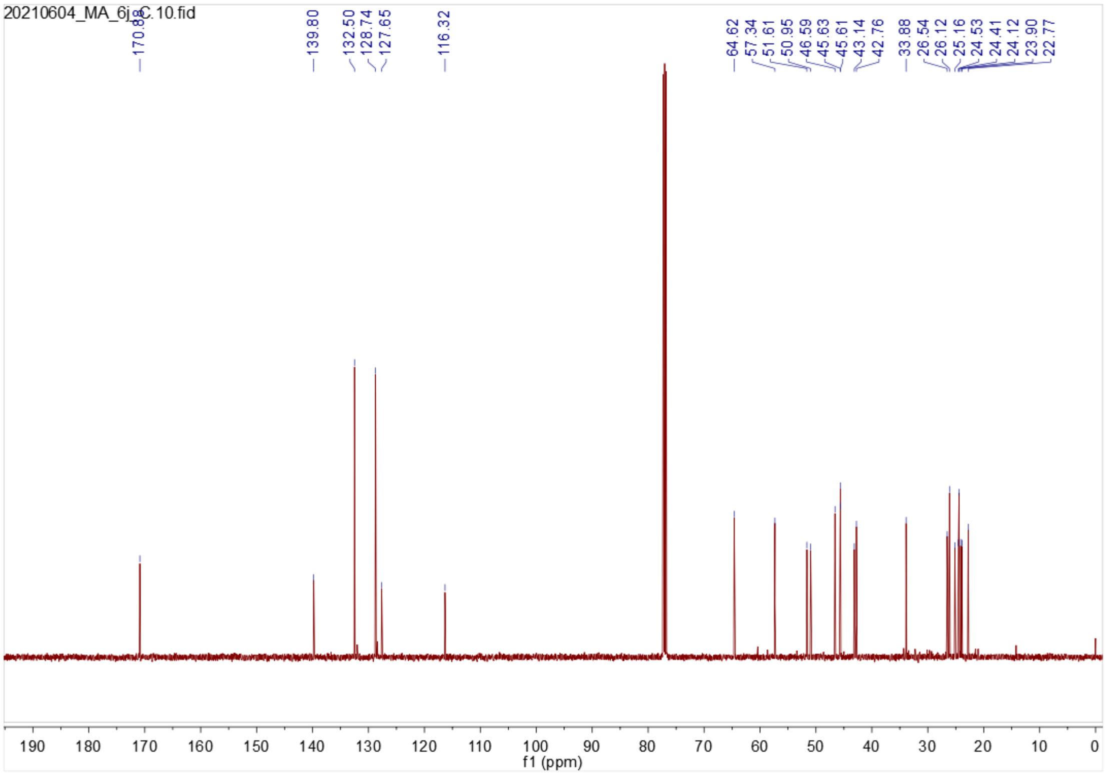

Figure S146. ^{13}C NMR spectrum (Chloroform-*d*, 126 MHz) of **6i**.

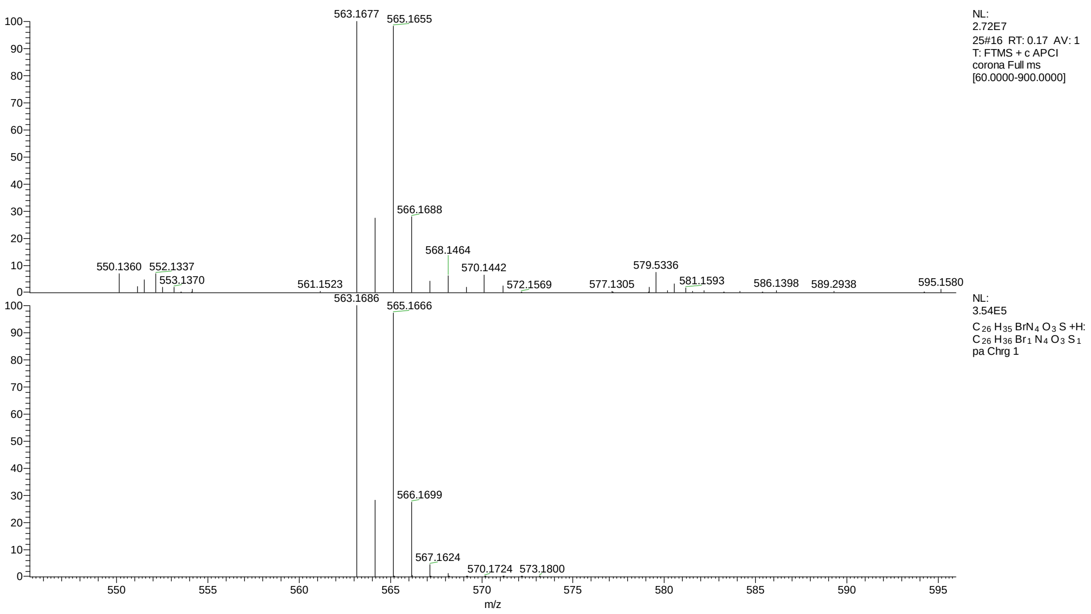

Figure S147. HRMS spectrum of **6i**.

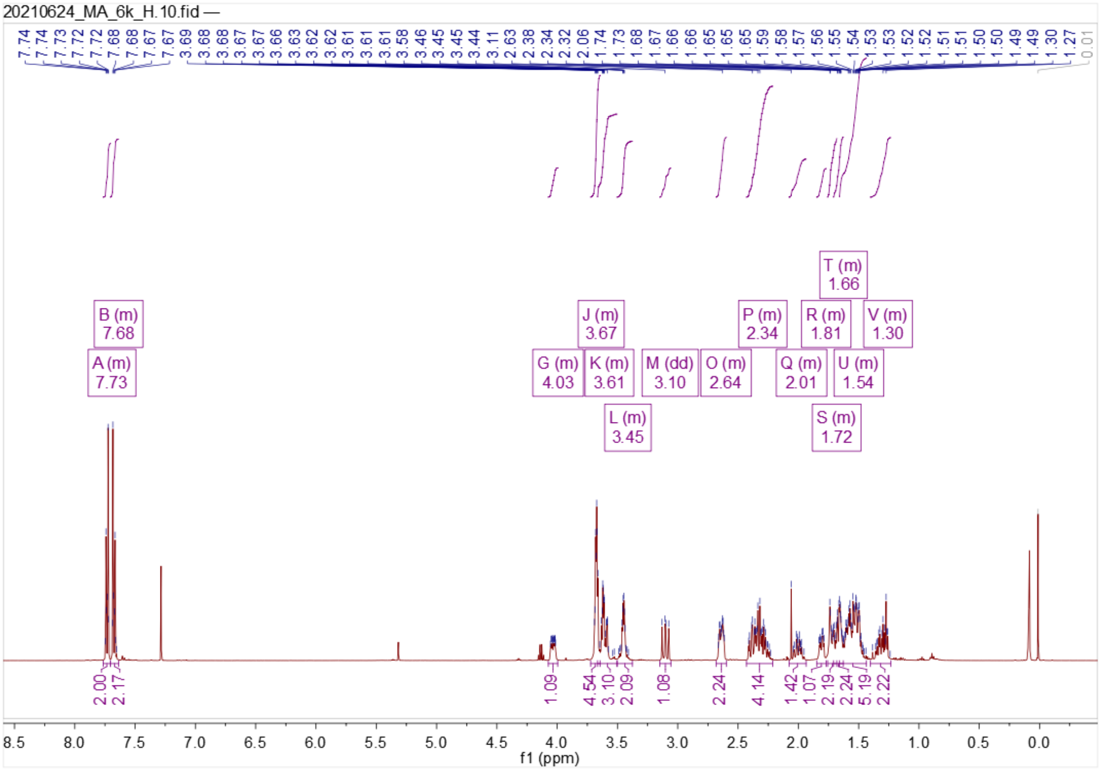

Figure S148. ^{1}H NMR spectrum (Chloroform-d, 500 MHz) of **6j**.

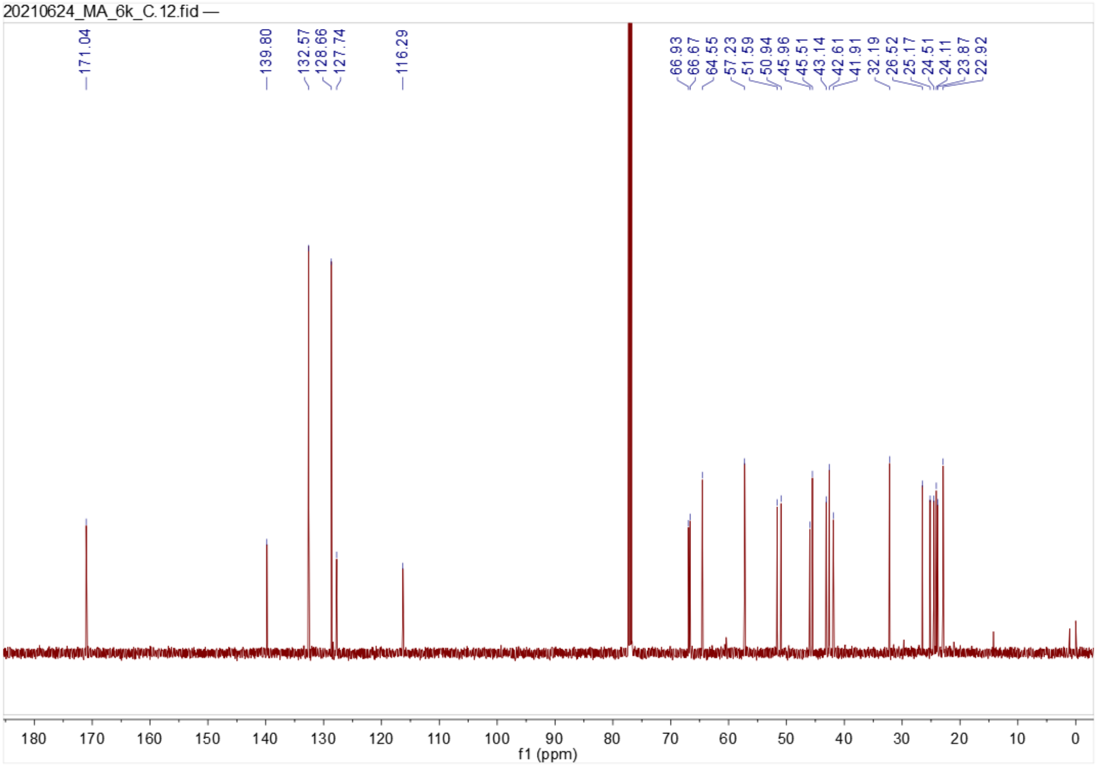

Figure S149. ^{13}C NMR spectrum (Chloroform-*d*, 126 MHz) of **6j**.

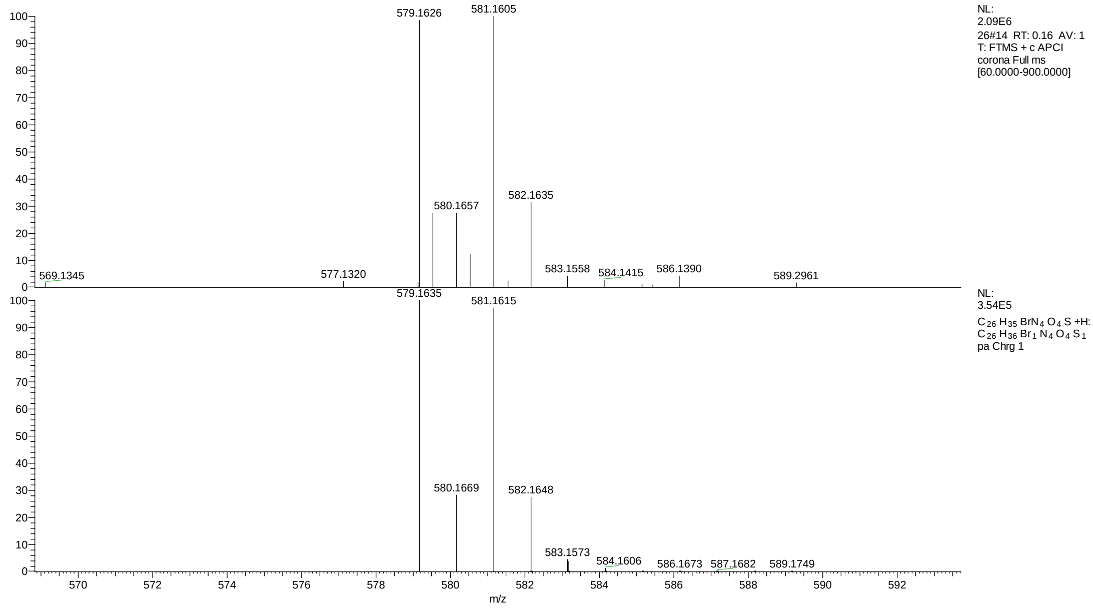

Figure S150. HRMS spectrum of **6j**.

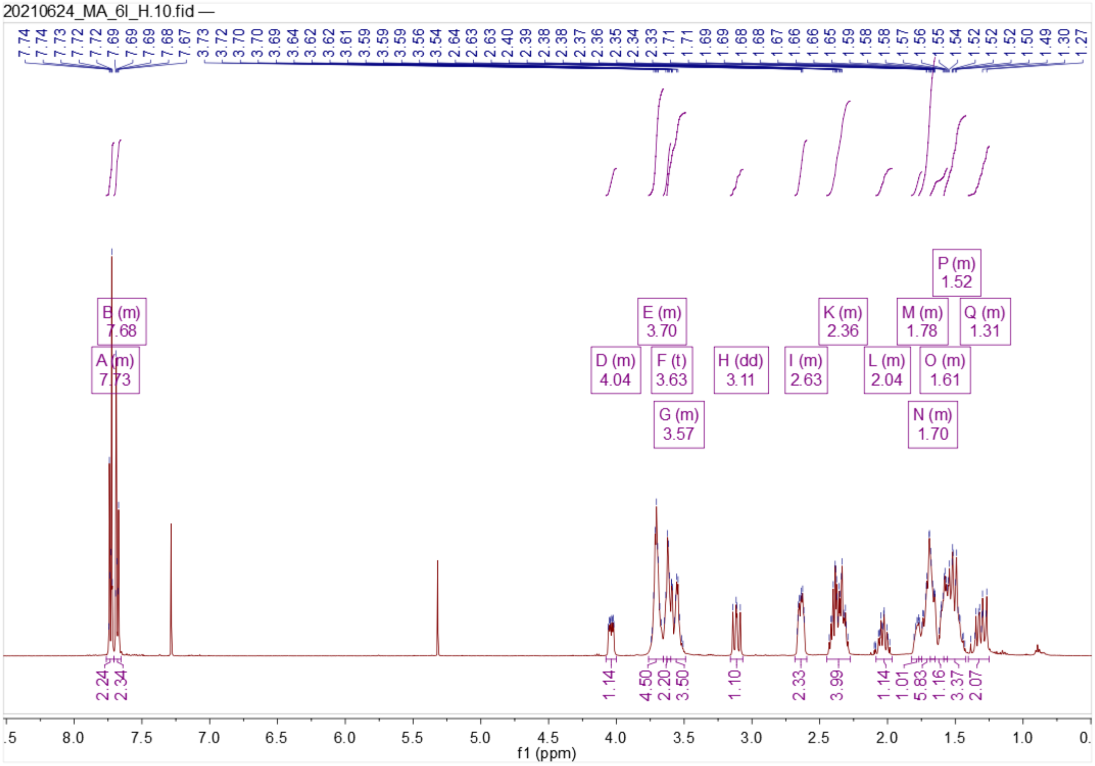

Figure S151. ^{1}H NMR spectrum (Chloroform-*d*, 500 MHz) of **6k**.

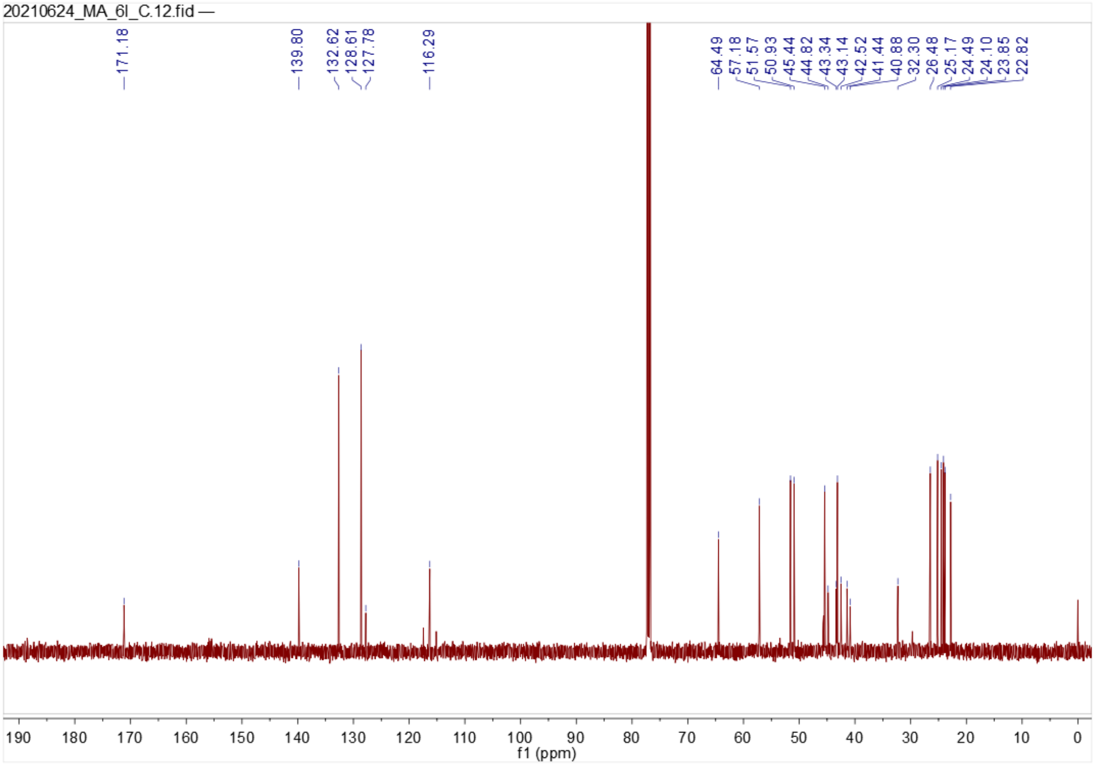

Figure S152. ^{13}C NMR spectrum (Chloroform-*d*, 126 MHz) of **6k**.

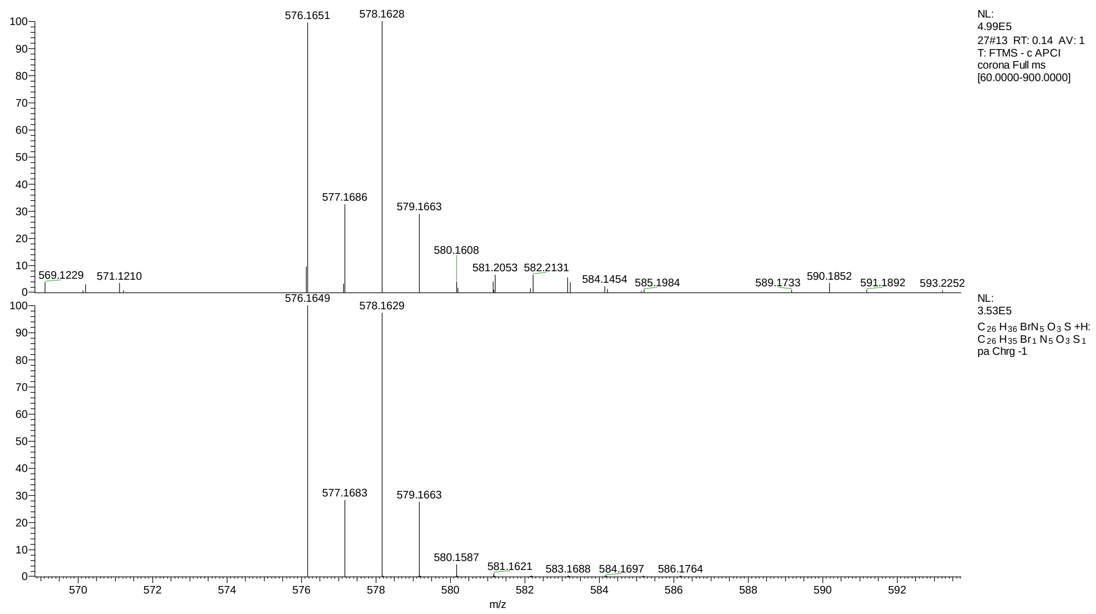

Figure S153. HRMS spectrum of **6k**.

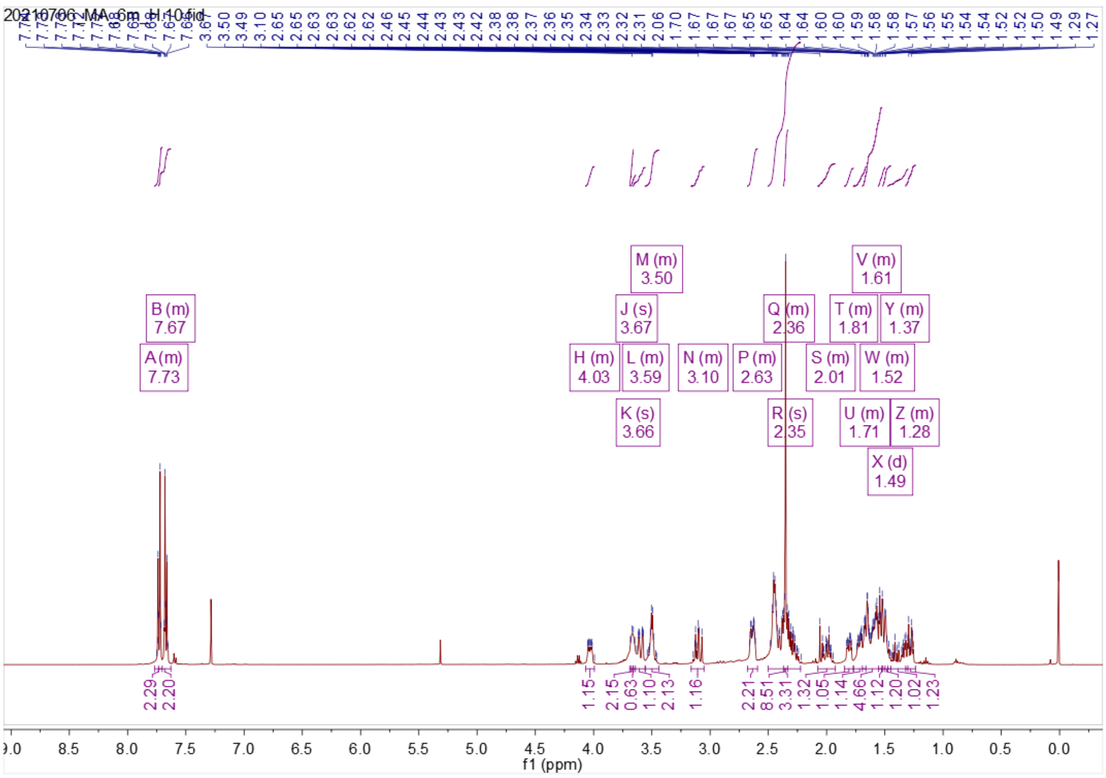

Figure S154. ^{1}H NMR spectrum (Chloroform-*d*, 500 MHz) of **6l**.

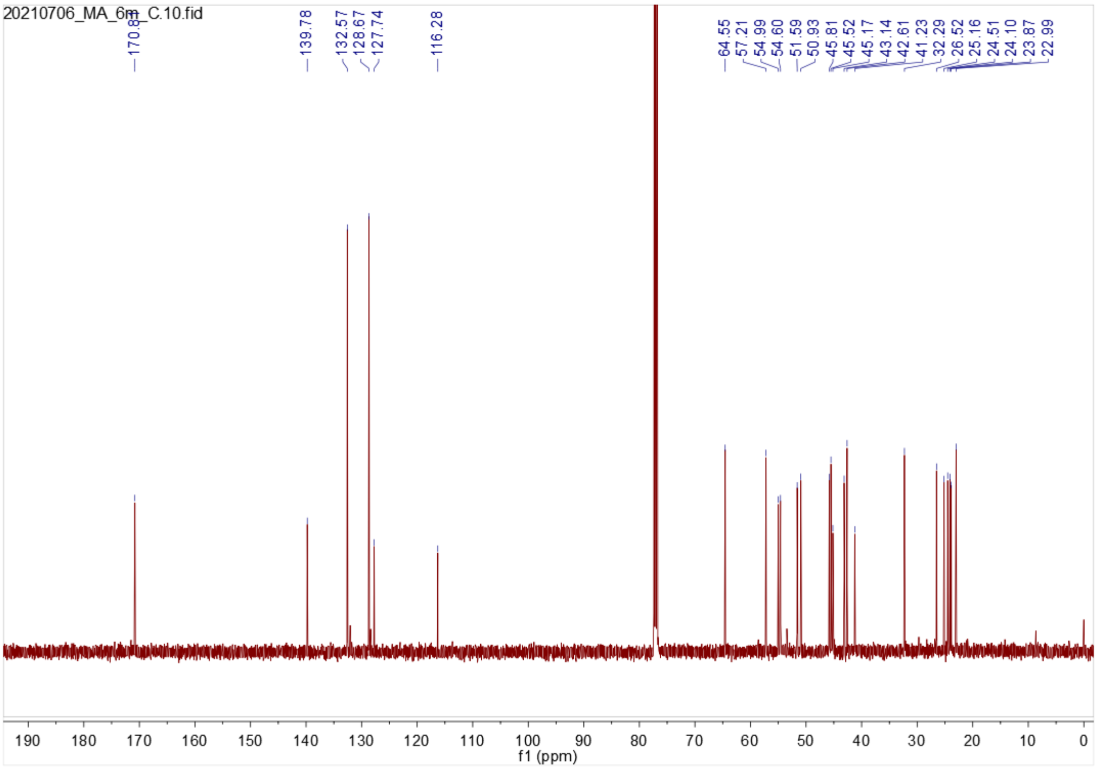

Figure S155. ^{13}C NMR spectrum (Chloroform-*d*, 126 MHz) of **6l**.

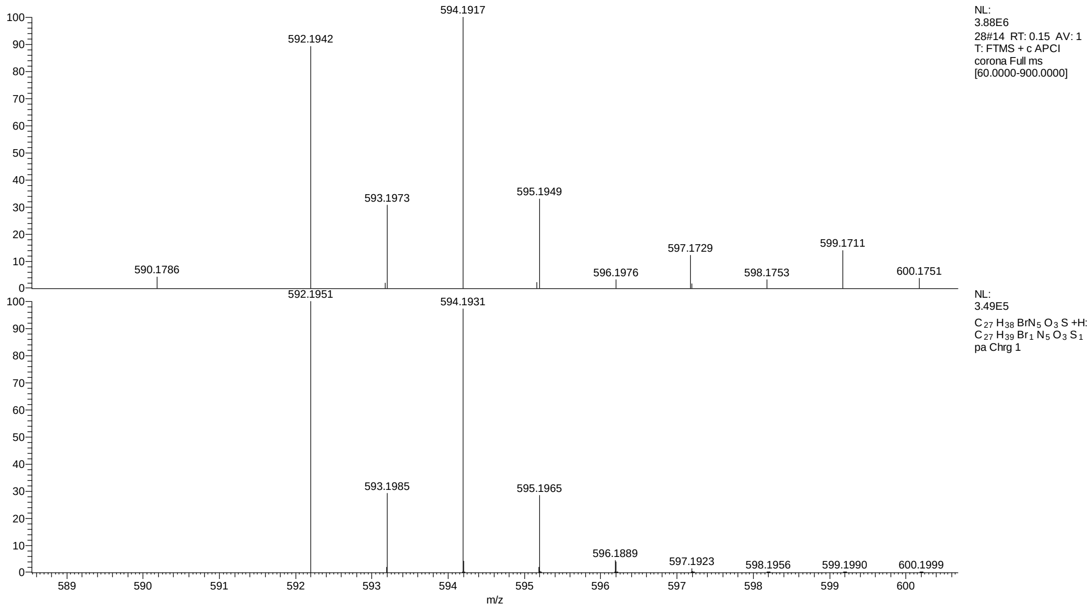

Figure S156. HRMS spectrum of **6l**.

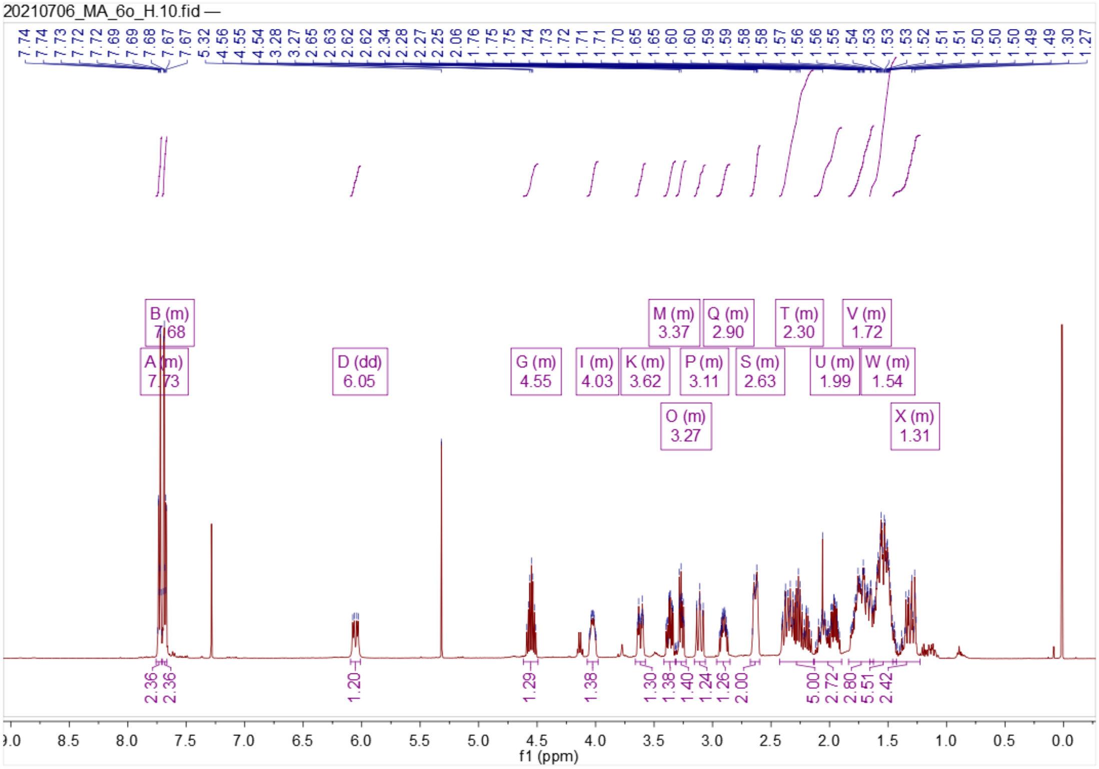

Figure S157. ^{1}H NMR spectrum (Chloroform-*d*, 500 MHz) of **6m**.

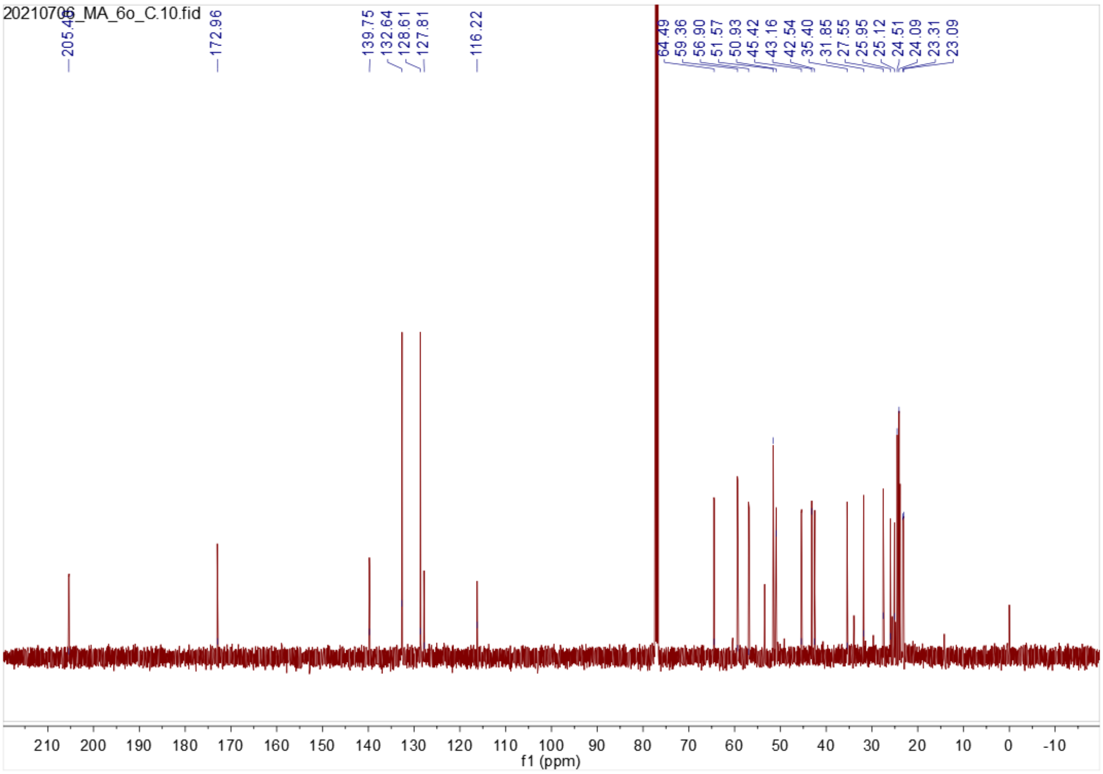

Figure S158. ^{13}C NMR spectrum (Chloroform-*d*, 126 MHz) of **6m**.

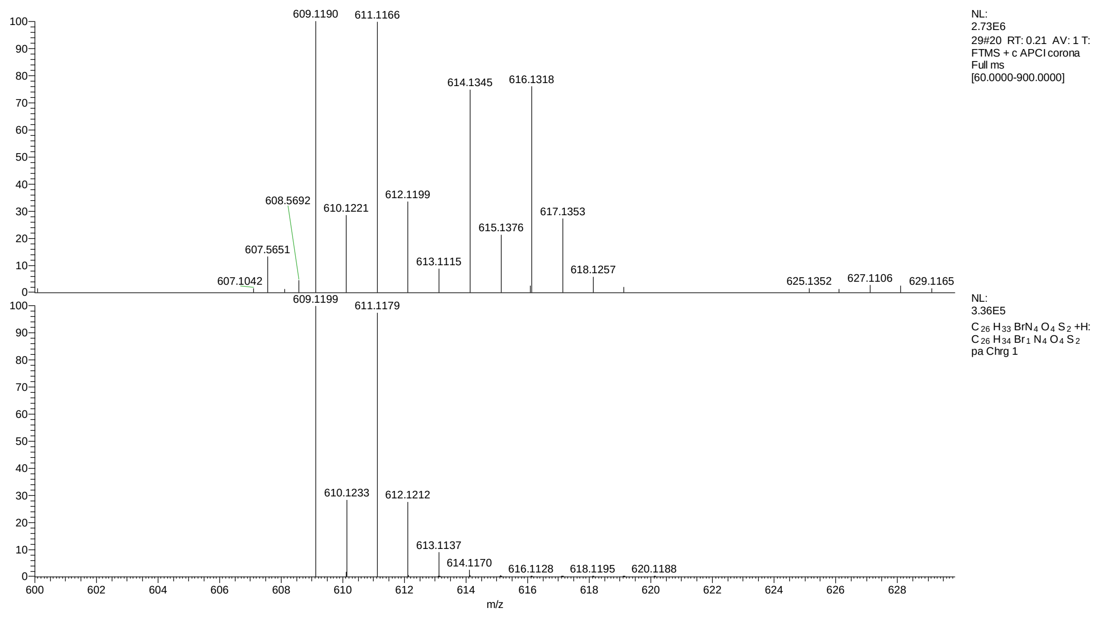

Figure S159. HRMS spectrum of **6m**.

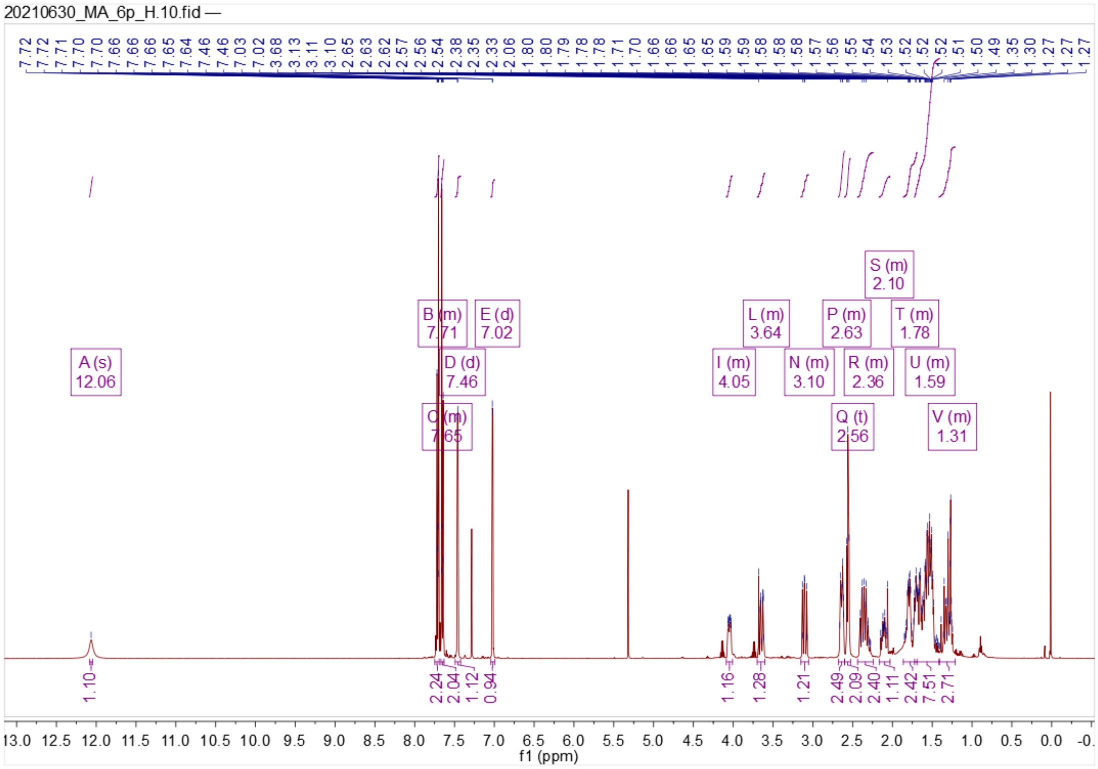

Figure S160. ^{1}H NMR spectrum (Chloroform-*d*, 500 MHz) of **6n**.

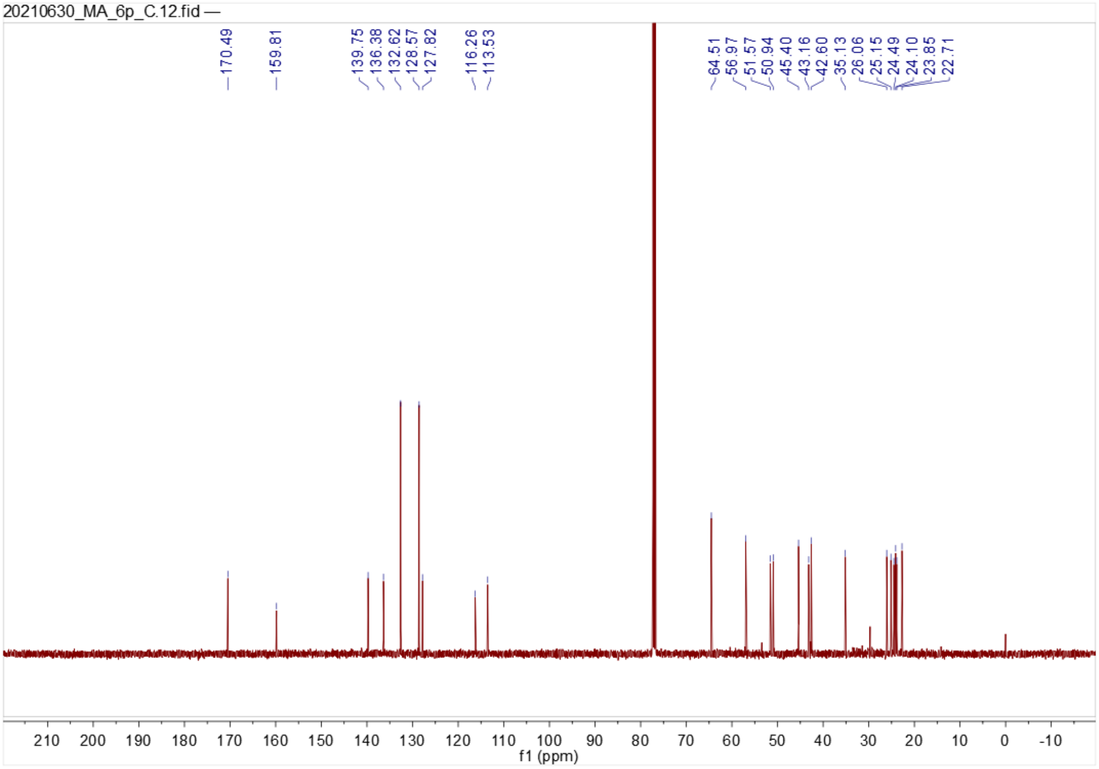

Figure S161. ^{13}C NMR spectrum (Chloroform-*d*, 126 MHz) of **6n**.

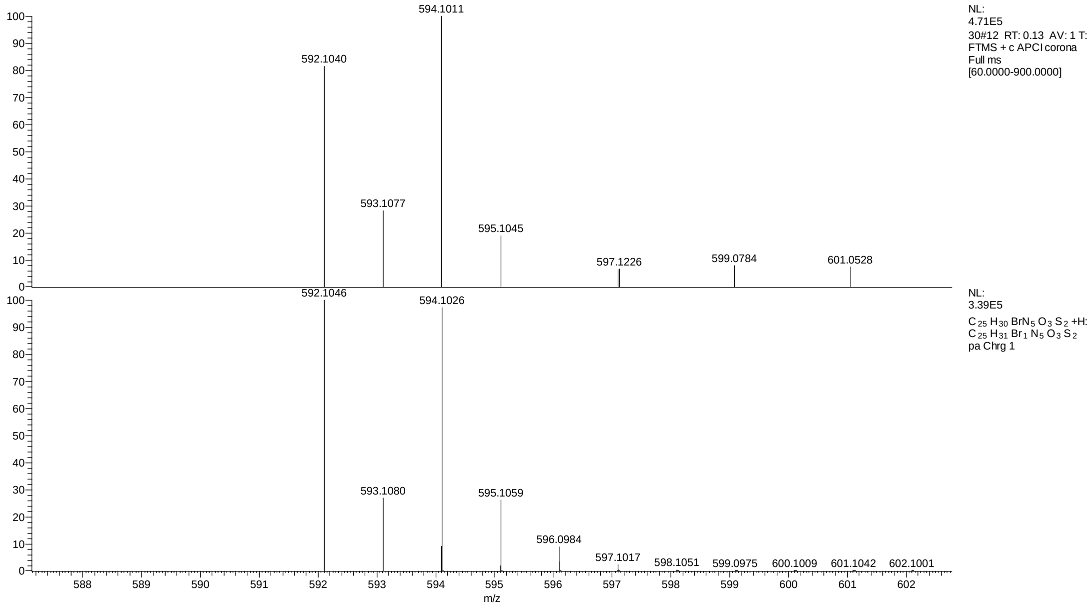

Figure S162. HRMS spectrum of **6n**.

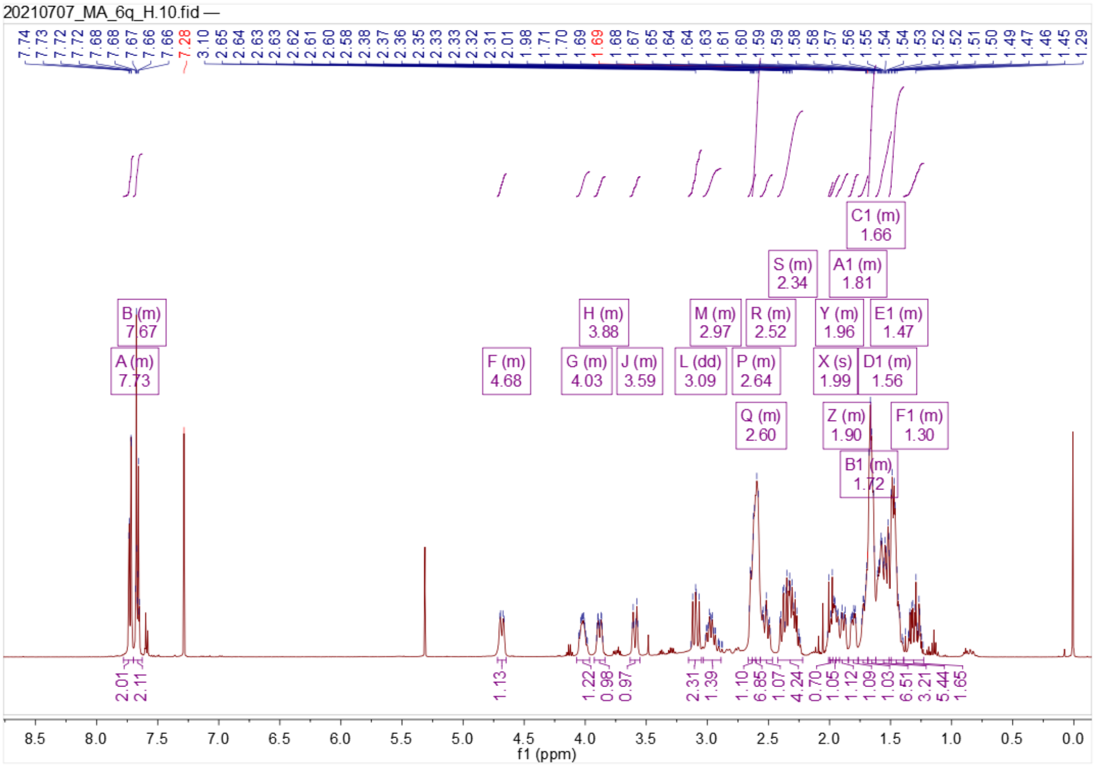

Figure S163. ^{1}H NMR spectrum (Chloroform-*d*, 500 MHz) of **6o**.

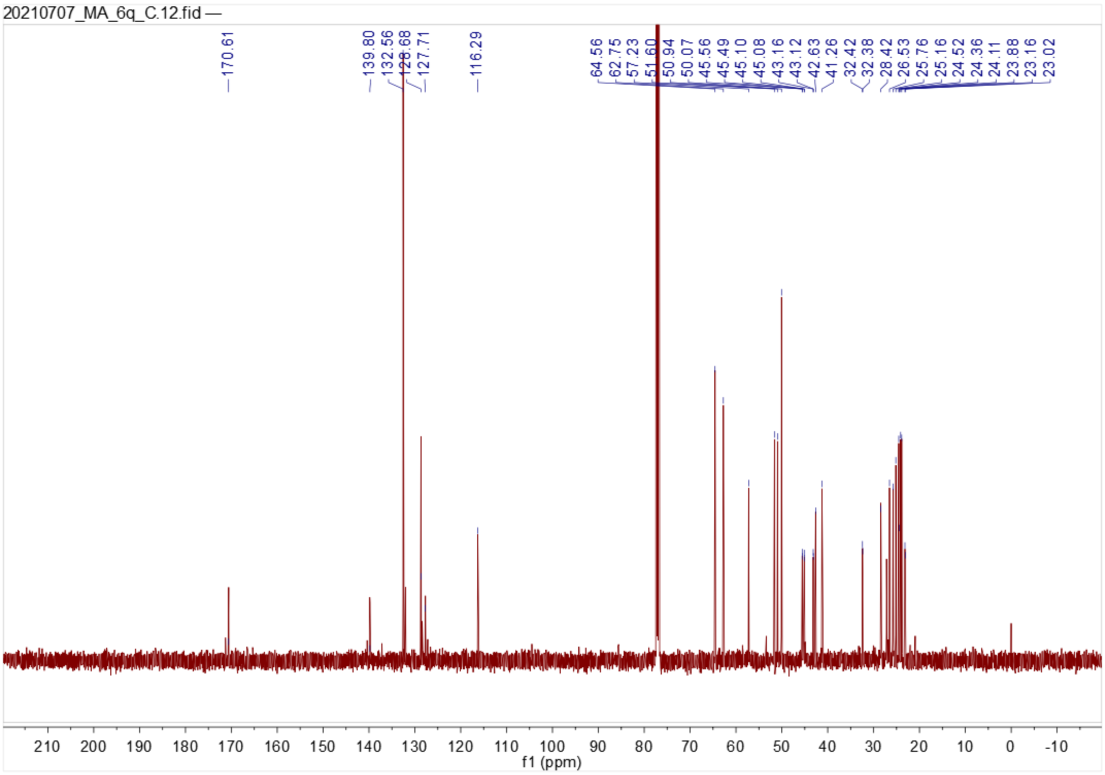

Figure S164. ^{13}C NMR spectrum (Chloroform-*d*, 126 MHz) of **6o**.

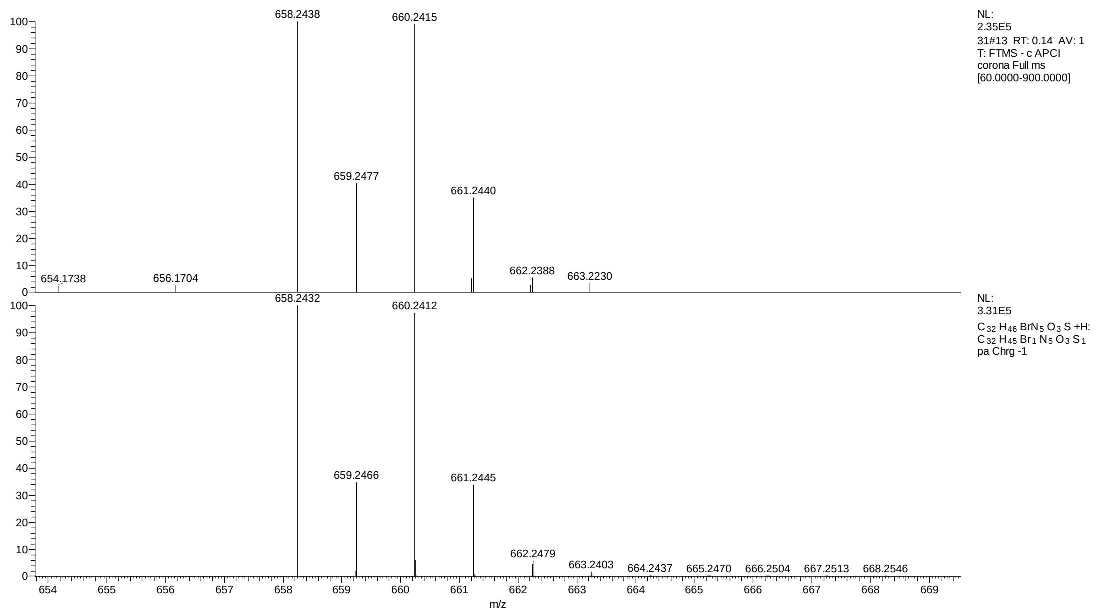

Figure S165. HRMS spectrum of **6o**.

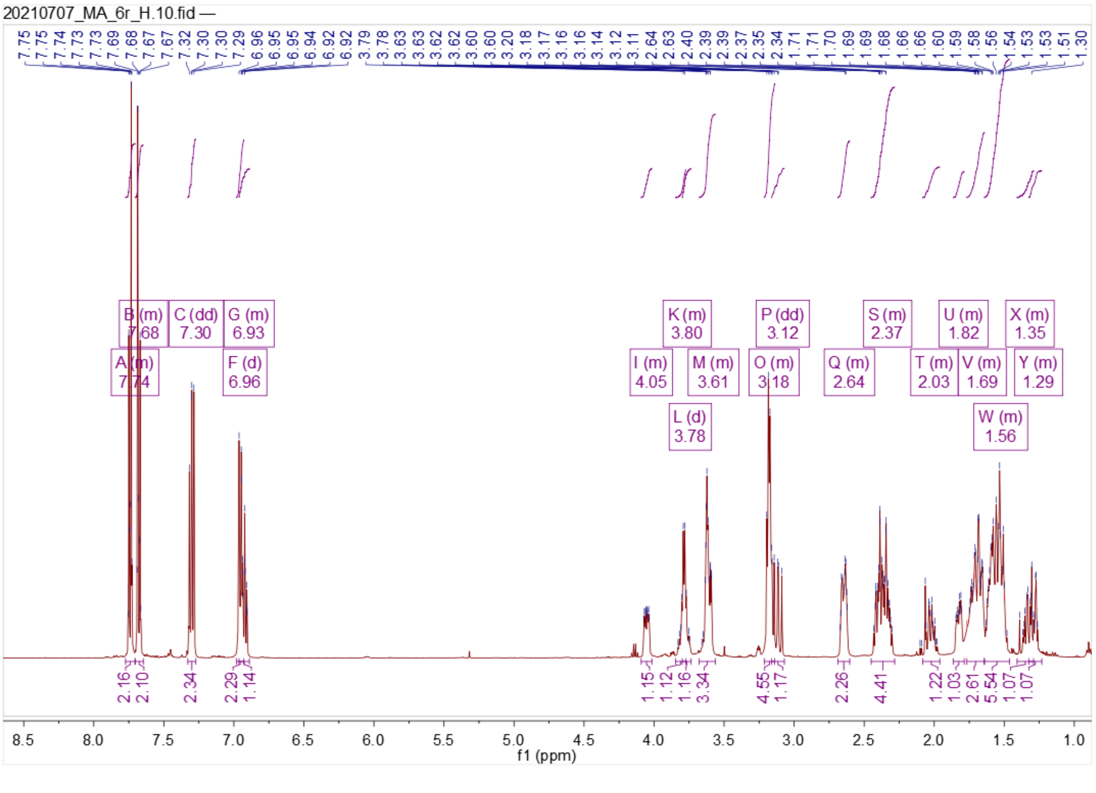

Figure S166. ^{1}H NMR spectrum (Chloroform-*d*, 500 MHz) of **6p**.

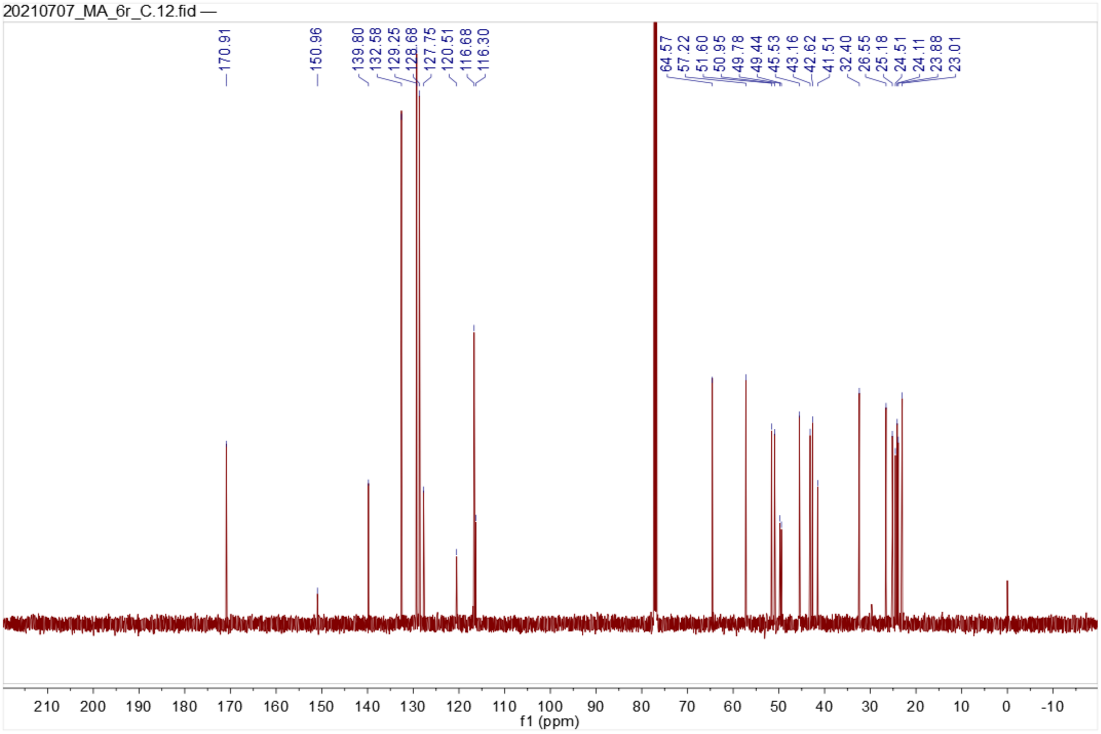

Figure S167. ^{13}C NMR spectrum (Chloroform-*d*, 126 MHz) of **6p**.

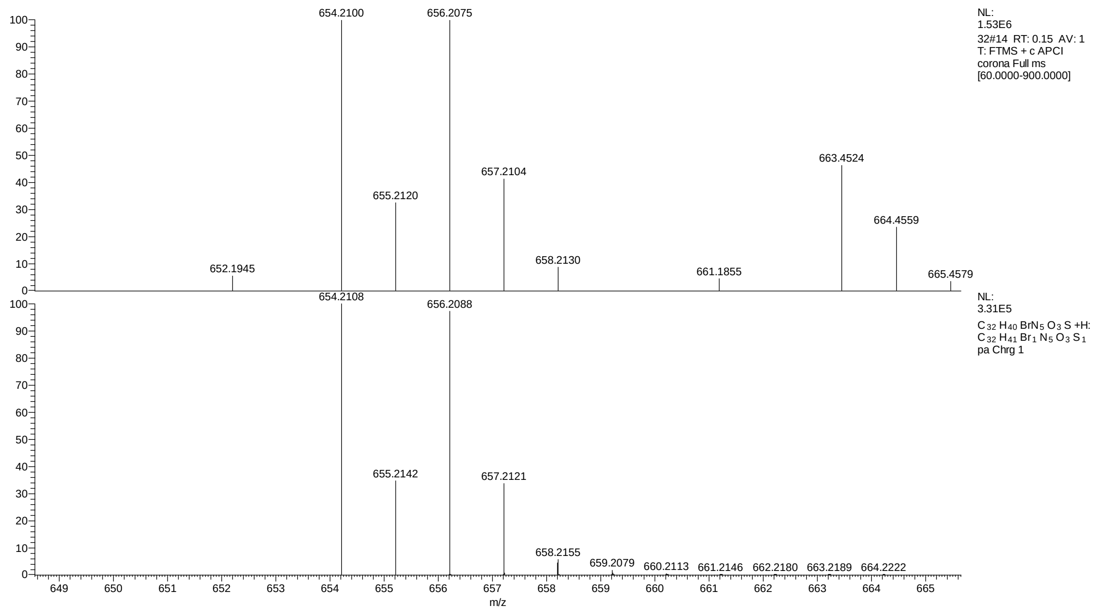

Figure S168. HRMS spectrum of **6p**.
